# Interventions that have potential to help older adults living with social frailty: a systematic scoping review

**DOI:** 10.1186/s12877-024-05096-w

**Published:** 2024-06-15

**Authors:** Monika Kastner, Isabella Herrington, Julie Makarski, Krystle Amog, Tejia Bain, Vianca Evangelista, Leigh Hayden, Alexa Gruber, Justin Sutherland, Amy Sirkin, Laure Perrier, Ian D. Graham, Michelle Greiver, Joan Honsberger, Mary Hynes, Charlie Macfarlane, Leela Prasaud, Barbara Sklar, Margo Twohig, Barbara Liu, Sarah Munce, Sharon Marr, Braden O’Neill, Alexandra Papaioannou, Bianca Seaton, Sharon E. Straus, Katie Dainty, Jayna Holroyd-Leduc

**Affiliations:** 1 North York General Hospital, Toronto, Ontario Canada; 2 Institute of Health Policy, Management and Evaluation, University of Toronto, Toronto, Ontario Canada; 3 Department of Family and Community Medicine, University of Toronto, Toronto, Ontario Canada; 4 University of Ottawa, Ottawa, Ontario Canada; 5 Sunnybrook Hospital, Toronto, Ontario Canada; 6 The KITE Research Institute, Toronto Rehabilitation Institute, University Health Network, Toronto, Ontario Canada; 7 Division of Geriatric Medicine, Department of Medicine, McMaster University, Hamilton, Ontario Canada; 8 St. Michael’s Hospital, Unity Health, Toronto, Ontario Canada; 9 Department of Medicine, University of Toronto, Toronto, Ontario Canada; 10 University of Calgary, Calgary, Alberta Canada

**Keywords:** Older adults, Social frailty, Geriatrics, Scoping review, Self-management, Information communication technology

## Abstract

**Background:**

The impact of social frailty on older adults is profound including mortality risk, functional decline, falls, and disability. However, effective strategies that respond to the needs of socially frail older adults are lacking and few studies have unpacked *how* social determinants operate or how interventions can be adapted during periods requiring social distancing and isolation such as the COVID-19 pandemic. To address these gaps, we conducted a scoping review using JBI methodology to identify interventions that have the best potential to help socially frail older adults (age ≥65 years).

**Methods:**

We searched MEDLINE, CINAHL (EPSCO), EMBASE and COVID-19 databases and the grey literature. Eligibility criteria were developed using the PICOS framework. Our results were summarized descriptively according to study, patient, intervention and outcome characteristics. Data synthesis involved charting and categorizing identified interventions using a social frailty framework.

**Results:**

Of 263 included studies, we identified 495 interventions involving ~124,498 older adults who were mostly female. The largest proportion of older adults (40.5%) had a mean age range of 70-79 years. The 495 interventions were spread across four social frailty domains: social resource (40%), self-management (32%), social behavioural activity (28%), and general resource (0.4%). Of these, 189 interventions were effective for improving loneliness, social and health and wellbeing outcomes across psychological self-management, self-management education, leisure activity, physical activity, Information Communication Technology and socially assistive robot interventions. Sixty-three interventions were identified as feasible to be adapted during infectious disease outbreaks (e.g., COVID-19, flu) to help socially frail older adults.

**Conclusions:**

Our scoping review identified promising interventions with the best potential to help older adults living with social frailty.

**Supplementary Information:**

The online version contains supplementary material available at 10.1186/s12877-024-05096-w.

## Background

By the year 2050, two billion people worldwide will be 60 years of age and older [1, 2]. In Canada, the prevalence of frailty in those age 65+ years is almost twice that of those age 50-64 years [3]. The association of older age with increased prevalence of frailty may be due to the accumulation of multiple risk factors over time [3] and includes physical, psychological and social dimensions [4–6]. As such, frailty is considered “*a syndrome that affects biological, psychological, and social processes of a person’s life and leads to increased vulnerability and adverse outcomes in old age*” [7, 8]. Frailty has traditionally been conceptualized as a physiological phenomenon, and related assessment tools often emphasize *physical* qualities such as multimorbidity, nutrition, and functional independence [9]. In social frailty, it is a person’s social activities, social supports, social networks, loneliness and whether they are living alone that are involved [10]. Social frailty has been identified as a risk to healthy aging and defined as: *“a continuum of being at risk of losing, or having lost, social and general resources, activities or abilities that are important for fulfilling one or more basic social needs during the life span”* [11]. Social frailty is associated with other related and overlapping concepts [12–15] such as loneliness, social isolation, social vulnerability and resilience, which highlights that we cannot investigate social frailty in isolation. We need to understand not only how these social frailty concepts operate within a person’s physical and psychological health state, but what strategies are the most helpful during stressful periods when this population becomes more vulnerable (e.g., COVID-19 or other infectious disease outbreaks such as the flu).

As adults get older, their physical and cognitive capacity can decrease [16], which can progressively decrease their social activities, social circles, and increase the likelihood of living alone [17] – all these factors can lead to social isolation and social frailty [17]. The prevalence of social frailty varies widely and estimated to be between 7.7% to 47% (socially frail), and between 25% to 32.1% (pre-socially frail) [18–20]. During the first three years of the COVID-19 pandemic (2020-2022), social frailty prevalence rose to 18-25% among community-dwelling older adults [20, 21] and 47.3% in hospital [20], which was largely attributed to lockdown measures and quarantine leading to increased social isolation and loneliness among older adults. The impact of social frailty on older adults is profound. It is associated with the risk of all-cause mortality [22], physical and cognitive decline [18] including the onset of Alzheimer’s disease [23], reduced social and psychological well-being [17], depression and anxiety [24], moderate hearing loss [25], and decreased quality of life [26]. As such, social frailty represents one of the greatest challenges to the care for older adults and the health care system today. However, very little is known about effective strategies that can address social frailty.

**Fig. 1:**
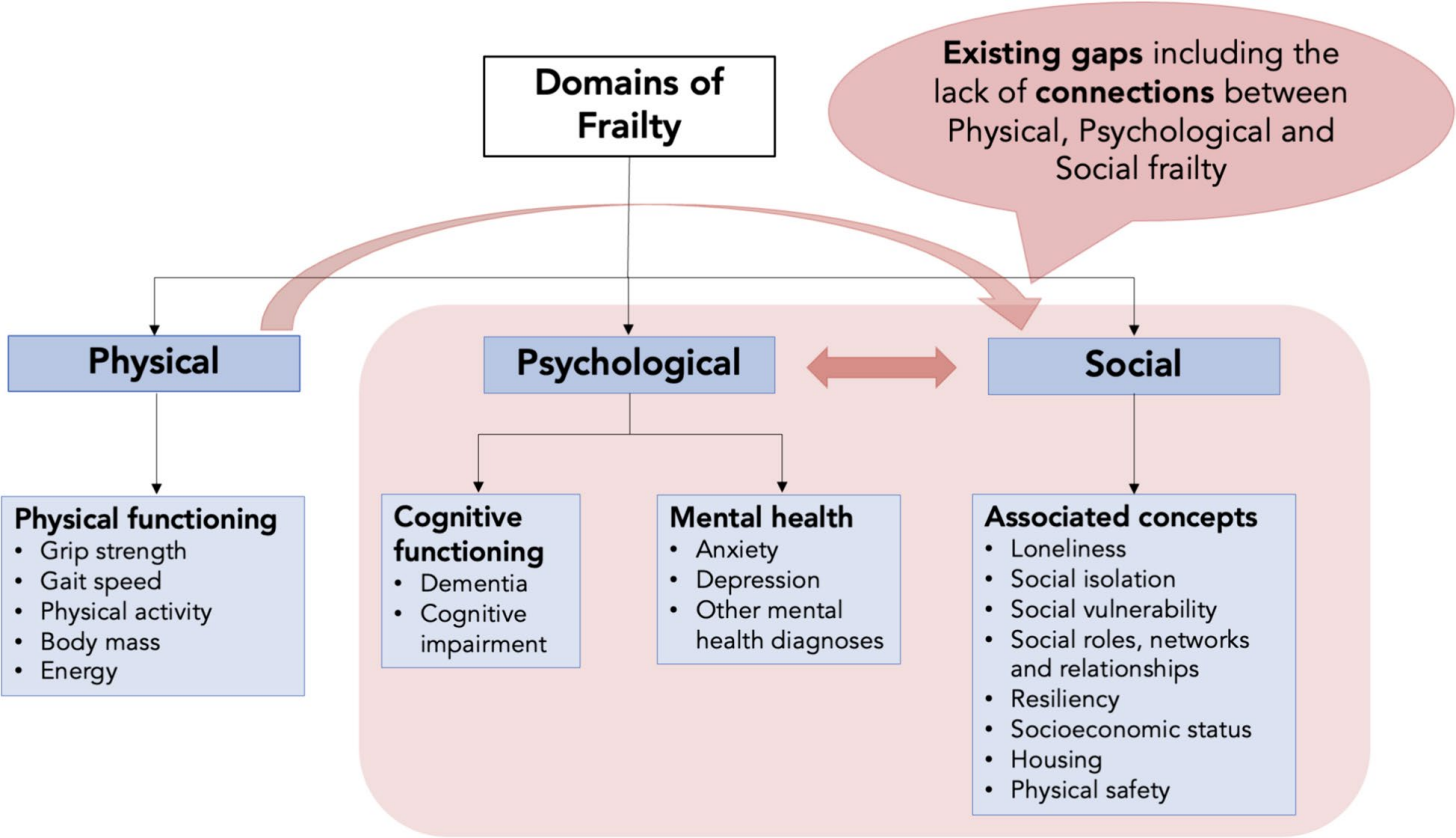
Domains of frailty and existing gaps

Many existing knowledge syntheses focus on the clinical aspects of frailty (i.e., physical and psychological) or only on social isolation, vulnerability or loneliness [27–30]. Very few studies consider how social factors intersect with physical or psychological frailty [31, 32] (Fig. 1). Increasingly, the physical, social and cognitive problems experienced by older adults are being considered as part of psychological frailty, but their differences and associations are not well understood in the context of overall frailty [31].

Existing reviews also have not identified how interventions can address social frailty or related concepts (e.g., social isolation, loneliness, resilience; poverty risk; housing; physical safety; and social roles, networks, and relationships). Furthermore, little is known about which social frailty interventions have potential to be adapted for use during social distancing and isolation measures due to infectious disease outbreaks such as the COVID-19 pandemic. The pandemic response had indirect consequences on older adults as these measures led to an even greater risk of morbidity and mortality and reduced physical, mental and social wellbeing [33–35]. To address these gaps, our objectives were to:

1. generate knowledge about which interventions have the best potential to help older adults (age ≥65 years) living with or at risk for social frailty or related concepts (e.g., social isolation, loneliness); and
2. to identify interventions that may be the most helpful (and can be adapted) during infectious disease outbreaks requiring isolation (e.g., the COVID-19 pandemic, flu) when older adults are at even greater risk to become social frail.

## Methods

### Study design

We conducted a scoping review using the JBI methodology [36]. Our protocol was registered with the Open Science Framework (OSF) (registration: 10.17605/OSF.IO/2MDQF). We used the PRISMA extension for scoping reviews (PRISMA-ScR) [37] to prepare this manuscript. Our integrated knowledge translation (IKT) team contributed to the design and conduct of our scoping review. Our IKT team included knowledge users with expertise in social frailty (clinicians), scoping review methods (clinicians, researchers), patient partners with lived experience of social frailty and their caregivers. Our patient partners contributed to refining our research objectives, provided feedback on our data gathering tools, screened articles and helped to interpret findings.

### Search strategy

An experienced information specialist (librarian) developed the search strategy, and a second information specialist appraised this strategy using the Peer Review of Electronic Search Strategies (PRESS) checklist [38]. We searched MEDLINE, CINAHL (EPSCO), EMBASE and COVID-19 databases (medRxiv and LitCovid) in English from 2000 to October 29, 2023, using a validated, age-specific search filter [39] to focus our search on studies on the older adult population (Supplement file 1). The search strategy utilized a combination of controlled vocabulary (e.g., “Social Isolation”, “Loneliness”, “Social Alienation”) and keywords (e.g., “connectedness”, “social vulnerability”, “aloneness”). We also searched the grey literature using the Canadian Agency for Drugs and Technologies in Health (CADTH) Grey Matter approach [40], including the sources of the Centre for Evidence-Based Medicine, Oxford COVID-19 Evidence Service.

### Eligibility criteria

Our draft eligibility criteria were defined by our IKT team (including our patient partners) and developed using the PICOS framework [41]: *Population*: Community-dwelling older adults (age ≥ 65 years) at risk for or living with social frailty with or without concurrent disease(s) (e.g., diabetes). We excluded interventions targeting older adults living in long-term care homes (i.e., nursing home) or admitted to the hospital. *Intervention:* Any intervention addressing social frailty or related concepts (loneliness, social isolation, social vulnerability). Interventions that focused *only* on physical or psychological frailty were excluded. *Outcomes:* Social frailty or related concepts as reported by included studies (e.g., social support, social participation, social networks, social frailty index, loneliness score). We also considered quality of life (QOL), functional status (social), and general well-being (overall, social) outcomes. *Study design*: Any qualitative, quantitative, mixed-methods, multi-methods studies and systematic knowledge syntheses and excluded opinion driven reports (e.g., commentaries, editorials).

### Article selection and extraction

Experienced reviewer pairs (IH, JH, JM, JS, KA, LH, LP, MM, MK) screened all potentially eligible records in duplicate for title and abstract screening and a verification process for full-text screening (one reviewer screened articles, and another verified 10% of these records). Reviewer pairs were calibrated at each screening level to ensure screening reliability (i.e., screening the same 10% set of articles by all reviewers until they reached ≥80% agreement, after which they screened independently). Our patient partners were also invited to screen articles. Any disagreements were resolved through team discussions.

### Data charting

We developed and pilot tested a standardized data abstraction form, which was iteratively revised until data abstractor pairs reached consensus on data items. Experienced reviewer pairs (IH, JS, KA, MK, JH, LP, LH, MH) abstracted data and second reviewers checked a 10% random sample of data on key study characteristics (study design, journal, year and country of conduct), population characteristics (age, sex, gender, race) and intervention characteristics: type, mode of delivery (in-person, virtual), level of interaction with the intervention (one-to-one, group-based, self-directed, pair-based or mixed); and outcomes. Although not recommended by the JBI guide [42], abstracting outcome results was relevant for this review to identify interventions that could support knowledge user decision making about social frailty interventions, and how they might be adapted during infectious disease outbreaks. We also wanted to identify which intervention should be investigated in a future systematic review.

### Data analysis and synthesis

Our results were summarized descriptively according to study, patient and intervention characteristics using tables and appendices. In our data analysis and reporting, all data was disaggregated by sex and gender if reported in included studies. To organize and categorize identified interventions, we used Bunt *et al*’s social frailty framework [11], which highlights that the concept of social frailty needs to include not only the absence (or threat) of general and social resources that are needed to fulfill basic social needs but also the absence (or threat) of social behaviours and social activities as well as self-management abilities [11]. As such, we categorized identified interventions according to Bunt *et al*’s four broad domains of social frailty: (i) General resource related (“*Resources that are beneficial generally, indirectly contributing to social need fulfilment*” such as education, financial status, housing, living environment, basic activities of daily living, cognition, lifestyle, life events); (ii) Social resource related (“*Resources that are likely to directly contribute to the fulfilment of one of more social needs* “such as marital status, family ties, living children, social network size, care or help from others) [43]; (iii) Self-management (to improve an individual’s ability to manage their behaviours and emotions to benefit their overall health [44]); and (iv) Social behavioural activities (*“social behaviours or activities that are performed towards social need fulfilment”* such as maintenance of close relationships, social participation, volunteerism, religiosity, occupation, neighborhood involvement) [43]. The intervention categorization process involved three reviewers (IH, KA, MK), iteratively developing a codebook and definitions using content analysis [45]. If interventions were represented by overlapping domains, we categorized interventions based on the predominant component via team discussion. We used a similar procedure to organize an expected large volume of outcomes and outcome categories from our studies. If reported, we also considered differences between participants of included studies in their social frailty risk (sex, gender, advanced age, low educational level and socioeconomic status and housing) as well as how they experience social frailty [10]. To classify loneliness, social and health and well-being outcomes in identified interventions, reviewer pairs (IH, JM, KA, MK) iteratively created a codebook using content analysis [45]. Our patient partners (BL, CM, JH, LP, MH, MT) had opportunities to review and provide feedback on these documents. Interventions were classified as “effective” if they reported outcome(s) as “effective” or “statistically significant”. We used the following effect direction definitions to classify all intervention result statements: “+*” = statistically significant positive impact; “-*” = statistically significant negative impact; “+” = positive direction in impact; “-“ = negative direction in impact; “+/-“ = mixed impact; NC = no change; NA = not applicable. One reviewer (IH) applied the effect direction classification scheme, and another reviewer (MK) audited a 10% random sample of classifications. We also identified effective interventions that may be helpful during infectious disease outbreaks such as the COVID-19 pandemic. This was based on whether interventions required participants to have any physical contact or in-person interaction with another individual (i.e., whether the intervention was feasible to be delivered virtually or remotely).

## Results

### Literature search

**Fig. 2:**
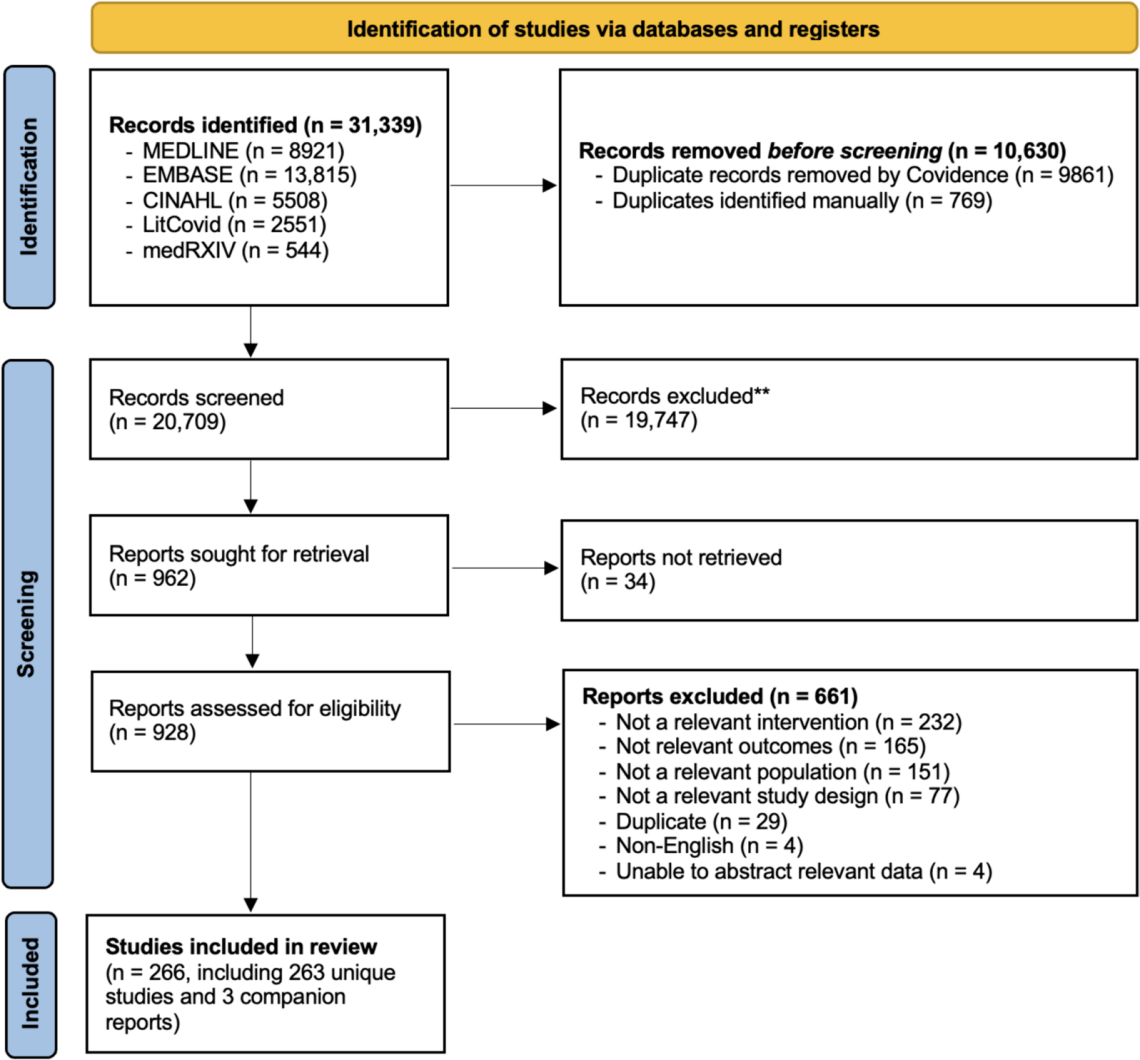
PRISMA Flowchart

Of 31,339 records that were identified by our search strategy, we screened 20,709 titles and abstracts and 928 full-text articles. Of these, 263 studies plus 3 companion reports met the eligibility criteria and were included in our scoping review (Fig. 2).

### Study characteristics

**Fig. 3:**
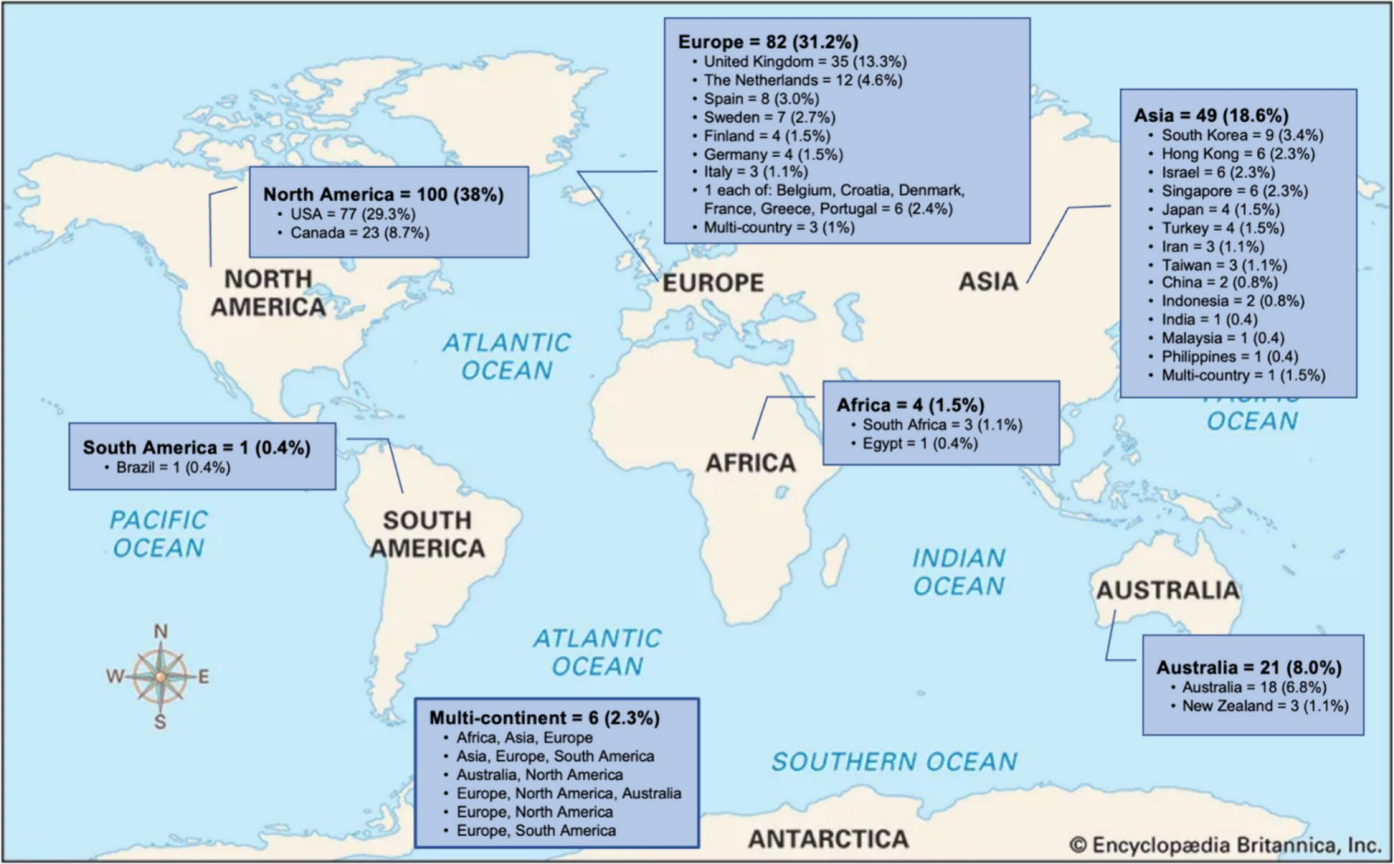
Country and continent of conduct*. *Percentages may not total to 100 due to rounding

Table 1 includes the study characteristics. Of the 263 included studies, 57% were published between 2021 and 2023 followed by 31%, which were published between 2016-2020 with the earliest published in 2000. Most studies were conducted in North America (38%), followed by Europe (31%), Asia (19%%) and Australia (8%) (Fig. 3). The study designs of included studies were quasi-experimental (23%), qualitative (19%), experimental (19%) including 43 randomized controlled trials (16%), knowledge synthesis (19%), mixed- or multi-methods (16%), cross-sectional (2.7%), observational (1.5%) and cost or economic analysis (0.4%).

**Table 1:** Summary of study, participant and intervention characteristics^a^

**Study characteristics**	
**Characteristic (number of interventions)**	**Number of studies (%)**
**Continent of conduct (*n*=263)**	
North America	100 (38.0)
Europe	82 (31.2)
Asia	49 (18.6)
Australia	21 (8.0)
Africa	4 (1.5)
South America	1 (0.4)
Multi-continent	6 (2.3)
**Year of publication (*n*=263)**	
2021-2023	151 (57.4)
2016-2020	81 (30.8)
2011-2015	17 (6.5)
2006-2010	10 (3.8)
2001-2005	3 (1.1)
2000	1 (0.4)
**Study design (*n*=263)**	
Quasi-experimental	60 (22.8)
Qualitative	50 (19.0)
Experimental	49 (18.6)
RCT	43 (16.3)
Prospective controlled trial	3 (1.1)
Controlled before and after	2 (0.8)
Experimental pilot study	1 (0.4)
Knowledge synthesis	49 (18.6)
Mixed- or multi-methods	43 (16.3)
Cross-sectional	7 (2.7)
Observational	4 (1.5)
Cost or economic analysis	1 (0.4)
**Participant characteristics**	
**Characteristic (number of interventions)**	**Number of interventions (%)**
**Mean age range** **(*n****=***385)**	
50-59	4 (1.0)
60-69	64 (16.6)
70-79	156 (40.5)
80-89	37 (9.6)
Unclear	124 (32.2)
**Sex** (***n***=**309)**	
Majority female participants	273 (88.3)
Majority male participants	27 (8.7)
Equal distribution of sex	9 (2.9)
**Gender** **(*n****=***2)**	
Transgender	2 (0.4)
**Race or ethnicity** **(*n*=83)**	
White	71 (85.5)
Black	44 (53.0)
Latinx	28 (33.7)
Asian undefined *(i.e., did not specify region of Asia)*	15 (18.1)
Indigenous	6 (7.2)
Non-white	4 (4.8)
East Asian	3 (3.6)
Southeast Asian	3 (3.6)
South Asian	2 (2.4)
Multiracial/mixed race	4 (4.8)
Not disclosed *(i.e., participant declined to report race)*	3 (3.6)
Unable to determine *(i.e., incomplete or undefined data based on study reporting)*	38 (45.8%)
**Intervention characteristics**	
**Intervention categories and sub-categories** **(*n*=495)**	**Number of interventions (%)**
**Social resource-related interventions**	196 (39.6)
Information Communication Technology (ICT)-based interventions	41 (8.3)
Intergenerational interventions	33 (6.7)
Aging in place interventions	31 (6.3)
Socially assistive robots and computer agents	29 (5.9)
Befriending interventions	27 (5.5)
Peer support interventions	23 (4.6)
Mentorship interventions	11 (2.2)
General social support interventions	1 (0.2)
**Self-management related interventions**	157 (31.7)
Self-management education interventions	106 (21.4)
Psychological self-management interventions	43 (8.7)
Social prescribing or asset-based interventions	8 (1.6)
**Social behavioural activity related interventions**	140 (28.3)
Leisure activity interventions	48 (9.7)
Physical activity interventions	48 (9.7)
Arts-based interventions	27 (5.5)
Mind-body interventions	14 (2.8)
Spiritual interventions	3 (0.6)
**General resource interventions**	2 (0.4)
**Mode of intervention delivery** **(*n****=***435)**	
**In-person**	259 (59.5)
**Virtual ** *(i.e., via computer, telephone, smart devices, video, audio)*	154 (35.4)
**Mixed** *(i.e., in-person and virtual)*	22 (5.1)
**Participant interaction with intervention** **(*n****=***379)**	
**Group-based** *(i.e., delivered as a uniform programme to a group of participants)*	184 (48.5)
**One-to-one** *(i.e., delivered to the participants who interacts solely and directly with an intervention facilitator) independently and do not have regular interactions with others)*	104 (27.4)
**Self-directed** *(i.e., participants navigate the intervention components*	38 (10.0)
**Pair-based** *(i.e., dyadic-based, delivered to two individuals whose social network is linked due to a relationship existing outside of the intervention such as a caregiver and care recipient, a spouse)*	2 (0.5)
**Mixed** *(i.e., combinations of the above)*	51 (13.5)
**Intervention setting** **(*n****=***364)**	
Home	173 (47.5)
Community *(not further specified)*	73 (20.1)
Community centre	19 (5.2)
Assisted living or retirement home	14 (3.8)
Schools or universities	13 (3.6)
Seniors Clubs	13 (3.6)
Community health centre	9 (2.5)
Natural spaces *(e.g., beach, parks, nature reserve, farms)*	6 (1.6)
Day centre	3 (0.8)
Hospital	1 (0.3)
Museum	1 (0.3)
Spiritual centre *(e.g., temple)*	1 (0.3)
Mixed settings	38 (10.4)

^a^For data from knowledge syntheses (*n=*49), only individuals representing the target population were added to the participant count; percentages may not total to 100 due to rounding

### Population characteristics

A total of ~124,498 older adults were included among our 263 included studies representing 90% of the 495 identified interventions. Table 1 includes the participant characteristics. Of the interventions that reported on age (*N*=385; 78%), the largest proportion of older adults (*N=*156; 40.5%) had a mean age range of 70-79 years. Of 115 interventions that reported on participant sex (62%), 273 interventions (88%) included mostly female participants. Two interventions reported on gender (0.4%) and 83 interventions reported on race or ethnicity (17%). Of these, the largest proportion of interventions reported racial groups that self-identified as white (59 of 83 interventions; 71%), black (44 of 83 interventions; 53%), Latinx (28 of 83 interventions; 34%) or Asian including east, southeast and south Asian (23 of 83 interventions; 28%).

### Intervention characteristics

Table 1 includes the intervention characteristics. Most of the 495 identified interventions were represented by three of the four social frailty domains [11]: 196 social resource related (40%), 157 self-management (32%), and 140 social behavioural activity interventions (28%); 2 interventions were identified from the general resource related domain (0.4 %). The largest proportion of interventions sub-categories were self-management education (21%), physical activity (9.7%) and leisure (9.7%) interventions followed by psychological self-management interventions (8.7%) and Information Communication Technology (ICT) (8.3%). The details of these intervention categories and sub-categories are in Supplement file 2. The most common mode of intervention delivery was in-person (59.5%) or virtual (35%). Participants most often interacted with interventions via group-based (48.5%) or one-to-one (27%) strategies. Intervention settings were mostly at home (47.5%) or in the community (20%).

### Outcome characteristics

We identified 10 categories of outcomes (Supplement file 3): *Loneliness* outcomes were investigated by the largest proportion of interventions (*n=*312; 63%) followed by *Social cohesion and connectedness* (*n=*120; 24%), *Quantity of social relationships* (*n=*98; 20%), *Social capital* (*n=*95; 19%), *Health and Wellbeing* (*n=*83; 17%), *Social engagement* (*n=*78; 16%), *Social isolation* (*n=*75; 15%),

*Social functioning and skills* (*n=*37; 7.5%), *Quality of life* (*n=*54; 11%), and *Frailty* (*n=*5; 1%) outcomes. The highest concentration of effective interventions was found to be among loneliness outcomes (*n=*275), which was spread in similar proportion across the three social frailty domains: social behavioural activity (47%), self-management (43%) and social resource-related (42%).

### Narrative synthesis of results

The detailed description of identified interventions and their impact across four social frailty domains are in Tables 2, 3 and 4.

**Table 2:** Summary of results and outcomes of *Social resource-related interventions* (*n =* 196)

**Intervention Type** (number of studies)	**Intervention name** *Author, year* D=data type (study design); M=mode of delivery; I=level of interaction*; N=*number of participants; MM=mixed-/multi methods; NR=NR; QL=D: QL; QN=D: QN	**Outcomes** Outcomes are quantitative (QN) unless otherwise labelled as qualitative (QL) Effect direction definitions: “+*” = statistically significant positive impact; “-*” = statistically significant negative impact; “+” = positive direction in impact; “-” = negative direction in impact; “+/-” = mixed impact; NC = no change; NA = not applicable.		
		**Loneliness outcomes**	**Social outcomes**	**Health, wellbeing, and other outcomes**
**Information Communication Technology (ICT) based interventions** (***n=*41)**	**SOCIAL NETWORKING PLATFORMS AND APPS** **(*n =* 30)**			
	**A Personal Reminder Information and Social Management (PRISM) Technology-Based Application on a PC** *Czaja SJ, 2018 from Shah SG, 2021* D: QN (RCT); M: Virtual (Computer); I: NR*; N=*300	Loneliness (NC)	-	-
	**About My Age (Social Networking Site)** *Ballantyne A, 2010 from Heins P, 2021* D: QL; M: Virtual (Not specified) ; I: One-to-one*; N=*6	QL: Temporal loneliness (+)	QL: Connectivity (+)	-
	**Accessible iPad-Based Communication App** *Barbosa Neves B, 2019* D: MM; M: Virtual (Tablet) ; I: Self-directed *; N=*12	-	QL: Frequency of social interaction (+) QL: Social connection(+/-) QL: Perceived social interaction (+) QL: Maintenance of social interaction (+) QL: Meaningful relationships (NC)	Well-being (+/-) Resilience (+/-)
	**Ambient Social Network System “Tlatoque”** *Cornejo R, 2013 from Heins P, 2021* D: QL; M: Virtual (Not specified) ; I: NR*; N=*Older adults = 2; Family members = 30	-	QL: Social connectedness (+)	-
	**Caring TV** *Hemberg J, 2018 from Heins P, 2021* D: QL; M: Virtual (Not specified) ; I: NR*; N=*7	-	QL: New experiences (+) QL: Developing social relationships (+) QL: Maintaining social relationships (+)	-
	**Communication Application** *Shinokawa S, 2023* D: MM; M: Virtual (Smartphone); I: Mixed (Group-based, self-directed)*; N=*9	QL: Loneliness (+)	-	-
	**Content Creation Management System** *Morganti L, 2016 from Rivera-Torres S, 2021* D: QN (RCT); M: Virtual (Computer); I: NR*; N=*34	Social loneliness (+) Emotional loneliness (+) General loneliness (NC) Feeling of loneliness (NC)	-	-
	**Facebook** *Myhre JW, 2017 from Heins P, 2021* D: QN (Clinical controlled trial); M: Virtual (Not specified) ; I: NR*; N=*60	Loneliness (NC)	Social integration (NC) Social support (NC)	-
	**Facilitator-led Remote Interactive Intervention for Loneliness, Quality of Life, and Social Support** *Liu CW, 2023* D: QN (RCT); M: Virtual (Smartphone); I: Group-based*; N=*100	Loneliness (NC)	Social support (NC)	Quality of life (+*)
	**Fik@ room** *Johansson-Pajala RM, 2023* D: MM; M: Virtual (tablet); I: Group-based*; N=*28	QL: Loneliness (+)	QL: Develop new friendships (+) QL: Increase one's social network (+)	-
	**Health Enhancement Support System (CHESS)** *Leszko M, 2020 from Mao W, 2023* D: MM; M: Virtual (Computer); I: Group-based *; N=*48	Loneliness (+*)	-	Psychological frailty (NC) Total frailty (NC)
	**In Touch Social Contact System** *Judges RA, 2017 from Heins P, 2021* D: QL; M: Virtual (Not specified) ; I: NR*; N=*Older adults = 10; Volunteers = 10	-	QL: Improved or enhanced relationships (+) QL: Communication (+)	-
	**Internet Social Networking** *Ballantyne A, 2010 from Noone C, 2022* D: QL; M: Virtual (Computer); I: One-to-one*; N=*4	-	QL: Social connectivity (+)	QL: Confidence (+)
	**iPad-Based Communication App** *Barbosa Neves B, 2019 from Choi HK, 2021* D: QN (Cross-sectional); M: Virtual (iPad); I: Self-directed*; N=*12	Loneliness (NC)	Social support (NC)	-
	**Media Parcels Social Networking System** *Zaine I, 2019* D: QL ; M: Virtual (Computer); I: Group-based *; N=*NR	-	QL: Deepened relationships (+) QL: Communication (+)	-
	**Media Parcels** *Zaine I, 2019 from Heins P, 2021* D: MM; M: Virtual (Not specified) ; I: NR*; N=*Older adults = 2; Family members = 2; Friends = 2	-	QL: Amount of contact with others (+) QL: Feelings of closeness with others (+)	-
	**Project VITAL At Home** *Nguyen LT, 2022 from Mao W, 2023* D: QN (Cross-sectional); M: Virtual (Tablet); I: Group-based *; N=*124	Loneliness (NC)	-	-
	**Remote Sharing with Family Members** *Noguchi T, 2022* D: QN (Quasi-experimental); M: Virtual (Television); I: Self-directed*; N=*115	Loneliness (NC)	Frequency of talking time with families living together (+*) Frequency of talking time with families not living together (+*) Satisfaction for the relationship with families living together (+*) Frequency of talking with friends (NC)	-
	**Remotely Delievered Technology** *Chen AT, 2021 from DesChâtelets JR, 2023* D: QL; M: Virtual (Not specified) ; I: NR*; N=*NR	-	QL: Social connectedness (+)	-
	**Senior App Suite** *Goumopoulos C, 2017 from Heins P, 2021* D: QN (Cross-sectional); M: Virtual (Not specified) ; I: NR*; N=*22	Loneliness (+*)	-	-
	**Services To Communicate** *Zaine I, 2019 from Rivera-Torres S, 2021* D: MM; M: Virtual (Smartphone); I: Group-based*; N=*6	-	QL: Developing relationships (+) QL: Amount of social contact (+) QL: Feelings of closeness with others (+) QL: Deepened relationships (+)	Well-being (+)
	**Social Internet-Based Activity (SIBA)** *Larsson E, 2016 from Shah SG, 2021* D: QN (RCT); M: Virtual (Computer); I: NR*; N=*30	Loneliness (NC)	-	-
	**Social Networking at Home** *Goumopoulos C, 2017 from Rivera-Torres S, 2021* D: MM; M: Virtual (Computer, tablet); I: NR*; N=*20	Loneliness (+*)	-	-
	**Social Networking** *Jansen-Kosterink SM, 2020 from Rivera-Torres S, 2021* D: QN (Quasi-experimental); M: Virtual (Computer, smartphone, tablet); I: NR*; N=*41	Loneliness (NC)	-	Quality of life (+)
	**SONIA communication platform** *Biniok P, 2015 from Heins P, 2021* D: QL; M: Virtual (Not specified) ; I: NR*; N=*30	-	QL: Social participation (+)	-
	**The Personal Reminder Information and Social Management (PRISM) System** *Czaja SJ, 2018 from Choi HK, 2021* D: QN (RCT); M: Virtual (Smartphone); I: Self-directed*; N=*244	Loneliness (+*)	Social isolation (+*) Social support (+*)	Self-efficacy (NC)
	**The Personal Reminder Information and Social Management (PRISM) System** *Czaja SJ, 2018 from Heins P, 2021* D: QN (Clinical controlled trial); M: Virtual (Not specified) ; I: NR*; N=*300	Loneliness (+*)	Perceived social support (+*)	-
	**Using Communication Technology** *Myhre JW, 2017 from Casanova G, 2021* D: QN (RCT); M: Virtual (Not specified); I: NR*; N=*43	Loneliness (NC)	-	-
	**Virtual Classroom to Message** *Czaja SJ, 2018 from Rivera-Torres S, 2021* D: QN (RCT); M: Virtual (Computer); I: Group-based*; N=*224	Loneliness (+*)	Social isolation (+*) Social support (+)	-
	**Web-based Telehealth System with Facebook and Game Like Features** *Dhillon JS, 2011 from Chen YRR, 2016* D: QL; M: Virtual (Computer); I: Self-directed*; N=*NR	QL: Loneliness (+)	QL: Social interaction (+)	-
	**DEVICE-MEDIATED COMMUNICATION** **(*n =* 9)**			
	**Digital Information Technology-Based Intervention**s *Czaja SJ, 2018; Hind D, 2014; Jarvis MA, 2019; Larsson E, 2016; Morton TA, 2018; Myhre JW, 2017; Tsai HH, 2020; Tsai HH, 2010 from Lestari WA, 2023* D: QN (RCT, pre-post); M: Virtual (Computer, telephone); I: Mixed (Group-based, one-to-one)*; N=*NR	Loneliness (+/-)	-	-
	**iPad To Socialize** *Barbosa Neves B, 2019 from Rivera-Torres S, 2021* D: MM; M: Virtual (iPad); I: Group-based*; N=*12	Loneliness (NC)	Social interaction (+) Social connectedness (+/-) Social support (NC)	-
	**Messaging Chat** *Garattini C, 2012 from Rivera-Torres S, 2021* D: MM; M: Virtual (Computer, smartphone); I: NR*; N=*19	-	Amount of social connections (+) New social interactions (+)	-
	**Phone and Video Conferencing Service** *Airola E, 2020 from Heins P, 2021* D: QL; M: Virtual (Telephone); I: One-to-one*; N=*5	QL: Loneliness (+)	QL: Networks (+)	-
	**Smart Technology Interventions** *Dew MA, 2004; Hill W, 2006; Weinert C, 2011;* *Weinert C, 2008; Barrera M Jr, 2002; Billipp SH, 2001; Bond GE, 2010; Chiu T, 2009; Fokkema T, 2007; Gustafson DH, 2005; Kahlbaugh PE, 2011; Lieberman MA, 2005; Mahoney DF, 2003; Pierce LL, 2009; Samoocha D, 2011; Slegers K, 2008; Van Straten A, 2008; Torp S, 2008 from Morris ME, 2014* D: QN (RCT, cohort study); M: Virtual (Computer); I: Mixed (Group-based, self-directed, one-to-one)*; N=*NR	Loneliness (+/-)	Social support (+*)	Quality of life (+) Health-related quality of life (+)
	**Technology for Long-Distance Interactions** *Ibarra F, 2020 + from Ibarra F, 2020* D: MM; M: Virtual (Computer, smartphone, tablet); I: NR*; N=*NR	Loneliness (+/-) QL: Loneliness (+/-)	Social network size (+/-)	-
	**Various Digital Tools to Support Social Engagement** *Choi M, 2012; Chen YR, 2016; Morris ME, 2014; Forsman AK, 2017 from Larsson E, 2020* D: MM; M: Virtual (Not specified) ; I: NR*; N=*NR	Loneliness (+*)	Social isolation (+*) Feelings of belonging (+*) Social interaction (+) Social support (+)	-
	**Video Calling** *Noon C, 1996 from Astasio-Picado Á, 2022* D: QN (Systematic review); M: Virtual (Not Specified); I: NR*; N=*201	Loneliness (+)	-	Quality of life (+)
	**Video Chat** *Kleinberger R, 2019 from Rivera-Torres S, 2021* D: MM; M: Virtual (Smartphone); I: NR*; N=*10	-	QL: Connectedness (+)	-
	**COMBINATION OF SOCIAL NETWORKING AND DEVICE-MEDIATED COMMUNICATION** **(*n =* 2)**			
	**General ICT** *Mellor D, 2008 from Chen YRR, 2016* D: QL; M: Virtual (Smartphone, tablet); I: Self-directed*; N=*NR	-	QL: Social connectedness (+)	Well-being (NC) Everyday functioning (NC) QL: Life satisfaction (+)
	**Various Communication Technology Interventions** *Baker S, 2018; Brimelow RE, 2017; Casanova G, 2021; Cattan M, 2005; Chen E, 2022; Chen YR, 2016; Choi HK, 2021; Choi M, 2012; Cohen-Mansfield J, 2015; Dickens AP, 2011; Dickens AP, 2011; Franck L, 2016; Gardiner C, 2018; Gasteiger N, 2021; Gorenko JA, 2021; Hagan R, 2014; Heins P, 2021; Ibarra, 2020; Ibrahim AF, 2022; Isabet B, 2021; Khosravi P, 2016; Khosravi P, 2016; Li J, 2018; Masi CM, 2011; Morris ME, 2014; Cochrane Public Health Group, 1996; O’Rourke HM, 2018; Poscia A, 2018; Shah SG, 2021 from Döring N, 2022* D: MM; M: Virtual (Not specified) ; I: NR*; N=*>71000	Loneliness (+/-)	Social isolation (+/-)	-
**Intergenerational interventions** (***n=*33)**	**TO PROMOTE OR BUILD SOCIAL CONNECTIONS AND ENGAGEMENT (INTERGENERATIONAL CONNECTEDNESS AND EXCHANGE)** (***n =* 12)**			
	**Intergenerational Nursing Communication Project** *Kirk L, 2023* D: MM; M: NR; I: Mixed (Group-based, one-to-one)*; N=*124	-	Social connectedness (+)	-
	**Big and Mini: Intergenerational Program for Social Connection** *Xu L, 2022* D: MM; M: Virtual (Telephone); I: One-to-one*; N=*Bigs: 63 participants; Minis: 53 participants	QL: Loneliness (+)	QL: Building new relationships (+) QL: Intergenerational closeness (+)	-
	**Friendship and Engaging with College Students** *Sehrawat S, 2017 from Ibrahim AF, 2022* D: QL; M: In-person; I: One-to-one*; N=*4	-	QL: Social network size (+) QL: Social connectedness (+)	QL: Eudaimonic well-being (+)
	**Intergenerational Connections** *Peterat L, 2006 from Peters R, 2021* D: QL; M: NR; I: NR*; N=*7	-	QL: Cross generation connection (+)	-
	**Intergenerational Engagement Intervention** *Barbosa MR, 2021 from Krzeczkowska A, 2021* D: MM; M: NR; I: NR*; N=*12	Loneliness (+*)	-	-
	**Intergenerational Engagement Intervention** *Kamei T, 2011 from Krzeczkowska A, 2021* D: MM; M: NR; I: NR*; N=*22	-	QL: Expansion of social interactions (+)	-
	**Intergenerational Forum Program** *Lee OE, 2022* D: QN (Quasi-experimental); M: In-person; I: Group-based*; N=*104	-	Social isolation (NC) Social capital (NC)	Well-being (+*)
	**Intergenerational Friendly Telephone Visit Program** *Kumar AB, 2023* D: QL; M: Virtual (Telephone); I: One-to-one*; N=*10	-	QL: Interpersonal connection (+) QL: Intergenerational connection (+) QL: Social interaction (+) QL: Ease of conversation (+)	-
	**Intergenerational Mentor-Up** *Lee OE, 2019 from Krzeczkowska A, 2021* D: MM; M: NR; I: NR*; N=*55	Loneliness (+*)	Social isolation (+*) Social support (NC)	-
	**Social Inclusion Intervention** *Alcock CL, 2011 from Krzeczkowska A, 2021* D: QL; M: NR; I: NR*; N=*13	-	QL: Sense of community (+) QL: Companionship (+)	-
	**Socrates Cafes** *Dinkins CS, 2019* D: QL ; M: In-person; I: Group-based; =NR	-	QL: Built relationships (+) QL: Social connection (+)	-
	**YOLG Program** *Sun Q, 2019 from Krzeczkowska A, 2021* D: QN (Quasi-experimental); M: NR; I: NR*; N=*150	-	Initiating conversation with young participants (+)	Self-confidence (+*)
	**TO SHARE EXPERIENCES AND MEMORIES INCLUDING REMINISCENCE** **(*n =* 7)**			
	**Aging Is Very Personal Program (AIVP)** *Beausoleil K, 2022* D: MM; M: Virtual (Computer); I: One-to-one*; N=*51	-	QL: Social isolation (+) QL: Meaningful intergenerational relationships (+)	-
	**DOROT's Summer Teen Internship Program** *Parkinson D, 2019* D: QL ; M: In-person; I: One-to-one*; N=*48	-	QL: Social connectedness (+)	-
	**Intergenerational Engagement Intervention** *Mahoney N, 2020 from Krzeczkowska A, 2021* D: MM; M: NR; I: NR*; N=*15	-	Social functioning (NC)	-
	**Intergenerational Reminiscence Intervention** *Gaggioli A, 2014 from Hutchinson TD, 2022* D: MM; M: In-person; I: Group-based*; N=*32	QL: Loneliness (+)	-	QL: Quality of life (+)
	**Intergenerational Reminiscence Intervention** *Gaggioli A, 2014 from Krzeczkowska A, 2021* D: QN (Cross-sectional); M: NR; I: NR*; N=*32	-	Closeness to children (+*) Communal involvement (+*)	-
	**Intergenerational Reminiscence Therapy** Gaggioli A, 2014 from Poscia A, 2018 D: QN (Cross-sectional); M: In-person; I: Group-based*; N=*32	Emotional loneliness (+*) Loneliness (+) Social loneliness (+)	-	Quality of life (+*)
	**Promoting Interaction Between Older Adults and Children** *Barbosa MR, 2021 from Carvalho MI, 2022* D: QN; M: In-person; I: NR*; N=*NR	Loneliness (+*)	-	-
	**COMMUNITY-BASED INTERGENERATIONAL PROGRAMS OR SERVICES** **(*n =* 6)**			
	**Art-Technology Intergenerational Community (ATIC) Program** *Seo JH, 2021* D: MM; M: In-person; I: Group-based*; N=*18	-	QL: Social connectedness (+)	-
	**Good Neighbor Program** *Sandu S, 2021* D: QN (Observational); M: Virtual (Telephone); I: One-to-one*; N=*261	Loneliness (NC)	-	Resilience (+*)
	**Intergenerational Community-Based Program** *Young TL, 2013 from Krzeczkowska A, 2021* D: QN (Cross-sectional); M: NR; I: NR*; N=*197	-	Social life (+*)	-
	**Intergenerational Program: Arts-Based** *Cohen-Mansfield J, 2022* D: MM; M: In-person; I: Group-based*; N=*84	Loneliness (+*)	QL: Meeting other generations (+) QL: Meeting new friends (+)	-
	**Intergenerational Program: Assistance-Based** *Cohen-Mansfield J, 2022* D: MM; M: In-person; I: One-to-one*; N=*84	Loneliness (+*)	QL: Building new relationships (+)	Instrumental activities of daily living (NC)
	**Intergenerational Program: Learning-Based** *Cohen-Mansfield J, 2022* D: MM; M: In-person; I: Mixed (Group-based, one-to-one)*; N=*84	Loneliness (+*)	QL: Meeting other generations (+)	-
	**GENERAL INTERGENERATIONAL INTERVENTIONS** **(*n =* 4)**			
	**Assessing Health and Social Capital** *de Souza EM, 2007 from Krzeczkowska A, 2021* D: QN (RCT); M: NR; I: NR*; N=*266	-	Social functioning (+/-)	-
	**Health Professional Mentoring** *Halpin SN, 2017 from Krzeczkowska A, 2021* D: MM; M: NR; I: NR*; N=*147	-	Social functioning (+*) QL: Relationship meaningfulness (+)	-
	**Intergenerational Empowerment Intervention** *Gamliel T, 2014 from Krzeczkowska A, 2021* D: MM; M: NR; I: NR*; N=*29	Loneliness (+*)	-	-
	**Older Adult Tutors** *Carstensen L, 1982 from Krzeczkowska A, 2021* D: MM; M: NR; I: NR*; N=*23	-	In touch with one's community (+)	-
	**TO PROMOTE SOCIALIZATION THROUGH ACTIVITIES OR SKILLS TRAINING** (***n =* 4)**			
	**Intergenerational Mentor-Up (IMU)** *Lee OEK, 2019* D: MM; M: In-person; I: Group-based*; N=*55	Loneliness (+*)	Social isolation (+*) Perceived social support (+)	Health (+*) Functioning autonomy (+*)
	**Nintendo Wii with a Partner** *Kahlbaugh PE, 2011 from Hagan R, 2014* D: QN (RCT); M: In-person; I: One-to-one*; N=*35	Loneliness (+*)	-	-
	**Television intervention with a partner** *Kahlbaugh PE, 2011 from Hagan R, 2014* D: QN (RCT); M: In-person; I: One-to-one*; N=*35	Loneliness (-*)	-	-
	**Virtual Intergenerational Reverse-Mentoring Program Cyber-Seniors** *Juris JJ, 2022* D: QN (Quasi-experimental); M: Virtual (Computer); I: One-to-one*; N=*9	Loneliness (+)	-	-
**Aging in place** (***n =* 31)**	**HOME CARE - HOME VISIT INTERVENTIONS** **(*n =* 14)**			
	**‘Someone To Talk to’ Intervention** *Eliezer K, 2022* D: QL; M: Virtual (elephone); I: One-to-one*; N=*142	-	QL: Fostered meaningful relationships (+)	-
	**CARELINK Program** *Nicholson NR, 2013* D: QN (Quasi-experimental); M: In-person; I: One-to-one*; N=*56	-	Social isolation (+*)	-
	**ElderHelp Concierge Club (CC)** *Scharlach AE, 2015 from Tricco AC, 2022* D: QN (Quasi-experimental); M: In-person; I: NR*; N=*21	-	Social isolation (NC) Amount of contact with family and friends (NC)	-
	**Health Teams Advancing Patient Experience: STRengthening QualitY (Health TAPESTRY)** *Dolovich L, 2019 from Tricco AC, 2022* D: QN (RCT); M: In-person; I: One-to-one*; N=*312	-	Social network scores (NC) Social satisfaction (NC)	-
	**Home Visits** *Hall N, 1992; McEwan RT, 1990; Van Rossum E, 1993; Sørensen KH, 1988; Vetter NJ, 1984 from van Haastregt JC, 2000* D: QN (RCT); M: In-person; I: NR*; N=*NR	Loneliness (+/-)	-	-
	**Home-Based Health Services** *McEwan RT, 1990; Bartsch DA, 2009; Bartsch DA, 2013 from Paquet C, 2023* D: MM; M: NR; I: NR*; N=*NR	Loneliness (+)	Social isolation (+)	-
	**Peer Counseling and Social Engagement** *Carandang RR, 2020* D: QN (Quasi-experimental); M: In-person; I: Mixed (Group-based, one-to-one)*; N=*133	Loneliness (NC)	Perceived social support (+*)	-
	**Pet Support Program (PSP)** *Cryer S, 2021* D: QL; M: In-person; I: NR*; N=*14	-	QL: Social isolation (+) QL: Social connectedness (+)	Resilience (+*) Activities of daily living (NC) Self-efficacy (NC) Instrumental activities of daily living (NC) QL: Quality of life (+)
	**Psychological Support Service for Socially Isolated Elderly People (PSIE)** *Santos-Olmo AB, 2022* D: QN (Quasi-experimental); M: In-person; I: One-to-one*; N=*68	-	Unmet social needs (+*)	-
	**Safety and Care Services (SCS)** *Lim JW, 2023* D: QN (RCT); M: Mixed (In-person and virtual (telephone); I: Self-directed*; N=*40	Loneliness (NC)	-	-
	**Social Health Intervention** *Clarke M, 1992 from Ibrahim AF, 2022* D: QN (RCT); M: In-person; I: One-to-one*; N=*523	Perceived loneliness (NC)	-	-
	**The Community-Based Health Home (CBHH) model** *Sadarangani T, 2019* D: QN (RCT); M: In-person; I: Group-based*; N=*176	Loneliness (+*)	Meaningful peer relationships (+) QL: Social isolation (+) QL: Productive engagement (+)	Subjective well-being (+*) Quality of life (+*)
	**The Public Health Intervention "Fall Prevention"** *Ožić S, 2020* D: QN (Prospective controlled trial); M: In-person; I: Mixed (Group-based, one-to-one)*; N=*410	Loneliness (NC)	-	Social frailty (NC)
	**HOME CARE - TELEHEALTH INTERVENTIONS** **(*n =* 7)**			
	**Care TV Duplex Video/Voice Network** *Van Der Heide LA, 2012* D: QN (Quasi-experimental); M: Virtual (Alarm, video); I: One-to-one*; N=*130	Feelings of loneliness (+*)	-	-
	**SIBA (Social Internet-Based Intervention Activities)** *Larsson E, 2016* D: QN (RCT); M: Mixed (In-person and virtual (not specified)); I: Mixed (Group-based, one-to-one)*; N=*30	Loneliness (+*)	Social interaction skills (+/-)	-
	**Social Bridging Project** *Noble LW, 2022* D: MM; M: Virtual (telephone); I: One-to-one*; N=*13	QL: Loneliness (+)	QL: Social connectedness (+) QL: Ease in expressing emotions (+)	QL: Well-being (+) QL: Health (+)
	**Tele-Health Interventons Bond** *GE, 2010; Morrow-Howell N, 1998 from Paquet C, 2023* D: MM; M: NR; I: NR*; N=*NR	Loneliness (+/-)	Social isolation (+/-)	-
	**Telecare** *Bowes A 2013 from Heins P, 2021* D: QL; M: Virtual (not specified); I: One-to-one*; N=*Older adults = 76; Caregivers = 16	-	QL: Improved or enhanced relationships (+) QL: Social networks (-)	-
	**The Use of Medical Alert Device** *Morgenstern LB, 2015 from Johnstone G, 2021* D: QN (Block RCT); M: NR; I: Self-directed*; N=*265	-	Perceived isolation (NC) Social connectedness (NC)	-
	**Video-Telephone Nursing Care** *Arnaert A, 2007 from Khosravi P, 2016* D: QN (Cross-sectional); M: Virtual (Telephone, video); I: One-to-one*; N=*71	Loneliness (+)	-	-
	**HOME CARE INTERVENTIONS - MEAL DELIVERY INTERVENTIONS** **(*n =* 4)**			
	**Food Recovery-Meal Delivery Program** *Ross JM, 2022* D: MM; M: In-person; I: One-to-one*; N=*49	Loneliness (+*)	-	Self-efficacy (NC)
	**Home-delivered meals services - Dartmoor Community Kitchen** *O’Leary MF, 2020* D: QN (Quasi-experimental); M: In-person; I: One-to-one*; N=*19	Loneliness (NC)	Sense of belonging (NC) Social capital (NC)	Life satisfaction (NC)
	**Home-delivered meals services - Meals on Wheels** *Wright L, 2015* D: QN (Quasi-experimental); M: In-person; I: One-to-one*; N=*62	Loneliness (+*)	-	-
	**Home-delivered meals services – Meals on Wheels** *Thomas KS, 2016* D: QN (RCT); M: In-person; I: One-to-one*; N=*376	Loneliness (+*)	-	-
	**DAY CARE OR SENIORS CENTRES** **(*n=* 8)**			
	**Day Care Services** *Lunt C, 2021* D: QN (Quasi-experimental); M: In-person; I: Group-based*; N=*94	Loneliness (+/-)	-	-
	**Day Centres for Older People** *Orellana K, 2020* D: MM; M: In-person; I: One-to-one *; N=*23	QL: Loneliness (+)	QL: Companionship (+) QL: Activities (+) QL: Maintaining social connections (+)	Independent living (+*) Quality of life (+*)
	**Nursing Intervention Program** *Abdel-Aziz HR, 2022* D: QN (Quasi-experimental); M: In-person; I: Group-based*; N=*50	Loneliness (+*)	-	-
	**Urban Health Centres Europe (UHCE)** *Franse CB, 2018 from Tricco AC, 2022* D: QN (Quasi-experimental); M: In-person; I: NR*; N=*2325	Loneliness (+*)	-	-
	**Day Care Services** *Lunt C, 2021* D: QN (Quasi-experimental); M: In-person; I: Group-based *; N=*94	Loneliness (+/-)	-	-
	**Village Membership for Ageing in Place** *Graham C, 2018 from Johnstone G, 2021* D: QN (Cross-sectional); M: In-person; I: NR*; N=*222	-	Frequency of talking with friends (-*) Getting together socially (NC) Community belonging (NC)	Ageing in place (+)
	**Village Membership for Ageing in Place** *Graham CL, 2014 from Johnstone G, 2021* D: QN (Cross-sectional); M: In-person; I: NR*; N=*282	-	Social engagement (+*)	-
	**Village Membership for Ageing in Place** *Graham CL, 2017 from Johnstone G, 2021* D: QN (Cross-sectional); M: In-person; I: NR*; N=*1753	-	Social connectedness (+) Social support received (+)	Quality of life (+)
**Socially Assistive Robots and Computer Agents** (***n =* 29)**	**COMPUTER AGENTS** **(*n =* 14)**			
	**Always On Virtual Agent** Sidner CL, 2018 from Gasteiger N, 2030 D: MM; M: In-person; I: One-to-one*; N=*44	Loneliness (NC)	Social support (NC) QL: Companionship (+)	-
	**Assistant Technology - Alexa Echo** *Balasubramanian GV, 2021* D: MM; M: Virtual (Alexa-Echo); I: Self-directed*; N=*44	QL: Loneliness (+)	-	-
	**Conversational Agent** *Ring L, 2013 from Choi HK, 2021* D: MM; M: Virtual (Computer); I: Self-directed*; N=*16	Loneliness (+*)	-	Well-being (NC)
	**Computer Conversational Agent** *Ring L, 2013 from Khosravi P, 2016* D: QN (Quasi-experimental); M: Virtual (Computer); I: Self-directed*; N=*14	Perceived loneliness (+)	Companionship (+)	-
	**Embodied Conversational Agent (ECA) “FitTrack”** *Bickmore TW, 2005 from Heins P, 2021* D: QN (Clinical controlled trial); M: NR; I: NR*; N=*21	Loneliness (NC)	-	-
	**Embodied Conversational Agent (ECA) Motion Sensor** *Ring L, 2015 from Heins P, 2021* D: MM; M: NR; I: NR*; N=*14	Loneliness (+*)	-	-
	**Personal Voice Assistants (PVA)** *Jones VK, 2021* D: MM; M: Virtual (Amazon Echo); I: Mixed (Self-directed, one-to-one)*; N=*16	Loneliness (+*)	-	Self-rated health (+*) QL: Well-being (+)
	**Relational Agent** *Bickmore TW, 2005 from Choi HK, 2021* D: QN (Quasi-experimental); M: Virtual (Computer); I: NR*; N=*21	Loneliness (NC)	-	-
	**Tanya Conversational Agent** *Ring L, 2015 from Gasteiger N, 2022* D: MM; M: In-person; I: One-to-one*; N=*12	Loneliness (+*) QL: Loneliness (+)	-	-
	**Tanya Conversational Agent** *Vardoulakis LP, 2012 from Gasteiger N, 2021* D: MM; M: In-person; I: One-to-one*; N=*12	-	QL: Companionship (+)	-
	**Virtual Robot** *Sidner CL, 2018 from Rivera-Torres S, 2021* D: QN (Quasi-experimental); M: Virtual (Computer); I: NR*; N=*44	-	Relationship status (NC)	QL: Coping (+)
	**Care Coach Conversational** **animal/pet avatar** *Chi NC, 2017 from Gasteiger N, 2023* D: MM; M: In-person; I: One-to-one*; N=*10	-	QL: Companionship (+) QL: Social interaction (+)	-
	**Digital Pet Avatar** *Chi NC, 2017 from Heins P, 2021* D: QL; M: In-person; I: One-to-one*; N=*10	-	QL: Social interactions (+) QL: Companionship (+)	-
	**Virtual Companion** *Machesney L, 2014 from Rivera-Torres S, 2021* D: MM; M: Virtual (Tablet); I: NR*; N=*13	QL: Loneliness (+)	-	-
	**ROBOTS** **(*n =* 16)**			
	**Animatronic Pet Program** *Tkatch R, 2021* D: QN (Quasi-experimental); M: In-person; I: Self-directed*; N=*216	Loneliness (+*)	-	-
	**Digital Pet** *Chi NC, 2017* D: QL ; M: Virtual (Computer); I: Self-directed*; N=*10	-	QL: Companionship (+) QL: Social interaction (+)	-
	**Joy for All Companion Pets** *Hudson J, 2020 from Gasteiger N, 2026* D: MM; M: In-person; I: One-to-one*; N=*20	QL: Feelings of loneliness (+)	QL: New connections (+) QL: Companionship (+)	-
	**Parret Shaped Social Robot** *Lim J, 2023* D: QN (Quasi-experimental); M: In-person; I: Self-directed*; N=*64	Loneliness (+*)	-	Quality of life (+)
	**Robotic Pet** *Hudson J, 2020* D: MM; M: In-person; I: Self-directed*; N=*20	-	QL: Companionship (+)	-
	**Robotic Pet** *Pollak C, 2022* D: QN (RCT); M: In-person; I: Self-directed*; N=*220	-	-	Social frailty (NC)
	**Ed Robot** *Wang RH, 2017 from Gasteiger N, 2032* D: QL; M: In-person; I: One-to-one*; N=*10	-	QL: Companionship (+)	-
	**Hyodol Human-Robot** *Lee OE, 2023* D: QL; M: In-person; I: One-to-one*; N=*12	QL: Loneliness (+)	-	-
	**Max Homecare and Companion Robot** *Gross H, 2015 from Gasteiger N, 2025* D: MM; M: In-person; I: One-to-one*; N=*9	QL: Feelings of loneliness (+)	-	-
	**NAO Robot with Incorporated Shakespearean Text Fields** *N, 2021* D: QN (Quasi-experimental); M: In-person; I: One-to-one*; N=*15	Loneliness (+*)	-	-
	**Prototype Robot** *Zuckerman O, 2020 from Gasteiger N, 2033* D: MM; M: In-person; I: One-to-one*; N=*39	QL: Loneliness (+)	QL: New connections (+)	-
	**Socially Assistive Robot**, *JamesVR Van Assche M, 2023* D: QL; M: Virtual (Computer); I: Self-directed*; N=*4	QL: Loneliness (+)	QL: Social isolation (+) QL: Companionship (+)	-
	**Giraff and Paro** *Baisch S, 2017 from Isabet B, 2021* D: QN (Cross-sectional); M: NR; I: NR*; N=*29	-	Social contact (+)	-
	**Giraff** *Cesta A, 2016 from Isabet B, 2021* D: QL; M: NR; I: NR*; N=*2	QL: Loneliness (+)	-	QL: Well-being (+)
	**MTR Texai project** *Beer JM, 2011 from Isabet B, 2021* D: QL; M: NR; I: NR*; N=*12	-	QL: Social isolation (+)	-
	**MTR-VGO system** *Seelye AM, 2012 from Isabet B, 2021* D: QL; M: NR; I: NR*; N=*Healthy OAs (*n =* 8) and MCI OAs (*n =* 1)	-	QL: Social connectedness (+)	-
**Befriending interventions** (***n =* 27)**	**TECHNOLOGY-MEDIATED BEFRIENDING INTERVENTIONS** **(*n =* 14)**			
	**Befriending Interventions** *Jones RB, 2015 from Paquet C, 2023* D: MM; M: Virtual (Not Specified); I: NR*; N=*NR	Loneliness (+)	Social networks (+)	-
	**Call in Time Programme** *Cattan M, 2011 from Noone C, 2022* D: QL; M: Virtual (Telephone); I: One-to-one *; N=*40	-	QL: Relationship meaningfulness (+) QL: Social participation (+)	-
	**Call in Time Programme** *Cattan M, 2011 from Ibrahim AF, 2022* D: MM; M: Virtual (Telephone); I: One-to-one*; N=*40	QL: Loneliness (+)	QL: Amounts of ordinary conversation (+) QL: Sense of belonging (+) QL: Reengage with the community (+) QL: Social activity (+) QL: Social functioning (-)	-
	**Loneliness Helpline calls at the Friendship at Every Age Program** *Balta M, 2023* D: QN (Quasi-experimental); M: Virtual (Telephone); I: One-to-one*; N=*275	Loneliness (+*)	-	Well-being (+*)
	**Medical Student-Led Social Phone Calls** *Hoyumpa G, 2022* D: QN (Quasi-experimental); M: Virtual (telephone); I: One-to-one*; N=*6	Loneliness (+)	-	-
	**Mobile application “Gezelsch App”** *Jansen-Kosterink SM, 2020* D: QN (Observational); M: Virtual (Computer, smartphone, tablet); I: Self-directed*; N=*41	Loneliness (+)	-	Quality of life (+)
	**Nationwide Telephone befriending and Helpline** *Preston C, 2019 from Noone C, 2022* D: QL; M: Virtual (telephone); I: One-to-one*; N=*42	-	QL: Forming light-hearted friendship (+) QL: Forming intimate friendship (+)	-
	**NEST Collaborative’s Remote Social Intervention** *Nolan RW, 2022* D: QN (Cross-sectional); M: Virtual (telephone); I: One-to-one*; N=*31	-	Social isolation (+)	QL: Independence (+)
	**One-on-one Telephone Friendship** *Hind D, 2014 from Heins P, 2021* D: MM; M: Virtual (telephone); I: Mixed (Group-based, one-to-one)*; N=*157	Loneliness (NC)	-	-
	**Telephone Befriending** *Newall NEG, 2015* D: QL ; M: Virtual (Telephone); I: One-to-one*; N=*26	-	QL: Feeling of connection with the outside world (+) QL: Feeling part of a community (+)	-
	**Telephone Befriending** *Mountain GA, 2014 from Douglas NF, 2023* D: QN (RCT); M: Virtual (Telephone); I: Mixed (Group-based, one-to-one)*; N=*35	Loneliness (+/-)	-	-
	**Telephone Befriending Program** *Cattan M, 2011 from Douglas NF, 2023* D: QL; M: Virtual (Telephone); I: One-to-one*; N=*40	QL: Feelings of loneliness (+)	QL: Social isolation (+)	-
	**The “Call in Time” Telephone Befriending** *Cattan M, 2011* D: QL ; M: Virtual (Telephone); I: One-to-one*; N=*40	QL: Loneliness (+)	QL: Amount of social activity (+) QL: Sense of belonging (+) QL: Re-engagement with community (+)	-
	**Volunteer Telephone Befriending** *Mountain GA, 2014 from Todd E, 2022* D: QN (RCT); M: Virtual (Telephone); I: NR*; N=*NR	Loneliness (NC)	-	-
	**NON-TECHNOLOGY-MEDIATED BEFRIENDING INTERVENTIONS** **(*n =* 10)**			
	**Befriending Intervention** *Niemann AL, 2023* D: QN (Quasi-experimental); M: In-person; I: One-to-one*; N=*26	Loneliness (+)	Social isolation (+)	-
	**Befriending Service** *Wiles J, 2019 from Noone C, 2022* D: QL; M: In-person; I: One-to-one *; N=*106	QL: Loneliness (+)	-	-
	**Caregiver Visits +** *Niemann AL, 2023* D: QN (Quasi-experimental); M: In-person; I: One-to-one*; N=*23	Loneliness (+)	Social isolation (+)	-
	**Coffee's in the Morning for Patients** *Johnson L, 2020* D: MM; M: In-person; I: Group-based*; N=*25	-	QL: Feeling part of a community (+)	Activities of daily living (-)
	**Community Engaged Arts (CEA) Programme** *Moody E, 2012 from Poscia A, 2018* D: QL; M: In-person ; I: Group-based*; N=*20	-	QL: Number of community connections (+)	-
	**Friendly Visitors** *Mulligan MA, 1978 from Ibrahim AF, 2022* D: QN (Quasi-experimental); M: In-person; I: One-to-one*; N=*23	-	Social isolation (+*) Amount of social contacts (+*)	QL: Independence (+) QL: Confidence (+) QL: General health (NC)
	**Peer Intervention** *Kotwal AA, 2021* D: MM; M: In-person ; I: One-to-one*; N=*74	QL: Loneliness (+)	QL: Self-perceived barriers to socializing (+)	Resilience (+*)
	**Volunteer-based Befriending** *Bantry-White E, 2018* D: QL ; M: In-person; I: One-to-one*; N=*22	-	QL: Relationship authenticity (+) QL: Relationship value (+)	QL: Perceived well-being (+/-)
	**Volunteer-based Befriending** *Gardiner C, 2016* D: QL ; M: In-person; I: One-to-one*; N=*11	-	QL: Social isolation (+)	-
	**Volunteer-based Befriending** *Smith R, 2018* D: MM; M: In-person; I: One-to-one*; N=*19	Loneliness (NC)	Social support (+*) Social isolation (+) QL: Making new friendships (+) QL: Relationship value (+)	QL: Well-being (+)
	**COMBINATION OF TECHNOLOGY- AND NON-TECHNOLOGY-MEDIATED BEFRIENDING INTERVENTIONS** **(*n =* 2)**			
	Befriending interventions Lai DWL, 2020; MacIntyre I, 1999, & Mountain GA, 2014 from Chau CMS, 2023 D: QN (RCT); M: Mixed (In-person and virtual (telephone)); I: One-to-one*; N=*NR	Loneliness (NC)	Social support (NC)	-
	Befriending Programme Lester H, 2012 from Noone C, 2022 D: QL; M: Mixed (In-person and virtual (telephone)); I: One-to-one *; N=*25	-	QL: Social engagement (+)	-
**Peer support group interventions** (***n =* 23)**	**IN-PERSON PEER SUPPORT GROUP INTERVENTIONS** **(*n =* 16)**			
	**Art, Exercise and Discussion Based Psychosocial Intervention** *Routasalo PE, 2009* D: QN (RCT); M: In-person; I: Group-based*; N=*235	Loneliness (NC)	New friendships (+*) Social networks (NC)	-
	**Bereavement Crisis Intervention (BCI)** *Constantino RE, 1988 from Dickens AP, 2011* D: QN (RCT); M: In-person; I: Group-based*; N=*150	-	Socialisation (+)	-
	**Bereavement Support Group** *Stewart M, 2001 from* D: QN (Quasi-experimental); M: In-person; I: Group-based*; N=*23	Social loneliness (+) Emotional loneliness (+) QL: Loneliness (+)	Social isolation (+) QL: Skills in developing social relationships (+)	-
	**Conducive Communities** *Andersson L, 1984 from Paquet C, 2023* D: MM; M: In-person; I: NR*; N=*NR	-	Social isolation (+)	-
	**Discussion Support Groups** *Anderson L, 1985 from Jarvis MA, 2020* D: QN (RCT); M: In-person; I: Group-based*; N=*NR	Loneliness (+*)	-	Self-efficacy (+*)
	**Men’s Shed Program** *Milligan C, 2015 from Poscia A, 2018* D: QL; M: In-person; I: Group-based*; N=*62	-	QL: Social isolation (+)	-
	**Path: From Loneliness to Participation** *Coll-Planas L, 2021 from Noone C, 2022* D: QL; M: In-person; I: Group-based*; N=*41	-	QL: Sense of belonging (+) QL: Companionship (+) Social integration (+)	-
	**Path: From Loneliness to Participation** *Coll-Planas, 2021* D: QL; M: In-person; I: Group-based *; N=*38	QL: Loneliness (+)	QL: Number of social relationships (+) QL: Engagement in social activities (+)	-
	**Psychosocial Group Rehabilitation** *Savikko N, 2009* D: MM; M: In-person; I: Group-based*; N=*117	Loneliness (+)	QL: Sense of belonging (+) Opportunities to meet friends (+) Opportunities to try new things (+) QL: Participating in community (+) QL: Social activation (+)	-
	**Self-Management Peer Support Groups** *Kremers IP, 2006 from Ibrahim AF, 2022* D: QN (RCT); M: In-person; I: Group-based*; N=*142	Social loneliness (+*)	-	-
	**Small Neighborhood Group Meetings** *Anderson L, 1985 from Johnstone G, 2021* D: QN (RCT); M: In-person; I: Group-based*; N=*57	Loneliness (+*)	Amount of social contact (+*)	-
	**Social Identity Intervention** *Lai DW, 2020 from Hickin N, 2021* D: QN (RCT); M: In-person ; I: Mixed (Group-based, self-directed)*; N=*60	Loneliness (+*)	-	-
	**Study Circle** *Åberg P, 2016* D: QL ; M: In-person; I: Group-based*; N=*1499	-	QL: Fellowship (+) QL: Sense of belonging (+) QL: possibility for meeting people (+)	-
	**Talking About Emotional & Social Estrangement** *Anderson L, 1985 from Douglas NF, 2023* D: QN (Cross-sectional); M: In-person; I: Group-based*; N=*35	-	Social integration (+)	-
	**The 'Participatory Group-based Care Management** *Ristolainen H, 2020* D: QN (RCT); M: In-person; I: Group-based*; N=*392	Loneliness (+)	-	Quality of life (NC)
	**The Dual-Process Bereavement Group Intervention-Chinese (DPBGI-C)** *Chow AYM, 2019* D: QN (RCT); M: In-person; I: Group-based*; N=*125	Social loneliness (+*) Emotional loneliness (+*)	-	-
	**VIRTUAL PEER SUPPORT GROUP INTERVENTIONS** **(*n =* 6)**			
	**Caregiving Support** *Banbury A, 2019 from Mao W, 2023* D: MM; M: Virtual (Computer); I: Group-based*; N=*69	-	QL: New connections (+)	-
	**Group Online Social Meetings** *Banbury A, 2017 from Douglas NF, 2023* D: MM; M: Virtual (Not specified); I: Group-based *; N=*52	-	-	QL: Self-confidence (+)
	**Koffee Klatch Support Chat Room** *Hill W, 2006 from Khosravi P, 2016* D: QN (RCT); M: Virtual (Computer); I: Group-based*; N=*183	Loneliness (NC)	Social support (+*)	-
	**Online Messaging for Caregivers** *McKechnie V, 2014 from Mao W, 2023* D: MM; M: Virtual (Computer); I: Group-based*; N=*61	QL: Loneliness (+)	QL: Social isolation (+)	-
	**Support Group** *O'Connor MF, 2014 from Mao W, 2023* D: MM; M: Virtual (Computer); I: NR*; N=*7	Loneliness (+)	-	Activities of daily living (+/-)
	**Virtual Caregiver Support Group** *O’Connor MF, 2014 from Khosravi P, 2016* D: QN (Cross-sectional); M: Virtual (Computer); I: Group-based*; N=*7	Loneliness (+)	-	-
	**COMBINATION OF IN-PERSON AND VIRTUAL PEER SUPPORT GROUP INTERVENTIONS** **(*n =* 1)**			
	**Memory Café** *Masoud SS, 2021 from Mao W, 2023* D: QL; M: Mixed (In-person and virtual (computer)); I: Group-based *; N=*12	-	QL: Social connectedness (+)	-
**Mentorship interventions** (***n=*11)**	**IN-PERSON MENTORSHIP INTERVENTIONS** **(*n =* 8)**			
	**Cadwyn Môn Programme** *Roberts JR, 2020* D: MM; M: In-person; I: Mixed (Group-based, one-to-one)*; N=*120	Loneliness (+*)	Social isolation (+*) QL: Social life (+)	-
	**Community Mentoring** *Dickens AP, 2011* D: QN (Prospective controlled trial); M: In-person; I: One-to-one*; N=*200	-	Getting along with others (-*) Social participation (+) Social participation (+)	-
	**Friendly Visits** *Baumgarten M, 1988 from Ibrahim AF, 2022* D: QN (Quasi-experimental); M: In-person; I: One-to-one*; N=*168	-	Satisfaction with social support (+) New social ties built (NC)	-
	**Mentoring Program** *Dickens AP, 2011 from Douglas NF, 2023* D: QN (Controlled trial); M: In-person; I: NR*; N=*200	-	Social isolation (NC)	-
	**Peer to Peer Support (P2P)** *Schwei RJ, 2021* D: QN (Observational); M: In-person; I: NR*; N=*448	Loneliness (NC)	Social support (NC)	Global functioning (+*) Health and psychosocial functioning (+*)
	**Peer-Support Program** *Fuller SM, 2022* D: QL; M: In-person; I: One-to-one*; N=*21	-	QL: Develop new friendships (+)	-
	**The Tai Chi Mentorship Intervention** *Chan AW, 2017* D: QN (RCT); M: In-person; I: Group-based*; N=*46	Loneliness (+*)	New friendships (+) Social support (+)	-
	**The Upstream Healthy Living Centre Intervention** *Greaves CJ, 2006* D: MM; M: In-person; I: One-to-one*; N=*172	-	Social support (+*) QL: Amount of social activity (+)	-
	**COMBINATION OF IN-PERSON OR VIRTUAL MENTORSHIP INTERVENTIONS** (***n =* 2)**			
	**Modified CARELINK Programme** *Hernández-Ascanio J, 2023* D: QN (RCT); M: Mixed (In-person and virtual (telephone)); I: One-to-one*; N=*121	Loneliness (NC)	Social isolation (NC)	Quality of life (NC)
	**Peer-Based Intervention** *Lai DWL, 2020* D: QN (RCT); M: Mixed (In-person and virtual (telephone)); I: Pair-based*; N=*60	Loneliness (+*)	Barriers to social participation (+*)	Life satisfaction (+)
	**Caring Callers Program** *Lee K, 2021* D: MM; M: Virtual (Telephone); I: One-to-one*; N=*15	Loneliness (NC)	-	-
**General social support interventions** (***n=*1)**	**GENERAL SOCIAL SUPPORT INTERVENTIONS** **(*n =* 1)**			
	**Social Support Interventions** *Carandang RR, 2020; Chen, MF, 2022; Cohen GD, 2006; Galinha IC, 2022, Heller K, 1991; Hind D, 2014; Johnson JK, 2020; Lai DWL, 2020; Li S, 2022; Ristolainen H, 2020; Rook KS, 2003; Thomas KS, 2016; Yang SY, 2023; Slegers K, 2008; Fokkema T, 2007; Larsson E, 2016; Thomas BH, 2004 from Yu DS, 2023* D: QN (RCT, N-RCT); M: NR; I: NR*; N=*NR	Loneliness (+*)	-	-

**Table 3:** Summary of results and outcomes (including effect direction) of *Self-management-related interventions* (*n =* 157)

**Intervention Type** (number of studies)	**Intervention name** *Author, year* D=data type (study design); M=mode of delivery; I=level of interaction*; N=*number of participants; MM=mixed-/multi methods; NR=NR; QL=D: QL; QN=D: QN	**Outcomes** Outcomes are quantitative (QN) unless otherwise labelled as qualitative (QL) Effect direction definitions: “+*” = statistically significant positive impact; “-*” = statistically significant negative impact; “+” = positive direction in impact; “-” = negative direction in impact; “+/-” = mixed impact; NC = no change; NA = not applicable.		
		**Loneliness outcomes**	**Social outcomes**	**Health, wellbeing, and other outcomes**
**Self-management education interventions** **(*n =* 106)**	**SOCIAL HEALTH TRAINING INTERVENTIONS** **(*n =* 30)**			
	**Activity Group** *Harris JE, 1978 from Dickens AP, 2011* D: QN (RCT); M: In-person; I: Group-based*; N=*102	-	Number of social interactions (+)	-
	**Adaptation of the Friendship Enrichment Program** *Bouwman TE, 2017 from Douglas NF, 2023* D: QN (Cross-sectional); M: Virtual (Not specified) ; I: Self-directed*; N=*239	Loneliness (+/-)	-	-
	**Analyzing Relationships and Making New FriendsMartina CM, 2006 from Douglas NF, 2023** D: MM; M: In-person; I: Group-based*; N=*60	Feelings of loneliness (NC)	Number of friends (+) Quality of friendships (+)	-
	**Analyzing Relationships, Making New Friends, and Improving Relationships** *Martina CM, 2018 from Douglas NF, 2023* D: MM; M: In-person; I: Group-based*; N=*108	Loneliness (NC)	New friendships (+)	-
	**Analyzing Social Relationships** *Vassilev I, 2019 from Douglas NF, 2023* D: QL; M: In-person; I: One-to-one*; N=*15	-	QL: Deepened relationships (+)	-
	**Community Socialization Intervention** *Rodrigues-Romero R, 2021* D: QN (RCT); M: In-person; I: Group-based*; N=*55	Loneliness (+*)	Social support (+*)	-
	**Education and Social Facilitation Intervention for Loneliness** *Alaviani M, 2015* D: QN (Quasi-experimental); M: In-person; I: Group-based*; N=*120	Loneliness (+*)	-	Subjective well-being (+)
	**Education on Making Friends** *Stevens NA, 2001 from Johnstone G, 2021* D: MM; M: In-person; I: Group-based*; N=*40	Loneliness (+*)	New friendships (+) Quality of friendships (+/-)	QL: Health (+)
	**Factsheets and Manual to Address Loneliness** *Gracia N, 2010 from Noone C, 2022* D: QL; M: In-person ; I: Self-directed*; N=*58	-	QL: Social participation (+)	-
	**Friendship Enrichment Programme (FEP)** *Owen L, 2016* D: QN (Cost or economic analysis); M: In-person; I: Group-based*; N=*115	Loneliness (NC)	-	Life satisfaction (+*)
	**Friendship Enrichment Programme (FEP)** *Martina CMS, 2006* D: QN (Quasi-experimental); M: In-person; I: Group-based*; N=*115	Loneliness (NC)	Number of friendships (+*) Quality of friendships (+)	-
	**Friendship Enrichment Programme (FEP)** *Martina CMS, 2018* D: QN (Quasi-experimental); M: In-person; I: Group-based*; N=*108	Loneliness (+/-)	-	Subjective well-being (+*)
	**Friendship Service** *Martina CM, 2006 from Ibrahim AF, 2022* D: QN (Quasi-experimental); M: In-person; I: One-to-one*; N=*115	Loneliness (+*)	Making friends (+*)	-
	**Group Course to Build Social Network and Foster Current Relationships** *Stevens NA, 2001 from Douglas NF, 2023* D: QN (Cross-sectional); M: In-person; I: Group-based*; N=*40	Loneliness (+)	QL: New social connections (+)	-
	**Group Course to Build Social Network and Foster Current Relationships** *Stevens NL, 2006 from Douglas NF, 2023* D: QN (Cross-sectional); M: In-person; I: Group-based*; N=*Study 1: 52; Study 2: 60	Loneliness (+)	-	-
	**Group Course to Build Social Network and Foster Current Relationships** *Tilburg NS, 2000 from Douglas NF, 2023* D: MM; M: In-person; I: Group-based*; N=*32	Loneliness (NC)	-	-
	**Improving Community Networks** *Saito T, 2012 from Douglas NF, 2023* D: QN (RCT); M: In-person; I: Group-based*; N=*20	Loneliness (+)	-	-
	**Lifestyle Matters Intervention** *Mountain G, 2017* D: QN (RCT); M: In-person; I: Mixed (Group-based, one-to-one)*; N=*288	Social loneliness (+*) Emotional loneliness (+*)	-	-
	**Loneliness Self-Help Print** *Gracia N, 2010* D: QL ; M: In-person; I: Self-directed*; N=*58	-	QL: Social participation (+) QL: Organization of activities (+)	-
	**Multi-Component Intervention to Decrease Loneliness** *Honigh-de Vlaming R, 2013 from Douglas NF, 2023* D: QN (Quasi-experimental); M: NR; I: NR*; N=*440	Loneliness (NC)	Social support (NC)	Well-being (NC)
	**Social Activities Group Program** *Nomura K, 2021* D: MM; M: In-person; I: Group-based*; N=*20	-	-	Independence of life (+*)
	**Social Participation Nursing Intervention** *Gosline MB, 2003* D: QL ; M: In-person; I: One-to-one*; N=*2	-	QL: Amount of interaction with friends and family (+) QL: Anticipation of social events (+) QL: Participation in social activities (+) QL: Socialization (+)	QL: Life satisfaction (+)
	**The ‘School of Health for Older People’** *Lapena C, 2020 from Noone C, 2022* D: QL; M: In-person; I: Group-based*; N=*28	-	QL: Peer relationships (+)	-
	**The ‘School of Health for Older People’** *Lapena C, 2020* D: QL; M: In-person; I: Group-based*; N=*28	QL: Feelings of loneliness (+)	QL: Social isolation (+) QL: Contact with others (+) QL: Feelings of belonging (+)	-
	**The ‘School of Health for Older People’** *Lapena C, 2022* D: QN (Quasi-experimental); M: In-person; I: Group-based*; N=*135	-	Social support (+)	Quality of life (+)
	**The Generating Engagement in Network Support (GENIE) Tool** *Welch L, 2020* D: QN (RCT); M: In-person; I: Mixed *; N=*60	-	Social network size (+) Frequency of social interactions (+) Amount of online engagement (+) Amount of activities (+)	Quality of life (NC)
	**The I-SOCIAL Intervention** *Cohen-Mansfield J, 2018* D: QN (RCT); M: In-person; I: Mixed (Group-based, one-to-one)*; N=*74	Loneliness (+*)	-	Well-being (+*)
	**The Social Isolation Prevention Intervention Program** *Saito T, 2012* D: QN (RCT); M: In-person; I: Group-based; N = 60	Loneliness (+*)	Social support (+*) Familiarity with services in the community (+*) Social activity (+)	-
	**The VRCHIVE Project** *Appel L, 2022 from Li M, 2023* D: QL; M: Virtual (Not Specified); I: NR*; N=*5	-	QL: Social isolation (+) QL: Social connection (+)	-
	**Virtual Coaching** *Brandenburgh A, 2014 from Rivera-Torres S, 2021* D: QN (Cross-sectional); M: Virtual (Computer); I: NR*; N=*7	Loneliness (+)	-	-
	**GENERAL HEALTH TRAINING INTERVENTIONS** **(*n =* 23)**			
	**A General Well-being Training Intervention** *Bartholomaeus JD, 2019* D: QN (Controlled before and after); M: In-person; I: Group-based*; N=*58	-	Social isolation (+/-)	-
	**Chronic Disease Self-Management Education (CDSME) Programs** *Smith ML, 2023* D: QN (Quasi-experimental); M: In-person; I: Group-based*; N=*295	Loneliness (+*)	-	-
	**Education About Assistive Devices** *de Craen AJM, 2006 from Cohen-Mansfield J, 2015* D: QN (RCT); M: In-person; I: One-to-one*; N=*402	Loneliness (NC)	-	-
	**Geriatric Rehabilitation Program** *Ollonqvist K, 2008 from Ibrahim AF, 2022* D: QN (RCT); M: In-person; I: Group-based*; N=*708	Loneliness (+)	Number of friends (-)	-
	**Health Aides** *Anderson L, 1985 from Ibrahim AF, 2022* D: QN (Quasi-experimental); M: In-person; I: Group-based*; N=*108	Loneliness (NC)	Social contact (+*)	-
	**Health and Social Provision Interventions** *de Craen AJM, 2006; Granbom M, 2017; Melin AL, 1993; Morrow-Howell N, 1998; Taube E, 2018; Thomas KS, 2016 from Chau CMS, 2023* D: QN (RCT); M: Mixed (In-person and virtual (telephone); I: One-to-one*; N=*NR	Loneliness (NC)	Social engagement (+*)	-
	**Health Education and Activities** *Rodríguez‐Romero R, 2021 from Douglas NF, 2023* D: QN (RCT); M: In-person; I: Group-based*; N=*55	Loneliness (+)	Social support (NC)	-
	**Health Promotion Interventions** *de Craen AJM, 2006; Franse CB, 2018; Gustafsson S, 2017; Taube E, 2017; van Rossum E; 1993 from Yu DS, 2023* D: QN (RCT, N-RCT); M: NR; I: NR *; N=*NR	Loneliness (-*)	-	-
	**Healthy Ageing Intervention** *Honigh-de Vlaming R, 2013* D: QN (Quasi-experimental); M: In-person; I: Group-based*; N=*1804	Loneliness (NC)	Social support (NC)	QL: Psycho-social well-being (+)
	**I'd Rather Stay 19-Minute Documentary Video** *Ottoni CA, 2020* D: QL ; M: In-person; I: Group-based*; N=*48	-	QL: Connection (+)	-
	**Lifestyle Redesign Intervention** *Juang C, 2018* D: QN (RCT); M: In-person; I: Group-based*; N=*460	-	Meaningful activity frequency (+*) Participating in activities (+*) Socializing with friends and family (+*) Activity significance (+*) Social connections (NC)	Well-being (+*)
	**Mental Health-Informed Lifestyle Program** *McKeon G, 2022* D: QN (Quasi-experimental); M: Virtual (Computer); I: Group-based*; N=*11	Loneliness (+)	-	Quality of life (+)
	**Persian Diabetes Self-Management Education (PDSME)** *Saghaee A, 2020* D: QN (RCT); M: In-person; I: Group-based*; N=*34	Loneliness (NC)	-	Quality of life (+*)
	**Self-Care Reinforcement Program (SCRP)** *Park M, 2019* D: QN (Quasi-experimental); M: In-person; I: Group-based*; N=*64	-	Social network quality (+*)	-
	**Senior CAN Educational Intervention** *Collins CC, 2006 from Cohen-Mansfield J, 2015* D: QN (Cross-sectional); M: In-person; I: Group-based*; N=*339	Loneliness (+*)	-	-
	**Successful Aging** *Kocken PL, 2998 from Ibrahim AF, 2022* D: QN (Quasi-experimental); M: In-person; I: Group-based*; N=*320	-	Social participation (NC))	-
	**Thanks, Sorry, Love, and Farewell Board Game** *Chen M, 2022* D: QN (Quasi-experimental); M: In-person; I: Group-based*; N=*91	Loneliness (+*)	Interpersonal communication (+*)	-
	**The Resident Wellness Coaching Program** *Fullen MC, 2023* D: QN (Quasi-experimental); M: Mixed (In-person and virtual (computer, paper)); I: Mixed (Group-based, self-directed)*; N=*79	Loneliness (+*)	Relatedness (+*)	Quality of life (NC)
	**The Virtual Learning Program** *Botner E, 2018* D: QL ; M: Virtual (Computer, tablet); I: Group-based*; N=*116	-	QL: Social isolation (+) QL: Social connection (+)	Health (+)
	**Video-Conferencing Program for Skill Development** *Yavuz C, 2023* D: QN (Cross-sectional); M: Virtual (Computer, smartphone); I: Group-based*; N=*92	Loneliness (+*)	-	QL: Well-being (+) QL: Health (+) QL: Self-efficacy (+) QL: Resilience (+)
	**Walk 'n Talk for Your Life** *Hwang J, 2019 from Noone C, 2022* D: QL; M: In-person ; I: Group-based*; N=*16	-	QL: Sense of belonging (+)	-
	**WESIHAT 2.0©** *Vanoh D, 2019 from Heins P, 2021* D: QN (RCT); M: Virtual (Smartphone); I: NR*; N=*60	Loneliness (NC)	Social support (NC)	-
	**Workshops for Health Resources** *Lapena C, 2020 from Douglas NF, 2023* D: QL; M: In-person; I: Group-based*; N=*26	-	QL: Social isolation (+)	-
	**COMBINATION OF TECHNOLOGY DEVICE AND INTERNET TRAINING INTERVENTIONS** **(*n =* 21)**			
	**Computer and Internet Training Provided by Volunteers** *Jones RB, 2015 from Poscia A, 2018* D: QN (Cross-sectional); M: In-person; I: Mixed (Group-based, one-to-one)*; N=*144	Loneliness (+/-)	Social isolation (+*)	Quality of life (+*)
	**Computer and Internet Training with Loaned PC** *Slegers K, 2008 from Chen YRR, 2016* D: QN (RCT); M: In-person; I: Group-based*; N=*236	Loneliness (NC)	Social networks (NC)	-
	**Computer Education** *Blažun, 2012 from Douglas NF, 2023* D: QN (Cross-sectional); M: In-person; I: Group-based*; N=*45	Loneliness (+)	-	-
	**Computer Training Program** *Kim J, 2016 from Heins P, 2021* D: QL; M: NR; I: NR*; N=*11	-	QL: Social connectedness (+)	-
	**Computer Training Program** *Slegers K, 2008 from Heins P, 2021* D: QN (Clinical controlled trial); M: NR; I: NR*; N=*236	-	-	-
	**Computer/Internet Training Program** *Woodward AT, 2011 from Heins P, 2021* D: QN (Clinical controlled trial); M: NR; I: NR*; N=*83	Loneliness (NC)	Social support (+)	-
	**Esc@pe Program** *Fokkema T, 2007 from Cohen-Mansfield J, 2015* D: QN (Quasi-experimental); M: In-person; I: One-to-one*; N=*15	Loneliness (+*)	-	-
	**How to Use Internet and Computer** *Fokkema T, 2007 from Douglas NF, 2023* D: MM; M: Virtual (Computer, smartphone, tablet); I: One-to-one*; N=*12	Loneliness (+)	-	-
	**How to Use the Internet** *White H, 2002 from Ibrahim AF, 2022* D: QN (RCT); M: Virtual (Computer); I: One-to-one*; N=*100	Loneliness (NC)	-	-
	**Internet at Home - Esc@pe** *Fokkema T, 2007 from Johnstone G, 2021* D: MM; M: NR; I: NR*; N=*26	Overall loneliness (+*) Emotional loneliness (+*) Social loneliness (NC)	QL: Connection (+)	-
	**Internet Information Station** *Mullins LB, 2020 from Heins P, 2021* • D: MM; M: In-person; I: Group-based*; N=*262	Loneliness (+)	QL: Social connectedness (+)	-
	**Internet Information Station (ISS) With Computer and Internet Training** *Mullins LB, 2020* D: MM; M: In-person; I: Group-based*; N=*36	Loneliness (+)	Amount of interactions (+) QL: Social engagement (+)	-
	**PC Computer and Internet Training with Online Discussion Forum** *Torp S, 2007 from Chen YRR, 2016* D: MM; M: In-person; I: Group-based*; N=*NR	-	Amount of social contact (+*) Social support (+*) QL: Contact with grandchildren (+) QL: New supportive friends with similar experience (+)	-
	**Skill Development Interventions** *Czaja SJ, 2018; Fields J, 2019 from Chau CMS, 2023* D: QN (RCT); M: Mixed (In-person and virtual (telephone, tablet); I: NR*; N=*NR	Loneliness (+*)	Social support (+*)	-
	**Technology and Internet Training** *Bornemann R, 2014 from Chipps J, 2017* D: QN (Meta-analysis); M: NR; I: NR*; N=*NR	Loneliness (+)	-	-
	**Using Communication Technology** *Blažun H, 2012 from Casanova G, 2021* D: QN; M: Virtual (Computer); I: NR*; N=*58	Loneliness (+*)	-	-
	**Using Communication Technology** *Cotten SR, 2012 from Casanova G, 2021* D: QN (RCT); M: Virtual (Computer); I: NR*; N=*205	Loneliness (+)	-	-
	**Using Communication Technology** *Morton TA, 2018 from Casanova G, 2021* D: QN (RCT); M: Virtual (Computer); I: NR*; N=*97	Loneliness (NC)	-	-
	**Using Communication Technology** *Slegers K, 2008 from Casanova G, 2021* D: QN (RCT); M: Virtual (Computer); I: NR*; N=*236	Loneliness (NC)	-	-
	**Facebook training** *Myhre JW, 2017* D: QN (Quasi-experimental); M: Mixed (In-person and virtual (not specified)); I: Mixed (Group-based, self-directed)*; N=*41	Loneliness (NC)	New relationships (+) New social interactions (+) Maintaining friendships (+) Social integration (NC) Social support (NC) Access to social support (NC)	-
	**Internet social networking website (ISNW) 'About My Age'** *Ballantyne A, 2010* D: QL ; M: Virtual (Computer); I: Mixed (Self-directed, one-to-one)*; N=*4	QL: Loneliness (+) QL: Temporal loneliness (+)	QL: Social connectedness (+) QL: New ways of linking with people (+)	-
	**TECHNOLOGY DEVICE TRAINING INTERVENTIONS** **(*n =* 16)**			
	**AGE-ON Tablet Training Program** *Neil-Sztramko SE, 2020 from Heins P, 2021* D: QN (Observational); M: In-person; I: NR*; N=*32	Loneliness (NC)	Social isolation (NC)	-
	**AGE-ON Tablet Training Program** *Neil-Sztramko SE, 2020* D: QN (Quasi-experimental); M: In-person; I: Group-based*; N=*32	Loneliness (NC)	Social isolation (NC) Social support (NC)	Quality of life (NC)
	**CATCH-ON Connect** *Wang S, 2023* D: QN (Quasi-experimental); M: Virtual (Tablet); I: One-to-one*; N=*129	Loneliness (+*)	Social isolation (NC)	General health (NC)
	**Cyber-Seniors Program** *Breck BM, 2018 from Heins P, 2021* D: QL; M: NR; I: NR ; N = Older adults = 29; Mentors = 28	-	QL: New connections (+) QL: Intergenerational engagement (+)	-
	**Intergenerational Mentor-Up** *Lee OE, 2019 from Heins P, 2021* D: MM; M: NR; I: Mixed (Group-based, one-to-one)*; N=*59	Loneliness (+*)	Social isolation (+*) Social support (NC)	-
	**iPad Training** *Burmeister OK, 2016 from Heins P, 2021* D: QL; M: NR; I: Group-based*; N=*6	-	QL: Social connectedness (+)	QL: Life satisfaction (+)
	**iPad Training** *Burmeister OK, 2016* D: QL ; M: In-person; I: Group-based*; N=*6	-	QL: Amount of social activity (+)	QL: Confidence (+)
	**iPad Training Program** *Arthanat S, 2016 from Heins P, 2021* D: MM; M: NR; I: NR*; N=*13	-	Number of activities involving social connections (+)	-
	**iPad/iPhone Training Program** *Emas S, 2018 from Heins P, 2021* D: MM; M: NR; I: Group-based*; N=*25	-	QL: Skills and knowledge in communicating (+)	-
	**iPads and Technology Training** *Delello JA, 2017 from Chen YRR, 2016* D: QN (Observational); M: In-person; I: Group-based*; N=*NR	-	Social connection (+)	-
	**Personal Reminder Information and Social Management (PRISM) System** *Czaja SJ, 2018* D: QN (RCT); M: Virtual (Smartphone); I: Self-directed*; N=*200	Loneliness (+*)	Social isolation (+*) Perceived social support (+*)	-
	**Project Wire Up** *Ngiam NH, 2022* D: QN (Quasi-experimental); M: Mixed (In-person and virtual (smartphone)); I: One-to-one*; N=*138	Loneliness (NC)	Social connectedness (NC)	Quality of life (NC)
	**Tablet Training** *Kim S, 2022* D: QL; M: In-person ; I: One-to-one*; N=*10	-	QL: Social connectedness (+) QL: Engagement with others (+)	-
	**Tablet Training** *(Fields J, 2021)* • D: MM; M: In-person; I: One-to-one; N = 83	Loneliness (NC)	QL: Feelings of connection with the world (+) Perceived social support (+)	-
	**Tech Clubs** *(Cutler C, 2016 from Heins P, 2021)* • D: QL; M: In-person; I: Group-based; N = 29	-	QL: Social interaction (+)	-
	**The Café-Multimedia Psychosocial Intervention** *Damne S, 2017* D: MM; M: In-person; I: One-to-one*; N=*13	Loneliness (NC)	Perceived social support (NC) QL: Positive social contact (+) QL: Everyday activity facilitation (+) QL: Engagement (+) QL: Making real friendships (-)	-
	**CAREGIVER SUPPORT EDUCATION** **(*n =* 7)**			
	**Assisting Carers Using Telematic Interventions to Meet Older People's Needs (ACTION)** *Savolainen L, 2008* D: QL ; M: Virtual (Computer); I: Pair-based*; N=*8	-	QL: Social isolation (+) QL: New friendships (+)	QL: Quality of life (+)
	**Caregiver Social Support Intervention** *Christie HL, 2022 from Mao W, 2023* D: QN (Randomized waitlist control); M: Virtual (Computer); I: NR*; N=*96	Loneliness (NC)	Social support (NC)	Quality of life (NC)
	**Caregiving Education Intervention** *Cox EO, 2007 from Cohen-Mansfield J, 2015* D: QN (Quasi-experimental); M: In-person; I: Mixed (Self-directed, one-to-one)*; N=*177	Loneliness (+*)	-	-
	**Dementia-Comprehensive Health Enhancement Support System (D-CHESS)** *Gustafson Jr DH, 2019 from Mao W, 2023* D: QN (RCT); M: Virtual (Computer); I: NR*; N=*31	Loneliness (+)	-	-
	**Engage Coaching for Caregivers** *Van Orden KA, 2023 from Mao W, 2023* D: QN (Cross-sectional); M: Virtual (Computer); I: NR*; N=*30	Loneliness (+)	Social isolation (+)	-
	**Engage Coaching for Caregivers** *Van Orden KA, 2023* D: QN (Quasi-experimental); M: Virtual (Computer, telephone); I: One-to-one*; N=*30	Loneliness (+*)	Social isolation (+*)	Quality of life (+*)
	**Psychoeducational Caregiver Program** *Cristancho-Lacroix V, 2015 from Mao W, 2023* D: MM; M: Virtual (Computer); I: NR*; N=*49	-	Social isolation (NC)	-
	**PEER-BASED SM EDUCATION** **(*n =* 5)**			
	**Engaged4Life Program** *Matz-Costa C, 2018 from Heins P, 2021* D: QN (RCT); M: Virtual (Not specified); I: NR *; N=*30	-	Social interaction (NC)	-
	**Peer Counseling** *Carandang RR, 2020* D: QN (Quasi-experimental); M: In-person; I: One-to-one*; N=*133	Loneliness (+)	Perceived social support (+*)	-
	**Peer Education Intervention** *Oetzel JG, 2020)* D: MM; M: NR; I: One-to-one *; N=*180	Loneliness (+*)	QL: Social connectedness (+)	Well-being (+*) Life satisfaction (+*) Health related quality of life (+*)
	**Peer Educators** *Simpson ML, 2021* D: MM; M: In-person; I: Group-based*; N=*26	-	QL: Social connectedness (+)	Health related quality of life (+*)
	**Samsam Language Café** *Ten Bruggencate T, 2019-a* D: QL ; M: In-person; I: Group-based*; N=*7	-	QL: Opportunities to connect with others (+)	-
	**SM SKILLS TRAINING** **(*n =* 4)**			
	**eHealth Self-Management System** *Jung H, 2017 from Johnstone G, 2021* D: QN (Quasi-experimental); M: Virtual (Computer, telephone); I: Mixed (Self-directed, one-to-one)*; N=*64	-	Social support (+*)	-
	**ElderTree** *Gustafson Sr DH, 2022* D: QN (RCT); M: Virtual (Computer); I: Mixed (Self-directed, one-to-one)*; N=*390	-	Social support provided (NC) Social support received (NC)	-
	**Self-Management Well-being Intervention** *Kremers IP, 2006* D: QN (RCT); M: In-person; I: Group-based*; N=*142	Social loneliness (+*) Emotional loneliness (NC) Overall loneliness (NC)	Self-management ability (+*)	-
	**Teaching Self-Management Skills** *Kremers IP, 2006 from Douglas NF, 2023* D: MM; M: In-person; I: Group-based*; N=*63	Loneliness (NC)	-	-
**Psychological self-management interventions** **(*n =* 43)**	**BEHAVIOURAL ACTIVATION OR COGNITIVE BEHAVIOURAL THERAPY (CBT)** **(*n =* 22)**			
	**Acceptance and Commitment Therapy (ACT)** *Zarling A, 2023* D: QN (Quasi-experimental); M: Virtual (Not Specified); I: One-to-one*; N=*529	Loneliness (+*)	-	Resilience (+*)
	**Cognitive Behaviour Therapy (CBT)** *Alaviani M, 2015 from Hickin N, 2021* D: MM; M: In-person ; I: Group-based *; N=*150	Loneliness (+*)	QL: Perceived social self-efficacy (+)	-
	**CBT** *Choi NG, 2020 from Hickin N, 2021* • D: QN (RCT); M: Virtual (Computer); I: Self-directed; N = 89	Loneliness (+)	Social interactions (+) Social support (+)	-
	**CBT** *Cohen-Mansfield J, 2018 from Hickin N, 2021* D: QN (RCT); M: In-person ; I: Mixed (Group-based, self-directed); N = 89	Loneliness (+*)	-	-
	**CBT** *Jarvis MA, 2019 from Hickin N, 2021* D: QN (RCT); M: Virtual (Computer); I: Mixed (Group-based, self-directed); N = 32	Loneliness (+*)	-	-
	**CBT** *Jing L, 2018 from Hickin N, 2021* D: QN (RCT); M: Virtual (Computer, telephone); I: Self-directed*; N=*80	Loneliness (+*)	-	-
	**CBT** *Kremers IP, 2006 from Hickin N, 2021* D: QN (RCT); M: In-person ; I: Group-based*; N=*142	Loneliness (NC)	-	-
	**Cognitive Telephone Therapy Groups** *Evans RL, 1986 from Cummings SM, 2004* D: QN (RCT); M: Virtual (Telephone); I: Group-based*; N=*43	Loneliness (+*)	Involvement in outside social activities (+)	-
	**Cognitive-Behavioral Based Intervention** *Cohen-Mansfield J, 2018 from Douglas NF, 2023* D: QN (RCT); M: In-person; I: Mixed (Group-based, one-to-one)*; N=*39	Loneliness (+)	-	-
	**Group Cognitive Behavioural Therapy** *Smith R, 2021* D: QN (RCT); M: In-person; I: Group-based *; N=*62	Loneliness (+*)	-	-
	**Integrative LISTEN Intervention** *Theeke LA, 2016 from Hickin N, 221* D: QN (RCT); M: In-person ; I: Group-based*; N=*27	Loneliness (+)	-	-
	**Lighten UP!** *Friedman EM, 2017 from Ibrahim AF, 2022* D: QL; M: In-person; I: Group-based*; N=*103	-	-	QL: Life satisfaction (+)
	**Low-Intensity Cognitive Behavior Therapy** *Jarvis MA, 2019* D: QN (RCT); M: Virtual (Computer, smartphone, tablet); I: Group-based*; N=*32	Overall loneliness (+*) Social loneliness (+*) Emotional loneliness (+*) Maladaptive social cognitions for loneliness and emotional deprivation (+*)	-	-
	**MoodTech - Cognitive Behaviorally Informed Internet Intervention** *Tomasino KN, 2017* D: QN (Experimental pilot study); M: Virtual (Computer, smartphone, tablet); I: Mixed (Group-based, one-to-one)*; N=*47	-	Social isolation (NC) Social support (NC)	-
	**Behavioural Activation (BA)** *Gilbody S, 2021 from Li M, 2023* D: QN (RCT); M: Virtual (Telephone); I: NR *; N=*96	Loneliness (+*)	-	-
	**Behavioural Activation (BA) Interventions** *Alaviani M, 2015; Carandang RR, 2020; Choi NG, 2020; Cohen-Mansfield J, 2018; Gilbody S, 2021; Hernández-Ascanio J, 2023; Mountain G, 2017; from Yu DS, 2023* D: QN (RCT, N-RCT); M: NR; I: NR *; N=*NR	Loneliness (NC)	-	-
	**Behavioural Activation in Social Isolation (BASIL)** *Gilbody S, 2021* D: QN (RCT); M: Virtual (Computer, telephone); I: Mixed (Self-directed, one-to-one)*; N=*96	Loneliness (+*)	-	-
	**Behavioural Activation in Social Isolation (BASIL)** *Littlewood E, 2022* D: QN (RCT); M: Virtual (Computer, telephone); I: One-to-one*; N=*96	Loneliness (+)	-	-
	**Brief Behavioral Activation for Improving Social Connectedness** *Pepin R, 2021* D: QL; M: Virtual (Telephone); I: One-to-one*; N=*3	QL: Loneliness (+)	QL: Social isolation (+)	-
	**Lay-Coach-Facilitated, Videocall, Short-Term Behavioral Activation Intervention** *Choi NG, 2020* D: QN (RCT); M: Virtual (Computer); I: Self-directed*; N=*89	Loneliness (+*)	Social interactions (+*) Satisfaction with social support (+*)	-
	**Social Engage (S-ENG)** *Van Orden KA, 2021* D: QN (RCT); M: In-person; I: One-to-one*; N=*62	-	Belonging (NC)	Social-emotional quality of life (+*)
	**Tele-delivered Behavioral Activation (BA)** *Bruce ML, 2021* D: QN (RCT); M: Virtual (Computer, telephone); I: One-to-one*; N=*89	Loneliness (+*)	Social interaction (+*) Satisfaction with social support (+*)	-
	**COMBINATION OF DIFFERENT PSYCHOLOGICAL INTERVENTIONS** **(*n =* 11)**			
	**Digital Group Intervention** *Shapira S, 2021* D: QN (RCT); M: Virtual (Computer); I: Group-based*; N=*86	Loneliness (+)	Social support (+)	QL: Well-being (+)
	**Holistic Therapy** *Parlak MM, 2023* D: QN (Quasi-experimental); M: In-person; I: Group-based*; N=*20	-	Social participation (-*) Social isolation (NC)	Quality of life (+*)
	**Mindfulness Intervention** *Creswell JD, 2012 from Hickin N, 2021* D: QN (RCT); M: In-person ; I: Mixed (Group-based, self-directed)*; N=*40	Loneliness (+*)	-	-
	**Mindfulness, Reflections, and Psychology Concepts** *Hudson J, 2023* D: QL; M: Virtual (Not Specified); I: One-to-one*; N=*11	-	QL: Improved relationships (+) QL: Social skills (+) QL: Confidence to initiate and maintain social contacts (+)	-
	**Psychological Interventions** *Keisari, 2022; Shapira, 2021; Sayied, 2015; Borji, 2020; Ojha, 2016; Pandya, 2021 from Yu DS, 2023* D: QN (RCT, N-RCT); M: NR; I: NR*; N=*NR	Loneliness (+*)	-	-
	**Psychological Therapies** *Choi NG, Marti CN, 2020; Choi NG, Pepin R, 2020; Gilbody S, 2021 from Chau CMS, 2023* D: QN (RCT); M: Virtual (Computer, telephone); I: NR*; N=*NR	Loneliness (NC)	Social engagement (+*)	-
	**Remotely Delivered Technology** *Conroy KM, 2020 from DesChâtelets JR, 2023* D: QL; M: NR ; I: NR *; N=*NR	QL: Loneliness (+)	QL: Maintaining social connections (+)	-
	**Resilience- and Wisdom-Focused Intervention** *Jeste DV, 2022* D: QN (Quasi-experimental); M: Virtual (Computer); I: One-to-one*; N=*20	Perceived loneliness (+)	-	-
	**Resilience- and Wisdom-Focused Intervention** *Jeste DV, 2023 from Li M, 2023* D: QN (Cross-sectional); M: Virtual (Not Specified); I: NR*; N=*20	Loneliness (+)	-	-
	**Teaching and Practicing Cognitive Behavioural and Mindfulness Skills** *Shapira S, 2021 from Li M, 2023* D: QN (RCT); M: Virtual (Not Specified); I: Group-based*; N=*82	Loneliness (+)	-	-
	**Therapy-Focused Interventions** *Creswell JD, 2012; Drentea P, 2006; Liu SJ, 2007 from Paquet C, 2023* D: MM; M: NR; I: NR*; N=*NR	Loneliness (+) QL: Loneliness (+)	-	-
	**PSYCHOSOCIAL INTERVENTIONS** **(*n =* 5)**			
	**Empowering Participants to Achieve Goals** *Routasalo PE, 2009 from Douglas NF, 2023* D: QN (RCT); M: In-person; I: Group-based*; N=*117	Loneliness (NC)	Social networks (+) New friendships (+)	Well-being (+)
	**Mood Lifters for Seniors Program** *Roberts JS, 2022)* D: QN (Quasi-experimental); M: Virtual (Computer); I: Group-based*; N=*24	Loneliness (NC)	-	QL: Independence (+) QL: Confidence (+)
	**Psychosocial Intervention Programme Using Volunteers** *Lorente-Martínez R, 2022* D: MM; M: In-person; I: One-to-one*; N=*48	Loneliness (NC) QL: Loneliness (+)	Subjective social participation (NC) QL: Subjective social participation (+)	-
	**Telephone Crisis Program** *Morrow-Howell N, 1998 from Cohen-Mansfield J, 2015* D: QN (Quasi-experimental); M: Virtual (Telephone); I: One-to-one*; N=*61	Loneliness (NC)	Amount of social contact (+*) Interpersonal contact (+*) Amount of telephone contact (NC)	-
	**The Happiness Route** *Weiss LA, 2020 from Douglas NF, 2023* D: QN (RCT); M: In-person; I: One-to-one*; N=*58	Loneliness (NC)	Social participation (NC)	-
	**REMINISCENCE THERAPY** **(*n =* 5)**			
	**Group Reminiscence Therapy Based on Chinese Traditional Festival Activities (CTFA-GRT)** *Li S, 2022* D: QN (RCT); M: In-person; I: Group-based*; N=*64	Loneliness (+*)	-	-
	**Memory Matters App** *Yu F, 2019 from Heins P, 2021* D: QN (RCT); M: In-person; I: Mixed (Group-based, self-directed)*; N=*80	-	Amount of social interactions (+*)	Social well-being (NC)
	**Reminiscence Therapy (RT)** *Diwan S, 2023* D: MM; M: In-person; I: Mixed (Group-based, one-to-one)*; N=*190	Loneliness (+*)	Relationship with family (+) Relationship with friends (+) QL: Connection with community (+)	Life satisfaction (+*) Well-being (NC)
	**Reminiscence Therapy in Combination with** **Physical Exercise** *Ren Y, 2021* D: QN (Quasi-experimental); M: In-person; I: Group-based*; N=*130	Loneliness (+*)	-	Resilience (NC)
	**Reminiscence Through Pictures** *Coll-Planas L, 2017 from Ibrahim AF, 2022* D: QN (Quasi-experimental); M: In-person; I: Group-based*; N=*38	Loneliness (+*)	Social participation (+*)	-
**Social prescribing or asset-based interventions** **(*n =* 8)**	**Connecting Points – Connecting People Project** *Bartlett H, 2013* D: QN (Quasi-experimental); M: In-person; I: One-to-one*; N=*15	Loneliness (NC)	Social support (NC)	-
	**Culturally Appropriate Volunteer Services (CAVS)** *Bartlett H, 2013* D: QN (Quasi-experimental); M: In-person; I: One-to-one*; N=*13	Loneliness (+*)	Social support (+*)	-
	**Health and Social Care Interventions** *Findlay RA; 2003, Gardiner C, 2018; Bickerdike L, 2017 & Poscia A, 2018 from Freedman A, 2020* D: QN (RCT); M: NR; I: NR*; N=*NR	-	Social isolation (+)	-
	**Link Worker Social Prescribing Programme** *Moffatt S, 2017* D: QL ; M: Mixed (In-person and virtual (computer, smartphone, tablet)); I: One-to-one*; N=*30	-	QL: Social isolation (+)	-
	**Program to Encourage Active, Rewarding Lives (PEARLS)** *Steinman L, 2021* D: QN (Quasi-experimental); M: In-person; I: One-to-one*; N=*320	Loneliness (+*)	Perceived isolation (+*) Social interaction (+*) Satisfaction with social support (+*)	-
	**Reconnections Program** *McDaid D, 2023* D: QL; M: In-person; I: Mixed (Group-based, one-to-one)*; N=*41	-	QL: New connections (+)	-
	**Social Prescribing** *Kim JE, 2021 from Li M, 2023* D: QN (Cross-sectional); M: In-person ; I: NR*; N=*10	Loneliness (+*)	Social participation (+*)	-
	**Social Prescribing** *Kim JE, 2021* D: QN (Quasi-experimental); M: Mixed (In-person and virtual (telephone)); I: Group-based*; N=*10	Loneliness (+*)	Social participation (+*)	-

**Table 4:** Summary of results and outcomes (including effect direction) of *Social behavioural activity interventions* (*n =* 140)^*^

**Intervention Type** (No. of studies)	**Intervention name** *Author, year* D=data type (study design); M=mode of delivery; I=level of interaction*; N=*No. of participants; MM=mixed-/multi methods; NR=NR; QL=D: QL; QN=D: QN	**Outcomes** Outcomes are quantitative (QN) unless otherwise labelled as qualitative (QL) Effect direction definitions: “+*” = statistically significant positive impact; “-*” = statistically significant negative impact; “+” = positive direction in impact; “-” = negative direction in impact; “+/-” = mixed impact; NC = no change; NA = not applicable.		
		**Loneliness outcomes**	**Social outcomes**	**Health, wellbeing, and other outcomes**
**Arts-based interventions** **(*n=*27)**	**DIRECT ENGAGEMENT ARTS-BASED INTERVENTIONS** **(*n =* 21)**			
	**Acting and Improvisation Course** *Sutherland L, 2023* D: MM; M: In-person; I: Group-based*; N=*14	-	Social isolation (NC) Community belonging (NC)	-
	**Activity-Based Musical Engagement Using iPads** *Engelbrecht R, 2015 from Heins P, 2021* D: MM; M: NR; I: NR*; N=*6	-	Social isolation (NC) QL: Social cohesion (+)	-
	**Arts-Based Intervention** *Watson B, 2023* D: QN (RCT); M: In-person; I: Group-based*; N=*252	Loneliness (+*)	Social quality (+*) Social activity (NC) Social functioning (NC)	-
	**Bibliotherapy and Therapeutic Creative Writing Group** *Malyn BO, 2020 from Noone C, 2022* D: QL; M: In-person; I: Group-based*; N=*12	-	QL: Social connection (+) QL: Relationships to self and others (+)	-
	**Chorale Singing Program** *Cohen GD, 2007 ****from*** *Ibrahim AF, 2022* D: QL; M: In-person; I: Group-based*; N=*128	Loneliness (NC)	Social activity levels (+*)	-
	**Community Dance Program (CDP)** *Wu VX, 2023* D: QL; M: Mixed (In-person and virtual (computer); I: Group-based*; N=*20	-	QL: Cultivation of intergenerational bonds (+) QL: Fostered meaningful relationships (+)	QL: Social well-being (+)
	**Community of Voices (COV)** *Johnson JK, 2020 from Ibrahim AF, 2022* D: QN (RCT); M: In-person; I: Group-based*; N=*390	Loneliness (+*)	-	-
	**Community of Voices (COV)** *Salazar M, 2020* D: QL; M: In-person; I: Group-based *; N=*31	QL: Loneliness (+)	QL: Social connectedness (+)	QL: Health (+)
	**Dance Interventions** *Shanahan J, 2016; Skingley A, 2016; Brustio PR, 2018; Pacheco E, 2016; Gouvêa JA, 2017; Douka S, 2019; Clifford AM, 2019; Westheimer O, 2015; McNeely ME, 2015; Shanahan J, 2017; Merom D, 2016; O’Toole L, 2015; Marquez DX, 2015; Merom D, 2016 ****from*** *McQuade L, 2023* D: MM; M: Mixed (In-person and virtual (video)); I: Mixed (Group-based, one-to-one)*; N=*NR	-	Social engagement (+/-)	Subjective well-being (+*) QL: Well-being (+) Quality of life (+/-)
	**Digital Storytelling (DST)** *Freeman S, 2020; Hausknecht S, 2019; Brandão, L, 2021; Hausknecht S, 2017; Stenhouse R, 2013; McGovern J, 2019; Loe M, 2013; Ward A, 2020; Bentley F, 2011; Karlsson E, 2014; Schoales C, 2020; Sljivic H, 2022; Sweeney L, 2021 ****from*** *Chang H, 2023* D: MM; M: NR; I: NR*; N=*NR	-	QL: Social connectedness (+) QL: Sense of belonging (+) QL: Improved or enhanced relationships (+)	Sense of social well-being (+)
	**Improvisation Comedy: Humor Doesn't Retire (HDR) Program** *Morse LA, 2018* D: QL ; M: In-person; I: Group-based*; N=*10	-	QL: Sense of community (+) QL: Social interaction behaviours (+)	-
	**Music and Singing Interventions** *Fu MC, 2018; Seinfeld S, 2013; Yap AF, 2017; Johnson JK, 2020; Davidson JW, 2014; Hallam S, 2014; Hallam S, 2016; Johnson JK, 2013 ****from*** *McQuade L, 2023* D: MM; M: In-person; I: Group-based*; N=*NR	Loneliness (+/-)	Social network scores (NC) QL: Social networks (+) QL: New connections (+) QL: Sense of belonging (+)	Quality of life (+/-)
	**Playful Living Program** *Brandão L, 2022* D: MM; M: Virtual (Computer); I: Mixed (Group-based, one-to-one)*; N=*34	-	QL: Social connectedness (+) QL: Sense of belonging (+)	-
	**Professionally Conducted Chorale** *(Cohen GD, 2006 ****from*** *Cohen-Mansfield J, 2015)* D: QN (N-RCT); M: In-person; I: Group-based*; N=*166	Loneliness (+)	-	-
	**Robot To Socialize** *Fields N, 2019 from Rivera-Torres S, 2021* D: QN (Pre-post); M: In-person; I: NR *; N=*15	Loneliness (+*)	-	-
	**Singing in Chorale** *Cohen GD, 2006 from Tricco AC, 2022* D: QN (N-RCT); M: In-person; I: Group-based*; N=*166	Loneliness (+)	-	-
	**Singing Sessions** *Teater B, 2014 from Ibrahim AF, 2022* D: MM; M: In-person; I: Group-based; N = 120	-	Amount of social contact (+) QL: Social isolation (+) QL: Social connectedness (+)	QL: Well-being (+)
	**The CALL-ME Community Arts Project** *Murray M, 2010* D: QL ; M: In-person; I: Group-based*; N=*11	-	QL: Opportunities for social interaction (+) QL: Opportunities for forming new friendships (+)	-
	**The Community of Voices (COV) Choir Program** *Johnson JK, 2020* D: QN (RCT); M: In-person; I: Group-based*; N=*390	Subjective feelings of loneliness (+*) Loneliness (+*)	-	Perceived control (NC)
	**The Golden Oldies Community-Engaged Arts (CEA) Singing Session Program** *Teater B, 2014 from Poscia A, 2018* D: MM; M: In-person; I: Group-based*; N=*120	-	QL: Social isolation (+) QL: Amount of social contact (+) QL: No. of community connections (+) Sense of community (+)	-
	**Traditional Music Engagement** *Engelbrecht R, 2015 from Heins P, 2021* D: MM; M: NR; I: NR *; N=*6	-	Social isolation (NC) QL: Social cohesion (+)	-
	**Montreal Museum of Fine Arts (MMFA)** *Beauchet O, 2022* D: QN (RCT); M: Virtual (Computer); I: Group-based*; N=*106	-	Social isolation (+*)	QL: Social health (+) QL: Capacity for activities of daily living (+) Quality of life (+*) Physical frailty (+*)
	**RECEPTIVE ENGAGEMENT ARTS-BASED INTERVENTIONS** **(*n =* 4)**			
	**The Bealtaine Art Festival Program** *O’Shea E, 2012* D: QL ; M: In-person; I: Group-based*; N=*253	QL: Loneliness (+)	QL: Social networking (+) QL: Social cohesion (+) QL: Connection with others (+)	QL: Quality of life (+)
	**Visual Art Discussions** *Wikström BM, 2002 from Cohen-Mansfield J, 2015* D: QN (RCT); M: In-person; I: Group-based*; N=*40	Loneliness (+*)	-	Levels of goal attainment (+)
	**Visual Arts Discussion** *Wikström BM, 2002 from Douglas NF, 2023* D: QN (Controlled trial); M: In-person; I: One-to-one*; N=*20	-	Social interaction (+*)	-
	**COMBINATION OF DIRECT AND RECEPTIVE ENGAGEMENT** **(*n =* 2)**			
	**Museum Social Prescribing Program** *Todd C, 2017* D: QL ; M: In-person; I: Group-based*; N=*20	QL: Loneliness (+)	QL: Built relationships (+) QL: Developed meaningful connections (+) QL: Novel social experiences (+) QL: Intense social experiences (+) QL: Opportunities to connect with others (+)	-
	**Music Engagement Program for Men** *Lindblad K, 2020* D: QL ; M: In-person; I: Mixed (Group-based, self-directed)*; N=*15	-	QL: Develop new friendships (+) QL: Maintaining friendships (+)	-
**Leisure Activity Interventions** **(*n =* 48)**	**GROUP-BASED LEISURE ACTIVITY INTERVENTIONS (N =19)**			
	**Come Eat Together (CET) Program** *Wildman JM, 2019* D: QL; M: In-person; I: Group-based*; N=*8	-	QL: Met new friends and people (+) QL: Social network size (+)	Well-being (+*)
	**Community Connection Program** *Siette J, 2021* D: MM; M: In-person; I: Group-based; N = 56	-	QL: Socialization (+) QL: Maintaining social connections (+)	QL: Confidence (+) QL: Quality of life (+)
	**Connect 60+ Wellness Program** *Weselman T, 2022* D: QL; M: Mixed (In-person and virtual (computer); I: Group-based*; N=*13	-	QL: Motivation to make social connections (+) QL: Motivation to seek out new activities (+) QL: Confidence to build social relationships (+)	-
	**Connection Through Calls: Seniors' Centre Without Walls (SCWW)** *Roland H, 2021* D: QN (Cross-sectional); M: Virtual (Telephone); I: Group-based*; N=*160	Loneliness (+*)	-	-
	**Engage with Age Program** *Montoro-Rodriguez J, 2022* D: QN (Pre-post); M: In-person; I: Group-based*; N=*86	Loneliness (+*)	Social isolation (+*)	-
	**Extra Time Hub (ETH)** *Jackman PC, 2023* D: QL; M: Mixed (In-person and virtual (computer); I: Group-based*; N=*10	QL: Loneliness (+)	QL: Social isolation (+) QL: Build new friendship networks (+) QL: Social connectedness (+) QL: Opportunities to meet people (+) QL: Engage in conversation (+) QL: Confidence in social situations (+)	Resilience (+)
	**Healthy Aging Web-Based Activity Program** *Cohen-Mansfield J, 2021* D: MM; M: Virtual (Computer); I: Group-based*; N=*105	Loneliness (+/-)	Social contact (+/-)	-
	**Horticulture Therapy** *Ng K, 2018* D: QN (RCT); M: In-person; I: One-to-one*; N=*59	-	Social connectedness (+*) Positive relationships (+*)	-
	**Men's Sheds Programme** *Nurmi MA, 2018 ****from*** *Noone C, 2022* D: QL; M: In-person; I: Group-based*; N=*64	-	QL: Opportunities for social engagement (+)	-
	**Men's Sheds Programme** *Reynolds KA, 2015 ****from*** *Noone C, 2022* D: QL; M: In-person; I: Group-based*; N=*12	-	QL: Enhanced friendships (+) QL: Social engagement (+)	-
	**Psychosocial Rehab** *Routasalo PE, 2009 ****from*** *Ibrahim AF, 2022* D: QN (RCT); M: In-person; I: Group-based*; N=*235	Loneliness (NC)	New friendships (+*) Social networks (NC)	Overall health (+*)
	**Seniors’ Satellite Program** *Hand C, 2022* D: MM; M: In-person; I: Group-based*; N=*28	-	Size of friend network (+) Quality of friend network (+) QL: Social isolation (+)	-
	**Social Activities Group Program** *Nomura K, 2021* D: MM; M: In-person; I: Group-based*; N=*20	-	-	QL: Subjective health (+)
	**Social Engagement** *Carandang RR, 2020* D: QN (N-RCT); M: In-person; I: Group-based*; N=*134	Loneliness (+*)	Perceived social support (+*)	Well-being (+*)
	**Social Farming Program** *Gagliardi C, 2019 ****from*** *Douglas NF, 2023* D: QN (Cross-sectional); M: In-person; I: Group-based*; N=*73	-	Amount of social contacts (+*)	-
	**The Garden Project** *Middling S, 2011 ****from*** *Ibrahim AF, 2022* D: QN; M: In-person; I: Group-based*; N=*200	-	QL: Socialization (+)	Overall health (+*) QL: Quality of life (+)
	**The Good Mood Well-being Intervention** *Pynnönen K, 2018* D: QN (RCT); M: In-person; I: Group-based*; N=*1167	Loneliness (+)	Togetherness (+) Attachment (+) Social integration (+/-)	-
	**Volunteering and Socializing Activities in City Parks** *Gagliardi C, 2020* D: MM; M: In-person; I: Self-directed*; N=*19	-	QL: Creation of strong relationship (+)	Self-reported health (+*) Independent living performance (NC)
	**Weekday Wow Factor Project** *Lowe JA, 2023* D: QL; M: In-person; I: Group-based*; N=*26	-	QL: Sense of belonging (+)	QL: Quality of life (+)
	**SELF-DIRECTED LEISURE ACTIVITY INTERVENTIONS** **(*n =* 10)**			
	**Computer and Internet Access Provided** *Mellor D, 2008 from Heins P, 2021* D: MM; M: NR; I: Self-directed*; N=*20	-	Social connectedness (NC) QL: Social connectedness (+)	-
	**Digital Technology Interventions** *Reviews from Adekpedjou R, 2023* D: QN (Umbrella review); M: NR ; I: Self-directed*; N=*NR	Loneliness (+)	Social isolation (+)	-
	**Leisure Activity Interventions** *Veazie S, 2019 from Freedman A, 2020* D: QN (Rapid review); M: In-person; I: NR *; N=*NR	Loneliness (+)	Social isolation (+)	-
	**Letter Writing to Help Others on Ageing** *Moieni M, 2021* D: QN (Cross-sectional); M: Virtual (Paper); I: Self-directed*; N=*73	Loneliness (NC)	Social support (NC)	-
	**Loneliness Alleviation Program (LAP)** *Ae-Ri J, 2023* D: QN (RCT); M: Virtual (Smartphone); I: Self-directed*; N=*40	Loneliness (+*)	-	Self-efficacy (+*)
	**Mobile Application“GezelschApp”** *Jansen-Kosterink SM, 2020 from Heins P, 2021* D: QN (Cohort); M: NR; I: Self-directed*; N=*41	Loneliness (+)	-	-
	**Plant Therapy** *Septianingtyas MC, 2023* D: QN (Quasi-experimental); M: In-person; I: Self-directed*; N=*32	Loneliness (+*)	-	-
	**The Lifestyle Engagement Activity Program (LEAP)** *Low LF, 2015 from Poscia A, 2018* D: QN (Quasi-experimental); M: In-person; I: NR*; N=*189	Loneliness (NC)	Social isolation (+*) Functional social support (+*)	QL: Well-being (+)
	**Using Internet-Based Applications** *Czaja SJ, 2015 from Todd E, 2022* D: MM; M: NR ; I: Self-directed; *; N=*NR	Loneliness (+*)	Social isolation (+*) Social support (+*)	-
	**Using Internet-Based Applications** *Fokkema T, 2007 from Todd E, 2022* D: MM; M: NR ; I: Self-directed; *; N=*NR	Loneliness (+*)	-	-
	**ONE-TO-ONE LEISURE ACTIVITY INTERVENTIONS** **(*n =* 6)**			
	**Caring Callers Program** *Fields NL, 2023* D: QL; M: Virtual (Telephone); I: One-to-one*; N=*18	QL: Loneliness (+)	QL: Feelings of reciprocity (+)	QL: Self-confidence (+) QL: Overall well-being (+)
	**Foster Grandparent Programme** *Rook KS, 2003 from Dickens AP, 2011* D: QN (Quasi-experimental); M: In-person; I: One-to-one*; N=*180	Loneliness (NC)	New relationships (+) New social ties built (+) Functional social support (NC)	-
	**Letter Writing** *Long EM, 2023* D: QN (Pre-post); M: Virtual (Paper) ; I: One-to-one; N=34	Loneliness (+*)	-	Self-reported health status (NC)
	**Senior Companion Program (SCP)** *Butler SS, 2006 from Hagan R, 2014* D: MM; M: In-person; I: One-to-one*; N=*66	Loneliness (+)	-	-
	**The Personalised Citizen Assistance for Social Participation (APIC)** *Levasseur M, 2016* D: MM; M: In-person; I: One-to-one*; N=*16	-	Difficulties in social environment (+*) Satisfaction with social participation (+*) QL: Creating new or reviving social relationships (+) QL: Social connectedness (+)	-
	**The Seniors Connecting: Greenvale Community Exercise Program** *Bartlett H, 2013* D: QN (Pre-post); M: In-person; I: One-to-one*; N=*31	Loneliness (NC)	Social support (NC)	Subjective well-being (+) Overall health (+)
	**LEISURE ACTIVITY INTERVENTIONS WITH MIXED LEVEL OF INTERACTIONS** **(*n =* 8)**			
	**Connect 60+ Wellness Program** *Naseri C, 2023* D: QN (Pre-post); M: Mixed (In-person and virtual (computer, smartphone)); I: Mixed (Group-based, self-directed)*; N=*47	-	Social connectedness (+*)	-
	**Exercise, Activity or Counselling Intervention** *Pynnönen K, 2018 from Ibrahim AF, 2022* D: QN (RCT); M: In-person; I: Mixed (Group-based, one-to-one); N = 223	Loneliness (NC)	Social integration (+*)	-
	**Experience Corps** *Fried LP, 2004 from Pool MS, 2017* D: QN (RCT); M: In-person; I: Mixed (Group-based, one-to-one)*; N=*128	-	QL: Amount of social activity (+*)	-
	**Experience Corps** *Morrow-Howell N, 2014* • D: QN (Observational); M: In-person; I: Mixed (Group-based, one-to-one)*; N=*338	-	Amount of activity engagement (+) New social connections (+) Confidence in social interactions (+)	QL: Problem solving abilities (+)
	**Experience Corps** *Parisi JM, 2015 from Pool MS, 2017* • D: QN (RCT); M: In-person; I: Mixed (Group-based, one-to-one)*; N=*702	-	QL: Amount of social activity (+*)	-
	**Experience Corps Program** *Carlson MC, 2008; Fried LP, 2004; Tan EJ, 2006 from Krzeczkowska A, 2021* D: QN (RCTs); M: NR ; I: NR *; N=*128	-	No. of people participants felt they could reach out to (+*) No. of adults seen in a week (NC) No. of adults one could depend on (NC)	Health status (NC)
	**Quartier Agil Intervention** *Thiel C, 2022* D: QN (Pre-post); M: Virtual (Smartphone); I: Mixed (Group-based, self-directed)*; N=*39	-	Social participation (NC)	Quality of life (NC)
	**Recreation, Education, and Socialization for Older Learning Veterans (RESOLV)** *Juang C, 2021* D: MM; M: Virtual (Telephone); I: Mixed (Group-based, one-to-one)*; N=*32	Loneliness (+*)	-	-
	**INTERACTION LEISURE ACTIVITY INTERVENTIONS WITH UNCLEAR LEVEL OF INTERACTIONS** **(*n =* 5)**			
	**Experience Corps Program** *Gruenewald TL, 2016; Parisi JM, 2015 ****from*** *Krzeczkowska A, 2021* D: QN (RCTs); M: NR ; I: NR *; N=*702	-	Social activity (+)	QL: Well-being (+)
	**Recreational Activities** *Clift S, 2012; Coffman D, 2009; Cohen GD, 2007; Davidson JW, 2014; Hillman S, 2002; Kattenstroth JC, 2013; Koga M, 2001; Solé C, 2010; Teater B, 2014; Verghese J, 2003; Yap AF, 2017; Cohen GD, 2006; Dickens AP, 2011; Moody E, 2012; Low LF, Arnetz BB, 1983; Baumgarten M, 1988; Gleibs IH, 2011; Hemingway A, 2013; Pettigrew S, 2008; Tse T, 2005; Valadez AA, 2006 ****from*** *Paquet C, 2023* D: MM; M: NR ; I: NR *; N=*NR	Loneliness (+) QL: Loneliness (+)	Social isolation (+) QL: Social isolation (+)	-
	**REPRINTS** *Fujiwara Y, 2009 ****from*** *Krzeczkowska A, 2021* D: QN (Quasi-experimental); M: NR ; I: NR *; N=*141	-	Social network scores (+*) No. of distant friends (+*) Contact with children (+*) Providing support to friends (+*) Social support received (+*)	-
	**REPRINTS** *Sakurai R, 2016 ****from*** *Krzeczkowska A, 2021* D: QN (Quasi-experimental); M: NR ; I: NR *; N=*118	-	Frequency of friends interaction (NC) Social functioning (NC)	-
	**The Healthy Aging Web-Based Activity Programme** *Cohen-Mansfield J, 2021 ****from*** *Li M, 2023* D: QL; M: Virtual (Not Specified); I: NR *; N=*49	QL: Loneliness (+/-)	QL: Social contacts (-)	-
**Mind-body Interventions** **(*n=*14)**	**IN-PERSON MIND-BODY INTERVENTIONS (*n*** **= 11)**			
	**Compassion Meditation (CM)** *Malaktaris A, 2022* D: MM; M: In-person; I: Group-based*; N=*NR	Loneliness (+)	Social connectedness (+) QL: Interpersonal skills (+)	Satisfaction with life (+*)
	**Daily Gratitude Exercise** *Bartlett MY, 2019* •D: QN (RCT); M: In-person; I: Self-directed*; N=*42	Loneliness (+)	-	-
	**Dance Movement Therapy (DMT)** *Ho RT, 2020* D: QN (RCT); M: In-person; I: Group-based*; N=*204	Loneliness (+*)	-	-
	**Laughter Yoga** *Kuru N, 2018 from Alici NK, 2020* D: QN (Quasi-experimental); M: In-person; I: Group-based*; N=*50	Loneliness (NC) Social loneliness (+*)	-	-
	**Meditation Program** *Pandya SP, 2021* D: QN (RCT); M: In-person; I: Group-based*; N=*378	Loneliness (+*)	-	Resilience (+*) Life satisfaction (+*)
	**Mindfulness Based Stress Reduction Program (MBSR)** *Creswell JD, 2012 from Hagan R, 2014* D: QN (RCT); M: In-person; I: Group-based*; N=*40	Loneliness (+*)	-	-
	**Preventing Loss of Independence through Exercise (PLIE)** *Chao LL, 2021* D: QN (Pre-post); M: In-person; I: Group-based*; N=*18	-	Social isolation (+*)	-
	**Tai Chi Easy (TCE)** *Larkey LK, 2023* D: QN (Pre-post); M: In-person; I: Group-based*; N=*21	-	Total connection (+*)	-
	**Tai Chi Intervention** *Taylor-Pillae RE, 2006 from Pool MS, 2017* D: QN (Quasi-experimental); M: In-person; I: Group-based*; N=*39	-	Social support (+*)	-
	**Yoga for Loneliness** *Panigrahi M, 2023* D: QN (Pre-post); M: In-person; I: Group-based*; N=*44	Loneliness (+*)	-	-
	**Yoga Intervention** *Wang DS, 2010* • D: QN (Pre-post); M: In-person; I: Group-based; N = 18	Loneliness (+)	-	-
	**VIRTUAL MIND-BODY INTERVENTIONS** **(*n =* 3)**			
	**Remote Chair Yoga (CY)** *Park J, 2022* D: QN (Pre-post); M: Virtual (Computer); I: Group-based*; N=*11	Social loneliness (NC) Emotional loneliness (+*)	-	-
	**Spiritual Counselling Programme (SCP)** *Pandya SP, 2021* D: QN (Pre-post); M: Virtual (Computer); I: Mixed (Group-based, pair-based, self-directed)*; N=*822	Loneliness (+*)	-	-
	**Virtually Mentally Stimulating Activities (VMSA) Program** *Weaver C, 2022* D: QN (Cross-sectional); M: Virtual (Computer); I: Group-based*; N=*4	Loneliness (+)	-	Life satisfaction (+)
**Physical activity or exercise-based interventions** **(*n=*48)**	**NON-TECHNOLOGY-MEDIATED PHYSICAL ACTIVITY INTERVENTIONS** **(*n =* 24)**			
	**Aerobic Intervention** *McAuley E, 2000 ****from*** *Cohen-Mansfield J, 2015* D: QN (RCT); M: In-person; I: Group-based; N = 174	Loneliness (+*)	-	-
	**Connecting Seniors to Their Community Through Walking Program** *Walters K, 2022* D: QL; M: In-person; I: Group-based*; N=*28	-	Connectedness with community (+)	-
	**ENJOY Project** *Levinger P, 2020* D: QN (Prospective controlled trial); M: In-person; I: Group-based*; N=*95	Loneliness (+*)	Social isolation (NC)	Self-rated quality of life (+*)
	**Exercise for Those with Dementia** *Long A, 2020* D: MM; M: In-person; I: Group-based*; N=*16	Loneliness (+)	-	QL: Independence (+) QL: Health (+) QL: Social well-being (+) Quality of life (-)
	**Exercise Intervention** *Ho RT, 2020* D: QN (RCT); M: In-person; I: Group-based*; N=*204	Loneliness (NC)	-	-
	**Floorball (FB)** *Pedersen MT, 2022* D: MM; M: In-person; I: Group-based *; N=*29	-	-	-
	**General Physical Activity Interventions** *(Veazie S, 2019 from Freedman A, 2020)* D: QN (Rapid review); M: In-person; I: Group-based*; N=*NR	Loneliness (+)	Social isolation (+)	-
	**Gippsland Health and Well-being Program** *Dabkowski E, 2021* D: QL; M: In-person; I: Group-based*; N=*23	-	QL: Social connection (+)	-
	**Group Fitness Program** *Bidonde MJ, 2009 from Johnstone G, 2021* D: QL; M: In-person; I: Group-based*; N=*9	-	QL: Social networks (+)	-
	**Group Outdoor Health Walks (GOHW)** *Irvine KN, 2022* D: QL; M: In-person; I: Group-based*; N=*9	QL: Loneliness (+)	QL: Social isolation (+) QL: Social interaction (+)	-
	**Health Behaviour Interventions/ Health Education Interventions** *McKay H, 2018; Mendoza-Ruvalcaba NM, 2016 from Tcymbal A, 2025* D: MM; M: In-person; I: NR *; N=*NR	-	Social participation (+/-)	-
	**Healthy Ageing Promotion Program for You (HAPPY)** *Merchant RA, 2021* D: QN (Cross-sectional); M: In-person; I: Group-based*; N=*700	-	Social isolation (+*)	Frailty status (+*)
	**Leveraging Exercise to Age in Place (LEAP)** *Mays AM, 2021* D: QN (Pre-post); M: In-person; I: Group-based; N = 382	Loneliness (+*)	Social connectedness (+*)	-
	**Physical Activity Promotion Interventions** *Barbosa BT, 2019; Barragan C, 2021; Bidonde MJ, 2009; Brustio PR, 2018; Cedergren A, 2007;Chan AW, 2017; Dionigi R, 2007; Ehlers DK, 2017; Figueira HA, 2012; Gomeñuka NA, 2019; Kohut ML, 2006; Komatsu H, 2017; Liu YWJ, 2013; Maki Y, 2012;McAuley E, 2000; Streber A, 2017; Wang DS, 2010; Wikman JM, 2017 from Tcymbal A, 2022* D: MM; M: In-person; I: NR *; N=*NR	-	Social participation (+/-) QL: Social connectedness (+) QL: Communication (+) QL: Social engagement (+)	-
	**Self-Organized Swimming Groups for Healthy Ageing** *Costello L, 2019* D: QL; M: In-person; I: Group-based*; N=*17	-	QL: Social connectedness (+) QL: Group cohesion (+)	-
	**Silver Sneakers Exercise Program** *Brady S, 2020* D: QN (Quasi-experimental); M: In-person; I: Group-based; N =3143	Loneliness (+)	Social isolation (+)	-
	**Social Activity with Physical Activity Component** *Austin EN, 2006; Boyes M, 2013;Gagliardi C, 2019; Johnson JK, 2020; Vadineia da Silva M, 2016 from Tcymbal A, 2022* D: MM; M: In-person; I: NR *; N=*NR	-	Social participation (+/-) QL: Social participation (+)	-
	**Steps for Change** *Rodriguez Espinosa P, 2023* D: QL; M: In-person; I: Group-based*; N=*35	-	QL: New connections (+) QL: Social connection (+)	Well-being (+*) QL: Well-being (+)
	**Supervised Activities** *Pynnönen K, 2018 from Douglas NF, 2023* D: QN (Cross-sectional); M: In-person; I: Mixed (Group-based, one-to-one)*; N=*105	-	Social integration (+*)	-
	**Supervised Seniors Exercise Park Program** *Ng YL, 2023* D: QL; M: In-person; I: Group-based; N = 24	-	QL: Develop new friendships (+)	-
	**The Choose to Move (CTM) Intervention** *McKay H, 2018* D: QN (Controlled before and after); M: In-person; I: Group-based*; N=*458	Loneliness (+*)	Social exclusion (+/-)	QL: Feelings of control (+) QL: Confidence (+)
	**The Physical Activity Intervention for Loneliness (PAIL)** *Shvedko AV, 2020* D: MM; M: In-person; I: Group-based*; N=*25	Loneliness (NC)	New friendships (+) QL: Bonding (+)	QL: Coping (+)
	**Walk 'n' Talk intervention** *Hwang J, 2019* D: QL ; M: In-person; I: Group-based*; N=*16	QL: Loneliness (+)	QL: Sense of belonging (+)	-
	**Walking Exercise Program** *Rejeski W, 2014 from Pool MS, 2017* D: QN (RCT); M: In-person; I: Group-based*; N=*178	-	Social functioning (+*)	-
	**TECHNOLOGY-MEDIATED PHYSICAL ACTIVITY INTERVENTIONS** **(*n =* 16)**			
	**Active Plus** *Boekhout JM, 2021* D: QN (RCT); M: Virtual (Computer); I: Self-directed*; N=*585	Social loneliness (+*) Emotional loneliness (+*) Loneliness (NC)	-	-
	**Choose to Move (CTM)** *McKay HA, 2023* D: QN (Pre-post); M: Virtual (Computer); I: Mixed (Group-based, one-to-one)*; N=*1012	Loneliness (+*)	Social isolation (+*)	Health related quality of life (NC)
	**Computer Exercise Advisor** *Bickmore TW, 2005 from Cohen-Mansfield J, 2015* D: QN (RCT); M: Virtual (Computer); I: One-to-one*; N=*21	Loneliness (NC)	-	-
	**Exercise Group** *Düzel S, 2022* D: QN (Pre-post); M: Virtual (Tablet); I: Mixed (Group-based, self-directed)*; N=*39	Loneliness (NC)	-	-
	**Exergaming** *Zhu YZ, 2023* D: QN (Quasi-experimental); M: Virtual (Computer, television); I: Group-based; N = 69	Loneliness (-*)	-	Self-perceived health (NC)
	**Personalized Fall Prevention Exercise Program** *Baez M, 2017* D: QN (RCT); M: Virtual (Tablet); I: Group-based*; N=*37	Loneliness (+)	-	-
	**Personalized Online Exercise Programme** *Zengin Alpozgen A, 2022 ****from*** *Li M, 2023* D: QN (RCT); M: Virtual (Not Specified); I: NR *; N=*30	Loneliness (+)	-	-
	**Playing Wii** *Kahlbaugh PE, 2011 from Heins P, 2021* D: QN (Clinical controlled trial); M: In-person; I: One-to-one*; N=*36	Loneliness (+*)	-	-
	**Tablet for Exercise** *Báez M, 2016 from Rivera-Torres S, 2021* D: QN (RCT); M: Virtual (Computer); I: Group-based*; N=*37	Loneliness (NC)	-	-
	**Tele-Exercise Program** *Alpogen AZ, 2022* D: QN (RCT); M: Virtual (Computer); I: Group-based*; N=*30	Loneliness (+*)	-	Health related quality of life (+*)
	**Using the Internet for Applications with a Phone and Tablet** *Baez M, 2017 from Todd E, 2022* D: MM; M: Virtual (Smartphone, tablet); I: NR *; N=*NR	Loneliness (NC)	-	-
	**Virtual Space Messaging** *Isaacson M, 2019 from Rivera-Torres S, 2021* D: MM; M: Virtual (Computer); I: Group-based*; N=*40	QL: Loneliness (+)	QL: Social engagement (+)	-
	**Wii Exergame** *Li J, 2017 from Choi HK, 2021* D: QN (N-RCT); M: Virtual (Wii) ; I: Self-directed*; N=*30	Loneliness (NC)	-	Life satisfaction (NC)
	**Wii Fit U** *Chao Y, 2018 from Li J, 2018* D: QN (Pre-post); M: In-person; I: NR *; N=*12	-	QL: Connection with others (+)	-
	**Wii Kinect Exercise Games** *Xu X, 2016 from Li J, 2018* D: QN (Pre-post); M: In-person; I: Group-based*; N=*89	Loneliness (+*)	Sociability (+*) Social anxiousness (NC)	-
	**Wii Sports + Wii Sports Resort Games** *Wollersheim D, 2010 from Chao YY, 2015* D: QN (Pre-post); M: In-person; I: Group-based*; N=*15	-	Amount of social connections (+)	-
	**COMBINATION OF TECHNOLOGY- AND NON-TECHNOLOGY-MEDIATED PHYSICAL ACTIVITY INTERVENTIONS** **(*n =* 3)**			
	**Choose to Move (CTM)** *Franke T, 2021* D: MM; M: Mixed (In-person and virtual (telephone); I: Mixed (Group-based, one-to-one)*; N=*458	Loneliness (+*)	-	-
	**Choose to Move (CTM)** *McKay HA, 2021* D: QN (Cross-sectional); M: Mixed (In-person and virtual (telephone)); I: Mixed (Group-based, one-to-one)*; N=*458	Loneliness (NC)	Social isolation (+/-)	Self-perceived health (+*)
	**SITLESS Intervention** *Blackburn NE, 2021* D: QL; M: Mixed (In-person and virtual (telephone); I: Mixed (Group-based, one-to-one)*; N=*150	-	QL: Sense of belonging (+) QL: Social relationships (+)	-
	UNCLEAR MODE OF DELIVERY (***n =* 5)**			
	**Exercise and Social Engagement Interventions** *Baez M, 2017; Chan AW, 2017;Larsen RT, 2021; Li J, 2017; ****from*** *Yu DS, 2023* D: QN (RCT, N-RCT); M: NR; I: NR ; N = NR	Loneliness (NC)	-	-
	**Exercise Interventions** *Baez, M, 2017; Larsen RT, 2021;* *Li J, 2017; Jing L, 2018; McAuley E, 2000; Mutrie N, 2012; Pinheiro HA, 2020 ****from*** *Yu DS, 2023* D: QN (RCT, N-RCT); M: NR; I: NR *; N=*NR	Loneliness (+/-)	-	-
	**Exergames Program** *Unbehaun D, 2018 from Heins P, 2021* D: QL; M: NR ; I: NR *; N=*23	-	QL: Social interaction (+)	-
	**Physical Activity Intervention** *Reviews from Adekpedjou R, 2023* D: QN (Umbrella review); M: NR ; I: NR*; N=*NR	-	Social functioning (+)	-
	**Physical Activity Interventions** *Agmon M, 2011; Chao YY, 2018; Jung Y, 2009; Rendon AA, 2012; Rosenberg D, 2012; Wollersheim D, 2010; Xu X, 2016; Bartlett H, 2013; Hopman-Rock M, 2002; Kamegaya T, 2014; McAuley E, 2000; Seino S, 2017 ****from*** *Paquet C, 2023* D: MM; M: NR ; I: NR *; N=*NR	Loneliness (+) QL: Loneliness (+)	Social network size (+) Social support (+) QL: Social network size (+)	-
**Spiritual Interventions** **(*n=*3)**	**Christian Faith-Based Intervention** *Don'L B, 2023* D: QN (Pre-post); M: Virtual (Computer); I: Mixed (Group-based, one-to-one)*; N=*16	Social loneliness (+*)	-	Social quality of life (NC)
	**mHealth-Supported Volunteer-Assisted Spiritual Well-Being (mVS)** *Lou VW, 2023* D: QN (Quasi-experimental); M: In-person; I: One-to-one*; N=*161	-	Relationship with others (+*) Relationship with family (NC)	-
	**Religious Quran Intervention** *Borji M, 2020* D: QN (Semi-experimental); M: In-person; I: Group-based*; N=*88	Loneliness (+*)	-	-

### Social resource-related interventions (*n*=196) (Table 2)

ICT-based (*n=*43), intergenerational (*n=*33), aging in place (*n=*31), socially assistive robots and computer agents (*n=*29), befriending (*n=*27), peer support group (*n=*23), mentorship, and mentorship (*n=*11) interventions.

*ICT-based interventions* included social networking platforms/apps (*n=*30), device-mediated communication strategies (*n=*9) or a combination of the two (*n=*2). Most of the social networking platforms/apps (70%) and device-mediated communication strategies (71%) showed non-significant improvements in social outcomes. Findings for loneliness outcomes were mixed with mostly non-significant improvement (70%) or no change (50%) for social networking platforms/apps, and non-significant improvement (71%), mixed findings (43%) or no change (14%) for device-mediated communication strategies. *Intergenerational interventions* included five types: strategies designed to promote or build social connections and engagement (*n=*12), to share experiences and memories including reminiscence therapy (*n=*7); community-based (*n=*6) or general (*n=*4) intergenerational programs; and interventions that promote socialization through activities and skills training (*n=*4). Most interventions from these five intergenerational intervention categories (range 67%-100%) significantly reduced loneliness outcomes. Similarly, most interventions across four of the five intervention categories (range 50%-100%) significantly improved health and wellbeing outcomes. Social outcomes across the five intervention categories mostly improved (range 50%-80%), but these were not significant. *Aging in place interventions* included home visit interventions (*n=*14), day care/seniors centres (*n=*8), telehealth interventions (*n=*7), and meal delivery interventions (*n=*4). Loneliness outcomes significantly improved in 40% of day care/seniors centre interventions, 40% of telehealth interventions, and 75% of meal delivery interventions; and no change was observed among 57% of home visit interventions. Among social outcomes, day care/seniors centres showed significant (50%) and non-significant (50%) improvement; and 44% of home visit interventions and 40% of telehealth interventions found non-significant improvement. Among health and wellbeing outcomes, most home visit or day care/seniors centre interventions (67% for both) showed significant improvement; one telehealth intervention showed non-significant improvement and meal delivery showed no change among the two interventions that investigated this outcome. *Socially assistive robots (n=16) and computer agent (n=14) interventions.* Among robot interventions, most reduced loneliness (67%), social (100%) and health and wellbeing (67%) outcomes non-significantly. Most computer agent interventions non-significantly improved social (83%) and wellbeing (67%) outcomes and of nine interventions that investigated loneliness outcomes, 44% significantly and 44% non-significantly showed improvement. *Befriending interventions* included technology mediated (*n=*14) and non-technology mediated (*n=*10) befriending interventions, and a combination of the two (*n=*2). None of the befriending interventions improved outcomes significantly, but most showed positive impact on loneliness (range 60%-80%), social (89%-100%) or health and wellbeing (40%-67%) outcomes regardless of whether they were mediated by technology or not. *Peer support group interventions* included those that were delivered in-person (*n=*16) or virtually (*n=*6). Half of in-person peer support interventions significantly reduced loneliness and health and wellbeing outcomes, and most (82%) non-significantly improved social outcomes. Most virtual peer support interventions (67%) improved social outcomes and half improved health and wellbeing outcomes but not significantly. *Mentorship interventions* included in-person delivered (*n=*8) or a combination of in-person and/or virtually delivered (*n=*3). Most in-person mentorship interventions (67%) significantly reduced loneliness outcomes. Of interventions that investigated social outcomes (*n=*3), most (75%) showed non-significant positive impact. When mentorship interventions were delivered in-person and/or virtually, findings were mixed with half of interventions finding significant improvement and half finding no change in loneliness, social or health and wellbeing outcomes.

### Self-management-related interventions (*n*=157) (Table 3)

Self-management education (*n=*106), psychological self-management interventions (*n=*43), and social prescribing or asset-based (*n=*8).

*Self-management education interventions* included social health training (*n=*30), general health training (*n=*23), combination of technology device and Internet training (*n=*21), technology device training (*n=*16), caregiver support education (*n=*7), peer-based self-management education (*n=*5), and self-management skills training (*n=*4). Among social health training interventions, 70% significantly reduced loneliness and 40% significantly improved wellness outcomes, and most interventions (79%) non-significantly improved social outcomes. General health training interventions had mixed results with a third showing significant impact and 31%-40% showing no change in loneliness and social outcomes; wellness outcomes non-significantly improved in 57% of interventions. Most technology device training interventions had no change in loneliness (63%) or wellness (60%) outcomes but showed a non-significant improvement in social outcomes among 63% of interventions. 40% of caregiver support education interventions significantly reduced loneliness but showed mixed results for social and health and wellness outcomes. Among peer-based self-management education interventions, 60% non-significantly improved social outcomes and the two interventions that investigated health and wellness outcomes showed significant improvement. Most self-management skills training interventions (67%) improved social outcomes significantly but found mixed results for loneliness outcomes. *Psychological self-management interventions* included behavioural activation and cognitive behavioural therapy (CBT) interventions (*n=*22); a combination of different psychological interventions (e.g., CBT + mindfulness; music + reminiscence) (*n=*11), psychosocial interventions (*n=*5) and reminiscence therapy (*n=*5). Most behavioural activation or CBT interventions significantly reduced loneliness (63%) and improved health and wellbeing (67%) outcomes and half non-significantly improved social outcomes. Of interventions that included a combination of different psychological strategies, most showed positive impact for loneliness (67%) and social (60%) outcomes, but these were not significant. Among psychosocial interventions, none of the interventions showed impact in loneliness outcomes, 75% also showed no change in social outcomes but there was a non-significant improvement in health and wellbeing outcomes in two interventions. Among reminiscence therapy interventions, most (67%) significantly reduced loneliness outcomes, all interventions non-significantly improved social outcomes, and none of the three interventions that investigated health and wellness outcomes showed impact. *Social prescribing or asset-based interventions* included eight interventions, most of which (80%) significantly reduced loneliness and half significantly improved social outcomes; none investigated health and wellbeing outcomes.

### Social behavioural activity interventions (*n*=140) (Table 4)

Physical activity (*n=*48) leisure activity (*n=*48), arts-based (*n=*27), mind-body (*n=*14), and spiritual (*n=*3) interventions.

*Physical activity interventions*: Among 16 technology-mediated physical activity interventions (including seven exergaming interventions), results were mixed for loneliness outcomes as 43% of interventions showed significant reduction in loneliness and 43% showed no change. For social outcomes, many interventions showed significant (40%) and non-significantly improvement, while 75% of interventions that investigated health and wellness outcomes found no change. Among physical activity interventions that were not technology mediated, some interventions investigating loneliness outcomes showed significant (36%) and non-significant (45%) improvement. Social outcomes improved non-significantly among 57% of interventions; and many interventions showed significant (50%) and non-significantly (67%) improvement in health and wellbeing outcomes. *Leisure activity interventions*: included group-based (*n=*19), self-directed (*n=*10), one-to-one (*n=*6) or mixed level of interaction (*n=*8) interventions. Many leisure activity interventions significantly reduced loneliness and improved health and wellbeing outcomes with group-based (43% and 55%, respectively) or self-directed (44% and 50%, respectively) interaction with the intervention. Results were mixed for strategies that were one-to-one or mixed level of interaction for loneliness and health and wellness outcomes. Social outcomes were non-significantly improved for most leisure interventions regardless of the type of interaction participants had with the intervention: group-based (55%), self-directed (50%), one-to-one (75%), mixed (71%). *Arts-based interventions*: included direct engagement (*n=*21), receptive engagement (*n=*3) or a combination of the two (*n=*2). Many arts-based interventions significantly reduced loneliness regardless of whether they were direct or receptive engagement strategies (44% and 50%, respectively). Most direct engagement in the arts strategies also improved social (71%) and health and wellbeing (75%) outcomes, but these were not significant. All the two arts-based interventions that included both direct and receptive engagement showed positive, non-significant reduction in loneliness and social outcomes. *Mind-body interventions*: Whether delivered in-person (*n=*11) or virtually (*n=*3), most mind-body intervention significantly reduced loneliness outcomes (44% and 67%, respectively). Among in-person strategies, most (75%) significantly improved social outcomes, and the two interventions that investigated health and wellbeing outcomes, also showed significant improvement. *Spiritual interventions:* Of three spiritual interventions, two significantly reduced loneliness. Social and health and wellness outcomes were mixed as one intervention significantly improved social outcomes while another showed no change in health and wellness outcomes.

### General resource interventions (*n*=2) (Supplement Table 2)

Two interventions were identified: A single mixed method environmental study showed that renovating the neighborhood open spaces had some positive effects on social participation. The second intervention involving a hearing aid and hearing diary showed a nonsignificant decrease in loneliness from baseline to 6-month follow-up.

### Interventions with potential to help older adults during isolation measures due to infectious disease outbreaks

Of the 495 interventions that were identified in our scoping review, 189 (38%) reported at least one effective loneliness, social or health and wellbeing outcome across the three social frailty domains. Of these, we identified 63 interventions (33%) that may be considered to address social frailty in older adults during infectious disease outbreaks requiring social distancing and isolation (i.e., interventions that don’t require physical contact with a person) (Table 5). Effective interventions were clustered within six intervention categories across three social frailty domains: (i) psychological self-management (*n=*11) and (ii) self-management education (*n=*9) within the self-management domain; (iii) leisure activity (*n=*11) and (iv) physical activity (*n=*8) interventions within the social behavioural activities domain; and (v) ICT (*n=*10) and (vi) socially assistive robots and computer agents (*n=*5) within the social resource domain. Loneliness outcomes were the most frequently investigated across the six intervention categories. Additionally, the highest proportion of effective interventions were those that reduced loneliness outcomes (70-100%) compared with the proportion of interventions that improved social (range 18-60%) or health and wellbeing outcomes (range 11-38%). There were two interventions categories (self-management education, leisure activity) that included interventions that had significant impact across all three outcome categories: loneliness (89% and 73% respectively), social (44% and 45%, respectively), and health and wellbeing (11%, 27%). Among 10 effective ICT-based interventions, 70% reduced loneliness outcomes and 60% improved social outcomes. Among 11 psychological self-management interventions, 82% reduced loneliness outcomes, and 18% improved social outcomes. Among five effective computer agent or robot interventions, all reduced loneliness and one improved self-rated health.

**Table 5:** Summary of effective social frailty interventions that may be feasible to be used during situations requiring social isolation (*n=*64)

**Intervention Type** (number of studies)	**Intervention name** *Author, year* D=data type (study design); M=mode of delivery; I=level of interaction*; N=*number of participants; MM=mixed-/multi methods; NR=NR; QL=D: QL; QN=D: QN	**Outcomes reported as effective** *(‘effective’ is defined according to what a study reported as a statistically significant positive change in an outcome)*
**1. Social Resource-related Interventions** (***n=*19)**		
**Information Communication Technology (ICT) based interventions** (***n=*10)**	**SOCIAL NETWORKING PLATFORMS AND APPS** **(*n=*8)**	
	**Facilitator-led Remote Interactive Intervention for Loneliness, Quality of Life, and Social Support** *Liu CW, 2023* D: QN (RCT); M: Virtual (Smartphone); I: Group-based*; N=*100	• Quality of life
	**Health Enhancement Support System (CHESS)** *Leszko M, 2020 from Mao W, 2023* D: MM; M: Virtual (Computer); I: Group-based *; N=*48	• Loneliness
	**Remote Sharing with Family Members** *Noguchi T, 2022* D: QN (Quasi-experimental); M: Virtual (Television); I: Self-directed*; N=*115	• Frequency of talking time with families living together • Frequency of talking time with families not living together • Satisfaction for the relationship with families living together
	**Senior App Suite** *Goumopoulos C, 2017 from Heins P, 2021* D: QN (Cross-sectional); M: Virtual (Not specified) ; I: NR*; N=*22	• Loneliness
	**Social Networking at Home** *Goumopoulos C, 2017 from Rivera-Torres S, 2021* D: MM; M: Virtual (Computer, tablet); I: NR*; N=*20	• Loneliness
	**The Personal Reminder Information and Social Management (PRISM) System** *Czaja SJ, 2018 from Choi HK, 2021* D: QN (RCT); M: Virtual (Smartphone); I: Self-directed*; N=*244	• Loneliness • Social isolation • Social support
	**The Personal Reminder Information and Social Management (PRISM) System** *Czaja SJ, 2018 from Heins P, 2021* D: QN (Clinical controlled trial); M: Virtual (Not specified) ; I: NR*; N=*300	• Loneliness • Perceived social support
	**Virtual Classroom to Message** *Czaja SJ, 2018 from Rivera-Torres S, 2021* D: QN (RCT); M: Virtual (Computer); I: Group-based*; N=*224	• Loneliness • Social isolation
	**DEVICE-MEDIATED COMMUNICATION** **(*n=*2)**	
	**Smart Technology Interventions** *Dew MA, 2004; Hill W, 2006; Weinert C, 2011;* *Weinert C, 2008; Barrera M Jr, 2002; Billipp SH, 2001; Bond GE, 2010; Chiu T, 2009; Fokkema T, 2007; Gustafson DH, 2005; Kahlbaugh PE, 2011; Lieberman MA, 2005; Mahoney DF, 2003; Pierce LL, 2009; Samoocha D, 2011; Slegers K, 2008; Van Straten A, 2008; Torp S, 2008 from Morris ME, 2014* D: QN (RCT, cohort study); M: Virtual (Computer); I: Mixed (Group-based, self-directed, one-to-one)*; N=*NR	• Social support
	**Various Digital Tools to Support Social Engagement** *Choi M, 2012; Chen YR, 2016; Morris ME, 2014; Forsman AK, 2017 from Larsson E, 2020* D: MM; M: Virtual (Not specified) ; I: NR*; N=*NR	• Loneliness • Social isolation • Feelings of belonging
**Intergenerational interventions** (***n=*1)**	**COMMUNITY-BASED INTERGENERATIONAL PROGRAMS OR SERVICES** (***n=*1)**	
	**Good Neighbor Program** *Sandu S, 2021* D: QN (Observational); M: Virtual (Telephone); I: One-to-one*; N=*261	• Resilience
**Aging in place** (***n=*1)**	**HOME CARE - TELEHEALTH INTERVENTIONS** (***n=*1)**	
	**Care TV Duplex Video/Voice Network** *Van Der Heide LA, 2012* D: QN (Quasi-experimental); M: Virtual (Alarm, video); I: One-to-one*; N=*130	• Feelings of loneliness
**Socially Assistive Robots and Computer Agents** (***n=*5)**	**COMPUTER AGENTS** **(*n=*2)**	
	**Conversational Agent** *Ring L, 2013 from Choi HK, 2021* D: MM; M: Virtual (Computer); I: Self-directed*; N=*16	• Loneliness
	**Personal Voice Assistants (PVA)** *Jones VK, 2021* D: MM; M: Virtual (Amazon Echo); I: Mixed (Self-directed, one-to-one)*; N=*16	• Loneliness • Self-rated health
	**ROBOTS** **(*n=*3)**	
	**Animatronic Pet Program** *Tkatch R, 2021* D: QN (Quasi-experimental); M: In-person; I: Self-directed*; N=*216	• Loneliness
	**Parret Shaped Social Robot** *Lim J, 2023* D: QN (Quasi-experimental); M: In-person; I: Self-directed*; N=*64	• Loneliness
	**Shakespearean Text Fields** *N, 2021* D: QN (Quasi-experimental); M: In-person; I: One-to-one*; N=*15	• Loneliness
**Befriending interventions** (***n=*1)**	**TECHNOLOGY-MEDIATED BEFRIENDING INTERVENTIONS** **(*n =* 14)**	
	**Loneliness Helpline calls at the Friendship at Every Age Program** *Balta M, 2023* D: QN (Quasi-experimental); M: Virtual (Telephone); I: One-to-one*; N=*275	• Loneliness • Well-being
**Peer support group interventions** (***n=*1)**	**VIRTUAL PEER SUPPORT GROUP INTERVENTIONS** **(*n =* 6)**	
	**Koffee Klatch Support Chat Room** *Hill W, 2006 from Khosravi P, 2016* D: QN (RCT); M: Virtual (Computer); I: Group-based*; N=*183	• Social support
**2. Self-management-related interventions** (***n=*20)**		
**Psychological self-management interventions** **(*n=*11)**	**BEHAVIOURAL OR COGNITIVE BEHAVIOURAL THERAPY (CBT)** **(*n=*5)**	
	**Acceptance and Commitment Therapy (ACT)** *Zarling A, 2023* D: QN (Quasi-experimental); M: Virtual (Not Specified); I: One-to-one*; N=*529	• Loneliness • Resilience
	**Cognitive Behavioural Therapy** *Jarvis MA, 2019 from Hickin N, 2021* D: QN (RCT); M: Virtual (Computer); I: Mixed (Group-based, self-directed); N = 32	• Loneliness
	**Cognitive Behavioural Therapy** *Jing L, 2018 from Hickin N, 2021* D: QN (RCT); M: Virtual (Computer, telephone); I: Self-directed*; N=*80	• Loneliness
	**Cognitive Telephone Therapy Groups** *Evans RL, 1986 from Cummings SM, 2004* D: QN (RCT); M: Virtual (Telephone); I: Group-based*; N=*43	• Loneliness
	**Low-Intensity Cognitive Behavioral Therapy** *Jarvis MA, 2019* D: QN (RCT); M: Virtual (Computer, smartphone, tablet); I: Group-based*; N=*32	• Maladaptive social cognitions for loneliness and emotional deprivation • Overall loneliness • Social loneliness • Emotional loneliness
	**BEHAVIOURAL ACTIVATION INTERVENTIONS** **(*n=*4)**	
	**Behavioural Activation (BA)** *Gilbody S, 2021 from Li M, 2023* D: QN (RCT); M: Virtual (Telephone); I: NR *; N=*96	• Loneliness
	**Behavioural Activation in Social Isolation (BASIL)** *Gilbody S, 2021* D: QN (RCT); M: Virtual (Computer, telephone); I: Mixed (Self-directed, one-to-one)*; N=*96	• Loneliness
	**Lay-Coach-Facilitated, Videocall, Short-Term Behavioral Activation Intervention** *Choi NG, 2020* D: QN (RCT); M: Virtual (Computer); I: Self-directed*; N=*89	• Loneliness • Social interactions • Satisfaction with social support
	**Tele-delivered Behavioral Activation (BA)** *Bruce ML, 2021* D: QN (RCT); M: Virtual (Computer, telephone); I: One-to-one*; N=*89	• Loneliness • Social interaction • Satisfaction with social support
	**COMBINATION OF DIFFERENT PSYCHOLOGICAL INTERVENTIONS** (***n=*1)**	
	**Psychological Therapies** *Choi NG, Marti CN, 2020; Choi NG, Pepin R, 2020; Gilbody S, 2021 from Chau CMS, 2023* D: QN (RCT); M: Virtual (Computer, telephone); I: NR*; N=*NR	• Social engagement
	**PSYCHOSOCIAL INTERVENTIONS** **(*n=*1)**	
	**Telephone Crisis Program** *Morrow-Howell N, 1998 from Cohen-Mansfield J, 2015* D: QN (Quasi-experimental); M: Virtual (Telephone); I: One-to-one*; N=*61	• Amount of social contact • QL: Interpersonal contact
**Self-management education interventions** **(*n=*9)**	**GENERAL HEALTH AND WELL-BEING TRAINING AND EDUCATION** (***n=*1)**	
	**Video-Conferencing Program for Skill Development** *Yavuz C, 2023* D: QN (Cross-sectional); M: Virtual (Computer, smartphone); I: Group-based*; N=*92	• Loneliness
	**SM SKILLS TRAINING** (***n=*1)**	
	**eHealth Self-Management System** *Jung H, 2017 from Johnstone G, 2021* D: QN (Quasi-experimental); M: Virtual (Computer, telephone); I: Mixed (Self-directed, one-to-one)*; N=*64	• Social support
	**CAREGIVER SUPPORT EDUCATION** **(*n=*1)**	
	**Engage Coaching for Caregivers** *Van Orden KA, 2023* D: QN (Quasi-experimental); M: Virtual (Computer, telephone); I: One-to-one*; N=*30	• Loneliness • Social isolation • Quality of life
	**COMBINATION OF TECHNOLOGY DEVICE AND INTERNET TRAINING** **(*n=*4)**	
	**Esc@pe Program** *Fokkema T, 2007 from Cohen-Mansfield J, 2015* D: QN (Quasi-experimental); M: In-person; I: One-to-one*; N=*15	• Loneliness
	**Internet at Home - Esc@pe** *Fokkema T, 2007 from Johnstone G, 2021* D: MM; M: NR; I: NR*; N=*26	• Overall loneliness • Emotional loneliness
	**Skill Development Interventions** *Czaja SJ, 2018; Fields J, 2019 from Chau CMS, 2023* D: QN (RCT); M: Mixed (In-person and virtual (telephone, tablet); I: NR*; N=*NR	• Loneliness • Social support
	**Using Communication Technology** *Blažun H, 2012 from Casanova G, 2021* D: QN; M: Virtual (Computer); I: NR*; N=*58	• Loneliness
	**TECHNOLOGY DEVICE TRAINING** (***n=*2)**	
	**CATCH-ON Connect** *Wang S, 2023* D: QN (Quasi-experimental); M: Virtual (Tablet); I: One-to-one*; N=*129	• Loneliness
	**Personal Reminder Information and Social Management (PRISM) System** *Czaja SJ, 2018* D: QN (RCT); M: Virtual (Smartphone); I: Self-directed*; N=*200	• Loneliness • Social isolation • Perceived social support
**3. Social Behavioural Activity Interventions** (***n=*24)**		
**Arts-based interventions** **(*n=*2)**	**DIRECT ENGAGEMENT ARTS-BASED INTERVENTIONS** **(*n=*2)**	
	**Robot To Socialize** *Fields N, 2019 from Rivera-Torres S, 2021* D: QN (Pre-post); M: In-person; I: NR *; N=*15	• Loneliness
	**Montreal Museum of Fine Arts (MMFA)** *Beauchet O, 2022* D: QN (RCT); M: Virtual (Computer); I: Group-based*; N=*106	• Social isolation • Quality of life • Physical frailty
**Leisure Activity Interventions** **(*n=*11)**	**GROUP-BASED LEISURE ACTIVITY INTERVENTIONS** **(*n=*3)**	
	**Connection Through Calls: Seniors' Centre Without Walls (SCWW)** *Roland H, 2021* D: QN (Cross-sectional); M: Virtual (Telephone); I: Group-based*; N=*160	• Loneliness
	**Horticulture Therapy** *Ng K, 2018* D: QN (RCT); M: In-person; I: One-to-one*; N=*59	• Social connectedness • Positive relationships
	**Volunteering and Socializing Activities in City Parks** *Gagliardi C, 2020* D: MM; M: In-person; I: Self-directed*; N=*19	• Self-reported health
	**SELF-DIRECTED LEISURE ACTIVITY INTERVENTIONS** **(*n=*5)**	
	**Loneliness Alleviation Program (LAP)** *Ae-Ri J, 2023* D: QN (RCT); M: Virtual (Smartphone); I: Self-directed*; N=*40	• Loneliness • Self-efficacy
	**Plant Therapy** *Septianingtyas MC, 2023* D: QN (Quasi-experimental); M: In-person; I: Self-directed*; N=*32	• Loneliness
	**The Lifestyle Engagement Activity Program (LEAP)** *Low LF, 2015 from Poscia A, 2018* D: QN (Quasi-experimental); M: In-person; I: NR*; N=*189	• Social isolation • Functional social support
	**Using Internet-Based Applications** *Czaja SJ, 2015 from Todd E, 2022* D: MM; M: NR ; I: Self-directed; *; N=*NR	• Loneliness • Social isolation • Social support
	**Using Internet-Based Applications** *Fokkema T, 2007 from Todd E, 2022* D: MM; M: NR ; I: Self-directed; *; N=*NR	• Loneliness
	**ONE-TO-ONE LEISURE ACTIVITY INTERVENTIONS** **(*n=*1)**	
	**Letter Writing** *Long EM, 2023* D: QN (Pre-post); M: Virtual (Paper) ; I: One-to-one; *N*=34	• Loneliness
	**LEISURE ACTIVITY INTERVENTIONS WITH MIXED LEVEL OF INTERACTIONS** **(*n=*2)**	
	**Connect 60+ Wellness Program** *Naseri C, 2023* D: QN (Pre-post); M: Mixed (In-person and virtual (computer, smartphone); I: Mixed (Group-based, self-directed)*; N=*47	• Social connectedness
	**Recreation, Education, and Socialization for Older Learning Veterans (RESOLV)** *Juang C, 2021* D: MM; M: Virtual (Telephone); I: Mixed (Group-based, one-to-one)*; N=*32	• Loneliness
**Mind-body Interventions** **(*n=*2)**	**VIRTUAL MIND-BODY INTERVENTIONS** **(*n=*2)**	
	**Remote Chair Yoga (CY)** *Park J, 2022* D: QN (Pre-post); M: Virtual (Computer); I: Group-based*; N=*11	• Emotional loneliness
	**Spiritual Counselling Programme (SCP)** *Pandya SP, 2021* D: QN (Pre-post); M: Virtual (Computer); I: Mixed (Group-based, pair-based, self-directed)*; N=*822	• Loneliness
**Physical activity or exercise-based interventions** **(*n=*8)**	**NON-TECHNOLOGY-MEDIATED PHYSICAL ACTIVITY INTERVENTIONS** **(*n=*3)**	
	**Aerobic Intervention** *McAuley E, 2000 ****from*** *Cohen-Mansfield J, 2015* D: QN (RCT); M: In-person; I: Group-based; *N* = 174	• Loneliness
	**ENJOY Project** *Levinger P, 2020* D: QN (Prospective controlled trial); M: In-person (outdoor park); I: Group-based*; N=*95	• Loneliness • Self-rated quality of life
	**Steps for Change** *Rodriguez Espinosa P, 2023* D: QL; M: In-person; I: Group-based*; N=*35	• Well-being
	**TECHNOLOGY-MEDIATED PHYSICAL ACTIVITY INTERVENTIONS** **(*n=*5)**	
	**Active Plus** *Boekhout JM, 2021* D: QN (RCT); M: Virtual (Computer); I: Self-directed*; N=*585	• Social loneliness • Emotional loneliness
	**Choose to Move (CTM)** *McKay HA, 2023* D: QN (Pre-post); M: Virtual (Computer); I: Mixed (Group-based, one-to-one)*; N=*1012	• Loneliness • Social isolation
	**Playing Wii** *Kahlbaugh PE, 2011 from Heins P, 2021* D: QN (Clinical controlled trial); M: In-person; I: One-to-one*; N=*36	• Loneliness
	**Tele-Exercise Program** *Alpogen AZ, 2022* D: QN (RCT); M: Virtual (Computer); I: Group-based*; N=*30	• Loneliness • Health related quality of life
	**Wii Kinect Exercise Games** *Xu X, 2016 from Li J, 2018* D: QN (Pre-post); M: In-person; I: Group-based*; N=*89	• Loneliness • Sociability
**Spiritual Interventions** **(*n=*1)**	**Christian Faith-Based Intervention** *Don'L B, 2023* D: QN (Pre-post); M: Virtual (Computer); I: Mixed (Group-based, one-to-one)*; N=*16	• Social loneliness

## Discussion

Our scoping review identified 495 interventions across four broad domains of social frailty: social resource-, self-management-, social behaviour- and general resource-related [11] (Supplement file 2). The spread of effective interventions was nearly equal among social behaviour activity (44%) and self-management (41%) domains, followed by the social resource-related domain (33%). None of the interventions in the general resource related domain were effective. This was expected given that this category within Bunt *et al*’s social frailty model represents “non-specific or general resources” that fulfill social needs more indirectly and which tend to be contextual or an unmodifiable resource (e.g., educational level, income) [11, 43].

The most promising interventions were clustered around the behavioural activity and self-management domains of social frailty. Social behavioural activity interventions promote social behaviours or activities with the goal of fulfilling social needs (e.g., maintaining relationships, social participation) [43]. Most of the effective interventions in this domain were in the leisure activity (*n=*11) and physical activity (*n=*8) intervention categories. Effective leisure activities included horticulture therapy, volunteering and socializing, letter writing, and lifestyle engagement. Effective physical activities included technology-mediated (e.g., via computer, exergames) or non-technology-mediated (e.g., walking, outdoor park). Overall, both intervention categories had the greatest impact on loneliness outcomes (64% of leisure activity and 88% of physical activity interventions).

Interventions among the self-management domain aim to improve an individual’s ability to manage their behaviours, emotions and lifestyle toward improving overall health [44]. Most of the effective interventions in the self-management domain clustered within the psychological self-management (*n=*11) and self-management education (*n=*9) categories. Psychological self-management interventions included behavioural activation and CBT strategies. Self-management education interventions included skills training in general health, self-management, technology device and Internet use; and caregiver support education. Self-management strategies also had the greatest impact on loneliness outcomes as 82% of psychological and 89% of self-management education interventions significantly reduced loneliness. The positive impact of self-management interventions is not surprising given that a person’s ability to self-manage in general but particularly their social lives and activities is an important determinant of loneliness [46] and may also support healthy aging among community-dwelling older adults [47]. A person with stronger self-efficacy is more likely to do activities and to put in the effort to reach their goals [48–50]. Those who have higher coping self-efficacy (i.e., *“a measure of self-confidence in one’s ability to effectively manage challenges using skills in problem-solving, emotional regulation, and coping through social support”*) have significantly lower odds of loneliness [51]. These suggest that we need to incorporate self-efficacy skills development and as an important component of a future social frailty intervention.

We also identified several effective interventions that may be considered during isolation measures due to infectious disease outbreaks such as the COVID-19 pandemic (i.e., physical activity, leisure activity, psychological self-management, ICT, self-management education). Most of these were delivered virtually via a computer or smart device (tablet, smartphone). Although ICT and digital communication technologies offer opportunities for older adults to remain socially connected and reinforce their existing or new social connections and relationships [52], their impact has generally been mixed [27], which is consistent with our findings. Reasons for this may be that few high-quality studies investigating ICT-based interventions exist, and many ignore confounding factors such as sociodemographic data [53]. Digital technologies have become a prominent part of social contact, interactions, and communication in society, and digital social media in particular, can expedite personal interactions and contactless communication [54]. However, digital inequities among older adults can occur on multiple levels, particularly if they are economically disadvantaged. Many older adults are excluded from social networks and online connectivity because they cannot easily access the internet either because they don’t have access to computers and smart devices, or they don’t have internet connection [54, 55]. Furthermore, older adults may have differing knowledge, capacity, digital literacy, motivation and competence to access and engage with technology [55]. The COVID-19 pandemic further highlighted the depth of inequities among vulnerable populations related to gender, race, socioeconomic status, newcomer status, education social network quality and health literacy [55–57]. Older adults can only benefit from virtually delivered effective interventions if they have access to and can afford the technology (including the internet) and have digital literacy to benefit from services and resources, which are often only available via online government and other organization websites.

### Considerations for future work in social frailty

Our scoping review results have several important considerations for future work in social frailty. First, we must ensure that a future social frailty innovation is usable, affordable, and accessible by all older adults by considering all possible inequities that they may experience. Second, a future social frailty intervention should target elements from all social frailty domains: the social resources of older adults (e.g., friendships or care from family members), their personal activities or social behaviours (e.g., social participation), as well as their self-management ability to gain or maintain their social resources and activities (e.g., their ability to make and maintain friends or to initiate social participation). Social frailty is a multidimensional concept because it’s not just about the absence of social resources or restrictions but the absence of social behaviours and social activities (e.g., maintaining cohesive relationships or social participation) as well as the absence of self-management abilities (e.g., feeling empowered or having the ability to make decision) [11]. Another important consideration is that the overall wellbeing of older adults is dependent on fulfilling needs according to *their* individual and personal circumstances, social resources, activities and capabilities [11, 58]. Third, we should consider social prescribing alongside self-management as these strategies can be particularly helpful for more vulnerable populations such as those with social frailty. These strategies can support self-management by connecting older adults to non-clinical supports in their communities [59]. Of the eight social prescribing interventions that we identified, 80% significantly reduced loneliness and 50% significantly reduced social outcomes. Lastly, social frailty is an important risk factor for developing physical frailty in non-frail older adults [60]. As older adults increasingly rely on their informal social relationships for well-being (e.g., family and friends), it may be more prudent to address social frailty directly, which has the potential to improve outcomes across other frailty types (e.g., physical and psychological) in addition to the overall frailty state [61]. People with stronger compared with weaker social relationships have a 50% increased likelihood of survival [62], and as the number and types of social activities increases, so does social participation and positive self-perceived health [63]. This means that social frailty doesn’t have to be an inevitable part of aging.

### Strengths and limitations

Our study used rigorous scoping review methods adhering to JBI guidelines [36]. We identified 189 social frailty interventions from 263 included studies that were reported as effective, and a subset of 63 interventions that may be feasible to be adapted during infectious disease outbreaks requiring further social isolation and distancing (Table 5). To our knowledge, this is the first scoping review of social frailty interventions grounded in theory [11]. Existing reviews focus on investigating the prevalence of social frailty [20] or focusing only on technology-based interventions [29, 52, 64] or on specific aspects of social frailty outcomes (loneliness, social isolation or vulnerability [27, 28, 30]. An important gap was that none of the 495 identified interventions were designed for social frailty and only one intervention measured social frailty as an outcome [65]. Our study also had some limitations. We identified a large number of interventions with considerable heterogeneity in the type of identified interventions and outcomes. It was also challenging to organize outcomes because they were inconsistently defined (e.g., social participation, social isolation, social vulnerability, social connectivity). To overcome this challenge, reviewer pairs (IH, JM, KA, MK) iteratively created a codebook for classifying outcomes using qualitative content analysis [45] (Supplement file 3).

## Conclusions

Our scoping review identified promising interventions for socially frail older adults with self-management- and social behavioural activity-related strategies showing the highest rates of significantly positive impacts on loneliness, social, health and wellbeing outcomes. We also identified that psychological self-management, self-management education, leisure activity, physical activity, ICT and socially assistive robots and computer agent interventions delivered mostly virtually would be most feasible to help older adults during isolation measures due to infectious disease outbreaks such as the COVID-19 pandemic.

### Supplementary Information

Supplementary Material 1.

Supplementary Material 2.

Supplementary Material 3.

Supplementary Material 4.

Supplementary Material 5.

## Data Availability

All data generated or analysed during this study are included in this published article [and its supplementary information files].

## Abbreviations

OSF: Open Science Framework
PRISMA-ScR: Preferred Reporting Items for Systematic Reviews and Meta-Analyses extension for Scoping reviews
CADTH: Canadian Agency for Drugs and Technologies in Health
PICOS: Population, Intervention, Comparator/Context, Outcomes, Study design
JBI: Joanna Briggs Institute
ICT: Information Communication Technology
RCT: Randomized Controlled Trial
CBT: Cognitive Behavioural Therapy
ICT: Information Communication Technology

## References

[1] Chatterji S, Byles J, Cutler D, Seeman T, Verdes E (2015). Health, functioning, and disability in older adults–present status and future implications. Lancet..

[2] Statistics_Canada. Canada Yearbook. Seniors. 2012; http://www.statcan.gc.ca/pub/11-402-x/2012000/chap/seniors-aines/seniors-aines-eng.htm. Accessed February 2020.

[3] Kehler DS, Ferguson T, Stammers AN (2017). Prevalence of frailty in Canadians 18–79 years old in the Canadian Health Measures Survey. BMC Geriatrics..

[4] Ament BHL, de Vugt ME, Verhey FRJ, Kempen GIJM (2014). Are physically frail older persons more at risk of adverse outcomes if they also suffer from cognitive, social, and psychological frailty?. Eur J Ageing..

[5] Gobbens RJ, van Assen MA, Luijkx KG, Schols JM (2012). The predictive validity of the Tilburg Frailty Indicator: disability, health care utilization, and quality of life in a population at risk. Gerontologist..

[6] Markle-Reid M, Browne G (2003). Conceptualizations of frailty in relation to older adults. J Adv Nurs..

[7] Gobbens RJ, Luijkx KG, Wijnen-Sponselee MT, Schols JM. Frail elderly. Identification of a population at risk. Tijdschr Gerontol Geriatr. 2007;38(2):65-76. 17605284

[8] Freitag S, Schmidt S (2016). Psychosocial Correlates of Frailty in Older Adults. Geriatrics..

[9] Chen Yi-Ru R, Schulz PJ (2016). The effect of information communication technology interventions on reducing social isolation in the elderly: A systematic review.

[10] Bessa B, Ribeiro O, Coelho T (2018). Assessing the social dimension of frailty in old age: A systematic review. Arch Gerontol and Geriatr.

[11] Bunt S, Steverink N, Olthof J, van der Shans C, Hobbelen J (2017). Social frailty in older adults: a scoping review. Eur J Aging.

[12] MacLeod S, Musich S, Hawkins K, Alsgaard K, Wicker ER (2016). The impact of resilience among older adults. Geriatr Nurs..

[13] Jeste DV, Savla GN, Thompson WK (2013). Association between older age and more successful aging: critical role of resilience and depression. Am J Psychiatry..

[14] Gale C, Westbury L, Cooper C (2018). Social isolation and loneliness as risk factors for the progression of frailty: the English Longitudinal Study of Ageing. Age and Ageing.

[15] Andrew MK (2015). Frailty and Social Vulnerability. Interdiscip Top Gerontol Geriatr..

[16] Lee Y, Chon D, Kim J, Ki S, Yun J (2020). The predictive value of social frailty on adverse outcomes in older adults living in the community. Journal of the American Medical Directors Association.

[17] Henry JD, Coundouris SP, Mead J, Thompson B, Hubbard RE, Grainger SA (2023). Social frailty in late adulthood: social cognitive and psychological well-being correlates. J Gerontol B Psychol Sci Soc Sci.

[18] Ma L, Sun F, Tang Z (2018). Social Frailty is Associated with Physical Functioning, Cognition, and Depression, and Predicts Mortality. J Nutr Health Aging..

[19] Yamada M, Arai H (2018). Social frailty predicts incident disability and mortality among community-dwelling Japanese older adults. Journal of the American Directors Association.

[20] Zhang ZM, Cao S, Gao M, Ziao S, Xie X, Wu X (2023). The prevalence of social frailty among older adults: A systematic review and meta-analysis. JAMDA.

[21] Onyeaka H, Anumudu CK, Al-Sharify ZT, Egele-Godswill E, Mbaegbu P (2021). COVID-19 pandemic: A review of the global lockdown and its far-reaching effects. Sci Prog..

[22] Goto T, Kishimoto T, Fujiwara S, Shirayama Y, Ichikawa T (2024). Social frailty as a predictor of all-cause mortality and functional disability: a systematic review and meta-analysis. Nature Scientific Reports.

[23] Tsutsumimoto K, Doi T, Nakakubo S (2019). Impact of Social Frailty on Alzheimer’s Disease Onset: A 53-Month Longitudinal Cohort Study. J Alzheimers Dis..

[24] Santini ZI, Jose PE, York Cornwell E, Koyanagi A, Nielsen L, Hinrichsen C (2020). Social disconnectedness, perceived isolation, and symptoms of depression and anxiety among older Americans (NSHAP): a longitudinal mediation analysis. Lancet Public Health..

[25] Yoo M, Kim S, Kim BS (2019). Moderate hearing loss is related with social frailty in a community-dwelling older adults: The Korean Frailty and Aging Cohort Study (KFACS). Arch Gerontol Geriatr..

[26] Chen WY, Huang XH, Pu YH, Xu JX, Gao JL (2022). Relationship between social frailty and quality of life in community elderly. Geriatr Health Care..

[27] Hoang P, King JA, Moore S, Moore K, Reich K, Sidhu H, Tan CV, Whaley C, McMillan J (2022). Interventions associated with reduced loneliness and social isolation in older adults: A systematic review and meta-analysis. JAMA Network Open..

[28] Williams CYK, Townson AT, Kapur M, Ferreira AF, Nunn R, Galante V, Gentry S, Usher-Smith JA (2021). Interventions to reduce social isolation and loneliness during COVID-19 physical distancing measures: A rapid systematic review. PLoS ONE.

[29] Mah J, Rockwood K, Stevens S, Keefe J, Andrew MK (2022). Do interventions reducing social vulnerability improve health in community dwelling older adults? A systematic review. Clinical Interventions in Aging.

[30] Gardiner C, Geldenhuys G, Gott M (2018). Interventions to reduce social isolation and loneliness among older people: an integrative review. Health Soc Care Community..

[31] ZhaoJ, Liu YWJ, Tyrovolas S, Mutz J. Exploring the concept of psychological frailty in older adults: a systematic scoping review. JCE 2023;159:300-308. 10.1016/j.jclinepi.2023.05.005 37156339

[32] Xue QL (2011). The frailty syndrome: definition and natural history. Clin Geriatr Med..

[33] Lebrasseur A, Fortin-Bedard N, Lettre J, Raymond E, Bussieres EL, Lapierre N, Faieta J, Vincent C, Duchesne L, Ouellet MC, Gagnon E, Tourigny A, Lamontagne ME, Routhier F (2021). Impact of the COVID-19 pandemic on older adults: Rapid Review. JMIR Aging.

[34] World Health Organization (WHO). Health care considerations for older people during COVID-19 pandemic. WHO. Available at: https://www.euro.who.int/en/health-topics/health-emergencies/coronavirus-covid-19/technical-guidance/health-care-considerationsfor-older-people-during-covid-19-pandemic (2020).

[35] Losada-Baltar A, Jimenez-Gonzalo L, Gallego-Alberto L, Pedroso-Chaparro MDS, Fernandes-Pires J, Marquez-Gonzalez M. "We Are Staying at Home." Association of Self-perceptions of Aging, Personal and Family Resources, and Loneliness With Psychological Distress During the Lock-Down Period of COVID-19. J Gerontol B Psychol Sci Soc Sci. 2021;76(2):e10–e16. 10.1093/geronb/gbaa048 PMC7184373 32282920

[36] Peters MDJ, Marnie C, Tricco AC (2020). Updated methodological guidance for the conduct of scoping reviews. JBI Evid Synth.

[37] Tricco AC, Lillie E, Zarin W, O'Brien KK, Colquhoun H, Levac D, Moher D, Peters MDJ, Horsley T, Weeks L, Hempel S, Akl EA, Chang C, McGowan J, Stewart L, Hartling L, Aldcroft A, Wilson MG, Garritty C, Lewin S, Godfrey CM, Macdonald MT, Langlois EV, Soares-Weiser K, Moriarty J, Clifford T, Tunçalp Ö, Straus SE. PRISMA Extension for Scoping Reviews (PRISMA-ScR): Checklist and Explanation. Ann Intern Med. 2018. 10.7326/M18-0850 30178033

[38] McGowan J, Sampson M, Salzwedel DM, Cogo E, Foerster V, Lefebvre C. PRESS Peer Review of Electronic Search Strategies: 2015 guideline statement. J Clin Epidemiol. 2016 Jul; 75:406. http://www.sciencedirect.com/science/article/pii/S0895435616000585 10.1016/j.jclinepi.2016.01.021 27005575

[39] Kastner M, Wilczynski NL, Walker-Dilks C, McKibbon KA, Haynes RB (2006). for the Hedges Team. Age-specific Search Strategies for MEDLINE. JMIR.

[40] CADTH. Grey Matters: a practical tool for searching health-related grey literature | CADTH.ca. 2015; https://www.cadth.ca/resources/finding-evidence/grey-matters. Accessed February 2020.

[41] Stone PW (2002). Popping the (PICO) question in research and evidence-based practice. Appl Nurs Res..

[42] Pollock D, Peters MDJ, Khalil H, McInerney P, Alexander L, Tricco AC, Evans C, de Moraes EB, Godfrey CM, Pieper D, Saran A, Stern C, Munn Z (2023). Recommendations for the extraction, analysis, and presentation of results in scoping reviews. JBI Evid Synth.

[43] Pek K, Chew J, Lim JP, Yew S, Tan CN, Yeo A, Ding YY, Lim WS (2020). Social Frailty is Independently Associated with Mood, Nutrition, Physical Performance, and Physical Activity: Insights from a Theory-Guided Approach. International Journal of Environmental Research and Public Health.

[44] Lin, C.E., Wood, J.J. (2013). Self-management Interventions. In: Volkmar, F.R. (eds) Encyclopedia of Autism Spectrum Disorders. Springer, New York, NY.

[45] Elo S, Kyngäs H (2008). The qualitative content analysis process. J Adv Nursing..

[46] Nieboer AP, Hajema K, Murray Gramm J (2020). Relationships of self-management abilities to loneliness among older people: a cross-sectional study. BMC Geriatrics.

[47] Gramm JM, Twisk J, Nijboer AP (2014). Self-management abilities and frailty are important for healthy aging among community-dwelling older people; a cross-sectional study. BMJ Geriatrics.

[48] Steverink N, Lindenberg S, Slaets JP (2005). How to understand and improve older people’s self-management of well-being. Eur J Ageing..

[49] Bandura A (1982). Self-efficacy mechanism in human agency. Am Psychol..

[50] Cramm JM, Strating MMH, de Vreede PL, Steverink N, Nieboer AP (2012). Development and validation of a short version of the self-management ability scale (SMAS). Health Qual Life Outcomes..

[51] Lee JW, Nersesian PV, Suen JJ, Mensah Cudjoe TK, Gill J, Stanton SL, Hlasek MD (2023). Loneliness is associated with lower coping self-efficacy among older adults. J App Gerontol.

[52] Balki E, Hayes N, Holland C (2022). Effectiveness of Technology Interventions in Addressing Social Isolation, Connectedness, and Loneliness in Older Adults: Systematic Umbrella Review. JMIR Aging.

[53] Jones RB, Ashurst EJ, Atkey J, Duffy B (2015). Older People Going Online: Its Value and Before-After Evaluation of Volunteer Support. J Med Internet Res..

[54] Van Dijk J, Hacker K (2003). The Digital Divide as a Complex and Dynamic Phenomenon. The Information Society.

[55] Beaunoyer E, Dupere S, Guitton MJ (2020). COVID-19 and digital inequalities: reciprocal impacts and mitigation strategies. Computers in Human Behaviour.

[56] Khunti K, Platt L, Routen A, Abbasi K (2020). Covid-19 and ethnic minorities: an urgent agenda for overdue action. BMJ..

[57] Kirby T (2020). Evidence mounts on the disproportionate effect of COVID-19 on ethnic minorities. Lancet Respir Med..

[58] Steverink N, Lindenberg S (2006). Which social needs are important for subjective well-being? What happens to them with aging?. Psychol Aging.

[59] Mulligan K, Khiung S, Bloch G, Park G, Richter A, Stebbins L, Talat S (2023). Social Prescribing in Canada: A Tool for Integrating Health and Social Care for Underserved Communities. Healthcare Quarterly.

[60] Makizako H, Shimada H, Doi T, Tsutsumimoto K, Hotta R, Nakakubo S, Makino K, Lee S (2018). Social Frailty Leads to the Development of Physical Frailty among Physically Non-Frail Adults: A Four-Year Follow-Up Longitudinal Cohort Study. Int. J. Environ. Res. Public Heal..

[61] Gobbens RJ, Luijkx KG, Wijnen-Sponselee MT, Schols JM (2010). Towards an integral conceptual model of frailty. J Nutr Health Aging..

[62] Holt-Lunstad J, Smith TB, Layton JB (2010). Social relationships and mortality risk: A meta-analytic review. PLOS Medicine.

[63] Gilmour H. Social participation and the health and well-being of Canadian seniors. Health Reports, 2012;23, 4. 23356042

[64] Choi HK, Lee SH (2021). Trends and effectiveness of ICT interventions for the elderly to reduce loneliness: A systematic review. Healthcare.

[65] Ožić S, Vasiljev V, Ivković V, Bilajac L, Rukavina T (2020). Interventions aimed at loneliness and fall prevention reduce frailty in elderly urban population. Medicine (Baltimore)..

