# Supplementary material for: Interventions that have potential to help older adults living with social frailty: a systematic scoping review
Source: BMC Geriatr. 2024 Jun 15;24:521. doi: 10.1186/s12877-024-05096-w (PMC11179268; doi:10.1186/s12877-024-05096-w)
Supplement: Supplementary file 1 — Supplementary Material 1. [file 12877_2024_5096_MOESM1_ESM.docx]

**Supplementary file 1**

Database Search Strategies

**Database: Ovid MEDLINE: Epub Ahead of Print, In-Process & Other Non-Indexed Citations, Ovid MEDLINE® Daily and Ovid MEDLINE® <1946-Present>**
**Search Strategy**:
1 (social$ adj frail$).tw,kf.

2 Social Isolation/ [ social frailty terms ]

3 Loneliness/

4 Social Alienation/

5 (social$ adj isolat$).tw,kf.

6 (social$ adj alien$).tw,kf.

7 lonel$.tw,kf.

8 (social$ adj fragil$).tw,kf.

9 (social$ adj vulnerab$).tw,kf.

10 (social$ adj marginaliz$).tw,kf.

11 (social$ adj marginalis$).tw,kf.

12 (social$ adj connect$).tw,kf.

13 (social$ adj depriv$).tw,kf.

14 connectedness.tw,kf.

15 aloneness.tw,kf.

16 (emotional adj deprivation).tw,kf.

17 or/2-16

18 1 or 17

19 Aged/ [ geriatric search filter - validated, optimized ]

20 18 and 19

21 exp Animals/ not (exp Animals/ and Humans/)

22 20 not 21

23 limit 22 to yr="2000 -Current"

24 limit 23 to english language

**Database: Embase Classic+Embase <1947 to 2023 October 27>**
**Search Strategy:**
**1**  (social$ adj frail$).tw,kw.
2  Social Isolation/ [ social frailty terms ]
3  Loneliness/
4  Social Alienation/
5  Emotional Deprivation/
6  (social$ adj isolat$).tw,kw.
7  (social$ adj alien$).tw,kw.
8  lonel$.tw,kw.
9  (social$ adj fragil$).tw,kw.
10  (social$ adj vulnerab$).tw,kw.
11  (social$ adj marginaliz$).tw,kw.
12  (social$ adj marginalis$).tw,kw.
13  (social$ adj connect$).tw,kw.
14  (social adj deprivation).tw,kw.
15  connectedness.tw,kw.
16  aloneness.tw,kw.
17  (emotional adj deprivation).tw,kw.
18  or/2-17
19  1 or 18
20  (geriatrics or geriatric).tw,kw.
21  elder$.tw,kw.
22  senior$.tw,kw.
23  (old adj age).tw,kw.
24  (older adj adult?).tw,kw.
25  Aged/
26  or/20-25
27  19 and 26
28  Nonhuman/
29  27 not 28
30  limit 29 to yr="2000 -Current"
31   limit 30 to english language

**Database: EBSCOHost CINAHL<April 11, 2023>**

(MH "Social Isolation") OR (MH "Loneliness") OR (MH "Social Alienation") OR (TX "social* isolat*") OR (TX "social* frail*") OR (TX "social* alien*") OR (TX lonel*) OR (TX "social* fragil*") OR (TX "social* vulnerab*") OR (TX "social* marginaliz*")

AND

(MH "Aged+") OR (TX senescence) OR (TX "old age") OR (TX "old people") OR (TX "old person") OR (TX geriatric) OR (TX elder*) OR (TX senior*)

Limits: Year: 2020-Present; English; Peer reviewed

**Database: medRxiv <https://www.medrxiv.org>**

Advanced Search:
"social isolation" OR "socially isolated" OR "social frailty" OR "socially frail" OR "social alienation" OR "socially alienated" OR lonely OR loneliness OR "social fragility" OR "socially fragile" OR "social vulnerability" OR "socially vulnerable" OR "social marginalization" OR "socially marginalized"
Limits: Geriatric Medicine; Year: July 21, 2020-October 29, 2023

**Database: LitCOVID <https://www.ncbi.nlm.nih.gov/research/coronavirus>**

("social isolation" OR "socially isolated" OR "social frailty" OR "socially frail" OR "social alienation" OR "socially alienated" OR lonely OR loneliness OR "social fragility" OR "socially fragile" OR "social vulnerability" OR "socially vulnerable" OR "social marginalization" OR "socially marginalized")

AND

(aged OR senescence OR "old age" OR "old people" OR "old person" OR geriatric OR elder OR elderly OR senior OR seniors)
