# Supplementary material for: Interventions that have potential to help older adults living with social frailty: a systematic scoping review
Source: BMC Geriatr. 2024 Jun 15;24:521. doi: 10.1186/s12877-024-05096-w (PMC11179268; doi:10.1186/s12877-024-05096-w)
Supplement: Supplementary file 2 — Supplementary Material 2. [file 12877_2024_5096_MOESM2_ESM.docx]

**Supplementary file 2**

**Table 1a:** Study and intervention characteristics, including a summary of study results and outcomes for *Social resource-related interventions* (n=196)

| **Intervention Type** (number of studies)  *Definition* | **Intervention name**  *(Author, year)* | **Study design** | **Intervention and population description** | **Intervention details** | **Summary of study results and outcomes** |
| --- | --- | --- | --- | --- | --- |
| **Information Communication Technology (ICT) based interventions (n=41)**  *ICT is an umbrella term encompassing all the technologies and services involved in computing, data management, telecommunications provision, and the internet (Selwyn N, 2003). These technologies all deal with the transmission and reception of information of some kind in various forms (i.e., textual, audio, and/or visual), and includes internet-based social communication and device-mediated mediated communication (Chen YRR, 2016).* | **Social networking platforms and apps (n = 30)** | | | | |
|  | **A Personal Reminder Information and Social Management (PRISM) Technology-Based Application on a PC**  *(Czaja SJ, 2018 from Shah SG, 2021)* | Quantitative (RCT) | This intervention was for independent living older adults. This was based on technology applications on a PC to enhance social connectivity and decrease loneliness. Older adults were provided with a notebook, PC, keyboard, mouse, monitor, the PRISM software application (Personal Reminder Information and Social Management), printer, and internet. | M: Virtual (Computer) I: NR  N = 300 | Loneliness did not significantly improve. |
|  | **About My Age (Social Networking Site)** *(Ballantyne A, 2010 from Heins P, 2021)* | Qualitative | The intervention examined the internet social networking site, About My Age, which included one-on-one education sessions delivered by project team members for 3 months. | M: Virtual (Not specified)  I: One-to-one N = 6 | Benefits included reduced temporal loneliness (the extent varied by case) and an increased sense of connectivity to some extent. |
|  | **Accessible iPad-Based Communication App**  *(Barbosa Neves B, 2019)* | Mixed-and/or multi-method | An accessible iPad-based tablet communication app that supports older adults’ asynchronous communication with family and friends. | M: Virtual (Tablet)  I: Self-directed N = 12 | Qualitative: Some of the participants reported increased frequency of social interaction and perceived social interaction. There was a mixed effect on participant’s reported social connection, and relationship meaningfulness did not increase. The maintenance of social interaction was enabled, and there was a mixed effect on participant’s reported perceived well-being. |
|  | **Ambient Social Network System “Tlatoque”** *(Cornejo R, 2013 from Heins P, 2021)* | Qualitative | The intervention examined the use of an interactive display, Tlatoque, for 21 weeks. | M: Virtual (Not specified)  I: NR; N = Older adults = 2; Family members = 30 | Tlatoque supported social connectedness. |
|  | **Caring TV** *(Hemberg J, 2018 from Heins P, 2021)* | Qualitative | The intervention examined was real video communication called Caring TV. | M: Virtual (Not specified); I: NR  N = 7 | Participants identified that technology facilitated making new experiences and maintaining or developing social relationships. |
|  | **Communication Application** *(Shinokawa S, 2023)* | Mixed-and/or multi-method | Participants aged 65 and above were provided with smartphones for a six-month study from October 16, 2019, to April 15, 2020. The smartphones featured the "Kikoeru" app, facilitating step tracking, image sharing, and voice recording for participant interaction. Researchers conducted 30-minute sessions to guide participants on app usage, distribute instruction manuals, and encourage free use. Monthly in-person and online meetings were held, except for two months when all interactions shifted online due to the COVID-19 pandemic. | M: Virtual (Smartphone) I: Mixed (Group-based, self-directed) N = 9 | The results of this study suggest that older men may be able to use app to alleviate worsening loneliness. |
|  | **Content Creation Management System** *(Morganti L, 2016 from Rivera-Torres S, 2021)* | Quantitative (RCT) | The intervention involved utilizing a desktop-based application, NoBits, to facilitate content creation and management for older adults with the goal of reducing loneliness. The NoBits application enabled participants to capture and upload their memories, including local history represented through photos, newspapers, and postcards. | M: Virtual (Computer) I: NR  N = 34 | Decrease in emotional loneliness and an improvement in social loneliness; however, values for general loneliness did not decrease in either group. Also, no difference was found in the feeling of loneliness between control and experimental condition. |
|  | **Facebook** *(Myhre JW, 2017 from Heins P, 2021)* | Quantitative (Clinical controlled trial) | The intervention examined using Facebook which included 2-hour training sessions for 1 week, use of Facebook (1×/day) and writing posts (1×/week) for 7 weeks. | M: Virtual (Not specified)  I: NR  N = 60 | There were no significant differences in social support, loneliness, and social integration (pretest vs. post-test) in any of the groups. |
|  | **Facilitator-led Remote Interactive Intervention for Loneliness, Quality of Life, and Social Support** *(Liu CW, 2023)* | Quantitative (RCT) | Participants (aged ≥ 65 years) received a 12-week bidirectional remote interaction intervention (participants could interact with instructors) involving daily (Monday-Friday) one-hour interactive and structured facilitator-led online sessions covering various topics. Activities included structured courses (static) and dynamic exercises. Participants could interact with the facilitator and other participants. Additional interactions occurred through news broadcasts, YouTube videos, music broadcasts, and text messaging among participants. | M: Virtual (Smartphone) I: Group-based N = 100 | There were no significant between group differences in loneliness, quality of life or social support. |
|  | **Fik@ room** *(Johansson-Pajala RM, 2023)* | Mixed-and/or multi-method | Older adults (65 years or older) facing social isolation and loneliness participated in a 12-week study using the Fik@ room, a web platform designed for social interaction. The platform, developed through a user-centered approach, aimed to provide a secure and user-friendly environment. Participants, provided with tablets, logged in at specified times three days a week and kept diaries. he Fik@ room featured digital coffee tables for group interactions, various communication modes, and a bulletin board for messages and meetings. | M: Virtual (tablet) I: Group-based N = 28 | The findings suggest that the Fik@ room is a feasible ICT tool for older people to develop new friendships, increase one’s social network, and reduce the experience of loneliness. |
|  | **Health Enhancement Support System (CHESS)** *(Leszko M, 2020 from Mao W, 2023)* | Mixed-and/or multi-method | This was a social forum for caregivers to maintain social connection for participants 55-64 years. | M: Virtual (Computer) I: Group-based  N = 48 | There was significant decrease in loneliness amongst caregivers. |
|  | **In Touch Social Contact System** *(Judges RA, 2017 from Heins P, 2021)* | Qualitative | The intervention examined was the digital communication tool, In Touch, a social contact system. Older adults were paired with a volunteer for 3 months in using the system. | M: Virtual (Not specified)  I: NR  N = 10 | Benefits included improved communication and positive changes in relationships. |
|  | **Internet Social Networking** *(Ballantyne A, 2010 from Noone C, 2022)* | Qualitative | The intervention was a social networking intervention with one-to-one tutoring on how to use the site for older adults. | M: Virtual (Computer) I: One-to-one  N = 4 | The intervention provided social connectivity with other members and the outside world. |
|  | **iPad-Based Communication App** *(Barbosa Neves B, 2019 from Choi HK, 2021)* | Quantitative (Cross-sectional) | The intervention for elderly participants aged 80 and above in a retirement home promotes social connectedness through iPads. The app facilitates sharing of photos, videos, and messages, featuring a user-friendly interface with large non-textual touch icons, enabling communication through swiping, or tapping without typing. | M: Virtual (iPad) I: Self-directed N = 12 | There were no significant changes in loneliness and social support. |
|  | **Media Parcels** *(Zaine I, 2019 from Heins P, 2021)* | Mixed-and/or multi-method | The intervention examined a human-facilitated social networking system, Media Parcels, with facilitation by a psychologist for two weeks with family members or friends. | M: Virtual (Not specified)  I: NR  N = 2 | Participants reported contacting each other more often and feeling closer to each other. |
|  | **Media Parcels Social Networking System**  *(Zaine I, 2019)* | Qualitative | Media Parcels is a human-facilitated social networking system that uses the metaphor of delivering parcels in the mail. The system supports a facilitator in designing time-based dialogue to request parcels from participants that bring out their memories and feelings, which are then wrapped in annotations and delivered to a target person. | M: Virtual (Computer) I: Group-based  N = NR | The intervention promoted communication and deepened social relations between participants of the same and different generations. |
|  | **Project VITAL At Home** *(Nguyen LT, 2022 from Mao W, 2023)* | Quantitative (Cross-sectional) | This intervention was for caregivers 68 years on average and encompassed 7-month use of iN2L tablets to engage with and socially connect with friends and family which is part of the Alzheimer’s Association program. | M: Virtual (Tablet) I: Group-based  N = 124 | There were no changes for loneliness. |
|  | **Remote Sharing with Family Members**  *(Noguchi T, 2022)* | Quantitative (Quasi-experimental) | Japanese adults aged ≥ 50 used a service called Mago-Channel that allowed them to receive photos/videos from their families for 3 months on a device set up on their home TVs. Changes in psychological health and social relationships with families living together and not together were evaluated. | M: Virtual (Television) I: Self-directed N = 115 | While loneliness and frequency of talking with friends did not change significantly, satisfaction with the relationship of families living together, talking time with families living together and frequency of talking with families not living together did. |
|  | **Remotely Delivered Technology** *(Chen AT, 2021 from DesChâtelets JR, 2023)* | Qualitative | Community-dwelling adults were delivered an intervention that focuses on using technology to promote social connectedness with family, online services for various activities, and teaching older adults’ tasks they can't do anymore (such as gardening). (Based on online discussions). | M: Virtual (Not specified)  I: NR  N = NR | Information and technology use kept participants connected. |
|  | **Senior App Suite** *(Goumopoulos C, 2017 from Heins P, 2021)* | Quantitative (Cross-sectional) | The intervention examined using the mobile application Senior App Suite for enhancing the social inclusion and well-being of older adults across 8 weeks. | M: Virtual (Not specified)  I: NR  N = 22 | “Senior App Suite” may reduce loneliness moderately (p = 0.034) |
|  | **Services To Communicate** *(Zaine I, 2019 from Rivera-Torres S, 2021)* | Mixed-and/or multi-method | The intervention focused on utilizing smartphones with messaging services encompassing video, audio, photos, and text capabilities. The primary objective was to increase social communication among individuals aged 60 and above who reside in their own homes. The study introduced a web-based application where a human facilitator initiated time-based requests, collected media messages, and distributed them to a target person along with text commentary, contributing to the deepening of existing relationships. | M: Virtual (Smartphone) I: Group-based N = 6 | Qualitative: Both case studies revealed positive social effects for both deepening and developing of relationships. All participants reported feeling closer to each other and contacting each other more than usual. |
|  | **Social Internet-Based Activity (SIBA)** *(Larsson E, 2016 from Shah SG, 2021)* | Quantitative (RCT) | This intervention was for older adults living in regular housing without any home care services. This was based on social internet-based activity (SIBA) from social websites. The aim was to decrease loneliness in older adults. | M: Virtual (Computer) I: NR  N = 30 | Loneliness did not significantly improve. |
|  | **Social Networking** *(Jansen-Kosterink SM, 2020 from Rivera-Torres S, 2021)* | Quantitative (Quasi-experimental) | This intervention was based on having a social network and being accessible to a smartphone, tablet, and desktop to increase social participation for those 60+ years living at home. | M: Virtual (Computer, smartphone, tablet); I: NR  N = 41 | There were no significant changes in loneliness; however, there were positive changes for quality of life. |
|  | **Social Networking at Home** *(Goumopoulos C, 2017 from Rivera-Torres S, 2021 )* | Mixed-and/or multi-method | The intervention aimed to create a social network for participants aged 60 and above through Facebook, providing them with a desktop and tablet at home to enhance social participation. | M: Virtual (Computer, tablet) I: NR  N = 20 | There were moderate improvements in loneliness p=0.034. |
|  | **SONIA Communication Platform** *(Biniok P, 2015 from Heins P, 2021)* | Qualitative | The intervention examined using a tablet with the SONIA communication platform for 6 months. | M: Virtual (Not specified)  I: NR  N = 30 | Participants with few social contacts increased their social participation. Whereas some participants (mostly with high technological skills) experienced only a slight change in social participation. |
|  | **The Personal Reminder Information and Social Management (PRISM) System** *(Czaja SJ, 2018 from Choi HK, 2021)* | Quantitative (RCT) | PRISM, a comprehensive support system for elderly participants aged 65 and above, living independently, offers training across various technology modules such as internet access, photos, email, games, etc. The system is designed to reduce loneliness through robust training and instructional support. PRISM includes modules like "Internet access," "Resource guide," "Classroom," "Calendar," "Photo," "E-mail," "Game," and "Online help." | M: Virtual (Smartphone) I: Self-directed N = 244 | There was a significant decrease in loneliness at six months (p<0.01), social isolation (p<0.01), and an increase in social support (P<0.01). |
|  | **The Personal Reminder Information and Social Management (PRISM) System** *(Czaja SJ, 2018 from Heins P, 2021)* | Quantitative (Clinical controlled trial) | The intervention examined was a comparing the use of a notebook with printed content similar to the personal reminder information and social management system (PRISM) for a 12-month period. | M: Virtual (Not specified)  I: NR  N = 300 | There was a significant decrease in loneliness at six months (p < 0.04) and an increase in perceived social support (p < 0.004) of the PRISM group vs. comparison group; but this was not maintained at 12 mos. |
|  | **Using Communication Technology** *(Myhre JW, 2017 from Casanova G, 2021)* | Quantitative (RCT) | This intervention was based on social interactions and social media for those 75-86 years. This was a total of 8 weeks with 6 hours of training. | M: Virtual (Not specified)  I: NR  N = 43 | No significant change for loneliness. |
|  | **Virtual Classroom to Message** *(Czaja SJ, 2018 from Rivera-Torres S, 2021)* | Quantitative (RCT) | The intervention involved creating virtual spaces or classrooms on desktops, equipped with messaging capabilities and games, to enhance social communication and participation for individuals aged 65 and older at home. These virtual spaces served as online environments where older adults could connect, discuss shared interests, and engage in voluntary activities, potentially including teaching other older adults. The emphasis was on leveraging web-based activities, such as gaming, to foster social interaction and involvement in this age group. | M: Virtual (Computer) I: Group-based N = 224 | There was a significant decrease in loneliness and social isolation and an increase in social support and well-being. |
|  | **Web-based Telehealth System with Facebook and Game Like Features**  *(Dhillon JS, 2011 from Chen YRR, 2016)* | Qualitative | An ICT, web-based telehealth system that had Facebook-like features and networked games. It was accessed through a computer and tested with participants aged 60 to 87 years. | M: Virtual (Computer) I: Self-directed N = NR | ICT use alleviated loneliness, fostered social interaction and social support. |
|  | **Device-mediated communication (n = 9)** | | | | |
|  | **Digital Information Technology-Based Interventions** *(Czaja SJ, 2018; Hind D, 2014; Jarvis MA, 2019; Larsson E, 2016; Morton TA, 2018; Myhre JW, 2017; Tsai HH, 2020; Tsai HH, 2010 from Lestari WA, 2023)* | Quantitative (RCT, pre-post) | This systematic review examined the effect of digital information technology-based interventions in reducing loneliness in the elderly. | M: Virtual (Computer, telephone) I: Mixed (Group-based, one-to-one) N = NR | All studies report outcomes of loneliness. The systematic review concluded that the evidence for the effectiveness of technology-based interventions for reducing loneliness in the elderly is uncertain. |
|  | **iPad To Socialize** *(Barbosa Neves B, 2019 from Rivera-Torres S, 2021)* | Mixed-and/or multi-method | The intervention involved providing older adults in a retirement home with iPads equipped with messaging services such as video, audio, and photo sharing. The purpose of this initiative was to address social isolation and loneliness by increasing social communication among individuals aged 70 and above. The technology facilitated various forms of communication, including web-based chat, videoconferencing, group chat, and email. | M: Virtual (iPad) I: Group-based N = 12 | Quantitative: Although the app increased sense of social interactions (communication frequency and type) with family and friends for 10 participants, only four of 12 participants reported high perceived social connectedness at post deployment. No significant changes in both social support and loneliness. |
|  | **Messaging Chat** *(Garattini C, 2012 from Rivera-Torres S, 2021)* | Mixed-and/or multi-method | The intervention involved the creation of a "building bridges device," utilizing a touch screen computer connected to a custom-made stand, along with a phone handset. This device aimed to enhance social communication for participants aged 65 and older at home by incorporating messaging chat and text functionalities, along with a phone handset equipped with functioning speakers. | M: Virtual (Computer, smartphone) I: NR  N = 19 | Quantitative: There was an increase in social connections and new interactions. |
|  | **Phone and Video Conferencing Service** *(Airola E, 2020 from Heins P, 2021)* | Qualitative | The intervention examined phone and videoconferencing calls from a volunteer once a week. | M: Virtual (Telephone) I: One-to-one N = 5 | Benefits included that the service facilitated networks and reduced loneliness. |
|  | **Smart Technology Interventions** *(Dew MA, 2004; Hill W, 2006; Weinert C, 2011;  Weinert C, 2008; Barrera M Jr, 2002; Billipp SH, 2001; Bond GE, 2010; Chiu T, 2009; Fokkema T, 2007; Gustafson DH, 2005; Kahlbaugh PE, 2011; Lieberman MA, 2005; Mahoney DF, 2003; Pierce LL, 2009; Samoocha D, 2011; Slegers K, 2008; Van Straten A, 2008; Torp S, 2008 from Morris ME, 2014)* | Quantitative (RCT, cohort study) | This systematic review examined the effectiveness of smart technologies to enhance social connectedness in older adults who live at home. | M: Virtual (Computer) I: Mixed (Group-based, self-directed, one-to-one) N = NR | Statistically significant improvements in social support were seen in 6 studies reporting use of smart technology over periods of less than one year. Three studies found positive results and two had inconclusive findings on levels of loneliness. Overall, smart technologies that included interactive, online programs, and discussion forums had positive effects on quality of life and health-related quality of life. |
|  | **Technology for Long-Distance Interactions** *(Ballantyne A, 2010; Fokkema T, 2007; Blažun H, 2012; Chiu CJ, 2019; Cotten SR, 2013; Czaja SJ, 2018, Gutierrez FJ, 2017; Jarvis MA, 2019; Jarvis MA, 2019; Larsson E, 2016; Machesney D, 2014; Széman Z, 2014 White H, 2002; Cattan M, 2011; Dodge HH, 2015; Garattini C, 2012; Baez M, 2017; Banbury A, 2017; Barbosa Neves B, 2019; Isaacson M, 2019; Morton TA, 2018; Myhre JW, 2017; Neves BB, 2018; Pauly T, 2019; Tomasino KN, 2017 from Ibarra F, 2020)* | Mixed-and/or multi-method | The intervention selection criteria for the systematic review were centered around older adults who utilized technology, including the internet, computers, tablets, mobiles, or smartphones, for purposes related to social communication, social interaction, social networking, or social participation. | M: Virtual (Computer, smartphone, tablet) I: NR  N = NR | Qualitative studies: Overall, 4/6 qualitative studies had outcomes reporting decreased loneliness however it is important to note that some of these studies reported the results as being "perceptions" not from a "standardized measurement tool."  Quantitative studies: n=7 quantitative studies reported no significant differences in loneliness and network size, however, some studies reported decreased loneliness (n=9) and increased network size (n=2). |
|  | **Various Digital Tools to Support Social Engagement** *(Choi M, 2012; Chen YR, 2016; Morris ME, 2014; Forsman AK, 2017 from Larsson E, 2020)* | Mixed-and/or multi-method | These various digital tools include internet and computer use and training, smartphone use, chats, games, email, and various application usage for those 60+ years old to decrease loneliness and increase social engagement and social participation*.  **Some intervention types included in this review may also fall into other domains; however, this classification represents the majority.* | M: Virtual (Not specified)  I: NR  N = NR | There was a significant decrease in loneliness for those who had internet and computer training and those who used chats, email, games, iPads, and smartphones. Internet use and phone, computer, or internet support intervention had a significant effect on social isolation. There was an increase in social interaction and social support for internet usage and technology interactive website interventions. There was a significant effect on feelings of belonging for social media, video calls, and mobile usage. |
|  | **Video Calling** *(Noon C, 1996 from Astasio-Picado Á, 2022)* | Quantitative (Systematic review) | Video calling information and communication interventions. | M: Virtual (Not Specified) I: NR  N = 201 | Overall, there was minimal effect on loneliness and quality of life for the video-calling interventions. |
|  | **Video Chat** *(Kleinberger R, 2019 from Rivera-Torres S, 2021)* | Mixed-and/or multi-method | The intervention utilized an Android device with a processor transformed into a Raspberry like board, aiming to enhance social communication for participants aged 70 and above in a senior center. It also included the creative technological solution, of a memory music box that triggers a photo slideshow, notifying grandchildren via email for a potential video call when opened. | M: Virtual (Smartphone) I: NR  N = 10 | Qualitative: Participants perceived potential for improved connectedness. |
|  | **Combination of social networking and device-mediated communication (n = 2)** | | | | |
|  | **General ICT**  *(Mellor D, 2008 from Chen YRR, 2016)* | Qualitative | General ICT use: Internet, mobile/smartphones, iPads, social networking sites, and audio/video chat apps. | M: Virtual (Smartphone, tablet) I: Self-directed N = NR | Elderly people’s use of computer and Internet at home increased their social connectedness at the 3-month stage of intervention but not at the 6-month or 9-month stage. ICT was an effective means for older adults to remain connected with others, and it had a positive influence on life satisfaction. |
|  | **Various Communication Technology Interventions** *(Baker S, 2018; Brimelow RE, 2017; Casanova G, 2021; Cattan M, 2005; Chen E, 2022; Chen YR, 2016; Choi HK, 2021; Choi M, 2012; Cohen-Mansfield J, 2015; Dickens AP, 2011; Dickens AP, 2011; Franck L, 2016; Gardiner C, 2018; Gasteiger N, 2021; Gorenko JA, 2021; Hagan R, 2014; Heins P, 2021; Ibarra, 2020; Ibrahim AF, 2022; Isabet B, 2021; Khosravi P, 2016; Khosravi P, 2016; Li J, 2018; Masi CM, 2011; Morris ME, 2014; Cochrane Public Health Group, 1996; O’Rourke HM, 2018; Poscia A, 2018; Shah SG, 2021 from Döring N, 2022)* | Mixed-and/or multi-method | This scoping review provides various studies on communication technology interventions to reduce loneliness and/or social isolation in those 55+ years old*. **Some intervention types included in this review may also fall into other domains; however, this classification represents the majority.* | M: Virtual (Not specified)  I: NR  N = >71000 | Quantitative: 55% of outcome measures on loneliness had positive effects (a decrease in loneliness) and 44% of outcomes on social isolation had positive effects (a decrease in social isolation). |
| **Intergenerational interventions (n=33)**  *Intergenerational programs and interventions connect younger and older generations to foster positive experiences, learning, and appropriate socialization for both young and old (Mosor E, 2019).* | **To promote or build social connections and engagement (intergenerational connectedness and exchange) (n = 12)** | | | | |
|  | **Intergenerational Nursing Communication Project** *(Kirk L, 2023)* | Mixed-and/or multi-method | To counteract social isolation in older adults, a semester-long clinical project was developed for sophomore Bachelor of Nursing Science (BSN) students in collaboration with a large, independent, not-for-profit senior living organization. Implemented in Fall 2020, and replicated in subsequent years, the project aimed to foster intergenerational connections. Students were paired with older adult mentors, initiating contact every 2 weeks over the 15-week semester, guided by a collaboratively developed conversation guide (Table 1). Framed as a mentor–mentee relationship, the initiative provided older adult mentors with the opportunity to reflect on and share their wisdom and life experiences with students, fostering intergenerational learning. | M: NR I: Mixed (Group-based, one-to-one) N = 124 | Quantitative: 88% indicated that the program increased their sense of social connection. |
|  | **Big and Mini: Intergenerational Program for Social Connection** *(Xu L, 2022)* | Mixed-and/or multi-method | This program's aim was to link younger people (18+) with older people who are 50+ years to promote social connectivity through weekly phone call. To promote activity there is a conversation starter that acts as a guide for the older population and one for the younger population. | M: Virtual (Telephone) I: One-to-one N = Bigs: 63 participants Minis: 53 participants | Qualitative: Older participants revealed that the program helped them with loneliness and build relationships.  Quantitative: There were high levels of intergenerational closeness. |
|  | **Friendship and Engaging with College Students** *(Sehrawat S, 2017 from Ibrahim AF, 2022)* | Qualitative | The intervention aimed to enhance social engagement and intergenerational connectedness among individuals aged 70 and above through pairs of college students and older adults. Over a span of six weeks, four pairs of individuals met weekly in public places. The interaction involved storytelling, discussions, and reflections, fostering meaningful connections between the older adults and college students. Additionally, each pair collaborated on creating digital storytelling content, providing a platform for shared experiences and narratives. | M: In-person I: One-to-one N = 4 | Participants had increased social connectedness and network size through sharing their digital story with friends, family, and project participants. |
|  | **Intergenerational Connections** *(Peterat L, 2006 from Peters R, 2021)* | Qualitative | Intergenerational intervention involving discussions between older adults and seventh-grade students on topics like land, food, society, etc. The goal was to create a space fostering sustained intergenerational conversations on land, food, community, society, and environmental issues with the hope of re-establishing connections. | M: NR  I: NR  N = 7 | Older volunteers experienced rewards beyond those of grand parenting relationships. Cross-generational connection took place. |
|  | **Intergenerational Engagement Intervention** *(Barbosa MR, 2021 from Krzeczkowska A, 2021)* | Mixed-and/or multi-method | This intervention for those 72-90 years was based on intergenerational relationships assessing loneliness, and self-esteem in older adults. This was for 2-hour sessions per month for 1 year. | M: NR  I: NR  N = 12 | There were large significant effects of loneliness. Qualitative findings found a positive impact on well-being. |
|  | **Intergenerational Engagement Intervention** *(Kamei T, 2011 from Krzeczkowska A, 2021)* | Mixed-and/or multi-method | This intervention for those 72.1 years on average was based on evaluating the progression of intergenerational interactions between older adults and children. This was for 3 hours per week in 2.5-hour sessions for 22 sessions. | M: NR  I: NR  N = 22 | Qualitative data revealed that IE encouraged expansion of social interactions outside the programme. |
|  | **Intergenerational Forum Program** *(Lee OE, 2022)* | Quantitative (Quasi-experimental) | Youth-led tutorials to learn about Information Communication Technology (ICT) were offered to older adults in a 12-week program. The course focused on encouraging intergenerational exchange and mutual aid between college students and older adults, and the program assessed changes in social capital and social isolation for participants. | M: In-person I: Group-based N = 104 | Social isolation and social capital did not significantly change after the intervention. |
|  | **Intergenerational Friendly Telephone Visit Program**  *(Kumar AB, 2023)* | Qualitative | This intervention targeted community-dwelling individuals aged 65 and older. The Harvard Concordium program, initiated in 2019 and adapted to a telephone format during the Covid-19 pandemic, paired student volunteers with older adults experiencing loneliness. The eight-week program, consisting of weekly 30-minute calls, aimed to build rapport and connections. Student volunteers underwent training and were matched based on common interests, engaging in conversational and social visits. | M: Virtual (Telephone) I: One-to-one N = 10 | Most responses indicated that the program fostered valuable intergenerational connections and participants valued the regular opportunities for social interaction and interpersonal connection. They described an increasing ease of conversation. |
|  | **Intergenerational Mentor-Up** *(Lee OE, 2019 from Krzeczkowska A, 2021)* | Mixed-and/or multi-method | This intervention was for those 73.82 years on average. This was 6 sessions, 1 hour each and was based on intergenerational engagement for older adults and to help with social isolation. | M: NR  I: NR  N = 55 | There was a significant decrease in loneliness and social isolation (p<0.001). There was no significant change in social support (p=0.21). |
|  | **Social Inclusion Intervention** (Alcock CL, 2011 from Krzeczkowska A, 2021) | Qualitative | This intervention was to promote social inclusion and well-being in those 65-80 years old. This was in 36 sessions, which were 90 minutes long for 7 months. | M: NR  I: NR  N = 13 | There was a greater sense of community and companionship. |
|  | **Socrates Cafes**  *(Dinkins CS, 2019)* | Qualitative | Socrates Cafés offer a distinctive social experience by encouraging communal exploration of questions related to values and meaning. Older adults and college students were both involved in the intervention. | M: In-person I: Group-based N = NR | Relationships and connection were formed across the two generations, because of self-disclose of experiences and values. |
|  | **YOLG Program**  *(Sun Q, 2019 from Krzeczkowska A, 2021)* | Quantitative (Quasi-experimental) | This was for those 72.54 years on average. This involved intergenerational interaction for 4 interactive weeks. | M: NR  I: NR  N = 150 | Significant group x time interaction effects were found for initiating conversation with young participants (p < .01; d = .46; pretest = .24, post-test = .40). |
|  | **To share experiences and memories including reminiscence (n = 7)** | | | | |
|  | **Aging Is Very Personal Program (AIVP)** *(Beausoleil K, 2022)* | Mixed-and/or multi-method | The Aging Is Very Personal (AIVP) program, established in 2015 at a large public university, connects third-year undergraduate nursing students with older adults in nearby assisted living communities. Students engage in four face-to-face conversations guided by weekly modules exploring the aging experience. The study evaluates the program's effect on social connectedness in challenging times. | M: Virtual (Computer) I: One-to-one N = 51 | Qualitative: The intervention promoted social connectedness, reduced social isolation, and created meaningful intergenerational relationships. |
|  | **DOROT's Summer Teen Internship Program**  *(Parkinson D, 2019)* | Qualitative | The Program brings together high school students and older adults to learn from each other through a year-long program that includes visits to clients' homes and intensive group experiences. | M: In-person I: One-to-one N = 48 | Most of the older adult participants felt more socially connected through participating in activities with teen interns and enjoyed these new experiences. |
|  | **Intergenerational Engagement Intervention** *(Mahoney N, 2020 from Krzeczkowska A, 2021)* | Mixed-and/or multi-method | This intervention was to examine the experiences of older male mentors involved with younger males with an intellectual disability. The aim was to increase generativity. This was for 1 session per week for 3-5 hours per week for 6 months for those 50-81 years. | M: NR  I: NR  N = 15 | There were no significant effects for social functioning. |
|  | **Intergenerational Reminiscence Intervention** *(Gaggioli A, 2014 from Hutchinson TD, 2022)* | Mixed-and/or multi-method | This intervention was based on intergenerational reminiscence groups for community-dwelling participants 67 years on average whereby the sessions consisted of a psychologist (facilitator) and 2 older adults and 6-8 younger students once a week for, 2 hours over the course of 3 weeks. The older adults would share photographs and other things during their sessions. The aim was to aim in loneliness and quality of life in older adults. | M: In-person I: Group-based N = 32 | Qualitative: Overall, there was a decreased sense of loneliness and increased quality of life. |
|  | **Intergenerational Reminiscence Intervention** *(Gaggioli A, 2014 from Krzeczkowska A, 2021)* | Quantitative (Cross-sectional) | This was for those 67.53 years on average. This was for 3 times per week for 2 hours for a total of 3 weeks. This encompassed seeing the effects of loneliness on older adults for intergenerational reminiscence. | M: NR  I: NR  N = 32 | There were significant increases in closeness to children, communal involvement, and self-confidence (p<0.001). |
|  | **Intergenerational Reminiscence Therapy**  *(Gaggioli A, 2014 from Poscia A, 2018)* | Quantitative (Cross-sectional) | Elderly participants were assigned to small groups, including one peer and 6-8 children, to form 16 mixed senior-student groups. Each group met once a week for two hours over three weeks. A psychologist led groups to encourage elderly participants to share their memories and to promote interaction with the pupils. | M: In-person I: Group-based N = 32 | Older adult participants reported significant decrease in emotional loneliness and improvement in quality of life. There was also a non-significant decreased feelings of loneliness and social loneliness. |
|  | **Promoting Interaction Between Older Adults and Children** *(Barbosa MR, 2021 from Carvalho MI, 2022)* | Quantitative | This program was based on older adults sharing experiences with the "institutional community" or younger generations (intergenerational connection). | M: In-person I: NR  N = NR | There was a significant decrease in loneliness as older adults were able to share life experiences and had the ability to create a bond. |
|  | **Community-based intergenerational programs or services (n = 6)** | | | | |
|  | **Art-Technology Intergenerational Community (ATIC) Program** *(Seo JH, 2021)* | Mixed-and/or multi-method | The article explores the impact of the Art-Technology Intergenerational Community (ATIC) program on the health, well-being, and social connectedness of individual older adults. The ATIC program comprises four sessions over four weeks, each lasting 1 hour. Participants, aged 60 and above and without diagnosed age-related mental illnesses, engaged in art and technology creations alongside undergraduate volunteers. Weekly projects included light-up cards, pop-up cards with light, interactive light painting, and interactive soft circuit ornaments, incorporating basic art forms like painting, drawing, paper folding, and sewing. Participants collaborated closely with undergraduate volunteers, sharing hobbies and personal interests. | M: In-person I: Group-based N = 18 | Qualitative: Overall, the ATIC program was effective in improving subjective health and social connectedness in older adults. |
|  | **Good Neighbor Program** *(Sandu S, 2021)* | Quantitative (Observational) | The Good Neighbor Program engaged individuals aged 60 and above with student volunteers (aged 18 or older) making regular phone calls over a year using standardized scripts. Community service agencies paired student volunteers with older adults, and upon consent during the first call, trained volunteers continued using the program's standardized phone scripts. | M: Virtual (Telephone) I: One-to-one N = 261 | Loneliness did not significantly change. |
|  | **Intergenerational Community-Based Program** *(Young TL, 2013 from Krzeczkowska A, 2021)* | Quantitative (Cross-sectional) | This program was based on identify older adult benefits and concerns in an intergenerational community-based intervention. This was for 50–89-year-olds who partook in the 5 yearlong initiatives. | M: NR  I: NR  N = 197 | There were significant effects on social life (p<0.001). |
|  | **Intergenerational Program: Arts-Based**  *(Cohen-Mansfield J, 2022)* | Mixed-and/or multi-method | This study found community-based intergenerational programs in Tel Aviv that were based in a community center or the participant's home. The aim was to evaluate 3 types of intergenerational programs (arts-based: theatre and dance; providing older participants with art to use their knowledge). The older adults included in the study were for participants 77 years on average. | M: In-person I: Group-based  N = 84 | Quantitative: There was a significant difference across all 3 intervention groups for decreased loneliness (p=0.01), where the highest rating was in the assistance group.  Qualitative: The art-based program revealed there being positive outcomes for meeting other generations and new friends. |
|  | **Intergenerational Program: Assistance-Based**  *(Cohen-Mansfield J, 2022)* | Mixed-and/or multi-method | This study found community-based intergenerational programs in Tel Aviv that were based in a community center or the participant's home. The aim was to evaluate 3 types of intergenerational programs (assistance-based: support with daily activity; providing companionship to homebound individuals). The older adults included in the study were for participants 77 years on average. | M: In-person I: One-to-one N = 84 | Quantitative: There was a significant difference across all 3 intervention groups for decreased loneliness (p=0.01), where the highest rating was in the assistance group.  Qualitative: The assistant-based program revealed there being positive outcomes for building relationships, specifically making new friendships. |
|  | **Intergenerational Program: Learning-Based** *(Cohen-Mansfield J, 2022)* | Mixed-and/or multi-method | This study found community-based intergenerational programs in Tel Aviv that were based in a community center or the participant's home. The aim was to evaluate 3 types of intergenerational programs (learning-based: acquiring knowledge and skill; improving mentoring and teaching to participants). The older adults included in the study were for participants 77 years on average. | M: In-person I: Mixed (Group-based, one-to-one) N = 84 | Quantitative: There was a significant difference across all 3 intervention groups for decreased loneliness (p=0.01), where the highest rating was in the assistance group.  Qualitative: The learning-based program revealed there being positive outcomes for meeting other generations. |
|  | **General Intergenerational interventions (n = 4)** | | | | |
|  | **Assessing Health and Social Capital** *(de Souza EM, 2007 from Krzeczkowska A, 2021)* | Quantitative (RCT) | This intergenerational intervention was for those 60+ years to assess social capital and self-rated health for 2 hours, once per week for four months. | M: NR  I: NR  N = 266 | Mixed findings for social functioning and non-significant effects for health status. |
|  | **Health Professional Mentoring** *(Halpin SN, 2017 from Krzeczkowska A, 2021)* | Mixed-and/or multi-method | This was for those 64-99 years. This was 1 session per month for 11 months. The intervention was seeing the impact of health professional students on older adults’ emotion, mental, and physical health. | M: NR  I: NR  N = 147 | Quantitative: There were significant results for social functioning (p=0.004). Qualitative results showed there was more relationship meaningfulness (having purposeful contact with younger generation). |
|  | **Intergenerational Empowerment Intervention** *(Gamliel T, 2014 from Krzeczkowska A, 2021)* | Mixed-and/or multi-method | This intervention was for those 66-77 years. This was for once per week for 2 hours for 8 months. The aim was to examine empowerment in the generational groups and social relationships between the groups. | M: NR  I: NR  N = 29 | There were significant decreases in loneliness (p<0.05). |
|  | **Older Adult Tutors** *(Carstensen L, 1982 from Krzeczkowska A, 2021)* | Mixed-and/or multi-method | This intergenerational intervention was examining older adult children tutors' morale. This was for 4 days per week for 15 minutes for 2 months. | M: NR  I: NR  N = 23 | 70% of participants reported feeling more in touch with their community. |
|  | **To promote socialization through activities or skills training (n = 4)** | | | | |
|  | **Intergenerational Mentor-Up (IMU)**  *(Lee OEK, 2019)* | Mixed-and/or multi-method | Intergenerational Mentor-Up (IMU) is an intervention that engages college students in tutoring older adults on how to use technology to engage in meaningful activities. | M: In-person I: Group-based N = 55 | Quantitative: Feelings of loneliness and social isolation were significantly decreased. There was also a non-significant reduction in perceived lack of social support among participants. |
|  | **Nintendo Wii with a Partner**  *(Kahlbaugh PE, 2011 from Hagan R, 2014)* | Quantitative (RCT) | Over 12-week period research assistants visited older adults and either watched television or played Wii with them. The research assistants were encouraged to be socially responsive to their partners. | M: In-person I: One-to-one N = 35 | Older adults had a significant reduction in loneliness for those playing the Wii. |
|  | **Television Intervention with a Partner**  *(Kahlbaugh PE, 2011 from Hagan R, 2014)* | Quantitative (RCT) | Over 12-week period, research assistants visited older adults and either watched television or played Wii with them. The research assistants were encouraged to be socially responsive to their partners. | M: In-person I: One-to-one N = 35 | Older adults had a significant increase in loneliness for those watching television. |
|  | **Virtual Intergenerational Reverse-Mentoring Program Cyber-Seniors**  *(Juris JJ, 2022)* | Quantitative (Quasi-experimental) | This intervention connects younger and older adults 61-86 years who are in rural areas so that the older adults could connect with younger adults to learn various things like finding YouTube videos, learning how to use a spreadsheet in Excel, connecting to Bluetooth in a car etc. This program lasted for about 1-4 sessions over the course of 3 months and each session was about 1.5 hours. | M: Virtual (Computer) I: One-to-one N = 9 | There was no change in social isolation (p=0.61). However, there was improvement in loneliness however the results were not significant (p=0.17). |
| **Aging in place (n=31)** | **Home care interventions - Home visit interventions (n = 13)** | | | | |
|  | **‘Someone To Talk to’ Intervention** *(Eliezer K, 2022)* | Qualitative | First-year social work students conducted a telephone intervention program for older adults in the community during the Coronavirus crisis. The program aimed to address physical and emotional challenges faced by older individuals under lockdown by offering in-kind services such as medication and food deliveries. Additionally, it aimed to respond to emotional distress and disruptions in routine caused by the lockdown. The program also had a focus on identifying at-risk older individuals and providing them with emergency care. | M: Virtual (Telephone) I: One-to-one N = 142 | Participants and students indicated that they benefitted from the programme and felt that the therapeutic relationships were meaningful. |
|  | **CARELINK Program**  *(Nicholson NR, 2013)* | Quantitative (Quasi-experimental) | University student model of care intervention that included nursing students to visit community dwelling older, community dwelling people to aid socialization using a variety of techniques (reminiscence, exercise-talk discussions, goal-oriented, social engagement-directed discussions, coaching, modelling) | M: In-person I: One-to-one N = 56 | Community dwelling older adults had significantly decreased social isolation. |
|  | **ElderHelp Concierge Club (CC)** *(Scharlach AE, 2015 from Tricco AC, 2022)* | Quantitative (Quasi-experimental) | This community-based intervention integrates care, volunteer services, assessments, and tiered services. Participants contacting CC undergo eligibility assessments and are categorized into Tier 1 (transportation) or Tier 2 (other CC services with in-home assessment). The model involves a comprehensive approach, including personal and environmental assessments, multilevel care coordination, professional and volunteer providers, and a fee model adjusted by income. Time points for assessment are at baseline and 6 months. | M: In-person I: NR  N = 21 | Social isolation and contact with family and friends did not change significantly overtime. |
|  | **Health Teams Advancing Patient Experience: STRengthening QualitY (Health TAPESTRY)** *(Dolovich L, 2019 from Tricco AC, 2022)* | Quantitative (RCT) | For participants with an average age of 78, this intervention involved trained community volunteers assessing health needs through surveys and narratives. The volunteers collaborated with a clinical team to create iterative plans addressing participants' health goals and issues. Community volunteers collected info on life and health goals, risks, and needs through surveys and narratives, sending reports to the primary care interprofessional team. The team reviewed and acted upon plans of care, involving community agencies and volunteers, with ongoing follow-up. | M: In-person I: One-to-one N = 312 | After 6 months of follow-up, no statistically significant differences were observed between groups for social network scores (mean difference 0.038; 95% CI –0.25 to 0.33) and social satisfaction scores (mean difference 0.102; 95% CI –0.35 to 0.55). |
|  | **Home Visits** *(Hall N, 1992; McEwan RT, 1990; Van Rossum E, 1993; Sørensen KH, 1988; Vetter NJ, 1984 from van Haastregt JC, 2000)* | Quantitative (RCT) | Community-dwelling participants 65+ years were part of the home visits intervention which aimed at looking at psychosocial functions. The intervention was based on reducing risks and problems of ageing. Home visits consisted of evaluation of issue and then resources were provided (medical, psychosocial etc.). The recommendation helped to reduce the risks found in ageing. | M: In-person I: NR  N = NR | Out of the five studies, only four reported on loneliness, whereby 4/5 reported no significant effect. |
|  | Home-Based Health Services (McEwan RT, 1990; Bartsch DA, 2009; Bartsch DA, 2013 from Paquet C, 2023) | Mixed-and/or multi-method | Home-based health services include interventions that are primarily one-on-one and provided by a regulated health care professional. They are based outside of an institutional setting and aim to directly or indirectly address social care needs and healthy living standards. | M: NR  I: NR  N = NR | Overall, this intervention type improved social isolation and loneliness. |
|  | **Peer Counseling and Social Engagement** *(Carandang RR, 2020)* | Quantitative (Quasi-experimental) | The combined intervention involved older adult peer counsellor volunteers conducting weekly one-hour home visits for three months with Filipino clients at risk of depression. These peer counsellors, trained for leadership and peer counselling, aimed to establish a strong alliance, identify client-defined problems, and encourage behaviours change. Simultaneously, these older Filipino adults at risk for depression participated in 3-hour weekly social events at the Office for Senior Citizens Affairs (OSCA) Center, addressing various topics related to healthy aging—both intervention components aimed to enhance social networks and promote active social participation. | M: In-person I: Mixed (Group-based, one-to-one) N = 133 | Perceived social support significantly improved, however, there was no meaningful change in loneliness. |
|  | **Pet Support Program (PSP)**  *(Cryer S, 2021)* | Qualitative | This study explores the experiences of individuals over 65 using Pet Support Programs (PSPs) in Melbourne, Australia. Fourteen participants, aged 65-90, predominantly female and Caucasian, were selected based on their use of PSPs. Most were dog owners, and participation involved expressing interest, passing screening, and being matched with a volunteer based on factors like personality and location. Participants and volunteers determined visit parameters, frequency, and duration, with an annual fee of A$30 charged by PSP providers. | M: In-person I: NR  N = 14 | Through the use of the PSP, the elderly were able to improve their quality of life and health and lessen their social isolation. This study ascertained that recipients of PSPs experience improved social connectedness. |
|  | **Psychological Support Service for Socially Isolated Elderly People (PSIE)** *(Santos-Olmo AB, 2022)* | Quantitative (Quasi-experimental) | The home care intervention, operating within the Madrid City Council's municipal care network, targets socially isolated individuals aged 65 and above. It employs five principles: quality, outreach, case management, personal assistance, and continuity of care. The intervention includes engagement, comprehensive assessment, and psychosocial support to address health and psychosocial needs. The primary objectives include strengthening the social network, destigmatizing isolation, and connecting individuals with normalized health services. Inclusion criteria require individuals to be 65 or older, live alone or with others over 65, have uncovered social and/or health needs, lack social support, refuse assistance from normalized services, and have exhausted municipal social service interventions without resolution. The intervention supports involuntary institutionalization and/or legal incapacitation as a last resort. | M: In-person I: One-to-one N = 68 | Pre-post, there were significant improvements observed in unmet social needs, global functioning, as well as health and psychosocial functioning. |
|  | **Safety and Care Services (SCS)** *(Lim JW, 2023)* | Quantitative (RCT) | Participants aged 50 and above participated in a three-month intervention involving the installation of sensors in their homes to identify life patterns and provide safety support and services, aiming to decrease loneliness. In addition, participants received support from SCS coordinators through home visits and phone calls, during which they provided safety support and service linkage (e.g., to health resources). The sensors helped inform the service provided by the coordinators. | M: Mixed (In-person and virtual (telephone)) I: Self-directed N = 40 | There were no significant differences in loneliness between the intervention and control group. |
|  | **Social Health Intervention** *(Clarke M, 1992 from Ibrahim AF, 2022)* | Quantitative (RCT) | This intervention involves participants 75+ years who are living alone. This in home assistance allows for the social worker to help the participant with collecting pension and liaising with administered for various things needed by the participants. | M: In-person I: One-to-one N = 523 | There were no significant differences for perceived loneliness. |
|  | **The Community-Based Health Home (CBHH) Model**  *(Sadarangani T, 2019)* | Quantitative (RCT) | Incorporates Health Home required services using strength-based Adult Day Health Care model (community-based centers designed to provide a socially supportive environment and health services to adults who require supervised care and health services during the day). CBHHs aim to stabilize individuals’ social, medical, and psychological conditions and reduce unnecessary utilization of health services while improving quality of life and self-care capacity. | M: In-person I: Group-based N = 176 | Quantitative: Significant improvement in loneliness and quality of life.  Qualitative: Participants felt the intervention facilitated meaningful peer relationships, supporting productive engagement (engaging in volunteering activities, or celebrating holidays) and reduced social isolation |
|  | **The Public Health Intervention "Fall Prevention"** *(Ožić S, 2020)* | Quantitative (Prospective controlled trial) | The intervention consisted of 2 activities for independently living older adults (75 years or older): determining and eliminating the risk of falls in the intervention participants home and organized custom exercise twice a week. The nurses at the participants’ home pointed out the risky obstacles in the space, educating the participants about safe movement in old age. Physiotherapists and physiotherapy students designed and maintain custom exercise for elderly at 4 locations in the City of Rijeka | M: In-person I: Mixed (Group-based, one-to-one) N = 410 | The intervention did not improve loneliness, or total, psychological, or social frailty. |
|  | **Home care interventions - Telehealth interventions (n = 7)** | | | | |
|  | **Care TV Duplex Video/Voice Network**  *(Van Der Heide LA, 2012)* | Quantitative (Quasi-experimental) | Older adults (mean age 73.2) used the intervention with the applications: (1) Alarm Service (2) Care Service (3) Good morning/good evening service (4) Welfare and housing and (5) Family Contact. It allowed clients to communicate 24 hours, 7 days a week with a nurse practitioner. | M: Virtual (Alarm, video) I: One-to-one N = 130 | Older adults had significantly decreased feelings of loneliness. |
|  | **Social Internet-Based Intervention Activities (SIBA)** *(Larsson E, 2016)* | Quantitative (RCT) | The intervention programme combines individual and group meetings, including in-home support and remote support via the internet or telephone from occupational therapists for older adults age ≥60 years. | M: Mixed (In-person and virtual (not specified)) I: Mixed (Group-based, one-to-one) N = 30 | Loneliness significantly improved in older adults and was sustained post-intervention. Satisfaction with social connection had non-significant changes. Changes in social-interaction skills showed mixed results. |
|  | **Social Bridging Project** (Noble LW, 2022) | Mixed-and/or multi-method | The Social Bridging Project sought to alleviate the impact of social isolation on older adults and enhance their comfort in expressing feelings of loneliness. Trained volunteers conducted wellness calls, engaging participants in conversation, offering technology-based support, and providing access to necessary resources. The project addressed issues related to technology, food access, medication management, and medical/mental health emergencies. Project staff and student volunteers underwent five hours of training, covering active listening, crisis intervention, and various call scenarios. Students were matched with participants based on their field of study. | M: Virtual (telephone) I: One-to-one N = 13 | Qualitative: Most participants reported decreased loneliness and increased connectedness after the calls. Half of participants reported increased ease in expressing their feelings. |
|  | **Tele-Health Interventions** *(Bond GE, 2010; Morrow-Howell N, 1998 from Paquet C, 2023)* | Mixed-and/or multi-method | Interventions in this category involved internet-based platforms to increase social support, help with suicide crisis, and reduce social isolation. | M: NR  I: NR  N = NR | Quantitative: There were only a few interventions in this category that were effective in decreasing loneliness and social isolation. |
|  | **Telecare** *(Bowes A 2013 from Heins P, 2021)* | Qualitative | The intervention addressed social participation in community-dwelling older adults with dementia using telecare. | M: Virtual (not specified) I: One-to-one N = Older adults = 76; Caregivers = 16 | Using telecare enhanced relationships but led to narrowing of social networks. |
|  | **The Use of Medical Alert Device** *(Morgenstern LB, 2015 from Johnstone G, 2021)* | Quantitative (Block RCT) | The aim of this study was to determine the benefits of wearing a medical alert device for healthy elderly women living alone for 90 days. This intervention was based on a 90-day trials where the medical device would for a range up to 120m from the speakerphone. There was a small call button (through a wristband or necklace) and if pressed would service an ambulance. | M: NR I: Self-directed N = 265 | There were no changes in perceived isolation or social connectedness. |
|  | **Video-Telephone Nursing Care**  *(Arnaert A, 2007 from Khosravi P, 2016)* | Quantitative (Cross-sectional) | Video-telephone nursing care for homebound adults age ≥60 years. | M: Virtual (Telephone, video);  I: One-to-one  N = 71 | Loneliness decreased after the video-telephone nursing care. |
|  | **Home care interventions - Meal delivery interventions (n = 4)** | | | | |
|  | **Food Recovery-Meal Delivery Program** *(Ross JM, 2022)* | Mixed-and/or multi-method | The study assessed the impact of a food recovery-meal delivery initiative on the nutritional status and well-being of homebound seniors. Eligible participants, aged 60 or older and on the Meals on Wheels waitlist, received home-delivered meals twice a week. The program, managed by student volunteers and overseen by registered dietitians, involved recovering unused food from hospitals, repackaging it into balanced meals, and delivering them to seniors' homes. The meals aimed to supplement existing food intake, promoting nutritional health &social well-being. | M: In-person I: One-to-one N = 49 | Statistical analysis indicated a significant improvement in well-being and loneliness. Thematic analysis revealed that meal recipients perceive that food recovery-meal delivery programs may improve their well-being. |
|  | **Home-delivered meals services - Dartmoor Community Kitchen**  *(O’Leary MF, 2020)* | Quantitative (Quasi-experimental) | The intervention includes three-week, daily meal provision service by a non-profit provider (Dartmoor Community Kitchen). | M: In-person I: One-to-one N = 19 | Home-delivered meal services did not reduce loneliness, social capital, life satisfaction, or sense of belonging in community dwelling older adults. |
|  | **Home-delivered meals services - Meals on Wheels**  *(Wright L, 2015)* | Quantitative (Quasi-experimental) | Participants in the Meals on Wheel intervention (age ≥55 years) received at least three home-delivered meals per week. | M: In-person I: One-to-one N = 62 | Improvements in loneliness and well-being were significant. |
|  | **Home-delivered meals services – Meals on Wheels** *(Thomas KS, 2016)* | Quantitative (RCT) | Participants in the intervention received home-delivered meals (regardless of delivery method) from the organization Meals on Wheels. | M: In-person I: One-to-one N = 376 | Home-delivered meal services significantly reduced loneliness in at risk older adults. |
|  | **Day care centres (n = 4)** | | | | |
|  | **Day Care Services**  *(Lunt C, 2021)* | Quantitative (Quasi-experimental) | Participants aged 65-99 with long-term conditions engaged in services at day centers, including games and exercise, for 12 weeks. The study compared paid, blended, and volunteer-led services with the aim of increasing quality of life and community engagement. All services involved older individuals who had undergone a needs assessment for physical, cognitive, and social well-being leading to a referral to the day centre. | M: In-person I: Group-based  N = 94 | While no statistically significant differences were reported for loneliness, a trend showed a reduction in mean total loneliness between baseline and 12 weeks for blended and voluntary services but an increase for those in paid staff services (46%, compared to 32% of blended service clients and only 10% of voluntary service users, p = 0.02).No significant differences were found for self-reported health status. |
|  | **Day Centres for Older People** *(Orellana K, 2020)* | Mixed-and/or multi-method | This study thoroughly examined four generalist day centres, considering them as potential services to assist individuals with evolving needs in staying at home. Day centres for older people are community-based services offering care, health-related services, and activities tailored for disabled or needy older individuals. Attendance can be for a full or partial day and may span any number of days. | M: In-person I: One-to-one  N = 23 | Quantitative: Attendance enhanced quality of life significantly. Qualitative: In addition, it facilitated companionship, activities, practical support, offset loneliness, and enabled maintenance of social connections. |
|  | **Nursing Intervention Program**  *(Abdel-Aziz HR, 2022)* | Quantitative (Quasi-experimental) | This intervention, hosted at a social club for participants aged 60+, aimed to assess a nursing program's impact on reducing loneliness. The program included sessions on depression, loneliness, and their effects on physical health; facilitation of social interaction; development of recreational activities; mindfulness and stress reduction techniques; cognitive therapy; and improvement of elderly self-esteem. | M: In-person I: Group-based  N = 50 | Overall, there was a statistically significant decrease in feelings loneliness post-intervention (p=0.01). |
|  | **Urban Health Centres Europe (UHCE)** *(Franse CB, 2018 from Tricco AC, 2022)* | Quantitative (Quasi-experimental) | This intervention, targeting an average age of 79 years, implemented a preventive coordination pathway addressing falls, medication, and loneliness based on participants' needs. The control group received standard care. The preventive health assessment covered multiple dimensions and, if a person was deemed at risk, involved coordinated care pathways for fall risk, appropriate meds use, loneliness, and frailty. | M: In-person I: NR  N = 2325 | The adjusted analysis revealed a statistically significant small positive effect on loneliness (B=−0.18, 95% CI −0.35 to −0.02), when compared with usual care. |
|  | **Village Membership for Ageing in Place** *(Graham C, 2018 from Johnstone G, 2021)* | Quantitative (Cross-sectional) | Village members allows for older adults to age in place, be socially connected, and have health services accessible. The 12-month longitudinal analysis reported on those participating in the villages. This Village model was a membership organization to promote ageing in place and independently (was highly community based). This Village was based on promoting awareness of services through information and referrals. | M: In-person I: NR  N = 222 | Respondents felt more socially connected. However, in the pre-post-test there were reports of decreased frequency in talking to friends (p=0.0425), but no significant difference in getting together socially or feeling like you belong to the community. |
|  | **Village centres (n = 3)** | | | | |
|  | **Village Membership for Ageing in Place** *(Graham CL, 2014 from Johnstone G, 2021)* | Quantitative (Cross-sectional) | Village members allows for older adults to age in place, be socially connected, and have health services accessible. The 12-month longitudinal analysis reported on those participating in the villages. This Village model was a membership organization to promote ageing in place and independently (was highly community based). This Village was based on promoting awareness of services through information and referrals. | M: In-person I: NR  N = 282 | Membership significantly encouraged social engagement. |
|  | **Village Membership for Ageing in Place** *(Graham CL, 2017 from Johnstone G, 2021)* | Quantitative (Cross-sectional) | Village members allows for older adults to age in place, be socially connected, and have health services accessible. The 12-month longitudinal analysis reported on those participating in the villages. This Village model was a membership organization to promote ageing in place and independently (was highly community based). This Village was based on promoting awareness of services through information and referrals. | M: In-person I: NR  N = 1753 | There were reports that those who had village membership had a greater impact on being socially connected and receiving social support. There was also an improved quality of life. Those living alone also reported great improvement in ageing in place. |
| **Socially Assistive Robots and Computer Agents (n=29)**  *Socially assistive robots (SARs) are one type of robot that aid humans through social interaction and comprise pets, companions, service robots, or both companion and service (Bedaf S, 2015). They aim to provide emotional, cognitive, and social support/cues. Computer agents are screen-based, digital entities that may include a dialogue system and animation (Norina G, 2021).* | **Computer agents (n = 14)** | | | | |
|  | **Always On Virtual Agent** *(Sidner CL, 2018 from Gasteiger N, 2030)* | Mixed-and/or multi-method | Participants used the Always On in their homes to decrease loneliness and increase happiness. | M: In-person I: One-to-one N = 44 | Quantitative: Loneliness and social support did not significantly change. Qualitative: Multiple participants reported the agent provided social support and companionship. |
|  | **Assistant Technology - Alexa Echo** *(Balasubramanian GV, 2021)* | Mixed-and/or multi-method | This intervention involved providing participants aged 50-90 with a voice-activated assistive technology device, specifically the Alexa Echo Show 8, for up to 2 months. The goal was to explore the user experience of the device, which features a screen and voice-controlled personal digital assistance with various built-in skills. Participants, who needed their own internet supply, were required to set up the device independently or with the assistance of a friend or family member. | M: Virtual (Alexa-Echo) I: Self-directed N = 44 | Qualitative: The device had a positive impact on the health and social well-being of the users. Additionally, for those living alone, the device helped combat perceptions of loneliness. Most patients felt they made them more independent. |
|  | **Conversational Agent**  (Ring L, 2013 from Choi HK, 2021) | Mixed-and/or multi-method | A virtual agent supports individuals aged 55 and older living alone. Appearing as a person on screen, it assesses their emotional state, offering tailored feedback to encourage physical activity and combat depression, fostering social support. | M: Virtual (Computer) I: Self-directed  N = 16 | There was a significant decrease in loneliness in the proactive group compared to the passive group. |
|  | **Computer Conversational Agent**  *(Ring L, 2013 from Khosravi P, 2016)* | Quantitative (Quasi-experimental) | Participants used the computer conversational agent-based system for a week. | M: Virtual (Computer) I: Self-directed N = 14 | The conversational agent led to participants feeling a sense of companionship with the agent and using the system reduced perceived loneliness. |
|  | **Embodied Conversational Agent (ECA) “FitTrack”** *(Bickmore TW, 2005 from Heins P, 2021)* | Quantitative (Clinical controlled trial) | The intervention examined was daily interaction with an embodied conversational agent (ECA, FitTrack (who acted as an exercise advisor) for 2 months. | M: NR I: NR  N = 21 | Loneliness decreased statistically significantly in the control group (p < 0.05) but not in the intervention group. No significant differences were found between the two groups. |
|  | **Embodied Conversational Agent (ECA) Motion Sensor** *(Ring L, 2015 from Heins P, 2021)* | Mixed-and/or multi-method | The intervention examined interacting with an ECA motion sensor on a touchscreen computer for 1 week. | M: NR I: NR  N = 14 | Loneliness significantly decreased. |
|  | **Personal Voice Assistants (PVA)** *(Jones VK, 2021)* | Mixed-and/or multi-method | The intervention targeted independent living participants aged 77-96, involving interaction with a device (e.g., giving commands like "Alexa, what's the weather today?") at least five times daily over an 8-week period. Researchers set up the Echo in participants' homes, provided training, and monitored device usage. Participants were required to interact with the device at least five times daily for the first four weeks, followed by voluntary usage for the remaining four weeks, allowing for a naturalistic approach to device interaction. | M: Virtual (Amazon Echo) I: Mixed (Self-directed, one-to-one) N = 16 | Loneliness significantly reduced overtime. |
|  | **Relational Agent** *(Bickmore TW, 2005 from Choi HK, 2021)* | Quantitative (Quasi-experimental) | This humanoid agent virtually updates health for intervention (73.8 years mean age) and control (74.2 years mean age) groups. It appears as a person on screen, discussing daily steps, displaying progress graphs, offering feedback, addressing obstacles, and setting goals for the next day. | M: Virtual (Computer) I: NR  N = 21 | Well-being and loneliness did not significantly change (p > 0.05). |
|  | **Tanya Conversational Agent** *(Ring L, 2015 from Gasteiger N, 2029)* | Mixed-and/or multi-method | The Tanya Conversational agent was assigned to participants to provide longitudinal social support to isolated older adults. | M: In-person I: One-to-one N = 12 | Quantitative: There was a non-significant trend towards decreased loneliness. Qualitative: Diary measures showed that participants reported feeling less lonely with the proactive system than with the passive system. They also reported that the agent gave them social support. |
|  | **Tanya Conversational Agent** *(Vardoulakis LP, 2012 from Gasteiger N, 2031)* | Mixed-and/or multi-method | Participants were given an embodied conversational agent that provided social support to isolated older adults. | M: In-person I: One-to-one N = 12 | All participants had something positive to say about their experience and many reported that the agent provided a sense of companionship. |
|  | **Virtual Robot** (Sidner CL, 2018 from Rivera-Torres S, 2021 ) | Quantitative (Quasi-experimental) | This intervention was based on having a virtual robot conversational agent for companionship for those 55+ years living at home. | M: Virtual (Computer) I: NR  N = 44 | There were no significant changes in relationships status. |
|  | **Care Coach Conversational Animal/Pet Avatar** *(Chi NC, 2017 from Gasteiger N, 2023)* | Mixed-and/or multi-method | Participants received a Care Coach Conversational animal/pet avatar that could be used at home. It provided companionship, entertainment, reminders, and assistance. | M: In-person I: One-to-one N = 10 | Qualitative: Most stated that the agent provided companionship and enhanced their social interaction. |
|  | **Digital Pet Avatar** *(Chi NC, 2017 from Heins P, 2021)* | Qualitative | The intervention examined older adults’ daily interaction with a conversational agent (cat or dog avatar) for 3 months. | M: In-person I: One-to-one N = 10 | Benefits included companionship and increased social interactions. |
|  | **Virtual Companion** *(Machesney L, 2014 from Rivera-Torres S, 2021)* | Mixed-and/or multi-method | The intervention involved the use of a virtual companion accessible via tablet, targeting individuals aged 65 and above who live at home. The virtual companion, described as a virtual pet application, was designed to fulfill emotional relationships for older adults. The technology, implemented in the form of a web-based pet application, was mediated by a human helper. Its purpose was to provide companionship by visually monitoring older adults, engaging in deep conversations, and having the capability to contact caregivers in case of an emergency. | M: Virtual (Tablet) I: NR  N = 13 | Qualitative: There was a reduction in loneliness. |
|  | **Robots (n = 16)** | | | | |
|  | **Animatronic Pet Program** *(Tkatch R, 2021)* | Quantitative (Quasi-experimental) | Lonely older adults were provided with an animatronic pet (cat or dog), instructed to treat it as a pet. Surveys (T1, T2, T3) were administered at pet receipt and approximately 30 and 60 days later. Over four weeks, participants received twice-weekly interactive voice reminder (IVR) calls encouraging pet interaction. IVR calls prompted participants to report interactions (0-no, 1-yes) and daily interaction times (0, 1 (1–2 h), 2 (3–5 h), or 3 (6þ hours)). Calls were scheduled on Tuesdays and Fridays between 10 am and 6 pm in their time zone; missed calls resulted in data collection gaps. The study aimed to assess whether pet ownership reduced loneliness and improved well-being in this demographic. | M: In-person I: Self-directed N = 216 | Loneliness and resilience significantly improved over time. |
|  | **Digital Pet**  *(Chi NC, 2017)* | Qualitative | An embodied conversational agent, which has a computer-generated character that facilitates real-time communication between computers and users. This intervention involved the use of conversational agent in the form of an avatar (termed “digital pet”) for older adults. | M: Virtual (Computer) I: Self-directed N = 10 | The digital pet provided older adults enhanced companionship and social interaction. |
|  | **Joy for All Companion Pets** *(Hudson J, 2020 from Gasteiger N, 2026)* | Mixed-and/or multi-method | The intervention involved providing participants with Joy for All companion pets, which are robot companion pets designed to be used at home. These robot companion pets were specifically targeted towards self-identified lonely individuals with the intention of alleviating loneliness. | M: In-person I: One-to-one N = 20 | Qualitative: Some felt companionship when taking the robot with them during the day. The robot helped forge new connections for those who were shy or felt uncomfortable interacting with new acquaintances. Many thought the robot’s presence positively influenced their feelings of loneliness. |
|  | **Parret Shaped Social Robot**  *(Lim J, 2023)* | Quantitative (Quasi-experimental) | This program targeted participants aged 65 and above in senior welfare centers, involving virtual interactions with the social support and tablet-based parrot-shaped robot PIO twice a week for six weeks in 50-minute sessions to reduce loneliness. The program, structured around the storytelling of PIO's growth, comprised 12 sessions featuring gymnastics and interactive activities with PIO, lasting about 30 minutes per session. | M: In-person I: Self-directed N = 64 | Overall, there was a significant decrease in pre-post values for loneliness (p<0.001), and a non-significant increase in quality of life (p = 0.066). |
|  | **Robotic Pet** *(Hudson J, 2020)* | Mixed-and/or multi-method | The study focused on understanding the perspectives and experiences of self-reported lonely individuals aged 65 and above who participated in an intervention involving robotic companion pets. These community-dwelling older adults were provided with robotic pets equipped with interactive features, including touch-activated sensors, reciprocal "nuzzling" effects, and responses to light stimuli. Robotic dogs and cats were offered in various colors. Over four weeks, they received interactive voice reminder phone calls twice a week, encouraging interaction with the pet and prompting participants to record their interaction details, such as the duration. | M: In-person I: Self-directed N = 20 | Qualitative: Robotic pets may provide the benefits of companionship. |
|  | **Robotic Pet** *(Pollak C, 2022)* | Quantitative (RCT) | Community-dwelling participants 65+ who were discharged from the hospital received either a robotic dog or cat. This intervention lasted for 30 days after the participant was discharged. The pet was interactive (reacted to hugs, petting, and motion) and had soft fur. The aim was to provide companionship for participants. | M: In-person I: Self-directed N = 220 | Overall, social frailty did not show any significant change in the intervention group. |
|  | **Ed Robot** *(Wang RH, 2017 from Gasteiger N, 2032)* | Qualitative | An Ed Robot was assigned to participants people with Alzheimer’s disease. The device provides stepwise prompting to complete at-home activities. | M: In-person I: One-to-one N = 10 | The robot was seen to provide companionship. |
|  | **Hyodol Human-Robot** *(Lee OE, 2023)* | Qualitative | Participants, who owned a doll-shaped companion robot named Hyodol for an average of 18 months, were examined within a friendship framework. Hyodol, a Korean-manufactured humanoid robot resembling a seven-year-old grandchild, was distributed to economically disadvantaged older adults through a public-private partnership. Equipped with AI features, Hyodol engages in two-way conversations and offers reminders for meals, medication, medical appointments, and social interactions. It also promotes exercise through guided programs, interactive touch, and positive verbal feedback, fostering a sense of companionship and emotional connection. | M: In-person I: One-to-one N = 12 | Qualitative: Participants reported reduced feelings of loneliness. |
|  | **Max Homecare and Companion Robot** *(Gross H, 2015 from Gasteiger N, 2025)* | Mixed-and/or multi-method | The Max Homecare intervention involved providing participants with a companion robot designed to offer various functionalities. The companion robot serves as a versatile support system by providing reminders, acting as a fitness coach, assisting with video calls, monitoring vital signs, displaying photos and videos, presenting weather forecasts, and responding to emergencies. | M: In-person I: One-to-one N = 9 | Qualitative: The robotic companion helped to cope with feelings of loneliness. |
|  | **NAO Robot with Incorporated Shakespearean Text** *(Fields N, 2021)* | Quantitative (Quasi-experimental) | This intervention incorporated a robot called NAO and Shakespearean text to encourage participatory activity between the participants (65+ years) and robot. The intervention worked in multiple sessions (visual, theatrical, and haptic) where the sessions were facilitated by a moderator. NAO works by interacting with the participant (for example: performing a sonnet together). The aim was to improve mood and loneliness and help with quality of life. | M: In-person I: One-to-one N = 15 | Overall, there was a significant decrease in loneliness (p=0.04). |
|  | **Prototype Robot** *(Zuckerman O, 2020 from Gasteiger N, 2033)* | Mixed-and/or multi-method | Participants engaged with a prototype robot that offered a cognitive word game designed for healthy older adults, where social interaction was a secondary but enjoyable aspect. The robot facilitated mental stimulation through the word game, contributing to the cognitive engagement of the participants. | M: In-person I: One-to-one N = 39 | Qualitative: Some felt they had formed a connection, and 14 out of 39 participants described the robot’s potential to relieve loneliness. |
|  | **Socially Assistive** **Robot, JamesVR** *(Van Assche M, 2023)* | Qualitative | An assistive robot, JamesVR, was deployed in the homes of older adults with initial cognitive decline as part of the ReMIND project. The study aimed to explore the robot's impact on loneliness and its contribution to meaningful time use and activities. Potential participants were contacted, and after obtaining consent, JamesVR was placed in their homes to minimize visits. Participants received an explanation, a demonstration of features, and a manual with visual guidance. | M: Virtual (Computer) I: Self-directed N = 4 | The robot showed promise in providing companionship and reducing loneliness and social isolation. |
|  | **Giraff** *(Cesta A, 2016 from Isabet B, 2021)* | Qualitative | Mobile-telepresence robots can be used to create connections and be social assistive robots where participants can interact and promote their participation in communication, movement, health, tasks, and use for videoconferencing. The aim of the study was to see how social robots can help with loneliness and social isolation. | M: NR I: NR  N = 2 | MTR was appreciated for its ability to alleviate loneliness. |
|  | **Giraff and Paro**  *(Baisch S, 2017 from Isabet B, 2021)* | Quantitative (Cross-sectional) | Mobile-telepresence robots can be used to create connections and be social assistive robots where participants can interact and promote their participation in communication, movement, health, tasks, and use for videoconferencing. The aim of the study was to see how social robots can help with loneliness and social isolation. | M: NR I: NR  N = 29 | There was an improvement in social contact. |
|  | **MTR Texai project** *(Beer JM, 2011 from Isabet B, 2021)* | Qualitative | Mobile-telepresence robots can be used to create connections and be social assistive robots where participants can interact and promote their participation in communication, movement, health, tasks, and use for videoconferencing. The aim of the study was to see how social robots can help with loneliness and social isolation. | M: NR I: NR  N = 12 | There was a decrease in social isolation. |
|  | **MTR-VGO System** *(Seelye AM, 2012 from Isabet B, 2021)* | Qualitative | Mobile-telepresence robots can be used to create connections and be social assistive robots where participants can interact and promote their participation in communication, movement, health, tasks, and use for videoconferencing. The aim of the study was to see how social robots can help with loneliness and social isolation. | M: NR I: NR  N = Healthy OAs (n = 8) and MCI OAs (n = 1) | There was potential to increase social connectedness, and well-being. |
| **Befriending interventions (n=27)**  *Befriending interventions aim to introduce individuals to others who provide them with additional social and emotional support over time (Siette J, 2017). The relationship between the individual and the befriender (a trained volunteer or peer), is based on social and emotional closeness.* | **Technology-mediated befriending interventions (n = 14)** | | | | |
|  | **Befriending Interventions** *(Jones RB, 2015 from Paquet C, 2023)* | Mixed-and/or multi-method | This intervention category involved befriending initiatives that were telephone or video-based and radio programs. | M: Virtual (Not Specified) I: NR  N = NR | Quantitative: Communication-technology interventions were associated with increased social networks and a decrease in loneliness. |
|  | **Call in Time Programme** *(Cattan M, 2011 from Noone C, 2022)* | Qualitative | The intervention was a telephone befriending scheme with volunteers offering emotional support to housebound older adults. | M: Virtual (Telephone) I: One-to-one  N = 40 | The program increased confidence and promoted social participation and meaningful relationships. |
|  | **Call in Time Programme** *(Cattan M, 2011 from Ibrahim AF, 2022)* | Mixed-and/or multi-method | The "Call in Time" program implemented a telephone-based befriending service to support the well-being of older individuals aged 60 and above. The program involved volunteers engaging in conversations with older participants, matching them based on shared interests and hobbies. Over the course of 17 months, participants received weekly 30-minute calls, fostering a friendly and supportive connection. | M: Virtual (Telephone) I: One-to-one N = 40 | Qualitative: The service helped older people reengage with the community and become socially active again. Participants gained a sense of belonging, alleviated loneliness, had increased confidence, increased amounts of ordinary conversation. Participants felt more independent, but there was no difference in general health and a decrease in social functioning. |
|  | **Loneliness Helpline calls at the Friendship at Every Age Program** *(Balta M, 2023)* | Quantitative (Quasi-experimental) | The Loneliness Helpline offered continuous support for participants over 60 who felt lonely or socially isolated during COVID-19. The helpline was to keep participants socially engage with a trained volunteer. The 2-part intervention was followed by the Interconnection Part of the Program (IC), where the participant chose to interact further with the trained volunteer for additional social support. These calls would occur at least once per week, where calls tended to last more than 30 minutes. After three months, the participants were asked to complete structured follow-up questionnaires. | M: Virtual (Telephone) I: One-to-one N = 275 | There was a significant decrease in loneliness for elderly participants (p<0.001). |
|  | **Medical Student-Led Social Phone Calls** *(Hoyumpa G, 2022)* | Quantitative (Quasi-experimental) | Academic medical school students volunteered for unstructured social phone calls to older adults calls who were elder mistreatment victims. | M: Virtual (telephone) I: One-to-one N = 6 | Loneliness dropped from moderate to none/low for older adults taking part in the social phone calls. |
|  | **Mobile application “Gezelsch App”**  *(Jansen-Kosterink SM, 2020)* | Quantitative (Observational) | The Gezelsch App is a social technology to encourage social participation of older adults, encouraged by stimulating older adults to visit local activities together with (new) friends; accessible by smartphone, tablet, and PC; access to a homepage with a tile for each of an inbox for messages, news, activities, information, tips, and friends. | M: Virtual (Computer, smartphone, tablet) I: Self-directed  N = 41 | Unemployed or retried older adults living in the community with a spouse or alone had positively improved loneliness and quality of life however the change was not significant. |
|  | **Nationwide Telephone befriending and Helpline** *(Preston C, 2019 from Noone C, 2022)* | Qualitative | The intervention was a phoneline service including Helpline, Friend's Service, and Well-being Service. | M: Virtual (telephone) I: One-to-one  N = 42 | Friend's service was useful for forming light-hearted or closer, more intimate friendships. |
|  | **NEST Collaborative’s Remote Social Intervention** *(Nolan RW, 2022)* | Quantitative (Cross-sectional) | The intervention involved weekly phone calls between older adults (mean age of 72) and diverse volunteers (18-70 years), aiming to reduce social isolation and associated issues. Differentiating from other programs, NEST utilized one-to-one phone calls, multigenerational volunteers, and a weekly or twice-weekly interaction frequency. The calls focused on empathy-based conversations, addressing emotional and informational social support needs. While primarily for social interaction, the calls also identified unmet needs, leading to instrumental social support referrals. Volunteers were recruited from universities and social services. | M: Virtual (telephone) I: One-to-one N = 31 | Social isolation improved, but not significantly. |
|  | **One-on-one Telephone Friendship**  *(Hind D, 2014 from Heins P, 2021)* | Mixed-and/or multi-method | The intervention examined calls delivered by volunteer facilitators for 6 weeks (10-20 minutes) followed by 1-hour one-on-one telephone friendship groups for 12 weeks. | M: Virtual (telephone) I: Mixed (Group-based, one-to-one) N = 157 | No statistically significant improvement for loneliness. |
|  | **Telephone Befriending** *(Mountain GA, 2014 from Douglas NF, 2023)* | Quantitative (RCT) | This intervention targeted well-being, loneliness, and social care for participants 80+ years who are living at home to engage in phone calls for 10 to 20 minutes one-on-one and in group calls that lasted for approximately 1 hour weekly for 12 weeks. | M: Virtual (Telephone) I: Mixed (Group-based, one-to-one) N = 35 | There were uncertain positive changes to loneliness. |
|  | **Telephone Befriending**  *(Newall NEG, 2015)* | Qualitative | The intervention aimed to offer companionship and the opportunity to establish meaningful friendships with priority populations through connecting them with trained befrienders over the phone. | M: Virtual (Telephone) I: One-to-one N = 26 | Participants felt more connected to the outside world. Socially isolated participants reported the intervention made them feel as if they were more part of a community than those not isolated and mentioned they felt less lonely. |
|  | **Telephone Befriending Program** *(Cattan M, 2011 from Douglas NF, 2023)* | Qualitative | This intervention for older adults was an ongoing telephone befriending program conducted with volunteers as the provider for participants 60-90 years to decrease social isolation and loneliness. | M: Virtual (Telephone) I: One-to-one N = 40 | There was an overall positive effect to feelings of loneliness, social isolation, and self-confidence. |
|  | **The “Call in Time” Telephone Befriending**  *(Cattan M, 2011)* | Qualitative | The telephone support befriending schemes were created with the aim of providing socially isolated older people with affordable means to become more independent and confident. The programs aimed for individuals to develop a sense of increased self-respect, and increased participation in meaningful relationships. | M: Virtual (Telephone) I: One-to-one N = 40 | Participants gained confidence, re-engaged with their community, and became more socially active. Participants talked about a sense of belonging and how they felt less lonely. |
|  | **Volunteer Telephone Befriending** *(Mountain GA, 2014 from Todd E, 2022)* | Quantitative (RCT) | The intervention included using volunteers trained to telephone older people living independently for 20 minutes per week over ten weeks. | M: Virtual (Telephone) I: NR  N = NR | There was no difference in loneliness. |
|  | **Non-technology-mediated befriending interventions (n = 10)** | | | | |
|  | **Befriending Intervention** *(Niemann AL, 2023)* | Quantitative (Quasi-experimental) | Members experiencing social isolation or loneliness had the option to participate in the Caregiver Visits intervention. This intervention involved a befriending model through in-home caregiver visits. | M: In-person I: One-to-one N = 26 | Loneliness and social isolation showed improvement over time, although statistical significance was not formally tested. |
|  | **Befriending Service** *(Wiles J, 2019 from Noone C, 2022)* | Qualitative | The intervention was a befriending service consisting of a volunteer visiting an older person upon their request. | M: In-person I: One-to-one  N = 106 | The program enhanced social networks and connectedness. |
|  | **Caregiver Visits +** *(Niemann AL, 2023)* | Quantitative (Quasi-experimental) | Members facing social isolation or loneliness were given the choice to opt into the Caregiver Visits + intervention. This intervention included befriending through in-home caregiver visits and participation in a social prescribing model known as social navigation. Social navigation involved nurses educating members about social isolation and loneliness, connecting them to local community and internet resources, and providing caregiver support for accessing resources (such as transportation, internet assistance, and event attendance). | M: In-person I: One-to-one N = 23 | Loneliness and social isolation showed improvement over time, although statistical significance was not formally tested. |
|  | **Coffees in the Morning for Patients** *(Johnson L, 2020)* | Mixed-and/or multi-method | This 8-week intervention, designed for patients aged 80 and above, aimed to alleviate social isolation by organizing regular coffee mornings at a GP practice. The sessions, held once a week for 2 hours, provided a platform for patients to come together, share coffee, and contribute ideas for activities such as Thai chi, puzzles, mindfulness, and board games. A designated room, safely accommodating 25 patients, was booked, and a local café generously supplied cakes. Transportation for housebound patients was arranged through a local service, ensuring accessibility. Clinical hours were adjusted to facilitate staff attendance, and various speakers, charities, and exercise specialists were invited to enrich the sessions. | M: In-person I: Group-based N = 25 | Qualitative: Patients reported they felt part of a community and knew how to better access support and services. |
|  | **Community Engaged Arts (CEA)** **Programme**  *(Moody E, 2012 from Poscia A, 2018)* | Qualitative | The CEA program for older community-dwelling adults aimed to provide connections between participants in the Arts, Health, and Seniors Program | M: In-person  I: Group-based N = 20 | Participant’s community connections were expanded through art. Participant’s worked within a group towards shared goals. |
|  | **Friendly Visitors** *(Mulligan MA, 1978 from Ibrahim AF, 2022)* | Quantitative (Quasi-experimental) | This intervention was based on a visitor who visits those 65+ years to encourage less social isolation. The sessions were one hour bi-weekly, and this lasted for 6 months. | M: In-person I: One-to-one N = 23 | There were significant decreases in social isolation and an increase in social contacts. |
|  | **Peer Intervention** *(Kotwal AA, 2021)* | Mixed-and/or multi-method | A two-year peer-outreach intervention involved 74 low-income older adults at an urban senior center. Peers aimed to cultivate trusting relationships and promote socialization by accompanying participants to health visits initially, followed by subsequent visits focused on various social activities. Peers were matched based on demographics and social interests, with flexibility to address participants' health needs and social interests. The frequency of visits depended on participant interest, perceived loneliness, or social isolation. The intervention started with "soft approach" home visits and expanded to include shared meals, group activities, and larger monthly events. | M: In-person  I: One-to-one N = 74 | Loneliness and self-perceived barriers to socializing reduced. |
|  | **Volunteer-based Befriending**  *(Bantry-White E, 2018)* | Qualitative | The program involved weekly home visits by volunteer befrienders with a focus on with a focus on social interaction as well as community-based social groups, activities, and outings. | M: In-person I: One-to-one N = 22 | Participants described the relationship with the befrienders as authentic, and many highly prized their relationships with the befriender. |
|  | **Volunteer-based Befriending**  *(Gardiner C, 2016)* | Qualitative | The befriending service aimed to support socially isolated older people with life limiting conditions. The service provided befriending recipients with a minimum of a 3-hour weekly visit from a trained volunteer. | M: In-person I: One-to-one N = 11 | Benefits of the intervention were reduced social isolation. |
|  | **Volunteer-based Befriending**  *(Smith R, 2018)* | Mixed-and/or multi-method | The intervention aimed to provide companionship and the opportunity to establish meaningful friendships to priority populations with trained volunteer befrienders and incorporated peer support elements. | M: In-person I: One-to-one N = 19 | Quantitative: Social support significantly increased and there was a non-significant reduction in social isolation. Loneliness was not reduced.  Qualitative: The befriending relationship was highly valued and considered a source of emotional support by carers. Participants felt the intervention helped them get through difficult situations and to sometimes cope better with these difficulties. Some participants made new friendships through the interventions. |
|  | **Combination of technology- and non-technology-mediated befriending interventions (n = 2)** | | | | |
|  | **Befriending interventions** *(Lai DWL, 2020; MacIntyre I, 1999; Mountain GA, 2014 from Chau CMS, 2023)* | Quantitative (RCT) | The review included befriending interventions consisting of peer-support home visits, and telephone visits. | M: Mixed (In-person and virtual (telephone)) I: One-to-one N = NR | There were no significant changes in loneliness (2 studies, 116 participants) and social support (2 studies, 82 participants). |
|  | **Befriending Programme** *(Lester H, 2012 from Noone C, 2022)* | Qualitative | The intervention was a befriending service with weekly contact. | M: Mixed (In-person and virtual (telephone)) I: One-to-one  N = 25 | Social engagement was valued by participants. |
| **Peer support group interventions (n=23)**  *Peer support group intervention brings together peers in the community often dealing with similar stressful life difficulties such as health conditions, loneliness, the death of a loved one, or divorce. The intervention usually includes a facilitator; however, while they guide the session, they do not offer treatment to the participants (Helgenson VS, 2000).* | **In-person peer support group interventions (n = 16)** | | | | |
|  | **Art, Exercise and Discussion Based Psychosocial Intervention**  *(Routasalo PE, 2009)* | Quantitative (RCT) | Psychosocial group nursing for community dwelling older adults, including art and inspiring activities, exercise and discussions, therapeutic writing, and group therapy. Weekly sessions over a 3-month period based in community centres | M: In-person I: Group-based N = 235 | Participants reported developing significantly more new friendships compared with control participants at 12 months. No differences were found between groups for loneliness or social networks. |
|  | **Bereavement Crisis Intervention (BCI)**  *(Constantino RE, 1988 from Dickens AP, 2011)* | Quantitative (RCT) | Participants (community-dwelling widows) met weekly for 1.5 hr planned group discussions on set themes across 6 weeks. | M: In-person I: Group-based N = 150 | The bereavement support group reported significantly enhanced structural social support compared with the other groups (social adjustment or control). Socialization was non-significantly improved across 12 months. The results suggest short, rather than long-term benefits of bereavement crisis intervention. |
|  | **Bereavement Support Group** (Stewart M, 2001) | Quantitative (Quasi-experimental) | Four face-to-face support groups for widowed older adults > 55 years were conducted weekly for a maximum of 20 weeks. Each support group was co-led by a peer (widow) and a professional facilitator. | M: In-person I: Group-based N = 23 | Quantitative: The intervention significantly enhanced support satisfaction. There was a trend toward decreased social isolation, emotional loneliness, and social loneliness.  Qualitative: In postintervention semi structured interviews, bereaved seniors reported improved skills in developing social relationships, enhanced coping, and less loneliness. |
|  | **Conducive Communities** *(Andersson L, 1984 from Paquet C, 2023)* | Mixed-and/or multi-method | The intervention included groups of older women discussing a chosen topic or relevant topic (e.g. nutrition, building new relationships, successfully ageing etc.). | M: In-person I: NR  N = NR | This intervention category reduced social isolation. |
|  | **Discussion Support Groups**  *(Anderson L, 1985 from Jarvis MA, 2020)* | Quantitative (RCT) | The intervention consisted of small group meetings which facilitated discussion in older women living in the community. | M: In-person I: Group-based N = NR | Older women experienced statistically significant reduction in loneliness. |
|  | **Men’s Shed Program**  *(Milligan C, 2015 from Poscia A, 2018)* | Qualitative | The sheds provide a physical space for older men to meet, socialise, teach, and learn new skills and participate in ‘Do it Yourself’ or similar activities with each other. All three Sheds were in the community and aimed to target lone-dwelling, lonely and socially isolated older men from deprived areas. | M: In-person I: Group-based N = 62 | The intervention alleviated social isolation through developing important connections between older men. It also provided a supportive environment that positively influenced participant’s well-being. |
|  | **Path: From Loneliness to Participation** *(Coll-Planas L, 2021 from Noone C, 2022)* | Qualitative | The intervention promoted peer support and participation in local community assets. | M: In-person I: Group-based N = 41 | The program provided companionship, social integration, and a sense of belonging. |
|  | **Path: From Loneliness to Participation** *(Coll-Planas, 2021)* | Qualitative | Older individuals with limited social engagement and occasional loneliness were recommended by primary health and social care professionals to participate in 15-week group sessions. Led by social workers or nurses, these 1.5-hour sessions encouraged active participation, following the empowerment theory. Participants engaged in discussions facilitated by professionals, fostering peer support through the exchange of opinions and experiences around loneliness. The sessions also utilized diverse pictures to prompt discussion. Additionally, local older volunteers from the same neighbourhood were involved in connecting participants with community assets. The group collectively visited and engaged in activities across five local community assets to enhance their involvement in these settings. | M: In-person I: Group-based  N = 38 | Different degrees of success were observed among participants in their reported alleviation of loneliness, increase in social relationships and engagement in social activities. |
|  | **Psychosocial Group Rehabilitation**  *(Savikko N, 2009)* | Mixed-and/or multi-method | Lonely older adults participated in psychosocial group rehabilitation where they visited cultural events, produced their own art, and engaged in group exercise and discussions. They also participated in therapeutic writing and group therapy where they wrote about their experiences and loneliness and discussed them in the group. All groups were tailored to participants' interests and allowed them to modify the programs, promoting their empowerment. Various artists visited the art and inspiring activities groups | M: In-person I: Group-based N = 117 | Quantitative: 86% of participants reported that the intervention provided opportunities to make new friends and try new opportunities. 95% reported that their feelings of loneliness alleviated during the intervention. Qualitative: Participants re-engaged in participating in their communities, socially activating them. Participants felt a high sense of belonging to the group as they created a basis for friendships. |
|  | **Self-Management Peer Support Groups** *(Kremers IP, 2006 from Ibrahim AF, 2022)* | Quantitative (RCT) | This intervention consisted of supervision by 2 females where each meeting helps participants 55+ years to discuss self-management topics like comfort, affection, and stimulation. The duration was for 6 weeks where each session per week lasted 2.5 hours. | M: In-person I: Group-based N = 142 | There was a significant improvement in social loneliness, but not after six months. |
|  | **Small Neighborhood Group Meetings** *(Anderson L, 1985 from Johnstone G, 2021)* | Quantitative (RCT) | This intervention was for small social meetings for older women living alone. There were 4 group meetings (2 of which were attended by a home-help assistant) and consisted of 3-5 people. Subjects discussed were retirees, social medication services, leisure activities, and residential areas). | M: In-person I: Group-based N = 57 | There were significant decreases in loneliness (p=0.037) and an increase in social contact (p=0.014). |
|  | **Social Identity Intervention** *(Lai DW, 2020 from Hickin N, 2021)* | Quantitative (RCT) | The intervention spans weekly sessions over a period of 5 months and is grounded in the psychological theories of the Social Identity Approach, employing a peer social program informed by Dynamic Social Impact Theory. | M: In-person  I: Mixed (Group-based, self-directed) N = 60 | There was a statically significant decrease in loneliness for the intervention group compared to the control. |
|  | **Study Circle**  *(Åberg P, 2016)* | Qualitative | This is a Swedish education and learning process that places a strong emphasis on individual learning and participation, and a less hierarchical form of learning with the focus on equality and collective effort, with participants' experiences and study circles directed by the group taking a central position. | M: In-person I: Group-based N = 1499 | Qualitative: The intervention created fellowship, the sense of belonging, and the possibility for meeting people. The social benefits from participating were closely connected to improved well-being. |
|  | **Talking About Emotional & Social Estrangement** *(Anderson L, 1985 from Douglas NF, 2023)* | Quantitative (Cross-sectional) | This intervention was conducted for older adults in 4 sessions where participants 60-80 years spoke about emotional and social estrangement to reduce loneliness. | M: In-person I: Group-based N = 35 | There were positive changes to social integration. |
|  | **The 'Participatory Group-based Care Management**  *(Ristolainen H, 2020)* | Quantitative (RCT) | Group meetings with older adults 65 years of age living alone, in full-time retirement with poor quality of life, aimed at enhancing quality of life and well-being using a participatory and needs-based approach. Designed for older people living alone (in a private home) and experiencing health or well-being deficit, hence at risk of social exclusion. Key elements of the intervention were: i) social support, ii) counselling, and iii) activities; attendance of five group meetings over 6 months lasting 2–3 hours/meeting; facilitated by a care manager and a researcher. Each group meeting combined three key elements (social support, counselling, and activities). | M: In-person I: Group-based N = 392 | Older adults who participated in the group-based care management had reduced loneliness. However, the effect was not significant. There were no changes on quality of life. |
|  | **The Dual-Process Bereavement Group Intervention-Chinese (DPBGI-C)**  *(Chow AYM, 2019)* | Quantitative (RCT) | The Dual-Process Bereavement Group Intervention-Chinese (DPBGI-C) and the control condition, the loss-oriented bereavement group intervention-Chinese (LOBGI-C) comprised weekly, 2-hr sessions for 7 weeks followed by a 4-hr outing in the eighth week delivered by social workers. | M: In-person I: Group-based N = 125 | The DPBGI-C intervention was statistically superior to the traditional LOBGI-C in significantly reducing grief after loss of spouse, and emotional and social loneliness in widowed older adults whose spouse had died with the previous past 2 years. Social support increased. |
|  | **Virtual peer support group interventions (n = 6)** | | | | |
|  | **Caregiving Support** *(Banbury A, 2019 from Mao W, 2023)* | Mixed-and/or multi-method | This intervention was a 6 week at home videoconferencing peer support program to encourage support between dementia caregivers who were 62 years on average. | M: Virtual (Computer) I: Group-based  N = 69 | Qualitative: There were strong virtual social connections developed. |
|  | **Group Online Social Meetings** *(Banbury A, 2017 from Douglas NF, 2023)* | Mixed-and/or multi-method | The intervention involved introducing participants to and supporting them in attending online social meetings. Participants received technology training to familiarize themselves with the online platforms, and health promotion professionals facilitated the intervention. Structured activities were implemented during the online meetings to enhance participant engagement and promote social interactions. | M: Virtual (Not specified) I: Group-based  N = 52 | There was a positive change towards increased social support networks. |
|  | **Koffee Klatch Support Chat Room** *(Hill W, 2006 from Khosravi P, 2016)* | Quantitative (RCT) | Asynchronous, peer-led support chat room (Koffee Klatch) for duration of 22 weeks, providing an opportunity for women (35 to 65 years of age) to chat about various health topics in the presence of health-care experts. | M: Virtual (Computer) I: Group-based N = 183 | Findings showed significant improvement on the social support score, but no significant differences were found in loneliness. |
|  | **Online Messaging for Caregivers** *(McKechnie V, 2014 from Mao W, 2023)* | Mixed-and/or multi-method | This intervention was 12 weeks and was an Alzheimer’s Society's online program for caregivers to share advice, share in discussions and feel supported. This was for participants 56 years on average. | M: Virtual (Computer) I: Group-based  N = 61 | Qualitative: Participants revealed that the online platform allowed for less loneliness and isolation. |
|  | **Support Group** *(O'Connor MF, 2014 from Mao W, 2023)* | Mixed-and/or multi-method | This 8-week program was for caregivers to have a real-time chat in a 3-dimensional environment. This was for participants 60 years on average. | M: Virtual (Computer) I: NR  N = 7 | There were no significant changes to loneliness, but there were lower levels. |
|  | **Virtual Caregiver Support Group**  *(O’Connor MF, 2014 from Khosravi P, 2016)* | Quantitative (Cross-sectional) | 3D virtual environment across an 8-week online dementia caregiver support group. Seven participants used avatars and participated in real-time chat. | M: Virtual (Computer) I: Group-based N = 7 | The 3D virtual environment reduced the level of loneliness however, the effect was not statistically significant. |
|  | **Combined in-person and virtual peer support group interventions (n = 1)** | | | | |
|  | **Memory Café** *(Masoud SS, 2021 from Mao W, 2023)* | Qualitative | This intervention allowed caregivers to socialize and provide a safe, supportive, and inclusive space for them without fear and stigma. Participants age not disclosed. | M: Mixed (In-person and virtual (computer) I: Group-based  N = 12 | This intervention may support social connectedness in those living in lower SES neighborhoods. |
| **Mentorship interventions (n=11)**  *Mentorship interventions facilitate a relationship between mentees and mentors who support them in meeting their unique social needs adapted to the community context (Sipe CL, 2005). The relationship can include friendship; however, it expands beyond that to include mentorship.* | **In-person mentorship interventions (n = 8)** | | | | |
|  | **Cadwyn Môn Programme** *(Roberts JR, 2020)* | Mixed-and/or multi-method | The Cadwyn Môn program, led by Age Cymru Gwynedd and Môn, provided one-to-one and group-based support to individuals aged 50 and older to alleviate loneliness and isolation. Following the referral, a local coordinator facilitated volunteer-client connections. Initially, volunteers offered companionship and support individually, focusing on enhancing social networks, confidence, and independence over 10-15 weeks. Subjective goals were established, and if participants felt ready, volunteers encouraged them to engage with existing community support networks. Participants also gained access to various local services, and a monthly Cadwyn Môn social club was established for referred clients, some of whom were directly referred to the club if volunteer assistance was unnecessary. | M: In-person I: Mixed (Group-based, one-to-one) N = 120 | Following the individually tailored Cadwyn Môn program, a significant reduction in loneliness and isolation was observed. Themes of increased confidence, enriched social life, and enhanced independence emerged as themes. |
|  | **Community Mentoring**  *(Dickens AP, 2011)* | Quantitative (Prospective controlled trial) | Delivered by two volunteer organizations. Mentoring teams supported older adults at risk or experiencing social isolation for 12 weeks, working closely with participants to engage them in meaningful social activities. | M: In-person I: One-to-one N = 200 | There was no evidence that mentoring was beneficial across a wide range of participation outcomes including social participation and social support. Getting along with others significantly worsened for the intervention group. |
|  | **Friendly Visits** *(Baumgarten M, 1988 from Ibrahim AF, 2022)* | Quantitative (Quasi-experimental) | This intervention was based on those who volunteered their time to help elderly participants feel more socially supported by planning leisurely activities. | M: In-person I: One-to-one N = 168 | Social support satisfaction non-significantly decreased. New social ties built did not significantly change. |
|  | **Mentoring Program** *(Dickens AP, 2011 from Douglas NF, 2023)* | Quantitative (Controlled trial) | This mentoring program was conducted over 12 weeks with volunteer mentors to help reduce social isolation and increase social support and social activities for socially isolated participants or participants at risk of social isolation 50+ years. | M: In-person I: NR  N = 200 | There were no changes to feelings of social isolation. |
|  | **Peer to Peer Support (P2P)** *(Schwei RJ, 2021)* | Quantitative (Observational) | This study evaluates P2P support programs for improving the well-being of community-dwelling older adults (65+) in California, Florida, and New York. The focus includes assessing social support and comparing P2P with standard community programs (SCS). Peers received stipends, and participants retained access to standard services. The study targeted older adults at risk, emphasizing companionship, addressing basic health and emotional needs, and providing information on available services. | M: In-person I: NR  N = 448 | Over time, there were no significant improvements observed in loneliness, self-efficacy, social support activities, and instrumental activities of daily living. However, there was a statistically significant improvement in resilience. |
|  | **Peer-Support Program** *(Fuller SM, 2022)* | Qualitative | The peer-support program targeted older community-dwelling adults in San Francisco, focusing on those from a meal program, care clinics, or related service settings. Participants included low-income individuals aged 55 and older, with diverse backgrounds, histories of homelessness, and substance use. The program aimed to alleviate loneliness by matching peers (aged 55 and older) with clients based on common interests, race, and sexual orientation. Peers underwent training and ongoing sessions, meeting clients weekly for various activities like errands and coffee, fostering emotional support. | M: In-person I: One-to-one N = 21 | They found that peers played a flexible, non-clinical role and were perceived as friends. |
|  | **The Tai Chi Mentorship Intervention**  *(Chan AW, 2017)* | Quantitative (RCT) | The intervention involved socially active older adults age ≥60 years (peer mentors) paired with participants to provide social support for 3-months, twice a week for 60 minutes. | M: In-person I: Group-based N = 46 | The intervention significantly improved loneliness in older adults and non-significantly improved social support satisfaction. Nine people said that they made new friendships after joining the classes. |
|  | **The Upstream Healthy Living Centre Intervention** *(Greaves CJ, 2006)* | Mixed-and/or multi-method | The Intervention provides mentoring to older adults who are experiencing life changes or have free time. The mentors work closely with the participants to encourage their interest in community programs such as creative, exercise or cultural activities | M: In-person I: One-to-one N = 172 | Quantitative: At 12 months, there were significant improvements in social support. Qualitative: The data indicated increased social activity. |
|  | **Combination of in-person and virtual mentorship interventions (n = 2)** | | | | |
|  | **Modified CARELINK Programme**  *(Hernández-Ascanio J, 2023)* | Quantitative (RCT) | Nursing students and volunteer staff conducted a comprehensive intervention for community-dwelling older adults aged 65 and above, aiming to alleviate social isolation and loneliness while enhancing overall quality of life. The experimental group received a systematic intervention comprising six home-based face-to-face sessions and five telephone calls. The intervention focused on stimulating social integration and renewed socialization through various components. Activities included objective-oriented exercises integrated into daily routines, discussions on current events, reminiscence therapy to evoke positive social memories, coaching for setting and achieving social goals, modeling appropriate social behavior, and face-to-face discussions addressing the root causes of social isolation, as well as planning engaging activities. This intervention allowed older adults to take the lead during each visit, offering information on community resources to enhance participation and networking. | M: Mixed (In-person and virtual (telephone)) I: One-to-one N = 121 | Loneliness, social isolation, and health-related quality of life did not improve after the intervention. |
|  | **Peer-Based Intervention** *(Lai DWL, 2020)* | Quantitative (RCT) | Community-dwelling older Chinese immigrants (aged ≥ 65) received an 8-week peer support intervention to reduce loneliness and social isolation and improve psychosocial well-being. They received two-to-one peer support through home visits/telephone calls to provide emotional support, problem-solving support, and community resource sharing. | M: Mixed (In-person and virtual (telephone)) I: Pair-based N = 60 | Statistically significant decreases in loneliness and increases in resilience were found. In addition, significantly fewer barriers to social participation and increased life satisfaction were reported. |
|  | **Virtual mentorship interventions (n = 1)** | | | | |
|  | **Caring Callers Program**  *(Lee K, 2021)* | Mixed-and/or multi-method | The Caring Callers program aimed to support homebound older adults (55 years and older) through weekly telephone calls from volunteers, focusing on providing assistance and friendship. Volunteers, trained via Zoom, underwent modules covering the program's purpose, problem-solving strategies, coping skills, listening skills, and cross-cultural communication. The dyads formed included one volunteer (caller) and one homebound older adult (client). Older adult volunteers, recruited through a local Senior Companion Program (SCP), received specialized training on common issues in older adults. Weekly conversations between volunteers and clients covered safety checkups, weekly events, client-raised topics, and coping methods, with an emphasis on emotional support and companionship. | M: Virtual (Telephone) I: One-to-one N = 15 | Quantitative: Findings did not include statistically significant effects on loneliness, and independent living performance. However, self-reported health significantly increased. |
| **General social support interventions (n=1)** | **General social support interventions (n = 1)** | | | | |
|  | **Social Support Interventions** *(Carandang RR, 2020; Chen, MF, 2022; Cohen GD, 2006; Galinha IC, 2022, Heller K, 1991; Hind D, 2014; Johnson JK, 2020; Lai DWL, 2020; Li S, 2022; Ristolainen H, 2020; Rook KS, 2003; Thomas KS, 2016; Yang SY, 2023; Slegers K, 2008; Fokkema T, 2007; Larsson E, 2016; Thomas BH, 2004 from Yu DS, 2023)* | Quantitative (RCT, N-RCT) | This systematic review compared the effects of various non-pharmacological interventions, particularly social support interventions, on loneliness in older adults residing in the community. The study analyzed 27 social support interventions, including 10 using digital means and 17 using in-person approaches. The digital interventions utilized technologies such as digital platforms, while the in-person interventions involved face-to-face interactions. | M: NR I: NR  N = NR | Social support interventions significantly reduced loneliness among older adults with a medium to large effect size (n = 18, Hedges' g = −0.52; 95%CI [−0.96, −0.07]; Z = −2.47, p = 0.02; I2 : 96%, τ2 : 0.86; p < 0.01). Subgroup analysis was conducted for social support interventions which delivered through either non-digital or digital mean. The nondigital interventions were found to significantly reduce loneliness with a large effect (n = 14, Hedge's g = −0.63; 95%CI [−1.16, −0.10]; Z = −2.33, p = 0.02; I2 : 97%, τ2: 0.96; p < 0.01). |

*****M=mode of delivery; I = level of interaction with interventions; N=number of participants; NR = Not reported.

**Table 1b:** Study and intervention characteristics, including a summary of study results and outcomes for *Self-management interventions* (n = 157)

| **Intervention Type** (number of studies)  *Definition* | **Intervention name**  *(Author, year)* | **Study design** | | **Intervention and population description** | **Intervention details** | **Summary of study results and outcomes** |
| --- | --- | --- | --- | --- | --- | --- |
| **Self-management education interventions**  **(n=106)**  *Self-management education is the ongoing process of facilitating the knowledge, skills, and confidence necessary to enable effective self-management of various conditions or disease (Howell et al., 2017). It involves providing individuals with specific knowledge.* | **Social health training interventions (n = 30)** | | | | | |
|  | **Activity group**  *(Harris JE, 1978 from Dickens AP, 2011)* | Quantitative (RCT) | A structured program for older adults involving techniques to expand consciousness and broaden their knowledge of community activities. The activity group met 1 × weekly for 2-hour sessions, across 6 weeks. | | M: In-person I: Group-based N = 102 | Older adults reported increased number of social interaction and significantly increased structural social support compared with controls at six weeks. |
|  | **Adaptation of the Friendship Enrichment Program**  *(Bouwman TE, 2017 from Douglas NF, 2023)* | Quantitative (Cross-sectional) | Isolated older adults engage in a 6 week and 6 session program that is self-guided program based on counselling for elderly aged 50-86 years to reduce loneliness. | | M: Virtual (Not specified)  I: Self-directed N = 239 | Positive changes to loneliness in general but no positive change to daily assessment of loneliness. |
|  | **Analyzing Relationships and Making New Friends** *(Martina CM, 2006 from Douglas NF, 2023)* | Mixed-and/or multi-method | This intervention, focused on well-being and loneliness, involved 12 sessions designed for older women aged 53-86. The program aimed to help participants analyze their relationships, develop plans to enhance existing connections, and make new friends. | | M: In-person I: Group-based N = 60 | Quantitative: Overall, there were no changes in feelings of loneliness. However, there were positive changes to the quantity and quality of friendships. |
|  | **Analyzing Relationships, Making New Friends, and Improving Relationships**  *(Martina CM, 2018 from Douglas NF, 2023)* | Mixed-and/or multi-method | This intervention targeted loneliness and was based on helping participants analyze their relationships a developing plan to make new friends and improve current relationships for older women 63 years on average. | | M: In-person I: Group-based N = 108 | Overall, there was no change in feelings of loneliness, however there was an increase in the number of friendships. |
|  | **Analyzing Social Relationships** *(Vassilev I, 2019 from Douglas NF, 2023)* | Qualitative | Older adults with a chronic disease enrolled in this intervention underwent a course that was three months long throughout 2-6 sessions that were 1.5 hours long to allow them to analyze their relationships and social networks while using resources. All of this was provided by a counsellor to reduce loneliness. | | M: In-person I: One-to-one N = 15 | Results showed that participants had deepened relationships within social networks. |
|  | **Community Socialization Intervention**  *(Rodrigues-Romero R, 2021)* | Quantitative (RCT) | The intervention aimed to promote socialization and inform available community resources for older adults age ≥65 years with moderate or severe perceived loneliness and moderate autonomy or dependence. It included 18 sessions over 6 months consisting of:(i) health promotion and disease prevention to increase quality of life and well-being; (ii) activities aimed at improving mental and emotional state (mindfulness, yoga, songs to remember, laughter therapy and relaxation); (iii) activities to create a social network among participants (neighbourhood resources, establish a common participative space (neighbour-hood kitchen, trip to the cinema, walk through the green areas of the neighbourhood, sewing workshop and cultural trip) | | M: In-person I: Group-based N = 55 | Older adults had significant improvements in loneliness and social support from the community intervention. |
|  | **Education And Social Facilitation Intervention for Loneliness**  *(Alaviani M, 2015)* | Quantitative (Quasi-experimental) | Older adult women with moderate loneliness participated in the lecture and question-answer intervention and were divided into three sub-groups. Each group attended 4 sessions (2x/week) for 60 minutes. In the first session, 2 study authors talked about the definitions, causes, clinical symptoms, and complications of loneliness; and at the end were given a summary of all contents and asked to think about the question for the next session: “when you feel lonely, what do you do?”. The goal of the 2nd session was to empower subjects to improve social relationships as an important factor in reducing loneliness. In the 3rd and 4th, their achievements were checked. | | M: In-person I: Group-based N = 120 | Loneliness decreased significantly in the interventional group compared to the control group (P<0.00). |
|  | **Education on Making Friends** *(Stevens NA, 2001 from Johnstone G, 2021)* | Mixed-and/or multi-method | The intervention aimed to support lonely women in achieving their friendship goals through a 12-week educational program delivered to groups of 8-12 women. The program was designed based on a four-stage model focusing on the impact of relational competence in various phases of relationships. The primary objective was to assess the effectiveness of the intervention in helping older women achieve their friendship-related goals. | | M: In-person I: Group-based N = 40 | Significant reduction in loneliness ( p < 0.001), which did not differ significantly between marital or living status. 70% of participants made new friends and 48% improved friendships. |
|  | **Factsheets and Manual to Address Loneliness** *(Gracia N, 2010 from Noone C, 2022)* | Qualitative | The intervention developed factsheets and a resource manual on dimensions of loneliness. | | M: In-person  I: Self-directed N = 58 | Encouraged social participation in pre-existing network, but the availability of more nuanced social support was limited. |
|  | **Friendship Enrichment Programme (FEP)** *(Owen L, 2016)* | Quantitative (Cost or economic analysis) | The FEP for older women (53–86 years) comprised 12 lessons that focused on friendship-related topics such as self-esteem | | M: In-person I: Group-based N = 115 | The FEP (friendship enrichment programme) significantly improved life satisfaction but did not improve loneliness. |
|  | **Friendship Enrichment Programme (FEP)**  *(Martina CMS, 2006)* | Quantitative (Quasi-experimental) | The FEP delivered in the community to women between 53-86 years of age consisted of 12 lessons to build self-esteem, setting goals and boundaries in friendship. | | M: In-person I: Group-based N = 115 | The FEP had mixed results: it significantly increased the quantity of friendships but non-significantly improved the quality of friendships. There were moderate improvements in subjective well-being; however, the FEP did not reduce loneliness. |
|  | **Friendship Enrichment Programme (FEP)**  *(Martina CMS, 2018)* | Quantitative (Quasi-experimental) | The FEP consisted of 12 lessons to build self-esteem, setting goals and boundaries in friendship of women. | | M: In-person I: Group-based N = 108 | The FEP intervention had mixed results in reducing loneliness: ¼ of women (including those who were severely lonely) were no longer lonely after the intervention; 1/3 experienced a significant reduction in loneliness; a minority remained lonely; a small group became lonely during the year but were not lonely at the end of the programme. |
|  | **Friendship Service** *(Martina CM, 2006 from Ibrahim AF, 2022)* | Quantitative (Quasi-experimental) | This intervention was to enrich the lives of those 55+ years with friendships whereby 12 lessons focused on how to improve relationships, self-esteem, setting goals etc. There was role-playing as well for how to deal with difficult situations in relationships. This lasted for 12 lessons over the course of 12 months. | | M: In-person I: One-to-one N = 115 | There was a significant improvement in making friends and reduced loneliness. |
|  | **Group Course to Build Social Network and Foster Current Relationships** *(Stevens NA, 2001 from Douglas NF, 2023)* | Quantitative (Cross-sectional) | A group course that is based on creating social networks and improving current social relationships for lonely women 63 years on average to help with social networks and loneliness. | | M: In-person I: Group-based  N = 40 | There were positive changes in loneliness, and in new social connections. |
|  | **Group Course to Build Social Network and Foster Current Relationships** *(Stevens NL, 2006 from Douglas NF, 2023)* | Quantitative (Cross-sectional) | A group course for lonely and non-lonely older women held for 12 weeks in 12 weekly sessions that is based on creating social networks and improving current social relationships for participants 63 years on average to help with loneliness. | | M: In-person I: Group-based  N = Study 1: 52 Study 2: 60 | There was a positive change to loneliness. |
|  | **Group Course to Build Social Network and Foster Current Relationships** *(Tilburg NS, 2000 from Douglas NF, 2023)* | Mixed-and/or multi-method | A group course held for older women for 12 weeks in 12 weekly sessions that is based on creating social networks and improving current social relationships for participants 63 years on average. | | M: In-person I: Group-based  N = 32 | There was no change to loneliness. |
|  | **Improving Community Networks** *(Saito T, 2012 from Douglas NF, 2023)* | Quantitative (RCT) | This intervention was based on preventing social isolation and improving social networks over the course of 8 weeks for 4 session each 2 hours long for older adults who recently moved to a new city 72 years on average to help with loneliness, social support, and well-being. | | M: In-person I: Group-based N = 20 | There was a positive change for loneliness and well-being. |
|  | **Lifestyle Matters Intervention**  *(Mountain G, 2017)* | Quantitative (RCT) | This intervention was based on an occupational approach to healthy ageing and designed to assist community dwelling adults age ≥65 years with emotional and social loneliness. Participants met in a weekly group of up to 12 people over 4 months at a local venue. Participants were also asked to engage in monthly individual sessions with a facilitator, who worked with participants to explore the selected topic through discussion, activities, and community enactment. | | M: In-person I: Mixed (Group-based, one-to-one) N = 288 | Emotional and social loneliness significantly improved in community dwelling older adults, although the relevance of this finding is questionable due to a lack of evidence to support a minimal clinically important difference |
|  | **Loneliness Self-Help Print**  *(Gracia N, 2010)* | Qualitative | The aim of the self-help print resource is to address loneliness and encourage social well-being in a retirement village community. It focuses on providing education about the five dimensions of loneliness. | | M: In-person I: Self-directed N = 58 | The intervention facilitated the organisation of activities and encouraged social participation. |
|  | **Multi-Component Intervention to Decrease Loneliness** *(Honigh-de Vlaming R, 2013 from Douglas NF, 2023)* | Quantitative (Quasi-experimental) | This intervention was varied in terms of session meetings to decrease loneliness and increase the knowledge of how to handle loneliness and increase social support in older participants that were 73 years on average. This intervention has components of media and face-to-face sessions which included developing social networks, lowering the standard for feelings of loneliness, and evaluating the relevance of the experience of loneliness. | | M: NR I: NR  N = 440 | Overall, there was no change to loneliness or social support. |
|  | **Social Activities Group Program** *(Nomura K, 2021)* | Mixed-and/or multi-method | Elderly males (aged 65 and older) in Japan participated in a 12-session program aimed at combating social isolation through increased engagement in social activities—the group delivery approach involved 120-minute weekly sessions comprising lectures, exercises, and experiences. Based on a management process using occupational therapy to reconstruct lifestyles, the program had participants explore issues in their environment, learn the significance of community engagement, and analyze the use of their time for social activities. | | M: In-person I: Group-based N = 20 | The program led to significant improvements in independent living , but it did not help increase satisfaction with social activities. |
|  | **Social Participation Nursing Intervention**  *(Gosline MB, 2003)* | Qualitative | The nursing intervention was designed to encourage social participation among clients by involving them in goal setting. Participants were given the task of writing down five social activities they wished to engage in. The intervention provided methods for planning, reminiscing, and looking forward to activities with enthusiasm. | | M: In-person I: One-to-one N = 2 | The intervention led to increased positive anticipation of social events and participation and enjoyment of social activities. Socialization, interaction with family and friends and life satisfaction also increased. |
|  | **The ‘School of Health for Older People’**  *(Lapena C, 2020 from Noone C, 2022)* | Qualitative | A weekly intervention called ‘School of Health for Older People.' Promotes resources to enhance participants’ ability to identify problems and activate solutions, ultimately encouraging their participation in the community. | | M: In-person I: Group-based N = 28 | The intervention improved peer relationships. |
|  | **The ‘School of Health for Older People’**  *(Lapena C, 2020)* | Qualitative | The 'School of Health for Older People' is a community-based weekly intervention targeting individuals aged 65 and older in deprived neighborhoods of Barcelona. Employing an 'asset model,' the program comprises 22 sessions focusing on biological, psychological, and social health topics. Offered to community-dwelling adults in selected neighborhoods who speak Spanish or Catalan, the free sessions are held every Wednesday morning in social centers. Led by local experts, including health and social service representatives, police, and market associations, the intervention promotes health, leisure, and social resources. The sessions aim to enhance personal self-care skills, inform about healthy habits, safety, and self-esteem, and build social networks through dynamic activities. | | M: In-person I: Group-based N = 28 | The most relevant benefits identified were that the intervention decreased perceived feelings of loneliness and social isolation, increased participants’ contacts with others, helped to increase their knowledge of other activities in the neighbourhood, enhanced the feeling of belonging, and improved health and well-being. |
|  | **The ‘School of Health for Older People’**  *(Lapena C, 2022)* | Quantitative (Quasi-experimental) | Community-dwelling individuals aged 60 and above in a low-income neighborhood participated in 22 group sessions, each lasting 1.5 hours. The sessions covered various topics (biological, social, or psychological) with the goal of enhancing quality of life and social support. Led by local experts in health, social services, markets, or neighborhood associations, the sessions aimed to encourage interaction and personal skill development among participants. | | M: In-person I: Group-based  N = 135 | The intervention helped to maintain quality of life and social support (non-significantly), which were worsened or maintained respectively in the comparison group. Self-perceived health did not change. |
|  | **The Generating Engagement in Network Support (GENIE) Tool**  *(Welch L, 2020)* | Quantitative (RCT) | This was a social networking tool to build social capacity to support self-management for older adults with chronic obstructive pulmonary disease (COPD): (i) a concentric circle modelling to map existing social networks; (ii) a questions sections to elicit preferences for activities; (iii) a map of selected resources is then produced, aligned with the user’s interests and suggestions for connections to existing network members and to new resources. | | M: In-person I: Mixed  N = 60 | 55% of participants increased social network members/size, frequency of social interactions, amount of online engagement, and engaged in additional activities. The overall quality of life showed non-significant improvements in the intervention arm. |
|  | **The I-SOCIAL Intervention**  *(Cohen-Mansfield J, 2018)* | Quantitative (RCT) | The intervention for older adults (age ≥65 years) focused on addressing psychosocial barriers (e.g., low social self-efficacy), and environmental barriers (e.g., lack of social opportunities in their vicinity). The intervention included: (1) identifying the barriers for the specific person; (2) up to ten individual meetings with an activities counsellor, which focused on helping to address personal barriers to social integration and included discussions concerning options for social contact as well as using techniques and local resources to tackle the barriers; and (3) up to 7 group sessions of participants and the activities counsellors were held to provide opportunities to increase social competence by practicing social skills within a protected setting. | | M: In-person I: Mixed (Group-based, one-to-one) N = 74 | Older adults showed a significant decline in loneliness both after the intervention and follow-up period. The number of group sessions attended predicted the decrease in loneliness rates after the intervention. It may also reflect the fact that those who attended group sessions were more ready socially to enhance their social activities. The number of individual meetings was not predictive of improvement in loneliness, as it reflected different underlying processes. |
|  | **The Social Isolation Prevention Intervention Program**  *(Saito T, 2012)* | Quantitative (RCT) | Intervention aimed to prevent social isolation in older adults by improving community knowledge and networking with other participants and community “gatekeepers.” | | M: In-person I: Group-based N = 60 | Older adults had significantly reduced loneliness, improved social support, familiarity with services in the community and subjective well-being. There were also non-significant improvements in social activity. |
|  | **The VRCHIVE Project** *(Appel L, 2022 from Li M, 2023)* | Qualitative | A community sample of older adults were part of the VRCHIVE project which aimed to target literacy skills, self-expression, relationship building, and a sense of community. The sessions were for 4 weeks, each week for 1 hour. | | M: Virtual (Not Specified) I: NR  N = 5 | The workshop allowed participants to feel less isolated and have greater connections with other people. |
|  | **Virtual Coaching** *(Brandenburgh A, 2014 from Rivera-Torres S, 2021 )* | Quantitative (Cross-sectional) | This intervention, utilizing virtual coaching, involved participants aged 65 and above using a tablet or computer within their homes. The primary goal was to enhance social communication. This application provided friendship enrichment lessons, encouraging older adults to make new friends through tasks such as "going for a walk with someone." | | M: Virtual (Computer) I: NR  N = 7 | All involved in the study had lower loneliness scores except one participant. |
|  | **General health training interventions (n = 23)** | | | | | |
|  | **A General Well-being Training Intervention**  *(Bartholomaeus JD, 2019)* | Quantitative (Controlled before and after) | A multi-component program comprising a set of techniques designed to increase one’s sense of well-being and resilience within two distinct populations: non-clinical older adults from the general community (population group 1) and older unpaid carers of dependent people with a disability, mental illness, or a chronic health condition (population group 2). The intervention was delivered by trained community staff and involved an 8-week face-to-face well-being training program, delivered in groups, one session each week: duration range 90-120 minutes/session. Each session taught the participants one of ten evidence-based skills to improve their well-being and resilience. Post-training, participants could access mentoring and peer-to-peer support to implement and practice the ten skills in their lives; they could also access monthly support groups to discuss well-being and resilience goals. | | M: In-person I: Group-based N = 58 | Results were mixed among the two population groups. Older adults from the general population (population group 1) had significantly lower social isolation scores, but no significant difference in well-being, or resilience. Older adult carers (population group 2) showed no significantly higher scores for social isolation, but significantly higher scores for well-being, and resilience. |
|  | **Chronic Disease Self-Management Education (CDSME) Programs** *(Smith ML, 2023)* | Quantitative (Quasi-experimental) | This intervention utilized the evidence-based Chronic Disease Self-Management Education (CDSME) program, targeting older adults with chronic diseases. The 6-week workshop series aimed to reduce loneliness through peer interactions, defining completion as attending 4-6 sessions. CDSME programs, including the leading Chronic Disease Self-Management Program (CDSMP), offer valuable skills for managing various chronic health conditions. These programs focus on universal concepts such as problem solving, goal setting, and action planning, fostering peer support and idea-sharing among participants. | | M: In-person I: Group-based  N = 295 | There was a significant decrease in loneliness from baseline to the 6-week follow-up (p<0.001). |
|  | **Education About Assistive Devices**  *(de Craen AJM, 2006 from Cohen-Mansfield J, 2015)* | Quantitative (RCT) | Educational intervention involving visits from an occupational therapist who provided training and education about assistive devices that were already present and who gave recommendations and information about procedures, possibilities, and costs of assistive devices and community-based services. | | M: In-person I: One-to-one N = 402 | At 2-yr follow-up, there was no difference between study groups in loneliness. |
|  | **Geriatric Rehabilitation Program** *(Ollonqvist K, 2008 from Ibrahim AF, 2022)* | Quantitative (RCT) | This program was for community-dwelling elderly that allows for groups lectures on topics of life and medical information for a length of 12 months. This program was intended for those 65+ years and older to help with emotional loneliness. | | M: In-person I: Group-based N = 708 | There was a decrease in the feelings of loneliness, but it was not significant. There was also a decrease in the number of friends. |
|  | **Health Aides** *(Anderson L, 1985 from Ibrahim AF, 2022)* | Quantitative (Quasi-experimental) | This intervention was led by health aides who led discussions on health topics like leisure activities. The aim was to strengthen local networks for those 60+ years. This intervention lasted 2 months. | | M: In-person I: Group-based N = 108 | There was no significant improvement in loneliness; however, there was significant improvements in social contact. |
|  | **Health and Social Provision Interventions** (*de Craen AJM, 2006; Granbom M, 2017; Melin AL, 1993; Morrow-Howell N, 1998; Taube E, 2018; Thomas KS, 2016 from Chau CMS, 2023)* | Quantitative (RCT) | The review included health and social care provision interventions consisting of education, training, case management, treatment plans, assessments, service management, meal delivery and supportive therapy*. **Some intervention types included in this review may also fall into other domains; however, this classification represents the majority.* | | M: Mixed (In-person and virtual (telephone)) I: One-to-one N = NR | Social engagement (2 studies, 244 participants) significantly improved, whereas there was no significant change in loneliness (1 study, 244 studies). |
|  | **Health Education and Activities** *(Rodríguez‐Romero R, 2021 from Douglas NF, 2023)* | Quantitative (RCT) | This intervention was for lonely and/or depressed adults that was based on a course which included material on health education and social group activities conducted by nurses in 18 sessions over the course of 6 months for participants 80 years on average. | | M: In-person I: Group-based N = 55 | Overall, there was a positive change for loneliness and social support. |
|  | **Health Promotion Interventions** *(de Craen AJM, 2006; Franse CB, 2018; Gustafsson S, 2017; Taube E, 2017; van Rossum E; 1993 from Yu DS, 2023)* | Quantitative (RCT, N-RCT) | This systematic review compared the effects of various non-pharmacological interventions, including five health promotion interventions to promote healthy aging and improve physical and psychosocial risk factors associated with loneliness in community-dwelling older adults. | | M: NR I: NR  N = NR | Data pooling for loneliness on four studies with computed SMD showed significant effect favoring the control interventions (n = 4; SMD = 0.12; 95%CI [0.00, 0.24]; Z =2.01, p = 0.04; I2: 18%, τ2: 0.0025; p = 0.30). |
|  | **Healthy Ageing Intervention**  *(Honigh-de Vlaming R, 2013)* | Quantitative (Quasi-experimental) | Five intervention components were incorporated: a mass media campaign, information meetings for interested local elderly people, psychosocial group courses for persons with mental health problems or chronic diseases, social activation by the community-based Neighbours Connected intervention, and training of intermediaries (homecare nurses, municipal advisors, and volunteers) across 11 weeks. The psychosocial courses were directed to elderly people with mild depressive symptoms and chronic diseases and focused on the development of coping and communication skills. The group courses, consisted of 8-10 meetings. Five intervention components were incorporated: a mass media campaign, information meetings for interested local elderly people, psychosocial group courses for persons with mental health problems or chronic diseases, social activation by the community-based Neighbours Connected intervention, and training of intermediaries (homecare nurses, municipal advisors, and volunteers) across 11 weeks. The psychosocial courses were directed to elderly people with mild depressive symptoms and chronic diseases and focused on the development of coping and communication skills. The group courses, consisted of 8-10 meetings. | | M: In-person I: Group-based N = 1804 | No overall effects were observed for social support, and loneliness in the long term. |
|  | **I'd Rather Stay 19-Minute Documentary Video**  *(Ottoni CA, 2020)* | Qualitative | The intervention provided education, and aimed to encourage, and activate older people age ≥65 years around issues such as independence, physical activity, and social connectedness. | | M: In-person I: Group-based N = 48 | Social connections were promoted. |
|  | **Lifestyle Redesign Intervention**  *(Juang C, 2018)* | Quantitative (RCT) | The intervention for older adults (age ≥60 years) includes didactic presentation, peer exchanges, direct experience, and personal reflection to enable participants to explore activity as it relates to healthy habit formation, routines, and overall wellness. The program is led by occupational therapists who are trained in the administration of the intervention prior to providing treatment. Weekly small (8–10 participants) group sessions and up to 10 individual sessions were held. | | M: In-person I: Group-based N = 460 | Older adults had significantly increased meaningful activity frequency, and improved perception of activity significance. Older adults showed significant increases for participating in community classes, creative activities, shopping talking on the phone as well as socializing with friends and family. Changes in perceived control, and social connections were non-significant. |
|  | **Mental Health-Informed Lifestyle Program** (McKeon G, 2022) | Quantitative (Quasi-experimental) | Older adults (aged ≥ 60) were recruited into a 6-week program delivered via a private Facebook group. Facilitators provided motivation and education on weekly topics, including goal setting and reducing sedentary behaviour through Facebook posts and group video calls. The program was assessed for quality of life and loneliness. | | M: Virtual (Computer) I: Group-based N = 11 | Exploratory analyses of all pre-post outcomes showed evidence of non-significant improvements in quality of life and loneliness. |
|  | **Persian Diabetes Self-Management Education (PDSME)** *(Saghaee A, 2020)* | Quantitative (RCT) | Participants (≥ 60 years) with type 2 diabetes participated in an evidence-based diabetes self-management education program in Iran. The intervention involved culturally oriented diabetes self-management education program comprising eight 2-hour workshops over four weeks. Led by a diverse team, including an occupational therapist, nurse educator, dietitian, and general practitioner, the program addressed nutrition misconceptions, emphasizing moderation. It integrated the ADAE's seven core aspects of diabetes self-management, promoting active participation and goal setting. | | M: In-person I: Group-based N = 34 | Quality of life increased significantly (p ˂ 0.05). However, there were no significant differences in loneliness or self-efficacy. |
|  | **Self-Care Reinforcement Program (SCRP)**  *(Park M, 2019)* | Quantitative (Quasi-experimental) | The program was designed to promote successful aging of socially vulnerable elderly females aged ≥65 years with more than 3 risk factors for metabolic syndrome by improving physical performance through balanced nutritional intake and to improve socio-psychological function by providing a social network to aid recovery from depressive mood (i.e., well-balanced nutritional intakes, regular exercise, self-help meetings, and community connection projects); 3x/week for 8 weeks (total of 24 sessions). | | M: In-person I: Group-based N = 64 | Older women had significant improvements on their social network quality. |
|  | **Senior CAN Educational Intervention**  *(Collins CC, 2006 from Cohen-Mansfield J, 2015)* | Quantitative (Cross-sectional) | The intervention for community dwelling older adults includes 15 lessons on topics including nutrition and food, personal safety, financial strategies to manage with limited resources, general wellness, and productive aging. The lessons were provided weekly over four months by paraprofessionals, volunteer peer educators and on-site staff, resulting in an average of 32-h of education/participant. Instructors emphasized how information could be readily applied and integrating one new idea or skill from each lesson aimed to increase mastery. | | M: In-person I: Group-based N = 339 | Older adults had significantly reduced loneliness. The greatest drop in loneliness was seen in low-income ethnic minorities and minorities with high levels of education. |
|  | **Successful Aging** *(Kocken PL, 2998 from Ibrahim AF, 2022)* | Quantitative (Quasi-experimental) | This intervention was based on health education whereby the sessions were led to discuss sleep, exercise, memory, medicine etc. The 4 sessions were 2 hours long and aimed to increase social support for those 55+ years. | | M: In-person I: Group-based N = 320 | There was no significant impact on social participation. |
|  | **Thanks, Sorry, Love, and Farewell Board Game** *(Chen M, 2022)* | Quantitative (Quasi-experimental) | "Four Themes of Life" board game intervention to enhance interpersonal communication, relationships, and self-efficacy and decrease loneliness in older adults (≥ 65 years). The game was designed to educate participants on incorporating the game's themes (thanks, sorry, love and farewell) into their lives. Participants played weekly for 90 minutes at a community centre over four weeks. | | M: In-person I: Group-based  N = 91 | The experimental group had significant improvements in interpersonal communication, self-efficacy, and loneliness three months after the end of the intervention. |
|  | **The Resident Wellness Coaching Program** *(Fullen MC, 2023)* | Quantitative (Quasi-experimental) | The nine-week intervention targeted elderly residents aged 71-97 in senior living communities, involving 20-30 minutes of program-related activities to achieve wellness goals. Instructors facilitated both group and individual sessions in a staff-led wellness coaching program to reduce loneliness through resident-driven goals. The program recommended a holistic wellness framework, a structured weekly schedule, and coaching sessions for optimal effectiveness. Coaches, selected from wellness-related roles, underwent 42 hours of standardized training. Participants were encouraged to spend daily time on program-related activities and received a workbook. The program concluded with a group reflection and individualized plans to maintain wellness changes. | | M: Mixed (In-person and virtual (computer, paper)) I: Mixed (Group-based, self-directed) N = 79 | Overall, there was no significant difference in quality of life across time. However, loneliness significantly decreased post-program (p= 0.001) and at the follow-up (p= 0.035), and relatedness (characterized by warm and caring relationships) significantly improved over time (p=0.018). |
|  | **The Virtual Learning Program**  *(Botner E, 2018)* | Qualitative | The Program offers live lectures and events from content experts, on-demand video library, and peer-to-peer and intergenerational discussion groups for free during the grant period. Participants can get help with technology through home visits and telephone support from trained volunteers. | | M: Virtual (Computer, tablet) I: Group-based N = 116 | Qualitative: Participant’s reported feeling less isolated, higher social connection, with the benefit of not needing to leave home. The discussion groups showed promise in reducing social isolation among participants. |
|  | **Video-Conferencing Program for Skill Development** *(Yavuz C, 2023)* | Quantitative (Cross-sectional) | This intervention, targeting individuals aged 60 and above, aimed to enhance psychosocial health during COVID-19 through an 8-week program. The curriculum included Sports (taichi, qigong, yoga, and Pilates), Skill Development (bottle and stone painting, crafts such as knitting and bag sewing, quilling works), and Interactive Social Sciences courses. Each course lasted approximately 45–60 minutes. Experts led the Sports and Skills courses, while the Social Sciences course covered diverse topics through interactions with faculty members, authors, drama educators, and artists. | | M: Virtual (Computer, smartphone) I: Group-based N = 92 | Overall, there was a significant difference in a decrease in loneliness for the experimental group compared to the control group post-test (p<0.05). |
|  | **Walk 'n Talk for your life** *(Hwang J, 2019 from Noone C, 2022)* | Qualitative | The intervention aimed to promote socialisation, health education, and falls prevention exercise. | | M: In-person  I: Group-based N = 16 | The intervention provided a sense of belonging, which appeared to be mediated by the group exercise/walking component of the programme. |
|  | **WESIHAT 2.0©** *(Vanoh D, 2019 from Heins P, 2021)* | Quantitative (RCT) | The intervention examined was WESIHAT 2.0©, a web-based wellness application, used for 6 months in combination with group counselling sessions in the first 3 months. | | M: Virtual (Smartphone) I: NR  N = 60 | No statistically significant interaction effects for loneliness and other dimensions of social support. |
|  | **Workshops for Health Resources** *(Lapena C, 2020 from Douglas NF, 2023)* | Qualitative | Over a span of 22 weeks, providers conducted community workshops and teachings on healthcare and resources in 1.5-hour sessions for participants at risk of loneliness in neighborhoods with low socio-economic status (age unspecified). Participants took part in structured activities facilitated by nurse coordinators and expert speakers at local community centers. | | M: In-person I: Group-based N = 26 | Overall, there were positive changes to social isolation. |
|  | **Combination of technology device and Internet training (n = 21)** | | | | | |
|  | **Computer and Internet Training Provided by Volunteers**  *(Jones RB, 2015 from Poscia A, 2018)* | Quantitative (Cross-sectional) | Older adults (age ≥65 years) who participated in the group-based computer and internet training received help in using the Internet from 32 volunteers one-on-one in their own homes, receiving an average of 12 hours of help over eight visits. The intervention included individual sessions that covered basic computer use, how to get online and search the Internet, online shopping, email, Skype or FaceTime, and online news and entertainment. | | M: In-person I: Mixed (Group-based, one-to-one) N = 144 | Older adults in the group-based training had significantly improved loneliness, social isolation, and quality of life. One-to-one computer-based training led to significant improvements in social isolation, but loneliness had non-significant changes. |
|  | **Computer and Internet Training with Loaned PC**  *(Slegers K, 2008 from Chen YRR, 2016)* | Quantitative (RCT) | The intervention participants were loaned a personal computer (PC) with Internet access and completed Internet-related tasks. | | M: In-person I: Group-based N = 236 | The results showed that using computers and the Internet neither positively nor negatively influenced loneliness, everyday functioning, well-being, and the social network of healthy older individuals. |
|  | **Computer Education** *(Blažun, 2012 from Douglas NF, 2023)* | Quantitative (Cross-sectional) | Participants meet once weekly in 4-hour sessions (Finland) and in 3-hour session (Solvenia) over the course of 3 weeks where they are taught to use computers to decrease social isolation and increase social support. | | M: In-person I: Group-based  N = 45 | There was a decrease in loneliness. |
|  | **Computer Training Program** *(Kim J, 2016 from Heins P, 2021)* | Qualitative | The intervention trained older adults on the use of computer and included training session to enhance computer and internet skills. | | M: NR I: NR  N = 11 | Benefits included enhanced social connectedness. |
|  | **Computer Training Program** *(Slegers K, 2008 from Heins P, 2021)* | Quantitative (Clinical controlled trial) | The intervention examined was a computer training program with three 4-hour training sessions for 2 weeks, independent use of the computer combined with assignments. | | M: NR I: NR  N = 236 | No significant positive (or negative) intervention effect on social well-being. |
|  | **Computer/Internet Training Program** *(Woodward AT, 2011 from Heins P, 2021)* | Quantitative (Clinical controlled trial) | The intervention examined involved 11 computer and internet training sessions in a group delivered by the project coordinator for 6 months. | | M: NR I: NR  N = 83 | No statistically significant differences in social support and loneliness between the groups. There was a trend of higher perceived social support in the intervention group vs. the control group. |
|  | **Esc@pe Program**  *(Fokkema T, 2007 from Cohen-Mansfield J, 2015)* | Quantitative (Quasi-experimental) | Seniors were loaned desktop computers and related equipment and received lessons on how to e-mail and how to use the Internet and supported and coached by visiting volunteers. | | M: In-person I: One-to-one N = 15 | The difference between the intervention and control groups for reducing loneliness was significant. |
|  | **How to Use Internet and Computer** *(Fokkema T, 2007 from Douglas NF, 2023)* | Mixed-and/or multi-method | This intervention was over three years in 5 sessions (2 hours per session) where participants were taught by volunteers (with support visits every 2-3 weeks) how to use the internet and computer for those who are homebound and physically disabled aged 66 years on average. | | M: Virtual (Computer, smartphone, tablet) I: One-to-one N = 12 | Overall, there were positive changes to loneliness. |
|  | **How to Use the Internet** *(White H, 2002 from Ibrahim AF, 2022)* | Quantitative (RCT) | This intervention involved a trainer who trained elderly participants for a total of 9 hours on how to use a computer (email and using the web). The aim was to decrease loneliness. | | M: Virtual (Computer) I: One-to-one N = 100 | There was no significant effect on loneliness. |
|  | **Internet at Home - Esc@pe** *(Fokkema T, 2007 from Johnstone G, 2021)* | Mixed-and/or multi-method | The study aimed to evaluate an internet-at-home intervention named Esc@pe, designed to reduce loneliness among chronically ill and physically handicapped older adults by introducing them to electronic communication. Participants were provided with loaned PCs and peripheral equipment for three years. Additionally, they received five two-hour lessons on email and internet use to enhance their digital communication skills. Support was offered every 2-3 weeks through home volunteer visitors, providing assistance and guidance. The intervention not only aimed to decrease loneliness but also fostered ongoing PC help and maintenance. | | M: NR I: NR  N = 26 | Quantitative: Overall and emotional loneliness significantly decreased. However, social loneliness did not.  Qualitative: Internet allowed connection despite poor health. |
|  | **Internet Information Station** *(Mullins LB, 2020 from Heins P, 2021)* | Mixed-and/or multi-method | The intervention examined the Internet Information Station program that provided three different computer classes delivered by students. | | M: In-person I: Group-based N = 262 | Qualitative: Participants reported enhanced social connectedness.  Quantitative: There were non-significant decreases in loneliness. |
|  | **Internet Information Station (ISS) With Computer and Internet Training**  *(Mullins LB, 2020)* | Mixed-and/or multi-method | The community created ISS to provide computer and internet training to residents. The station had two computers with enlarged screens, and students from a university service-learning program were recruited to teach technology classes. The course included three lessons on computer basics, internet basics, and social media. The course was modified to include more time for small group practice and answering residents’ device-related questions. | | M: In-person I: Group-based N = 36 | Quantitative results: Non-significant decreases in loneliness and increased interactions among residents. Qualitative results: Providing computers with internet access in common areas encouraged residents to leave their apartment. |
|  | **PC Computer and Internet Training  with Online Discussion Forum**  *(Torp S, 2007 from Chen YRR, 2016)* | Mixed-and/or multi-method | PC with internet and online discussion forum, three 3-hour group training sessions over 3 weeks for informal carers (60 years of age and older) of frail elderly people living at home. | | M: In-person I: Group-based N = NR | Quantitative results: ICT use significantly increased social contacts (mean change 1.5; CI 0.06-2.88; p = 0.04) and social support (mean change 3.4, CI 1.14-5.61; p = 0.010) for carers.  Qualitative results: ICT use led to facilitated contacts with grandchildren, gaining new and supportive friends with same experience for carers. |
|  | **Skill Development Interventions** *(Czaja SJ, 2018; Fields J, 2019 from Chau CMS, 2023)* | Quantitative (RCT) | This review includes interventions based on skill development interventions including training on how to use information and communication technologies. | | M: Mixed (In-person and virtual (telephone, tablet) I: NR  N = NR | Loneliness (1 study, 244 participants) and social support (1 study, 244 participants) significantly changed. |
|  | **Technology and Internet Training**  *(Bornemann R, 2014 from Chipps J, 2017)* | Quantitative (Meta-analysis) | Older adults aged 71-82 years receiving computer and internet training. | | M: NR I: NR  N = NR | There was a non-significant decrease in loneliness. |
|  | **Using Communication Technology** *(Blažun H, 2012 from Casanova G, 2021)* | Quantitative | Computer training for those 58-93 years over the course of 3 weeks with 4 hours of training. | | M: Virtual (Computer) I: NR  N = 58 | There was a significant decrease in loneliness (specifically among women). |
|  | **Using Communication Technology** *(Cotten SR, 2012 from Casanova G, 2021)* | Quantitative (RCT) | Computer and internet training for those 82.7 years on average over the course of 8 weeks. | | M: Virtual (Computer) I: NR  N = 205 | Weak beneficial impact on loneliness. |
|  | **Using Communication Technology** *(Morton TA, 2018 from Casanova G, 2021)* | Quantitative (RCT) | Computer and internet training for those 60-95 years. Was 12 weeks long and had 18 hours of training. | | M: Virtual (Computer) I: NR  N = 97 | No significant effect on loneliness. |
|  | **Using Communication Technology** *(Slegers K, 2008 from Casanova G, 2021)* | Quantitative (RCT) | Computer use and how to use the internet intervention for those 64-75 years. The intervention was 54 weeks long and 4 hours of training. | | M: Virtual (Computer) I: NR  N = 236 | No significant differences were observed between groups and between times of follow-ups for loneliness. |
|  | **Internet social networking website (ISNW) 'About My Age'**  *(Ballantyne A, 2010)* | Qualitative | The intervention was tested with older adults (age 69-85 years) from a community aged care program. The participants were connected to the internet and given individualized tutoring on how to use the unique social networking site. | | M: Virtual (Computer) I: Mixed (Self-directed, one-to-one) N = 4 | The intervention reduced loneliness, specifically temporal loneliness, and connectedness in older adults. Participants developed new innovative ways of linking with people in the community, online and in person. |
|  | **Facebook Training**  *(Myhre JW, 2017)* | Quantitative (Quasi-experimental) | The Facebook training taught adults age >50 years how to use Facebook; they attended six 2-hr classes over 2 weeks, followed by 6 weeks of continued use at home. It also involved computer skills, and the use of the Facebook Starter Kit. Led by an instructor and several tutors in a computer lab classroom. Participants completed homework assignments between classes and the instructor monitored progress. | | M: Mixed (In-person and virtual (not specified)) I: Mixed (Group-based, self-directed) N = 41 | During the 8-week intervention period, new relationships were formed and maintained among participants in the Facebook group. Several people used Facebook to arrange face-to-face meetings and reported that they planned to continue those friendships after the conclusion of the study. Those in the Facebook group were therefore truly engaged in new social interactions during the study. However, there were no changes in feelings of social support, loneliness, or access to social support and integration. |
|  | **Technology device training (n = 16)** | | | | | |
|  | **AGE-ON Tablet Training Program** *(Neil-Sztramko SE, 2020 from Heins P, 2021)* | Quantitative (Observational) | The intervention examined was AGE-ON, that included 2-hour education sessions for 6 weeks and use of an iPad and internet at home. | | M: In-person I: NR  N = 32 | No significant differences in social isolation or loneliness. |
|  | **AGE-ON Tablet Training Program** *(Neil-Sztramko SE, 2020)* | Quantitative (Quasi-experimental) | AGE-ON tablet training explored the program's impact on social isolation, loneliness, and quality of life. Adults aged >60 years took part in a series of 6 weekly workshops covering the basic features of a tablet computer. | | M: In-person I: Group-based N = 32 | No differences in social isolation, loneliness, social support, or quality of life after completing the program were found. |
|  | **CATCH-ON Connect** *(Wang S, 2023)* | Quantitative (Quasi-experimental) | The CATCH-ON Connect program paired adults aged 55 and older with a technical support specialist for training on basic tablet use. Technical specialists provided up to 5 hours of training so that participants could do things that mattered most to them on any subject they requested, such as accessing their electronic health record. The impact of the program on social isolation and loneliness was assessed. | | M: Virtual (Tablet) I: One-to-one N = 129 | Loneliness improved significantly over time, while social isolation remained relatively stable across assessments. |
|  | **Cyber-Seniors Program** *(Breck BM, 2018 from Heins P, 2021)* | Qualitative | The intervention examined technology training lessons delivered weekly by young adult mentors (i.e., reverse mentoring) to seniors. | | M: NR I: NR  N = Older adults = 29 Mentors = 28 | Intergenerational engagement and connections emerged. |
|  | **Intergenerational Mentor-Up** *(Lee OE, 2019 from Heins P, 2021)* | Mixed-and/or multi-method | The intervention examined 6 technology tutorial sessions delivered by college students to older adults. | | M: NR I: Mixed (Group-based, one-to-one) N = 59 | Social isolation and loneliness significantly decreased (p < 0.001), but there were no significant changes in social support. |
|  | **iPad Training**  *(Burmeister OK, 2016 from Heins P, 2021)* | Qualitative | The intervention examined an iPad training program that included 2-hour training sessions in groups delivered weekly for 4 months. | | M: NR I: Group-based N = 6 | Benefits included increased social connectedness and improved life satisfaction. |
|  | **iPad Training**  *(Burmeister OK, 2016)* | Qualitative | A seniors citizen's club held weekly training sessions for four months, led by an experienced retired computer teacher. The program focused on using iPads. Participants attended the sessions which lasted up to two hours. After initial training, iPads were lent to participants who had been trained to use them for four months. | | M: In-person I: Group-based N = 6 | Findings indicated that ICT-based social interaction can enhance social connectedness and increase social activity. |
|  | **iPad Training Program** *(Arthanat S, 2016 from Heins P, 2021)* | Mixed-and/or multi-method | The intervention examined one-on-one training delivered by a coach for 3 months. | | M: NR I: NR  N = 13 | Quantitative: There were non- significant positive increases in activities involving social connections. |
|  | **iPad/iPhone Training Program** *(Emas S, 2018 from Heins P, 2021)* | Mixed-and/or multi-method | The intervention examined iPad/iPhone training in groups for 7 weeks. | | M: NR I: Group-based N = 25 | Qualitative: Participants reported having gained skills and knowledge in communicating with loved ones using concepts such as FaceTime, texts, e-mails, and phone calls. |
|  | **iPads and Technology Training**  *(Delello JA, 2017 from Chen YRR, 2016)* | Quantitative (Observational) | The intervention involved using an iPad, 3 group training of 1.5-hour lessons over 6 weeks for elderly people. | | M: In-person I: Group-based N = NR | iPad training fostered the social connection with family, online community, and existing friends of elderly people. |
|  | **Personal Reminder Information and Social Management (PRISM) System**  *(Czaja SJ, 2018)* | Quantitative (RCT) | Special software (PRISM) for seniors age ≥65 years living alone in independent housing to support social connectivity, memory, and leisure activities. | | M: Virtual (Smartphone) I: Self-directed N = 200 | Individuals had significantly improved loneliness, perceived social support, well-being and decreased social isolation. |
|  | **Project Wire Up** *(Ngiam NH, 2022)* | Quantitative (Quasi-experimental) | Project Wire Up, a digital literacy program for community-dwelling older adults aged 55+, involved volunteers providing 6 one-on-one sessions lasting 1-2 hours over 3 months. The aim was to teach smartphone usage (calling, texting, watching videos) to enhance social connections, reduce loneliness, and improve quality of life. The program included providing smartphones and internet access, tailored training, and connecting older adults to existing social networks. The training covered basic to advanced smartphone functions based on participants' needs, aiming to integrate older adults into formal and informal networks through mobile communication apps. | | M: Mixed (In-person and virtual (smartphone)) I: One-to-one  N = 138 | There was no statistically significant difference loneliness, social connectedness, personal well-being, or quality of life. |
|  | **Tablet Training** (Kim S, 2022) | Qualitative | Participants 65 to 80 years took part in using a tablet for 16 weeks, they were told to freely use a tablet as much or as little as they wanted throughout the study period. Over in-person interview sessions participants were taught how to use the tablet for its various features. | | M: In-person  I: One-to-one N = 10 | Sharing digital data gave older adults a sense of being connected to and engaging with others. |
|  | **Tablet Training**  *(Fields J, 2021)* | Mixed-and/or multi-method | Training program received 1:1 digital training session for eight weeks. The program was incorporated into Little Brothers – Friends of the Elderly's friendly visitor program, with volunteers trained as technology instructors before the in-home sessions. Participants were provided with a tablet, case, stylus, internet access, and a certificate of completion. Internet connection was paid for the duration of the program, and participants were allowed to keep their tablet, case, and stylus after completing the program. | | M: In-person I: One-to-one N = 83 | Quantitative results: The intervention led to no changes in loneliness but a non-significant improvement in perceived social support.  Qualitative results: Participants felt more connected to the world. |
|  | **Tech Clubs** *(Cutler C, 2016 from Heins P, 2021)* | Qualitative | The intervention examined digital gaming training with 2-hour training sessions delivered by facilitators for 6-8 weeks. | | M: In-person I: Group-based N = 29 | Impact of digital gaming on healthy aging included the promotion of social interaction. |
|  | **The Café-Multimedia Psychosocial Intervention**  *(Damne S, 2017)* | Mixed-and/or multi-method | The intervention included two psychologists training frail older adults to use tablets and smartphones in two-hour sessions per week for three months. The program aimed to improve social links and technology acceptance parameters. | | M: In-person I: One-to-one N = 13 | Quantitative: The intervention led to no changes in loneliness or perceived social support.  Qualitative: It provided positive engagement for participants and facilitated everyday activities, and positive social contact with other participants. However, some participant’s felt frustrated for not being to make real friendships. |
|  | **Caregiver support education interventions (n = 7)** | | | | | |
|  | **Assisting Carers Using Telematic Interventions To Meet Older People's Needs (ACTION)** *(Savolainen L, 2008)* | Qualitative | The ACTION Project aims to use ICT to support frail elderly people and their family carers at home, improving their independence and quality of life through a combination of videophone and multimedia information database services. A call center with experienced nursing staff acts as the hub of the network, providing support and practical advice to families. The key idea is that both information services and communication services are provided at the same time. | | M: Virtual (Computer) I: Pair-based  N = 8 | Participants made new friends and reported feeling less isolated. There were also positive impacts on participant’s quality of life. |
|  | **Caregiver Social Support Intervention** *(Christie HL, 2022 from Mao W, 2023)* | Quantitative (Randomized waitlist control) | This 16-week intervention, known as Inlife, was designed to enhance social support for caregivers of persons with dementia (PWD) and promote positive interactions within informal support networks. It utilized a web-based multicomponent approach to provide psychoeducational support. The development of Inlife closely adhered to the iterative Medical Research Council framework, emphasizing co-creation with potential users. | | M: Virtual (Computer) I: NR  N = 96 | There were no significant changes to social support, loneliness, or quality of life. |
|  | **Caregiving Education Intervention**  *(Cox EO, 2007 from Cohen-Mansfield J, 2015)* | Quantitative (Quasi-experimental) | The educational interventions had a psychosocial focus for older adult care recipients (age ≥55 years), it focused on the caregiving relationship, and used one-to-one and group intervention modalities. | | M: In-person I: Mixed (Self-directed, one-to-one) N = 177 | Older adult care recipients had significantly reduced loneliness and the intervention was considered effective for both one-to-one and group modalities. |
|  | **Dementia-Comprehensive Health Enhancement Support System (D-CHESS)**  *(Gustafson Jr DH, 2019 from Mao W, 2023)* | Quantitative (RCT) | This 6-month intervention involved a computer-based program called D-CHESS for caregivers of persons with dementia (PWD). The D-CHESS system enabled users to access information, engage with other caregivers, seek assistance for care decisions, and share information with experts. In contrast, the control groups received a caregiving book only. | | M: Virtual (Computer) I: NR  N = 31 | There was decreased loneliness in the intervention group but was not statistically significant. |
|  | **Engage Coaching for Caregivers**  *(Van Orden KA, 2023 from Mao W, 2023)* | Quantitative (Cross-sectional) | This intervention was for participants 61 years on average and was a 3-month program based on psychotherapy that allows participants to identify barriers to an action plan and challenges when put in a rewarding activity. | | M: Virtual (Computer) I: NR  N = 30 | There were improvements in loneliness, and perceived social isolation. |
|  | **Engage Coaching for Caregivers**  *(Van Orden KA, 2023)* | Quantitative (Quasi-experimental) | The Engage Coaching for Caregivers intervention targets caregivers aged 50 and above, aiming to increase awareness of social connection and motivation to overcome barriers to engagement. It comprises 8 weekly 30-minute sessions, including coaching and therapeutic activities, with a 3-month follow-up. The study adapts Engage Psychotherapy, emphasizing personalized action plans and addressing loneliness contributors and is based on 'social reward exposure. Engage Coaches, include clinical psychologists and graduate students. | | M: Virtual (Computer, telephone) I: One-to-one N = 30 | Loneliness, relationship satisfaction, social isolation and quality of life significantly improved over time. |
|  | **Psychoeducational Caregiver Program** *(Cristancho-Lacroix V, 2015 from Mao W, 2023)* | Mixed-and/or multi-method | The intervention included a 3-month psychoeducational program for caregivers to improve social support and increase networks and interactions. | | M: Virtual (Computer) I: NR  N = 49 | There were no significant changes to social isolation. |
|  | **Peer-based self-management education interventions (n = 5)** | | | | | |
|  | **Engaged4Life Program** *(Matz-Costa C, 2018 from Heins P, 2021)* | Quantitative (RCT) | The intervention examined the Engaged4Life program that included technology-assisted self-monitoring of physical activity for 8 weeks, a 3-hour psycho-education group session, and phone calls by peer mentors for 2.5 weeks. | | M: Virtual (Not specified) I: NR  N = 30 | There were no significant changes in the social interactions between the intervention group and the comparison group. |
|  | **Peer Counseling**  *(Carandang RR, 2020)* | Quantitative (Quasi-experimental) | Older adult peer counsellor volunteers conducted weekly one-hour home visits for three months with Filipino clients at risk of depression they were assigned. These peer counsellors, who underwent a 40-hour leadership and peer counselling training, aimed to establish a strong working alliance, identify client-defined problems, encourage behaviour change, and facilitate community engagement. The initial visit involved establishing a client-identified goal, which they worked on together. | | M: In-person I: One-to-one N = 133 | Loneliness improved; however, not significantly. Perceived social support improved significantly. |
|  | **Peer Education intervention** *(Oetzel JG, 2020)* | Mixed-and/or multi-method | The intervention involved a peer education model called tuakana-teina, where older individuals (tuakana) engaged in conversations with up to six younger recipients (teina), providing information on health and social services. The program, framed as an "orientation," focused on Māori values, principles, communication skills, and support types. It included a resource basket of health services. The tuakana attended a four-session orientation program, with subsequent conversations occurring over 12-16 weeks. The teina were matched to tuakana of the same sex. | | M: NR I: One-to-one  N = 180 | Quantitative: Health-related quality of life, desired support, loneliness, and life satisfaction improved significantly over time. In addition, perceived support increased significantly. Qualitative: Independence, social connectedness, and access to information (about services and information that can make a difference) improved. |
|  | **Peer Educators** *(Simpson ML, 2021)* | Mixed-and/or multi-method | This 12-week intervention focused on co-developing a peer education program to enhance communication among Māori kaumātua, serving as peer educators to support social connectedness and well-being. The peer educators engaged with kaumātua (teina) facing life transitions, assisting them with social and health outcomes. | | M: In-person I: Group-based  N = 26 | Quantitative: Three measures increased significantly from the baseline to the final period for tuakana, including: self-rated health (p = .05), and health-related quality of life (p = .04). Qualitative: The qualitative analysis supported the benefits of the peer educator role for older Māori including enhanced sense of well-being, and social connectedness. |
|  | **Samsam Language Café**  *(Ten Bruggencate T, 2019-a)* | Qualitative | The intervention involves older volunteers age > 60 years teaching Dutch to expats, refugees, and immigrants in a residence for older people to ensure accessibility for older volunteers. The learning is informal, with no workbooks, and only themes and matching materials are provided. | | M: In-person I: Group-based N = 7 | The intervention provided opportunities for participants to connect with others. Participants had overall positive experiences, indicating that their social needs were met, and this contributed to their well-being. |
|  | **Self-management skills training interventions (n = 4)** | | | | | |
|  | **eHealth Self-Management System** *(Jung H, 2017 from Johnstone G, 2021)* | Quantitative (Quasi-experimental) | This intervention was looking at how eHealth self-management affects self-efficacy and self-care behaviours in older adults who live alone. As the participants did not have computer and internet at home, they spent most of their time accessing this through the community-based computer. The eHealth monitoring pilot study functioned as a 4-week class education on improving health (i.e., systolic blood pressure) and monthly telephone counselling for 24 weeks. | | M: Virtual (Computer, telephone) I: Mixed (Self-directed, one-to-one) N = 64 | There was significant improvement in social support (p=0.033) and self-efficacy (p=0.0000). |
|  | **ElderTree** *(Gustafson Sr DH, 2022)* | Quantitative (RCT) | ElderTree is a 12-month online platform designed to enhance the quality of life and provide social support for participants aged 65 and older. Rooted in self-determination theory, the interactive website addresses key components of older adults' quality of life (QOL), offering information to promote competence, connections for social support, and tools for self-management to promote autonomy. ElderTree encompasses informational, social, self-management, and motivational services to improve the overall quality of life for its users. | | M: Virtual (Computer) I: Mixed (Self-directed, one-to-one) N = 390 | No main effects were found for social support provided or received were found. |
|  | **Self-Management Well-being Intervention**  *(Kremers IP, 2006)* | Quantitative (RCT) | The intervention is based on the Self-Management of Well-being theory involved supervised by two female leaders and consisted of six meetings, each lasting 2½ hours. Single older women identified as socially isolated and/or lonely met in groups of 8–12, for six consecutive weeks. Each meeting focused on one or more of six self-management abilities. The women were taught to apply these abilities to the five basic needs (dimensions) of well-being. | | M: In-person I: Group-based N = 142 | Older adults had significant improvements in well-being, self-management ability and social loneliness at six weeks, however, the effect was not maintained after six months. There were no differences in emotional loneliness or overall loneliness between the control and intervention. |
|  | **Teaching Self-Management Skills** (*Kremers IP, 2006 from Douglas NF, 2023)* | Mixed-and/or multi-method | This 6-week intervention addressing loneliness, well-being, and self-management consisted of six sessions lasting approximately 2.5 hours each. The program aimed to teach self-management skills to lonely older women, with an average age of 62. The intervention was facilitated by two female "leaders." | | M: In-person I: Group-based N = 63 | Overall, there were no changes to loneliness and well-being. |
| **Psychological self-management interventions**  (n = 43)  *Psychological self-management goes beyond the educational and technical components (such as training, problem-solving, or behavioural approaches) of self-management education (Steed L, 2003). The interventions aim to implement cognitive or therapeutic approaches to address outcomes (Steed L, 2003). Often, this category has a motivational component that encourages people to improve their self-management capabilities (Winkley K, 2020).* | **Behavioural activation or cognitive behavioural therapy (CBT) interventions (n = 22)** | | | | | |
|  | **Behavioural Activation (BA)** *(Gilbody S, 2021 from Li M, 2023)* | Quantitative (RCT) | | An 8-session behavioural activation intervention for older adults from primary care with two or more physical conditions. | M: Virtual (Telephone) I: NR  N = 96 | Loneliness significantly decreased at 3-months. |
|  | **Behavioural Activation (BA) Interventions** *(Alaviani M, 2015; Carandang RR, 2020; Choi NG, 2020; Cohen-Mansfield J, 2018; Gilbody S, 2021; Hernández-Ascanio J, 2023; Mountain G, 2017; from Yu DS, 2023)* | Quantitative (RCT, N-RCT) | | This systematic review compared the effects of diverse non-pharmacological interventions, including behavioral activation, on loneliness in community-dwelling older adults. The behavioral activation interventions employed goal setting and counseling strategies to enhance efficacy and promote behavior changes associated with active social engagement. | M: NR I: NR  N = NR | The results of pairwise meta-analysis did not show a positive effect on loneliness at the post-test endpoint for behavioural activation (Hedges' g = −0.66; 95%CI [−1.36, 0.04]; Z = −1.86, p = 0.06; I2 : 96%, τ2 : 0.83; p < 0.01). |
|  | **Behavioural Activation in Social Isolation (BASIL)**  *(Gilbody S, 2021)* | Quantitative (RCT) | | Isolated participants aged 65 and older received virtual delivery of materials and a workbook from a trained therapist in eight sessions over 4-6 weeks, featuring activities permissible during COVID-19 social isolation measures, with each session lasting around 36 minutes. The goal of this intervention was to reduce loneliness. The program was designed as a brief telephone-delivered intervention grounded in sound psychological principles, specifically Behavioral Activation (BA). | M: Virtual (Computer, telephone) I: Mixed (Self-directed, one-to-one) N = 96 | Loneliness significantly improved. |
|  | **Behavioural Activation in Social Isolation (BASIL)**  *(Littlewood E, 2022)* | Quantitative (RCT) | | The 12-month intervention involved participants aged 65 and above in an 8-session evidence-based behavioural activation (BA) program spanning 4-6 weeks. Each session conducted remotely lasted 30 minutes (except the initial 1-hour session). Trained Behavioral Support Workers (BSWs) delivered the sessions and provided participants with a BASIL Behavioral Activation booklet. The approach paid attention to the function of behaviours, emphasizing functional equivalence to maintain social interactions. The goal was to find alternative behaviours that serve the same function as previous ones, considering physical health issues or pandemic-related limitations. | M: Virtual (Computer, telephone) I: One-to-one N = 96 | Compared to the control, loneliness non-significantly decreased in the intervention group at 12 months. |
|  | **Brief Behavioral Activation for Improving Social Connectedness** *(Pepin R, 2021)* | Qualitative | | This intervention, rooted in modified behavioural activation (BA), aimed to enhance social support, and alleviate loneliness through six weekly hour-long sessions led by a bachelor's-level interventionist. The counselling approach encompassed psychoeducation on social isolation and loneliness, behavioural activation to instigate behavioural changes, identification of personally relevant improvement areas, and activity monitoring and planning. Adapted from the Brief Behavioral Activation Treatment for Depression (BATD-R), traditionally an 8–15 session treatment, the intervention focused on reinforcing problem-solving skills and defining values to increase engagement in values-based activities. | M: Virtual (Telephone) I: One-to-one N = 3 | The preliminary research suggests that Behavioral Activation modified to address social connectedness in homebound older adults improves both social isolation and loneliness. |
|  | **Lay-Coach-Facilitated, Videocall, Short-Term Behavioral Activation Intervention**  *(Choi NG, 2020)* | Quantitative (RCT) | | Lay-Coach-Facilitated, Videocall, Short-Term Behavioral Activation (Tele-BA) intervention for homebound older adults. | M: Virtual (Computer) I: Self-directed N = 89 | Number of social interactions and satisfaction with social support significantly increased while loneliness significantly decreased after the Tele-BA (short-term behavioral activation intervention). |
|  | **Social Engage (S-ENG)** *(Van Orden KA, 2021)* | Quantitative (RCT) | | This adapted intervention aimed at individuals aged 60 and above, addressing depression-related social withdrawal. The goal was to boost social and physical engagement over a 10-week, in-home coaching program to reduce suicide risk. Participants formulated action plans, with therapists addressing identified barriers and implementing simple behavioral interventions during follow-up sessions. The intervention specifically targeted barriers like negativity bias, affect regulation, and apathy. Therapists, comprising clinical psychologists and master's level social workers, ensured fidelity throughout the program. | M: In-person I: One-to-one N = 62 | While the S-ENG intervention did not show improvements in the sense of belonging, it did lead to significant and meaningful improvements in social-emotional quality of life. |
|  | **Tele-delivered Behavioral Activation (BA)** *(Bruce ML, 2021)* | Quantitative (RCT) | | Participants aged 50 and older who were homebound and socially isolated underwent a counselling program comprising five 1-hour tele-video sessions. The goal was to address loneliness and enhance social support by engaging in healthy and social behaviours. This intervention utilized Brief Behavioral Activation (BA), a structured approach designed to boost positive behaviours aligned with personal values while reducing depressive behaviours. | M: Virtual (Computer, telephone) I: One-to-one N = 89 | Participants in the Tele-BA group, compared to those in the Tele-FV group, experienced significantly improved social interactions, satisfaction with social support, and loneliness. |
|  | **Acceptance and Commitment Therapy (ACT)** *(Zarling A, 2023)* | Quantitative (Quasi-experimental) | | This intervention targeted individuals aged 65 and older, aiming to teach skills for addressing loneliness through an 8-module, self-paced program. The modules focused on developing awareness, acceptance, self-compassion, evaluating importance, and effective action. Participants interacted with a coach during each session, completing one module per week as recommended. The intervention drew inspiration from Acceptance and Commitment Therapy (ACT), a third-generation derivative of cognitive-behavioural therapy, emphasizing acceptance, mindfulness, commitment, and behaviour change processes aligned with personal values. | M: Virtual (Not Specified) I: One-to-one N = 529 | Overall, loneliness significantly improved overtime and compared to the control. |
|  | **Cognitive Behavioural Therapy (CBT)**  *(Alaviani M, 2015 from Hickin N, 2021)* | Mixed-and/or multi-method | | The intervention consists of four sessions lasting 60 minutes each, scheduled twice per week, and is grounded in the theoretical framework of Cognitive Behavioural Theory (CBT). | M: In-person  I: Group-based  N = 150 | There was a statically significant decrease in loneliness and an increase in perceived social self-efficacy. |
|  | **Cognitive Behavioural Therapy (CBT)**  *(Choi NG, 2020 from Hickin N, 2021)* | Quantitative (RCT) | | The intervention spans five sessions, followed by a 12-week follow-up period, and is guided by the theoretical approach of Cognitive Behavioural Theory (CBT) with a focus on behavioral activation. | M: Virtual (Computer) I: Self-directed N = 89 | There was a decrease in loneliness and an increase in social interaction and social support for the intervention compared to the control. |
|  | **Cognitive Behavioural Therapy (CBT)**  *(Cohen-Mansfield J, 2018 from Hickin N, 2021)* | Quantitative (RCT) | | The intervention encompasses 10 meetings, including 7 sessions, and features a 3-month follow-up, all within the framework of Cognitive Behavioural Theory (CBT). | M: In-person  I: Mixed (Group-based, self-directed) N = 89 | There was a statically significant decrease in loneliness at the end of the intervention and at 3-months follow-up. |
|  | **Cognitive Behavioural Therapy (CBT)**  *(Jarvis MA, 2019 from Hickin N, 2021)* | Quantitative (RCT) | | The intervention spans a total of 40 sessions, conducted twice weekly over a 5-month period, with each session lasting 90 minutes, and is rooted in Cognitive Behavioural Theory (CBT). | M: Virtual (Computer) I: Mixed (Group-based, self-directed) N = 32 | There was a statically significant decrease in loneliness, and this was maintained at follow-up. |
|  | **Cognitive Behavioural Therapy (CBT)**  *(Jing L, 2018 from Hickin N, 2021)* | Quantitative (RCT) | | The intervention consists of 4 weekly phone check-ins, followed by 6 bi-monthly sessions over a 3-month period. Subsequently, there are 9 monthly sessions with follow-ups at 3, 6, and 9 months, all guided by the psychological therapy of Cognitive Behavioural Theory (CBT). | M: Virtual (Computer, telephone) I: Self-directed N = 80 | There was an improvement in loneliness for both intervention and control (intervention had higher levels). |
|  | **Cognitive Behavioural Therapy (CBT)**  *(Kremers IP, 2006 from Hickin N, 2021)* | Quantitative (RCT) | | The intervention is composed of 2.5-hour sessions conducted over 6 sessions, with a 6-month follow-up period, and is informed by the psychological theories of Cognitive Behavioural Theory (CBT), as well as self-management and goal setting. | M: In-person  I: Group-based  N = 142 | No difference in loneliness reduction compared to the control. |
|  | **Cognitive Telephone Therapy Groups**  *(Evans RL, 1986 from Cummings SM, 2004)* | Quantitative (RCT) | | Older adults living with physical disabilities participated in Cognitive Telephone Therapy Groups which met one hour a week for 8 weeks. The treatment groups focused on the development of behavioral goals, goal achievement, the use of positive reinforcement and problem-solving. | M: Virtual (Telephone) I: Group-based N = 43 | Older adults that attended telephone group therapy had significantly decreased loneliness, non-significantly higher levels of goal attainment and non-significant increased involvement in outside social activities. |
|  | **Cognitive-Behavioral Based Intervention** *(Cohen-Mansfield J, 2018 from Douglas NF, 2023)* | Quantitative (RCT) | | This intervention was conducted for 6 months over the course of 10 individual sessions with up to 10 group sessions where participants help identify barriers causing loneliness with the help of resources and counsellors. This intervention was delivered to participants aged on average 76.6 years. | M: In-person I: Mixed (Group-based, one-to-one) N = 39 | There were positive changes to loneliness. |
|  | **Group Cognitive Behavioural Therapy** *(Smith R, 2021)* | Quantitative (RCT) | | This intervention consisted of a Cognitive Behaviour Therapy (CBT) called Aging Wisely that was a 12-weekly program with 2-hour sessions with a 3-month follow-up. The program consisted of education on mental health disorders, sleep strategies, problem-solving, dealing with loss etc. The sessions were conducted with participants 60-84 years recruited from the Center for Emotional Health Clinic. This intervention underlyingly targeted loneliness. | M: In-person I: Group-based  N = 62 | There was a significant decrease in loneliness for those who completed the Cognitive Behavioural Therapy at baseline to follow-up (p=0.076). |
|  | **Integrative LISTEN Intervention** *(Theeke LA, 2016 from Hickin N, 221)* | Quantitative (RCT) | | The intervention involves five 2-hour sessions delivering educational information on aging and is guided by the psychological theory of rethinking loneliness through the integration of narrative therapy and Cognitive Behavioural Theory (CBT). The targeted population for this intervention is chronically ill older adults. | M: In-person  I: Group-based  N = 27 | There was a decrease in loneliness compared to the control. |
|  | **Lighten UP!** *(Friedman EM, 2017 from Ibrahim AF, 2022)* | Qualitative | | This intervention for those 60+ years aims to use cognitive behavioural therapy to increase well-being where each session is 90 minutes long and occurred for 8 weeks. | M: In-person I: Group-based N = 103 | Significant improvement in eudaimonic well-being, life satisfaction, and social integration after the intervention. |
|  | **Low-Intensity Cognitive Behavior Therapy**  *(Jarvis MA, 2019)* | Quantitative (RCT) | | Mhealth-Supported Intervention which targeted maladaptive cognitions in older people (age ≥60 years) experiencing loneliness in four inner-city residential care facilities. The three-month intervention using WhatsApp was implemented and included: technology acceptance, psychoeducation, and individualized positively worded messages addressing maladaptive cognitions. | M: Virtual (Computer, smartphone, tablet) I: Group-based N = 32 | Significant improvements in total loneliness, emotional loneliness, and social loneliness; the effect was maintained one month after the active intervention. Significant improvements in maladaptive social cognitions for loneliness, and emotional deprivation. |
|  | **MoodTech - Cognitive Behaviorally Informed Internet Intervention**  *(Tomasino KN, 2017)* | Quantitative (Experimental pilot study) | | MoodTech is an 8-week online intervention for depression based on CBT principles. There were 16 lessons (2/week) that included didactic content and followed 2-character storylines. Each lesson ended with directions to practice skills using program tools. Peer support features to promote social engagement and adherence via accountability. Individual coaching and group moderation provided by 2 clinical psychologists; individual coaching provided via phone calls and messages. Coaches used a dashboard to view participant activity and responses to a weekly symptom questionnaire. | M: Virtual (Computer, smartphone, tablet) I: Mixed (Group-based, one-to-one) N = 47 | Social isolation and social support non-significantly decreased among older adults in both the intervention and control group. |
|  | **Combination of different psychological interventions (n = 11)** | | | | | |
|  | **Digital Group Intervention** *(Shapira S, 2021)* | Quantitative (RCT) | | Amid the COVID-19 outbreak, a brief seven-session online intervention on Zoom equipped seniors with cognitive-behavioral and mindfulness techniques. This pilot randomized control trial assessed the impact of the intervention on enhancing coping abilities, reducing loneliness, and alleviating depressive symptoms among older adults in Israel over 3.5 weeks | M: Virtual (Computer) I: Group-based N = 86 | The intervention had a positive short-term effect on loneliness, but the effect was not maintained. Social support non-significantly improved over time. |
|  | **Holistic Therapy** *(Parlak MM, 2023)* | Quantitative (Quasi-experimental) | | Participants 60-90 years with mild to moderate Alzheimer's disease were part of a therapy intervention for 6 weeks every Thursday. Participant's received reminiscence therapy which was once per week for an hour and was based on a topic from a past experience (i.e. marriage) and the discussion lasted 25-35 minutes. Next, music therapy was administered for 5-10 minutes where music was chosen from past experiences. Participants were asked to write songs they liked. Lastly, reality orientation therapy was administered where participants were asked about personal information from their life (i.e. name, day of the week, occupation, city of birth etc.). This lasted for 30-35 minutes. | M: In-person I: Group-based  N = 20 | Overall, from pre-test to post-test there was a significant decrease in social participation (p=0.027), no significant change in social isolation (p=0.285), and a significant increase in quality of life for participants (p=0.039). |
|  | **Mindfulness Intervention** *(Creswell JD, 2012 from Hickin N, 2021)* | Quantitative (RCT) | | The intervention comprises 8 weekly sessions lasting 120 minutes each, supplemented by a day-long retreat and 56 individual practice sessions lasting 30 minutes each, all guided by the psychological theory of mindfulness. | M: In-person  I: Mixed (Group-based, self-directed) N = 40 | There was a statically significant decrease in loneliness. |
|  | **Mindfulness, Reflections, and Psychology Concepts** *(Hudson J, 2023)* | Qualitative | | This intervention was for participants 73 years on average and was based on an 8-module set-up. Each module had a lesson, quiz, and activity to apply the learned content. At the end of each module, participants were asked to initiate contact with a coach to discuss goals. The session modules included various topics on cognitive-behaviour, acceptance, and mindfulness such as: values, coping, thoughts, relationships skills etc. After the sessions, participants were locked out of the program to encourage contemplation on goals and learned material. | M: Virtual (Not Specified) I: One-to-one N = 11 | Participants gained new social skill, improved relationships, and increased confidence to initiate and maintain social contact. |
|  | **Psychological Interventions** *(Keisari, 2022; Shapira, 2021; Sayied, 2015; Borji, 2020; Ojha, 2016; Pandya, 2021 from Yu DS, 2023)* | Quantitative (RCT, N-RCT) | | This systematic review compared the effects of various non-pharmacological interventions, including psychological interventions, on loneliness in community-dwelling older adults. The psychological interventions were characterized by promoting positive cognitive beliefs about self, others, and interpersonal contexts, managing risk factors, and enhancing emotional well-being. | M: NR I: NR  N = NR | Psychological interventions showed large effects on loneliness at the first post-test endpoint (n = 6, Hedges' g = −2.33; 95%CI [−4.40, −0.25]; Z = −2.20, p = 0.003; I2: 98%, τ2 : 6.61; p < 0.01). |
|  | **Psychological Therapies** *(Choi NG, Marti CN, 2020; Choi NG, Pepin R, 2020; Gilbody S, 2021 from Chau CMS, 2023)* | Quantitative (RCT) | | This review includes interventions based on psychological therapies such as cognitive behavioural therapy (CBT). | M: Virtual (Computer, telephone) I: NR  N = NR | Social engagement (2 studies, 366 participants) significantly improved, whereas there was no significant change in loneliness (2 studies, 179 participants). |
|  | **Remotely Delivered Technology** *(Conroy KM, 2020 from DesChâtelets JR, 2023)* | Qualitative | | Community-dwelling adults were delivered an intervention that focuses on digital health that could track and monitor mental health. | M: NR  I: NR  N = NR | The intervention may address loneliness among elderly populations and aid in the maintenance of social connections. |
|  | **Resilience- and Wisdom-Focused Intervention** *(Jeste DV, 2022)* | Quantitative (Quasi-experimental) | | A 6-week virtual individual-level intervention for community-dwelling older adults (≥ 65 years) aimed to reduce perceived stress and loneliness through cognitive, affective, and behavioural components. The intervention was conducted remotely once a week and addressed cognitive aspects through education on empathy, compassion, self-compassion, and attitudes toward aging, including Cognitive Behavioral Therapy (CBT)-informed thought-challenging skills. Affective components included role plays, mindfulness, meditations, and self-compassion exercises. Behavioural aspects involved savouring and gratitude practices, daily gratitude diaries, social skills training, engagement in value-based activities, exercises to enhance self-esteem and self-efficacy, and daily home-based practice. Interactive sessions incorporated deep breathing exercises, and therapists assisted participants in identifying and encouraging value-driven activities to achieve short-term well-being goals. | M: Virtual (Computer) I: One-to-one N = 20 | While the sample was too small for demonstrating efficacy, there was a reduction (small-to-medium effect size) in perceived loneliness and an increase in resilience. |
|  | **Resilience- and Wisdom-Focused Intervention** *(Jeste DV, 2023 from Li M, 2023)* | Quantitative (Cross-sectional) | | Community-dwelling older adults participated in an intervention centered around resilience and wisdom. The program encompassed cognitive training, mindfulness, meditation, social skills, self-esteem, and self-efficacy components. | M: Virtual (Not Specified) I: NR  N = 20 | There was a small to medium effect size decrease in loneliness and the effect was sustained for the follow-up period. |
|  | **Teaching and Practicing Cognitive Behavioural and Mindfulness Skills** *(Shapira S, 2021 from Li M, 2023)* | Quantitative (RCT) | | Community-dwelling adults engaged in a comprehensive intervention program, conducted twice a week over 7 weeks. Each session, lasting 60-90 minutes, centred around cognitive-behavioural therapy principles, incorporating elements of mindfulness, meditation, and coping skills. The intervention utilized an online course format, fostering group discussions as a key component. | M: Virtual (Not Specified) I: Group-based  N = 82 | There was a decrease in loneliness from T0 to T1; however, this was not sustained for T2 (p>0.05). |
|  | **Therapy-Focused Interventions** *(Creswell JD, 2012; Drentea P, 2006; Liu SJ, 2007 from Paquet C, 2023)* | Mixed-and/or multi-method | | This intervention type included reminiscence therapy and mindfulness to reduce stress. | M: NR  I: NR  N = NR | Overall, the interventions were effective in decreasing loneliness. However, there was a risk of bias, small sample sizes, and poor quality, limiting confident conclusions. |
|  | **Psychosocial interventions (n = 5)** | | | | | |
|  | **Empowering Participants to Achieve Goals** *(Routasalo PE, 2009 from Douglas NF, 2023)* | Quantitative (RCT) | | This intervention was based on a psychosocial intervention to help lonely adults 75-92 years feel empowered over their lives and themselves which occurred over the course of 3 months in 12 weekly sessions to decrease loneliness and help with well-being. | M: In-person I: Group-based N = 117 | Overall, there were no positive changes to feelings of loneliness and social networks; however, there was a positive change to making new friends. |
|  | **Mood Lifters for Seniors Program** *(Roberts JS, 2022)* | Quantitative (Quasi-experimental) | | The Mood Lifters program, led by trained layperson graduates, engages older adults (65 and older) in 15 weekly, 1-hour meetings. These sessions focus on diverse biopsychosocial topics impacting mood and quality of life. Participants earn points for practicing learned behaviors between sessions, aiming for a self-determined behavioral goal. | M: Virtual (Computer) I: Group-based N = 24 | No significant changes were reported in participants’ loneliness, or resilience |
|  | **Psychosocial Intervention Programme Using Volunteers** *(Lorente-Martínez R, 2022)* | Mixed-and/or multi-method | | A 25-hour psychosocial support intervention, called the Acompaña-Té program, was delivered by university volunteers to prevent loneliness, and enhance self-efficacy and social participation in older women (aged ≥ 65) living alone in Spain. The program focused on three components: conversation, attribution retraining (sessions 2 to 4), and behavioral activation (sessions 4 to 9). University volunteers, mainly undergraduate students aged 18 to 30 from various disciplines, administered the intervention. They were selected through a motivational telephone interview, declaring no criminal record or health conditions hindering their participation. Most volunteers (66%) had no previous volunteering experience. | M: In-person I: One-to-one N = 48 | No subjective social participation changes were identified with quantitative data, although positive qualitative changes were reported. Likewise, no quantitative changes in loneliness were observed, but participants reported they felt less lonely in the qualitative findings. |
|  | **Telephone Crisis Program**  *(Morrow-Howell N, 1998 from Cohen-Mansfield J, 2015)* | Quantitative (Quasi-experimental) | | One-to-one intervention for older adult participants (age ≥55 years) with a psychosocial element involving a telephone crisis program that included supportive therapy on communication skills. | M: Virtual (Telephone) I: One-to-one N = 61 | The amount of social contact and interpersonal contact significantly increased compared to waitlisted controls. Loneliness was unchanged. No significant changes in the amount of telephone contact, or satisfaction with socialization. |
|  | **The Happiness Route** *(Weiss LA, 2020 from Douglas NF, 2023)* | Quantitative (RCT) | | This intervention teaches older adults with chronic illness 59 years old (on average) positive psychological treatments (based on the self-determination theory) with a counsellor's help over the course of 3 months, for 1.5-hour visits throughout 2-6 visits to reduce loneliness and increase social participation. | M: In-person I: One-to-one N = 58 | There was no change to loneliness or social participation. |
|  | **Reminiscence therapy (n = 5)** | | | | | |
|  | **Group Reminiscence Therapy Based on Chinese Traditional Festival Activities (CTFA-GRT)** *(Li S, 2022)* | Quantitative (RCT) | | Older adult participants 60+ who were living alone participated in one, 4-hour session which was based on improving loneliness for Chinese participants living in a rural China. The various topics were chosen based on the month's certain celebrations such as lantern festival, international women's day etc. | M: In-person I: Group-based  N = 64 | Loneliness significantly improved. |
|  | **Memory Matters App** *(Yu F, 2019 from Heins P, 2021)* | Quantitative (RCT) | | The intervention examined was a mobile reminiscing therapy app, Memory Matters, that offered one-on-one 30 min sessions with an interventionist (2×/week) for 6 weeks followed by independent use for 6 weeks. | M: In-person I: Mixed (Group-based, self-directed) N = 80 | At six weeks, there was significantly higher social interaction in the individual Memory Matters app user group; However, this was not maintained for 12 weeks. |
|  | **Reminiscence Therapy (RT)** *(Diwan S, 2023)* | Mixed-and/or multi-method | | The intervention involved 10-12 weekly visits to Hispanic and Vietnamese participants, averaging 75 years, using a culturally adapted Reminiscence Therapy (RT). The adaptation included family involvement, community events showcasing participants' life stories, and ethnically matched bilingual community workers. Core modifications incorporated family participation, community event involvement, and case management for additional referrals. | M: In-person I: Mixed (Group-based, one-to-one) N = 190 | Participants’ loneliness, and life satisfaction significantly improved. The qualitative data suggest that the storytelling intervention led to improvements in family relationships. |
|  | **Reminiscence Therapy in Combination with Physical Exercise** *(Ren Y, 2021)* | Quantitative (Quasi-experimental) | | The intervention group included reminiscence therapy in combination with physical exercise (Taijiquan) for 8 once a week, 50–60-minute sessions. The control group listened to 4 routine health lectures, for which the experimental group also listed to. The themes of weekly group activities were set according to the feedback from group counseling. | M: In-person I: Group-based N = 130 | Loneliness and resilience significantly improved over time (p < 0.05) in the intervention group. |
|  | **Reminiscence Through Pictures** *(Coll-Planas L, 2017 from Ibrahim AF, 2022)* | Quantitative (Quasi-experimental) | | This intervention was based on creating social support and participation for those 60+ years. Each group was led by a nurse or social worker where they started with reminiscence through personal items. This occurred for 15 weeks and was held in 1.5-hour sessions. | M: In-person I: Group-based N = 38 | There was a significant decrease in loneliness and significant increase in social participation after a 2-year evaluation. |
| **Social prescribing or asset-based interventions**  (n = 8)  *World Health Organization (WHO) [*[*2*](https://bmcpublichealth.biomedcentral.com/articles/10.1186/s12889-024-17736-2#ref-CR2)*] has defined social prescribing as “a means of connecting patients to a range of non-medical services in the community to improve their health and well-being.” Asset-based interventions include components that encourage participants to utilize community resources to improve their self-management (Cassetti et al., 2019).* | **Connecting Points – Connecting People Project**  *(Bartlett H, 2013)* | Quantitative (Quasi-experimental) | Hervey Bay City Council coordinated the Connecting Points – Connecting People project. Activities included community forums, better integration of services for older people, establishing a shop front contact point, developing an action plan and resource kit, and implementing a buddy system (to connect a volunteer with a socially isolated older person to help build confidence and encourage engagement in social activities). The support provided encouragement of self-reliance and independence. | | M: In-person I: One-to-one N = 15 | The Connecting Points program did not improve social support or loneliness in older adults. |
|  | **Culturally Appropriate Volunteer Services (CAVS)** (Bartlett H, 2013) | Quantitative (Quasi-experimental) | This project aimed to develop a culturally appropriate model of volunteer service delivery for seniors that incorporated a focus on social isolation. It included a resource worker to assist agencies recruit and train volunteers and share information and resources. The project also involved delivering social and leisure activities and library services for older migrants through two ethnic community organisations, the OzPol Seniors’ Day Centre and the Cathay Community Association Incorporated. | | M: In-person I: One-to-one N = 13 | CAVS produced a significant positive difference in loneliness and social support for older adult volunteers. |
|  | **Health and social care interventions** *(Findlay RA; 2003, Gardiner C, 2018; Bickerdike L, 2017 & Poscia A, 2018 from Freedman A, 2020)* | Quantitative (RCT) | Health and social care interventions to support the identification and referral of at-risk individuals to outreach and geriatric rehabilitation. | | M: NR I: NR  N = NR | Health and social care interventions can best address social isolation and loneliness if they involve trained individuals or health care professionals. |
|  | **Link Worker Social Prescribing Programme**  *(Moffatt S, 2017)* | Qualitative | Personalised support to assist participants in identifying meaningful health and wellness goals, while providing ongoing support to achieve agreed objectives and linkage with appropriate community services. Link workers and participants could meet in-person face to face, or via telephone, email and/or text message. | | M: Mixed (In-person and virtual (computer, smartphone, tablet)) I: One-to-one N = 30 | The program increased feelings of control and self-confidence and reduced social isolation. |
|  | **Program to Encourage Active, Rewarding Lives (PEARLS)** *(Steinman L, 2021)* | Quantitative (Quasi-experimental) | PEARLS (Program to Encourage Active, Rewarding Lives) offers targeted support to underserved older adults (aged 50 or older) dealing with depression. In collaboration with community-academic partnerships, PEARLS trains front-line providers to engage homebound individuals who have limited access to traditional clinical care. Over eight 1-hour home visits spanning 4 to 6 months, PEARLS providers (referred to as "coaches" or "counselors") employ Problem Solving Treatment (PST) to help participants develop problem-solving skills, gain a sense of control over life's challenges, and plan meaningful activities through Behavioral Activation. Additionally, providers offer psychoeducation and facilitate linkages to social and health services when needed. Clinical supervision is provided for those with complex health needs. | | M: In-person I: One-to-one N = 320 | At 6-months, social interactions, satisfaction with social support, perceived isolation and loneliness significantly improved. |
|  | **Reconnections Program** *(McDaid D, 2023)* | Qualitative | Individuals aged 50 and above, identified as lonely and not in full-time employment, were enrolled in the Reconnections program in Worcestershire, England. This initiative aimed to alleviate loneliness through personalized support and community response service. Over 6-9 months, participants worked with local voluntary and community sector partners to develop individualized plans. The program focused on understanding their strengths and needs, rebuilding confidence, and facilitating connections with people, places, or activities in their communities. Volunteers from local partners played a crucial role in linking participants with various activities, such as coffee mornings, lunch clubs, and arts groups. | | M: In-person I: Mixed (Group-based, one-to-one) N = 41 | Most participants established some meaningful social connections and helped people whose confidence had been knocked back by significant life events. |
|  | **Social Prescribing**  *(Kim JE, 2021 from Li M, 2023)* | Quantitative (Cross-sectional) | Rural residents participated in a social prescribing activity that encompassed (music, storytelling, gardening, and self-help groups). This was conducted for 10 weeks, once per week. | | M: In-person  I: NR  N = 10 | There was a decrease in loneliness (p<0.1) and an increase in social participation after the intervention (p<0.05). |
|  | **Social Prescribing**  *(Kim JE, 2021)* | Quantitative (Quasi-experimental) | This intervention used a social prescribing strategy and was for participants 65 and older years where the intervention encompassed music storytelling (once per week for 90 minutes), self-help (COVID-19 prevention, song writing, healthy fruit choices) (once per week for 1 hour), gardening (education on gardening, monitoring, harvesting) (once per week for 30 minutes), and COVID-19 prevention (voluntary (three times per week), COVID-19 call (4 times per week), education on nutrition (once per week)). The aim was to decrease loneliness in elderly Korean folk living in a rural area. | | M: Mixed (In-person and virtual (telephone) I: Group-based  N = 10 | Overall, results show that loneliness was decreased (p<0.01) and social participation increased (p<0.05) after the intervention. |

*****M=mode of delivery; I = level of interaction with interventions; N=number of participants; NR = Not reported.

**Table 1c:** Study and intervention characteristics, including a summary of study results and outcomes for *Social behavioural activity-related interventions* (n = 140)

| **Intervention Type** (number of studies)  *Definition* | **Intervention name**  *(Author, year)* | **Study design** | **Intervention and population description** | **Intervention details** | **Summary of study results and outcomes** |
| --- | --- | --- | --- | --- | --- |
| **Arts-based interventions**  (n = 27)  *Arts-based interventions deliver activities that facilitate a creative experience for participants (Carswell et al., 2019) in any arts-based discipline, such as performing arts, community and cultural festivals, visual arts, design and craft, literature, fairs and events, and online digital/electronic arts (Davie et al., 2012). It can involve both direct engagement (e.g., painting a picture or playing a musical instrument) or receptive engagement (e.g., attending a concert or museum).* | **Direct engagement arts-based interventions (n = 21)** | | | | |
|  | **Acting and Improvisation Course**  *(Sutherland L, 2023)* | Mixed-and/or multi-method | Conducted a 12-week acting and improvisation intervention for participants aged 52-71 from a senior center, led by trained theatre professionals. Aimed at fostering group bonding and social connections among older adults in low-income housing in urban Detroit. The course, administered three times between 2017 and 2018, culminated in a public performance, with a master’s-trained teaching artist facilitating and researchers documenting each session. | M: In-person I: Group-based  N = 14 | Our quantitative results indicated no significant change in social isolation, community belonging, and social exclusion. |
|  | **Activity-Based Musical Engagement Using iPads** *(Engelbrecht R, 2015 from Heins P, 2021)* | Mixed-and/or multi-method | The intervention provided 1-hour sessions of activity-based musical engagement with iPads in groups over 5 weeks. | M: NR I: NR  N = 6 | Quantitative: No significant differences in social isolation between the iPad and the traditional music instrument group and within the groups (pre- vs. post-test).  Qualitative: Reported benefits for both groups included enhanced development of social cohesion. |
|  | **Arts-Based Intervention** *(Watson B, 2023)* | Quantitative (RCT) | Seniors (aged 65 and older) were recruited into an arts-based intervention. Weekly 2-hour sessions were held over a 16-month period and consisted of drawing, painting, collage, clay work, performance, sculpting, and mixed media. The impact on loneliness and well-being were assessed, along with social quality, activity, and functioning. | M: In-person I: Group-based N = 252 | When controlling for various factors, loneliness and social quality significantly improved over time. However, social activity, social functioning, and general health did not significantly change. |
|  | **Bibliotherapy and Therapeutic Creative Writing Group** *(Malyn BO, 2020 from Noone C, 2022)* | Qualitative | The intervention included community-based bibliotherapy and therapeutic creative writing groups, consisting of three reading and writing for well-being groups. | M: In-person I: Group-based N = 12 | The intervention enhanced social connections and relationships with self and others. |
|  | **Chorale Singing Program** *(Cohen GD, 2007 from Ibrahim AF, 2022)* | Qualitative | A 2-year chorale singing program for individuals aged 65 and above, featuring weekly rehearsals led by professionals, totalling 30 weeks, and culminating in 10 public concerts. The aim was to promote social engagement, with a focus on measuring the impact on physical health, mental health, and social activities. | M: In-person I: Group-based N = 128 | Significant improvement in overall health and social activity levels after the intervention. No significant difference between intervention and control groups for loneliness. The intervention group sustained social activity levels, while the comparison group declined after two years. |
|  | **Community Dance Program (CDP)** *(Wu VX, 2023)* | Qualitative | Older adults, aged 62-83, participated in an 8-week Community Dance Program (CDP) led by undergraduate students from the Dance Synergy program. The CDP, aiming to enhance psychosocial well-being through dance, included exercises, choreography, and intergenerational interaction. | M: Mixed (In-person and virtual (computer)) I: Group-based N = 20 | Group interactions in dance sessions facilitated networking among older adults and student instructors, forming a social support system and reducing the risk of social isolation. Verbal and nonverbal exchanges during the activity fostered meaningful relationships, allowing the cultivation of intergenerational bonds alongside dance skill development. |
|  | **Community of Voices (COV)** *(Johnson JK, 2020 from Ibrahim AF, 2022)* | Quantitative (RCT) | This intervention took place for 12 months and was based on a community choir program that was delivered at senior centers. This was for those 60+ years and the aim was to reduce loneliness. | M: In-person I: Group-based N = 390 | There was a significant decrease in loneliness for those who participated in the choir. |
|  | **Community of Voices (COV)** *(Salazar M, 2020)* | Qualitative | The COV intervention involved weekly 90-minute meetings held over 44 sessions throughout a year for older adults, aiming to foster social connections and engagement. Delivered by professionals, it included activities like learning songs, breathing exercises, and group discussions | M: In-person I: Group-based  N = 31 | Group singing enhanced the overall sense of well-being. Additional benefits included improved self-confidence, heightened social connectedness, increased social support, and reduced loneliness. |
|  | **Dance Interventions** *(Shanahan J, 2016; Skingley A, 2016; Brustio PR, 2018; Pacheco E, 2016; Gouvêa JA, 2017; Douka S, 2019; Clifford AM, 2019;  Westheimer O, 2015; McNeely ME, 2015; Shanahan J, 2017; Merom D, 2016; O’Toole L, 2015; Marquez DX, 2015; Merom D, 2016 from McQuade L, 2023)* | Mixed-and/or multi-method | This mixed-method systematic review aimed to develop a better understanding of the evidence on the impact of arts and creativity, including dance, on older people’s (aged ≥ 50) psychological health and well-being. | M: Mixed (In-person and virtual (video)) I: Mixed (Group-based, one-to-one) N = NR | For dance interventions, three of four studies found significant improvements in activities in daily living over time and compared with a control group. Similarly, five of nine dance interventions significantly improved quality of life. Two studies assessed social engagement, finding mixed significance after dance interventions. |
|  | **Digital Storytelling (DST)** *(Freeman S, 2020; Hausknecht S, 2019; Brandão, L, 2021;  Hausknecht S, 2017; Stenhouse R, 2013; McGovern J, 2019; Loe M, 2013; Ward A, 2020; Bentley F, 2011; Karlsson E, 2014; Schoales C, 2020; Sljivic H, 2022; Sweeney L, 2021 from Chang H, 2023)* | Mixed-and/or multi-method | Digital storytelling (DST) is a way for people to tell their stories through digital media, such as videos, music, photos, etc. DST can include fusing pictures and audio and including narration to produce a film of one's lived experiences. It can also be a way of self-expression for older adults. The older adults included in this study were 50-99 years old. | M: NR  I: NR  N = NR | Digital storytelling interventions helped older adults feel more connected with others. Students and spouses who accompanied older adults in these tasks and the older adults themselves reported a greater sense of belonging and strengthened relationships after performing the tasks related to digital storytelling. |
|  | **Improvisation Comedy: Humor Doesn't Retire (HDR) Program**  *(Morse LA, 2018)* | Qualitative | The Humor Doesn't Retire program at The Second City in Chicago was specifically designed for adults aged ≥ 55 years who wanted to learn humour improvisation. There were six different levels available, ranging from beginner classes for those who have no experience to advanced ensembles that culminate in a final performance. | M: In-person I: Group-based N = 10 | The experience provided participants with a sense of community and developed behaviours that promoted social interaction and better problem-solving abilities. |
|  | **Music and Singing Interventions** *(Fu MC, 2018; Seinfeld S, 2013; Yap AF, 2017;  Johnson JK, 2020; Davidson JW, 2014; Hallam S, 2014; Hallam S, 2016; Johnson JK, 2013 from McQuade L, 2023)* | Mixed-and/or multi-method | This mixed-method systematic review aimed to develop a better understanding of the evidence on the impact of arts and creativity, including music and singing, on older people’s (aged ≥ 50) psychological health and well-being. | M: In-person I: Group-based N = NR | For music and singing interventions, four found significant improvements in measures of subjective well-being. For quality of life, six of eight studies found no significant changes. Loneliness improved significantly in one of two music and singing interventions. Social network scores did not improve in one study. Perceptions from 15 studies suggested singing and music interventions enhanced well-being, and sense of belonging. Fourteen studies also found perceptions of increased social networks and connections. |
|  | **Playful Living Program**  *(Brandão L, 2022)* | Mixed-and/or multi-method | 3-month online intergenerational intervention in southern Brazil targeting vulnerable older adults (60 and above), especially those with aphasia and dementia, amid the COVID-19 pandemic. Conducted weekly for 1 hour, the program, adapted to the pandemic, involved clowning, storytelling, dancing, and cooking led by undergraduate students to enhance well-being and social connections. | M: Virtual (Computer) I: Mixed (Group-based, one-to-one) N = 34 | Three-month evaluative sessions revealed participants felt a sense of belonging, with positive affirmations about social connection being the most common responses across all groups. |
|  | **Professionally Conducted Chorale**  *(Cohen GD, 2006 from Cohen-Mansfield J, 2015)* | Quantitative (N-RCT) | Adults 65 and older participating in a professionally conducted chorale, including several public performances. | M: In-person I: Group-based N = 166 | At 1-year follow-up, both groups showed a slight decrease in loneliness for the intervention group, but this was insignificant. |
|  | **Robot To Socialize** *(Fields N, 2019 from Rivera-Torres S, 2021 )* | Quantitative (Pre-post) | This intervention was based on having a robot to increase social participation in a participatory art intervention of reciting Shakespeare sonnets for those 65+ years. | M: In-person I: NR  N = 15 | There were reports of significantly improved loneliness. |
|  | **Singing in Chorale** *(Cohen GD, 2006 from Tricco AC, 2022)* | Quantitative (N-RCT) | This intervention was based on participating in a professional singing chorale where participants, who were 79 years old on average, were a part of rehearsals for 30 weeks and public performances. The control group conducted their usual activities. | M: In-person I: Group-based N = 166 | There was a decrease in loneliness after 12 months however this was not statistically significant (p=0.08). |
|  | **Singing Sessions** *(Teater B, 2014 from Ibrahim AF, 2022)* | Mixed-and/or multi-method | A 3-month community arts intervention with weekly 1-hour singing sessions led by paid instructors using the 'Golden Oldies' songbook, aiming to reduce loneliness and enhance participants' health, self-development, and social connectedness. | M: In-person I: Group-based N = 120 | Qualitative analysis found the program contributed to participants' social connectedness, reduced social isolation, and increased social contact. Self-rated overall health significantly increased after the program. |
|  | **The CALL-ME Community Arts Project**  *(Murray M, 2010)* | Qualitative | This intervention aims to promote social interaction among older people in disadvantaged neighborhoods. It has four sub-projects investigating different forms of social intervention in four different neighborhoods allocated by city officials based on social indicators. Its sub-project explores how community arts activities can connect with the material and psychosocial worlds of older residents and provide opportunities for transformation. Participants had the opportunity to present their artistic representations and experiences through an exhibition to other local residents. | M: In-person I: Group-based N = 11 | The intervention increased opportunities for social interaction and forming new friendships, which in turn caused feelings of challenging the negative outsider social representation of their community. |
|  | **The Community of Voices (COV) Choir Program**  *(Johnson JK, 2020)* | Quantitative (RCT) | Each choir session included activities targeting cognitive, physical, and psychosocial engagement by which a choir could promote health and well-being of older adults. The choirs were led by professional choir directors and accompanists from local communities. Choir directors identified music repertoire that could be culturally tailored for each site and for a wide range of singing abilities and experiences. The 90-min choir sessions took place at the senior centers, and each choir met weekly for 44 weeks, including 3‚ informal public performances | M: In-person I: Group-based N = 390 | The choir intervention significantly decreased loneliness and significantly improved subjective feelings of loneliness (also understood as reducing apathy). |
|  | **The Golden Oldies Community-engaged arts (CEA) singing session program**  *(Teater B, 2014 from Poscia A, 2018)* | Mixed-and/or multi-method | The intervention provides an environment and resources for older adults to get together and sing songs for 1 hour per week. | M: In-person I: Group-based N = 120 | Quantitative: Participants identified participation as contributing to their sense of community. Qualitative: The intervention led to expanded community connections, increased amount of social contact, and decreased social isolation. |
|  | **Traditional Music Engagement** *(Engelbrecht R, 2015 from Heins P, 2021)* | Mixed-and/or multi-method | The intervention provided 1-hour sessions of activity-based musical engagement in groups over 5 weeks using traditional music instruments. | M: NR I: NR  N = 6 | Quantitative: No significant differences in social isolation between the iPad and the traditional music instrument group and within the groups (pre- vs. post-test). Qualitative: Reported benefits for both groups included enhanced development of social cohesion. |
|  | **Receptive engagement arts-based interventions (n = 4)** | | | | |
|  | **Montreal Museum of Fine Arts (MMFA)** *(Beauchet O, 2022)* | Quantitative (RCT) | Montreal residents aged 65 and over participated in virtual tours through the MMFA (Montreal Museum of Fine Arts), where 6-8 people met for 45 minutes and were in groups with a trained guide meeting once a week for 3 months after there was a 15-minute period for socializing and discussion. | M: Virtual (Computer) I: Group-based N = 106 | Social isolation, well-being, quality of life and physical frailty significantly improved in the intervention group. |
|  | **The Bealtaine Art Festival Program**  *(O’Shea E, 2012)* | Qualitative | The festival promotes involvement of older people in arts through a month-long celebration, offering various events in music, theatre, literature, dance, film, and more, encouraging creativity and participation at all levels. The festival emphasizes enjoyment and participation at all levels, with the goal of celebrating creativity in older age and encouraging continued and future participation. | M: In-person I: Group-based N = 253 | Considerable gains in social cohesion and participant connectivity with others, as well as positive impacts on quality of life, reducing loneliness, and increased social networking, were reported. |
|  | **Visual Art Discussions**  *(Wikström BM, 2002 from Cohen-Mansfield J, 2015)* | Quantitative (RCT) | Older women aged 70-97 living in a block of specially designed older persons’ flats in Sweden met as a group and had facilitated discussion about well-known artists’ visual works of art. | M: In-person I: Group-based N = 40 | There were significant reductions in loneliness after the visual art discussions. |
|  | **Visual Arts Discussion** *(Wikström BM, 2002 from Douglas NF, 2023)* | Quantitative (Controlled trial) | This intervention was to help with social interaction and was based on having discussion on visual arts for 4 months with one weekly session 1 hour long for adults living in housing aged 70-79 years. | M: In-person I: One-to-one N = 20 | Overall, there was a significant positive change in social interactions. |
|  | **Combination of direct and receptive engagement arts-based interventions (n = 2)** | | | | |
|  | **Museum Social Prescribing Program**  *(Todd C, 2017)* | Qualitative | Involved museums who offered varied activities and sessions, with all including information sharing components led by staff followed by a range of activities such as object handling, participatory arts, crafting, and music making. The activities were not uniform but were tailored to each museum's unique offerings. | M: In-person I: Group-based N = 20 | Participants experienced more novel and intense social experiences, built relationships, and made meaningful connections. They reported feeling less lonely and the program provided an opportunity to connect with others. |
|  | **Music Engagement Program for Men**  *(Lindblad K, 2020)* | Qualitative | Comprised of collecting and listening to records, alone or with others, attending festivals and concerts, listening to the radio, dancing, singing, participating in a choir or amateur orchestras, and playing instruments. | M: In-person I: Mixed (Group-based, self-directed) N = 15 | Engagement with music was more than an activity for participants; it filled their deep social needs. Men in the study described developing and maintaining friendships as a factor related to their engagement with music. |
| **Leisure activities interventions**  (**n=48)**  *Leisure activities interventions facilitate an individual's participation in non-obligatory activities and recreational activities (excluding work, self-care, or sleep), such as gardening, cooking, or playing sports (Li J, 2021). The non-obligatory nature of the activities refers to an individual having the choice, desire, and preference to engage in the activity (i.e., it is not out of need).* | **Group-based leisure activity interventions (n = 19)** | | | | |
|  | **Come Eat Together (CET) Program**  *(Wildman JM, 2019)* | Qualitative | The CET project's main aim is to promote social interaction among older adult participants by bringing them together around food‐related activities. | M: In-person I: Group-based N = 8 | 97% of participants agreed that they had met new people and friends, and all reported wider social networks. |
|  | **Community Connection Program** *(Siette J, 2021)* | Mixed-and/or multi-method | Enrich Living Services' Community Connections program is a 6-month excursion-based initiative for older adults (aged 65+) in Western Australia. The program, designed to enhance participants' quality of life, involves a variety of organized group outings, categorized into highly adventurous experiences and regular social engagements. Activities from supported ice skating to river cruises occur in public and community settings. | M: In-person I: Group-based N = 56 | Our study presents evidence that excursion-based group community care programs can positively improve older adults’ quality of life. Our findings indicate that participation in this program was associated with increased socialisation and maintaining social connections. |
|  | **Connect 60+ Wellness Program**  *(Weselman T, 2022)* | Qualitative | The Connect 60+ program, targeting individuals aged 60 and above, ran for nine weeks, focusing on wellness and healthy aging. Sessions, occurring once a week for 3 hours, were offered both online and in person at a community hub. The program utilized a community-based participatory research approach, incorporating local member preferences. The sessions included 60 minutes of strength and balance exercises, following guidelines for improving health among older individuals. Online participants joined live-streamed classes facilitated by the primary researcher, engaging in breakout rooms for discussions and participating in various wellness activities such as ballroom dancing, chair yoga, or storytelling. Outdoor activities like pole walking and nature walks were inclusive for all participants. | M: Mixed (In-person and virtual (computer)) I: Group-based N = 13 | The study found that the intervention facilitated sustained change in their motivation to make social connections and even seek out new activities, which helped them develop the confidence to build relationships outside the program. |
|  | **Connection Through Calls: Seniors' Centre Without Walls (SCWW)** *(Roland H, 2021)* | Quantitative (Cross-sectional) | This intervention is intended for those 55+ years and are community-dwelling. The goal is to provide social connections and build inclusivity. This intervention is based on conference calls in small groups (7-10 people), where the participant can choose to join a program based on their interests (recreation, learning, health/wellness) which was led by a facilitator. The calls were about 2 hours a day a various time in the week. | M: Virtual (Telephone) I: Group-based N = 160 | Overall, there was an improvement in loneliness from baseline to follow-up (p<0.05). |
|  | **Engage with Age Program** *(Montoro-Rodriguez J, 2022)* | Quantitative (Pre-post) | The Engage with Age (EWA) activities program was designed to improve the health, quality of life and support residents of senior residents in low-income affordable housing to age well in place. The range of activities targeted physical, cognitive, socio-emotional health, and well-being needs. Activities include informational lectures, community social activities, exercising, or health education. Standard support included support from service coordinators who provide residents with information and acted as liaisons between community agencies, service providers, and residents via occasional transportation to off-site services, and coordination of services. | M: In-person I: Group-based N = 86 | Loneliness and social isolation significantly improved over time (as measured by the repeated measures). |
|  | **Extra Time Hub (ETH)** *(Jackman PC, 2023)* | Qualitative | The 'Extra Time Hub' (ETH) is a social group for individuals aged 55 and above, organized by a Midlands football club since 2019. It targets retirees or those approaching retirement, promoting socialization and physical activity through weekly gatherings. Participants are recruited via club communication, local media, and health services. Wednesday morning sessions include activities like quizzes, bingo, bowling, and table tennis, costing £3 and providing socialization opportunities with light refreshments. Additional activities, both free and fee-based, are held throughout the week at the club's stadium and community venues. During the COVID-19 pandemic, online Zoom calls were introduced for continued engagement. | M: Mixed (In-person and virtual (computer)) I: Group-based N = 10 | Participants perceived improvements in social connectedness, social isolation, and loneliness. Participants felt ETH enabled opportunities to meet people, engage in conversation, build new friendship networks and boost their confidence in social situations. |
|  | **Healthy Aging Web-Based Activity Program** *(Cohen-Mansfield J, 2021)* | Mixed-and/or multi-method | Healthy Aging, a for-profit organization offering rehabilitation services to older individuals, transitioned to providing online activities during the COVID-19 pandemic. These activities, including exercise, mindfulness, tai-chi, and lectures, were initially offered free of charge and later for a small subscription fee. Held on Zoom, these activities occurred five days per week, featuring three 30-minute sessions daily between 10 AM and 11:30 AM. The first activity focused on seated exercises, followed by varied sessions such as mindfulness, musical tai-chi, self-help, and lectures covering world travel, history, health, and mental health. Informal chat sessions began at 9:30 AM, and participants were encouraged to discuss during lectures and mindfulness activities. | M: Virtual (Computer) I: Group-based N = 105 | The results suggested insufficient provision of social contact and preventing loneliness, as this was a benefit mentioned by only 16% of the participants. |
|  | **Horticulture Therapy**  *(Ng K, 2018)* | Quantitative (RCT) | Horticultural therapy is the engagement of an individual in plant-based gardening activities, usually facilitated by an experienced person. Outdoor sessions were conducted at select parks, gardens, and nature reserves in Singapore | M: In-person I: One-to-one N = 59 | Horticulture therapy significantly improved positive relations with others among older adults. A significant improvement in social connectedness was observed. |
|  | **Men's Sheds Programme** *(Nurmi MA, 2018 from Noone C, 2022)* | Qualitative | The intervention called the Men's Sheds programme aimed to provide men with opportunities to socialise while participating in ongoing learning and activities. | M: In-person I: Group-based N = 64 | The intervention increased opportunities for social engagement for men who had previous experience in similar environments. |
|  | **Men's Sheds Programme** *(Reynolds KA, 2015 from Noone C, 2022)* | Qualitative | The intervention called the Men's Sheds programmed aimed to provide activities to older male adults. | M: In-person I: Group-based N = 12 | Promoted social engagement and enhanced friendships among men. |
|  | **Psychosocial Rehab** *(Routasalo PE, 2009 from Ibrahim AF, 2022)* | Quantitative (RCT) | This intervention sought to decrease loneliness by enrolling participants in various intervention options like arts, exercise, or writing. This was intended for participants 75+ years. | M: In-person I: Group-based N = 235 | There was a significant increase in friends made; however, there was no difference in loneliness and social network. |
|  | **Seniors’ Satellite Program** (Hand C, 2022) | Mixed-and/or multi-method | The Seniors’ Satellite program, catering to individuals aged 55 and older, was designed to provide affordable and accessible social and recreational activities to enhance wellness and alleviate social isolation. Offered at a low fee, the program includes physical fitness and social activities such as aerobics, yoga, dance, Pilates, crafts, and social time. The Seniors’ Satellites operate once a week in various city neighbourhoods, partnering with organizations like churches to overcome transportation and cost barriers. | M: In-person I: Group-based N = 28 | Regarding the potential impacts of attending the Satellite, no change in variables was measured at the start of attending the Satellite and after a few months, except for the size and quality of the friend network, which decreased slightly. Participants seemed to view satellite programming as an opportunity to improve and maintain their connection with others and avoid potential adverse outcomes such as social isolation. |
|  | **Social Activities Group Program** *(Nomura K, 2021)* | Mixed-and/or multi-method | A program was initiated in Japan targeting elderly males (65 years and older) to address social isolation through participation in social activities. The group-based approach comprised 120-minute sessions held weekly for 12 weeks, featuring lectures, exercises, and experiences. Participants, residing in the Tokyo metropolitan area, needed to independently access the venue and were excluded if hospitalized, living in care homes, or facing communication difficulties. The program focused on changing nine aspects crucial for engaging in social activities, with sessions structured to include discussions, learning about local resources, and planning for continued social engagement after program completion. | M: In-person I: Group-based N = 20 | The program significantly improved the independence of life but did not improve satisfaction with social activities. |
|  | **Social Engagement** *(Carandang RR, 2020)* | Quantitative (N-RCT) | Older Filipino adults at risk for depression participated in 3-hour weekly social events at the OSCA Center for three months, organized in two batches of 30–35 individuals each. The events included prayer, dancing, educational talks, group discussions, interactive games, and karaoke. Health providers delivered lectures, while peer counsellors and community health workers assisted with activities. The program covered topics such as healthy aging, nutrition, stress management, confidence building, and community resources. The primary goal was to enhance social networks and promote active social participation. | M: In-person I: Group-based N = 134 | Loneliness and perceived social support significantly improved. |
|  | **Social Farming Program** *(Gagliardi C, 2019 from Douglas NF, 2023)* | Quantitative (Cross-sectional) | This intervention involved participants averaged 72 years over the course of 1 year in one weekly session which consisted of farming as a group to promote social health. | M: In-person I: Group-based  N = 73 | There were significant positive changes in social contacts. |
|  | **The Garden Project**  *(Middling S, 2011 from Ibrahim AF, 2022)* | Quantitative | This garden project engages the neighborhood of a disadvantaged community to enhance social interaction for those 60+ years. They planned activities and got gardening supplies to plant fruits and vegetables. This occurred for the course of 3 years. | M: In-person I: Group-based N = 200 | Themes from interviews included enhanced well-being, quality of life and greater socialization. |
|  | **The Good Mood Well-being Intervention**  *(Pynnönen K, 2018)* | Quantitative (RCT) | Participants selected from three interventions they thought would benefit them the most: an exercise program, personal counseling, and a social activity program. All included social interaction. Those in the exercise and the social activity programs met weekly 19-21 times at municipal gyms. The social activity program was delivered by health care students and participants met in the city library. Activities included group discussions, self-expression using art and creative methods, and going on daytrips. Personal counseling was conducted by a rehabilitation counselor and meetings took place in a health care center. | M: In-person I: Group-based N = 1167 | Feelings of loneliness and melancholy decreased in both the intervention and control group; perceived togetherness, and attachment increased in both groups. Social integration increased significantly, but only among those who felt lonely but were not depressed. |
|  | **Volunteering and Socializing Activities in City Parks** *(Gagliardi C, 2020)* | Mixed-and/or multi-method | Older adults were recruited as volunteers for a program involving sustainable gardening practices in two city parks. Activities scheduled twice a week included cleaning less groomed areas, trail maintenance, reporting hazards, and repair work when possible. Participants were given autonomy in planning activities, aligned with municipal guidelines, to enhance motivation and prevent monotony. | M: In-person I: Self-directed  N = 19 | Social support experienced by the participants did not show a significant variation, except for increasing interactions with relatives. The qualitative findings highlighted some aspects of the experience contributed to the creation of strong relationships between participants. |
|  | **Weekday Wow Factor Project** *(Lowe JA, 2023)* | Qualitative | The Weekday Wow Factor, run by an occupational therapist, offers a range of community-based participatory activities for older adults (55 and older), including the development of a daytime disco in a city centre nightclub for participants to enjoy music and dancing. Other activities have included zip lining, trampolining, go-carting, and speedboat rides. The activities offered are based on the choices of attendees. | M: In-person I: Group-based N = 26 | The social aspect and inclusivity of the activities were paramount to participants in terms of their quality of life. Participants expressed an increased sense of belonging. |
|  | **Self-directed leisure activity interventions (n = 10)** | | | | |
|  | **Computer and Internet Access Provided** *(Mellor D, 2008 from Heins P, 2021)* | Mixed-and/or multi-method | The intervention provided internet access and use of a computer for 12 months with daily support for the first 2 weeks. | M: NR I: Self-directed N = 20 | Quantitative: At 12 months there was no significant differences in social connectedness.  Qualitative: Benefits reported in interviews was that there was positive impact on social connectedness. |
|  | **Digital Technology Interventions** *(Reviews from Adekpedjou R, 2023)* | Quantitative (Umbrella review) | Participants were community-dwelling older adults who had a mean age of 65+ years, were 65+ years, were defined as older adults, or were defined as older adults as being 50+ years. Participants were healthy or had chronic conditions or co-morbidities. Interventions were digital technologies.*  **Some intervention types included in this review may also fall into other domains; however, this classification represents the majority.* | M: NR  I: Self-directed  N = NR | Digital technologies may decrease social isolation and loneliness. |
|  | **Leisure Activity Interventions** *(Veazie S, 2019 from Freedman A, 2020)* | Quantitative (Rapid review) | Leisure activities. | M: In-person I: NR  N = NR | Leisure activities are more effective if they include exercise or social support at addressing social isolation and loneliness. |
|  | **Letter Writing to Help Others on Ageing** *(Moieni M, 2021)* | Quantitative (Cross-sectional) | This intervention involved participants aged 60 and older engaging in a 6-week assignment where they wrote about their life experiences. In the generativity condition, participants shared wisdom and advice for middle-aged adults on topics such as important life lessons. They were informed that their responses would be collected, made anonymous, and published in a book or website to help middle-aged adults. The aim was to explore the impact of writing about life experiences on health, particularly in terms of addressing loneliness. | M: Virtual (Paper) I: Self-directed N = 73 | There was no effect of the generativity intervention on post-intervention perceptions of social support (F(1,70)=2.52, p > .1) or feelings of loneliness (F(1,70)=.273, p > .6), controlling for pre-intervention values. |
|  | **Loneliness Alleviation Program (LAP)**  *(Ae-Ri J, 2023)* | Quantitative (RCT) | An integrated information and communication technology (ICT)-based intervention for community-dwelling older adults (≥ 65 years) for loneliness alleviation, including physical, psychological, and interpersonal support components. Consists of 12 sessions delivered via YouTube twice a week for six weeks, with contents consisting of smartphone usage, horticultural therapy, drug management, laughter, music, exercise, and sleep relaxation therapy. Participants watched the session content and were encouraged by a research assistant to practice and share their experiences with other participants. | M: Virtual (Smartphone) I: Self-directed  N = 40 | The experimental group had statistically significant improvements in loneliness. |
|  | **Mobile Application“GezelschApp”** *(Jansen-Kosterink SM, 2020 from Heins P, 2021)* | Quantitative (Cohort) | The mobile application GezelschApp stimulates users to engage in local activities together with other users. | M: NR I: Self-directed N = 41 | Loneliness decreased among study participants with time but was not statistically significant. |
|  | **Plant Therapy** (*Septianingtyas MC, 2023)* | Quantitative (Quasi-experimental) | This intervention was delivered to participants 60-74 years old who were part of a social service home in Indonesia but could carry out usual activities without aid. There was an empty plot of land in the social service home where participants could look after their plants. The aim was to reduce loneliness, creating a sense of calm and happiness. | M: In-person I: Self-directed  N = 32 | Overall, there was significant decrease in loneliness for the intervention group with plant therapy (p<0.000). |
|  | **The Lifestyle Engagement Activity Program (LEAP)** *(Low LF, 2015 from Poscia A, 2018)* | Quantitative (Quasi-experimental) | The 12-month program for home care clients had 3 components: engaging support of management and staff; a champion to drive practice change; and staff training. Case managers were trained to set meaningful social and/or recreational goals during care planning. Care workers were trained in good communication, to promote client independence and choice, and in Montessori activities, reminiscence, music, physical activity, and humor. | M: In-person I: NR  N = 189 | Significant increase in functional social support and decreased social isolation was observed among the home care clients. There were no significant changes in loneliness. |
|  | **Using Internet-Based Applications** *(Czaja SJ, 2015 from Todd E, 2022)* | Mixed-and/or multi-method | Using an internet-based "purpose-built application." | M: NR  I: Self-directed N = NR | There was a significant decrease in loneliness and social isolation and increased social support which was maintained at 12-months for the technological-intervention group; however, this was not maintained. |
|  | **Using Internet-Based Applications** *(Fokkema T, 2007 from Todd E, 2022)* | Mixed-and/or multi-method | Use of loaned computers. | M: NR  I: Self-directed  N = NR | There was a significant decrease in loneliness for those who were loaned computers to participate in the experimental group. |
|  | **One-to-one leisure activity interventions (n = 6)** | | | | |
|  | **Caring Callers Program**  *(Fields NL, 2023)* | Qualitative | This intervention involves offering weekly phone calls to socially isolated older adults. Developed in collaboration with a community agency in a large North Texas city, this telephone-based initiative connects older adult peer-volunteers with isolated individuals. The program addresses issues of loneliness by providing emotional support and linking participants with community resources for unmet needs such as transportation and financial assistance. Volunteers, paired with participants, engage in safety checks, share weekly events, discuss coping mechanisms, and generate conversation based on selected topics. | M: Virtual (Telephone) I: One-to-one N = 18 | Senior Companion volunteers experienced feelings of reciprocity. For some volunteers, the calls helped minimize feelings of loneliness during COVID-19. |
|  | **Foster Grandparent Programme**  *(Rook KS, 2003 from Dickens AP, 2011)* | Quantitative (Quasi-experimental) | Foster Grandparent Programme for developmentally disabled child. Older adults to have contact with child 4 hrs/day, five mornings a week. | M: In-person I: One-to-one N = 180 | For participants taking part in the Foster Grandparent Program, structural social support was significantly increased but no effect was observed for loneliness, and functional social support at one and two years compared with both control groups. The number of new relationships formed at one and two years increased, and number of new social ties at two years increased compared with both control groups. |
|  | **Letter Writing**  *(Long EM, 2023)* | Quantitative (Pre-post) | Homebound participants, aged 78-86, from a faith community engaged in a 10-week letter-writing intervention facilitated by nursing students to reduce loneliness. The initiative involved students sending introductory letters in the first week and continuing with weekly exchanges of letters, arts, crafts, and creative items. Each student was encouraged, but not required, to include a self-addressed stamped envelope if they wanted to return correspondence with the homebound member. While 34 homebound participants were involved, 18 actively corresponded with their assigned students through the letter-writing process. | M: Virtual (Paper)  I: One-to-one N = 34 | Overall, there was a significant decrease pre-intervention and post intervention for loneliness (p=0.005) . |
|  | **Senior Companion Program (SCP)**  *(Butler SS, 2006 from Hagan R, 2014)* | Mixed-and/or multi-method | This was a federal program which provides volunteer opportunities with small stipends to low-income older adults (age ≥60 years), who provide companionship and offer assistance to frail community elders. | M: In-person I: One-to-one N = 66 | Older adult volunteers had reduced loneliness. |
|  | **The Personalised Citizen Assistance for Social Participation (APIC)**  *(Levasseur M, 2016)* | Mixed-and/or multi-method | Older adults received personalized support from attendants for three hours per week over a six-month period. The attendants were non-professionals with experience working with older adults, and they underwent two days of training before starting the intervention. The attendants helped the older adults target goals for significant social and leisure activities that were difficult to accomplish, encouraging empowerment, gradual mobilization of personal and environmental resources, and community integration. | M: In-person I: One-to-one N = 16 | Quantitative results: The intervention significantly increased satisfaction with social participation, decreased difficulties in their social environment, and increased functioning autonomy and health.  Qualitative results: Social connectedness increased, and participants reported creating new or reviving social relationships. |
|  | **The Seniors Connecting: Greenvale Community Exercise Program**  *(Bartlett H, 2013)* | Quantitative (Pre-post) | The program targeted mature-aged (persons age ≥55 years) particularly socially isolated older adults. The project established a regular fitness programme based on a range of exercises, including a swimming, as well as an arts programme. It focused on building individual and community capacity by providing community transport, and training to enable seniors to manage their own activities and seek ongoing funding (e.g. accreditation for volunteer bus drivers, swim coaching, and food handling) plus provision of guest speakers on healthy ageing topics. | M: In-person I: One-to-one N = 31 | The program did not improve loneliness or social support. |
|  | **Combination of interactions leisure activity interventions (n = 8)** | | | | |
|  | **Connect 60+ Wellness Program**  *(Naseri C, 2023)* | Quantitative (Pre-post) | This 10-week intervention targets isolated individuals aged 60 and above, promoting wellness through varied activities like ballroom dancing, nature walks, and tai chi. It consists of 3-hour sessions, including 1 hour of exercise, 1 hour of wellness activities, and socializing over tea. Led by a health professional, the online delivery involves weekly exercises and wellness activities aligned with active aging guidelines. Participants complete daily wellness activities and record them in a workbook, guided by cues to action incorporating behavioral therapy and positive psychology. | M: Mixed (In-person and virtual (computer, smartphone)) I: Mixed (Group-based, self-directed) N = 47 | Overall, results show that social connectedness increased for the whole group form week 1 to week 10 (p<0.01). Whereas instrumental activities of daily living did not. |
|  | **Exercise, Activity or Counselling Intervention** *(Pynnönen K, 2018 from Ibrahim AF, 2022)* | Quantitative (RCT) | This intervention targets participants aged 60 and above, offering a choice between exercise, social activities, or counselling to promote social integration and reduce loneliness. The experimental group is given the option to participate in the supervised exercise, social activities, or personal counselling, with the underlying theory that increased social interaction and integration will decrease loneliness. The activities offered encompass a variety of options, such as arts, crafts, and day trips. | M: In-person I: Mixed (Group-based, one-to-one) N = 223 | There was a significant increase in social integration. Loneliness decreased in both groups. |
|  | **Experience Corps**  *(Fried LP, 2004 from Pool MS, 2017)* | Quantitative (RCT) | Older adult volunteers worked in elementary school classrooms, trained by the program in roles of schools’ greatest unmet needs (e.g., literacy support or support library functioning). Volunteers were trained in team building and organized into teams of 7–10 that met regularly to problem solve, plan, and socialize. | M: In-person I: Mixed (Group-based, one-to-one) N = 128 | A significant increase in the amount of social activity, number of people one could turn to for help was found. Changes in emotional support were non-significant. |
|  | **Experience Corps**  *(Morrow-Howell N, 2014)* | Quantitative (Observational) | Experience Corps is a nation-wide program that brings older adults as volunteers into public elementary schools to work with students to improve their academic achievement. | M: In-person I: Mixed (Group-based, one-to-one) N = 338 | Almost half of participants felt they made social connections through volunteering in the program; 75% of participants reported more activity engagement. They also had increased confidence in social interactions (72.2%). |
|  | **Experience Corps**  *(Parisi JM, 2015 from Pool MS, 2017)* | Quantitative (RCT) | Older adult volunteers worked in elementary school classrooms, trained by the program in roles selected by the principals as the schools’ greatest unmet needs (e.g., literacy support or support library functioning). Volunteers were trained in team building and organized into teams of 7–10 that met regularly to problem solve, plan, and socialize. | M: In-person I: Mixed (Group-based, one-to-one) N = 702 | Analysis after 12 months shows significant increase in the amount of social activities. |
|  | **Experience Corps Program** *(Carlson MC, 2008; Fried LP, 2004; Tan EJ, 2006 from Krzeczkowska A, 2021)* | Quantitative (RCTs) | Those aged 60-68 years participated in an Experience Corps Program, an intergeneration engagement program based on physical, social, and cognitive activity. This program ran 3-4 days a week and 15 hours weekly. | M: NR  I: NR  N = 128 | There was a significant effect on the number of people participants felt they could reach out to (p=0.03). However, no significant effect was found for other social outcomes (p>0.2), including the number of adults one could depend on or the number of adults seen in a typical week. |
|  | **Quartier Agil Intervention** *(Thiel C, 2022 )* | Quantitative (Pre-post) | In this proof-of-concept study, community-dwelling older adults aged 63 and older were equipped with smartphones and a specifically designed app called Quartier Agil. The participants were encouraged to engage in physically and cognitively stimulating activities in their neighborhood using the app, which also supported self-directed activities. Hot spots in the neighborhood were identified based on group discussions and smartphone GPS signals. | M: Virtual (Smartphone) I: Mixed (Group-based, self-directed) N = 39 | Quality of life and social participation did not change significantly overtime. |
|  | **Recreation, Education, and Socialization for Older Learning** **Veterans (RESOLV)**  *(Juang C, 2021)* | Mixed-and/or multi-method | RESOLV, an intervention for veterans aged 65 and older, facilitates social engagement through scheduled telephone activities, promoting socialization. Participants, including self-referred veterans and those referred by healthcare providers, undergo eligibility screening based on age, veteran status, and cognitive function. Collaborating with SCWW, RESOLV offers diverse group activities, categorized by topics such as Fun & Conversation, Hobbies and Interests, Practical Training, Reflection and Meditation, Writing Groups, Poetry, Supportive Groups, Celebrating Diversity, Special Events, and Health & Well-being Presentations. | M: Virtual (Telephone) I: Mixed (Group-based, one-to-one) N = 32 | A significant decrease in level of loneliness was found among older Veterans who participated in the program at 3-month follow up. |
|  | **Unclear interaction leisure activity interventions (n = 5)** | | | | |
|  | **Experience Corps Program** *(Gruenewald TL, 2016; Parisi JM, 2015 from Krzeczkowska A, 2021)* | Quantitative (RCTs) | This intervention was for those 60-89 years who participated in the Experience Corps program which was based on self-perceived generativity and lifestyle activity. This program was for 15 hours per week, 3-4 times per week, for 2 years. | M: NR  I: NR  N = 702 | There was a significant effect on social activity at 12-month follow-up (p<0.05) but not at 24 months. |
|  | **Recreational Activities** *(Clift S, 2012; Coffman D, 2009; Cohen GD, 2007; Davidson JW, 2014; Hillman S, 2002; Kattenstroth JC, 2013; Koga M, 2001; Solé C, 2010; Teater B, 2014; Verghese J, 2003; Yap AF, 2017; Cohen GD, 2006; Dickens AP, 2011; Moody E, 2012; Low LF, Arnetz BB, 1983; Baumgarten M, 1988; Gleibs IH, 2011; Hemingway A, 2013; Pettigrew S, 2008; Tse T, 2005; Valadez AA, 2006* from Paquet C, 2023) | Mixed-and/or multi-method | Music intervention groups comprised a large part of this category. The category also included leisure activities in social groups and selecting activities to promote autonomy. | M: NR  I: NR  N = NR | This intervention category was found to mostly reduce social isolation and loneliness. |
|  | **REPRINTS** *(Fujiwara Y, 2009 from Krzeczkowska A, 2021)* | Quantitative (Quasi-experimental) | This intervention was for those 60-69 years old who took part in volunteering in the REPRINTS intervention. The aim was to assess social support, social function, and social participation. The sessions were once per 1-2 weeks and involved reading and playing for 30 minutes with a kindergarten class and reading picture books for 15 minutes per class. This went on for 18 months. | M: NR  I: NR  N = 141 | There were significant results for providing support to friends (p=0.046), receiving social support (p=0.038), social networking scores (p=0.007), number of distant friends (p=0.044), and contact with children outside of neighborhoods through volunteering (p<0.001). |
|  | **REPRINTS** *(Sakurai R, 2016 from Krzeczkowska A, 2021)* | Quantitative (Quasi-experimental) | This study was focusing on the long-term effects of REPRINTS focusing on functional capacity. This was for 1 session per 1-2 weeks for 7 years. The sessions were once per 1-2 weeks and involved reading and playing for 30 minutes with a kindergarten class, reading picture books for 15 minutes per class. | M: NR  I: NR  N = 118 | There were no significant effects on social functioning and frequency of interacting with friends. |
|  | **The Healthy Aging Web-Based Activity Programme** *(Cohen-Mansfield J, 2021 from Li M, 2023)* | Qualitative | Participants were part of the Healthy Aging organization which included mindfulness, coping skills, social connection, and seated physical exercise components for 5 days per week. Each activity lasting for 30 minutes. | M: Virtual (Not Specified) I: NR  N = 49 | There was insufficient social contact provision, and 16% of participants reported that the intervention prevented loneliness. |
| **Mind-body interventions**  **(n=14)**  *Mind-body interventions focus on the interactions among the brain, mind, body, and behaviour (Morone NE, 2007). These interventions can include biofeedback, progressive muscle relaxation (PMR), meditation, guided imagery, gratitude, hypnosis, tai chi, qi gong, and yoga.* | **In-person mind-body interventions (n = 11)** | | | | |
|  | **Compassion Meditation (CM)** *(Malaktaris A, 2022)* | Mixed-and/or multi-method | The study involved participants aged 66-73 in a 10-week Compassion Meditation (CM) program based on the Tibetan Lojong tradition, emphasizing mind training for stabilizing emotions and fostering inner satisfaction. The 90-minute sessions covered various aspects, including introduction, focus, attention, awareness, positive reflection, interconnectedness, gratitude, empathy, and integration. Participants practiced meditation between sessions, recording time and emotions in daily journals, with increasing meditation times each week. | M: In-person I: Group-based N = NR | Significant pre- to post-intervention improvements in satisfaction with life and resilience were observed. Although not statistically significant (p > .05), there were small effect size reductions in loneliness (d = .31) and social connectedness (d =.26). Participants’ open-ended descriptive responses indicated self-perceived improvements in interpersonal skills. |
|  | **Daily Gratitude Exercise**  *(Bartlett MY, 2019)* | Quantitative (RCT) | Older adults living in subsidized housing were asked to complete a daily gratitude exercise for 20 days, consisting of listing to three good things that happened that day and why they happened. | M: In-person I: Self-directed N = 42 | Gratitude cultivation decreased loneliness and subjective well-being and boosted self-reported health. |
|  | **Dance Movement Therapy (DMT)** *(Ho RT, 2020)* | Quantitative (RCT) | DMT is a mind-body intervention that integrates exercise and psychosocial therapeutic components and was based on four elements which was simple dance, movement, improvisation, and dancing with other members. This intervention emphasized expressions, social connection, and creativity. At the end of the session there was group discussion to express feelings on their experience in the session. The session was 12 weeks long and 1-hour sessions in groups for participants 79 years on average who have mild dementia. | M: In-person I: Group-based N = 204 | There was a significant decrease in loneliness compared to control (no intervention) (p <0.01). |
|  | **Laughter Yoga** *(Kuru N, 2018 from Alici NK, 2020)* | Quantitative (Quasi-experimental) | This intervention involved individuals aged 65 and older participating in laughter yoga led by a certified instructor for 10 sessions over 5 weeks (twice per week). The sessions included introductions, warm-ups, breathing exercises, laughter exercises, games, singing songs, and playing with balloons. Laughter yoga, a non-invasive and non-pharmacological method, incorporates yoga breathing techniques and laughter exercises. Simulated laughter, initiated as a physical exercise with eye contact and group engagement, is indistinguishable to the body from genuine laughter, leading to the onset of laughter. | M: In-person I: Group-based N = 50 | The social loneliness scores decreased significantly in the intervention group compared to the control group. However, total loneliness did not significantly change. |
|  | **Meditation Program** *(Pandya SP, 2021)* | Quantitative (RCT) | Older adults underwent 45-minute weekly classes of a customized meditation program for two years to examine the impact of the program in mitigating loneliness and promoting well-being as compared to the control group who underwent no intervention. The meditation program was designed by meditation training experts with experience developing customized programs for older adults. The key features of the classes led by an instructor were (i) postures interspersed with relaxation, (ii) slowness in movements and (iii) inner watchful awareness. Home practice was encouraged. | M: In-person I: Group-based N = 378 | Loneliness, well-being, and life satisfaction significantly improved over time (p < 0.01). |
|  | **Mindfulness Based Stress Reduction Program (MBSR)**  *(Creswell JD, 2012 from Hagan R, 2014)* | Quantitative (RCT) | The program was administered to healthy older adults by one of three trained clinicians over three cohorts and consisted of eight weekly 120-min group sessions, a day-long retreat in the sixth or seventh week, and 30-min of daily home mindfulness practices. During each group session, an instructor leads participants in guided mindfulness meditation exercises, mindful yoga and stretching, and group discussions with the intent to foster mindful awareness of one’s moment-to-moment experience. The daylong 7-h retreat focused on integrating and elaborating on the exercises learned during the course. Participants were also asked to participate in 30 minutes of daily home mindfulness practice six days a week | M: In-person I: Group-based N = 40 | Healthy older adults had significantly reduced loneliness after the MBSR intervention. |
|  | **Preventing Loss of Independence through Exercise (PLIE)** *(Chao LL, 2021)* | Quantitative (Pre-post) | PLIE classes, designed for people with mild-to-moderate dementia, spanned 12 weeks, with twice-weekly one-hour sessions led by two instructors incorporating breathing and movement exercises. The classes aimed to address social isolation and well-being, fostering participant interaction through mindful body awareness exercises, and concluding with opportunities for expressing gratitude or happiness. The Preventing Loss of Independence through Exercise (PLIE) program integrated principles from established traditions such as the Feldenkrais Method®, Rosen Method, Tai Chi, and yoga while incorporating elements from occupational therapy, physical therapy, and dance movement therapy. | M: In-person I: Group-based N = 18 | Participants reported a significant decrease in social isolation (p=0.02) and significant increases in well-being (p=0.03). |
|  | **Tai Chi Easy (TCE)** (Larkey LK, 2023) | Quantitative (Pre-post) | This intervention targeted participants aged 55-80, focusing on a 50-minute Tai Chi session led by a senior instructor. The sessions emphasized rhythmic motion, synchronized breathing, meditation, and a connection to nature. Participants were encouraged to stay present in the moment, focusing on breath, body, and connection to nature. Chairs were available for those unable to stand, and breaks were encouraged. | M: In-person I: Group-based N = 21 | Total connection improved significantly overtime (p = .01) |
|  | **Tai Chi Intervention**  *(Taylor-Pillae RE, 2006 from Pool MS, 2017)* | Quantitative (Quasi-experimental) | A group intervention providing physical activity through Tai Chi for 12-weeks to community dwelling Chinese older adults with cardiovascular disease | M: In-person I: Group-based N = 39 | Participants had significantly improved social support after the Tai Chi intervention. |
|  | **Yoga for Loneliness** *(Panigrahi M, 2023)* | Quantitative (Pre-post) | This yoga program was for participants between the ages 60-80 years over the course of 12 weeks which consisted of lectures, exercise, breathing, chanting, and meditation. Loneliness was evaluated before and after the program. | M: In-person I: Group-based  N = 44 | There was a significant decrease in loneliness pre-test to post-test (p=0.01757). |
|  | **Yoga Intervention**  *(Wang DS, 2010)* | Quantitative (Pre-post) | 8 yoga sessions held twice a week for four weeks. Each session lasted 1 hour and consisted of 45 minutes of posturing and breathing and 15 minutes of relaxation and teachings. The yoga intervention was held on-site at the low-income senior’s residents housing building. The socialization comparison group attended hour-long sessions twice a week for four weeks where they were shown movies that were administered by the researcher. The socialization group were asked to continue with their daily routines and activities | M: In-person I: Group-based N = 18 | The yoga group showed improvement from pre- to post-test in the measure of loneliness, but the change was not significant. |
|  | **Virtual mind-body interventions (n = 3)** | | | | |
|  | **Remote Chair Yoga (CY)** *(Park J, 2022)* | Quantitative (Pre-post) | This intervention targeted home-based older adults with dementia during the pandemic, comprising 60-minute sessions held twice weekly for 8 weeks. The sessions commenced with a 5-minute socialization period, utilizing digital gallery view on Zoom to enhance social connection. During the yoga segment, the interventionist was spotlighted to minimize distractions. Participants attended remotely supervised sessions, encompassing check-ins, breathing exercises, physical postures, guided relaxation, and wrap-ups. Certified yoga interventionists led each session, with caregivers ensuring participant safety and pose accuracy. | M: Virtual (Computer) I: Group-based  N = 11 | Overall, there was no significant change in social loneliness and there was a significant increase in emotional loneliness from baseline to post-intervention (p=0.016). |
|  | **Spiritual Counselling Programme (SCP)** *(Pandya SP, 2021)* | Quantitative (Pre-post) | Older empty nesters participated in a year-long, 24-session mindfulness and emotional awareness program led by spiritual trainers to reduce loneliness. Sessions, conducted online biweekly, involved couples sharing feelings, centring exercises, and mindfulness activities. The program, developed with input from spiritual experts and family counselors, aimed to help couples reflect on emotions, alleviate loneliness, and enhance affect balance. Home practice, crucial for the intervention, included posting weekly self-reflections on an online platform. | M: Virtual (Computer) I: Mixed (Group-based, pair-based, self-directed) N = 822 | Loneliness significantly decreased in participants. |
|  | **Virtually Mentally Stimulating Activities (VMSA) Program**  *(Weaver C, 2022)* | Quantitative (Cross-sectional) | Community-dwelling older adults aged 60 and above participated in a Virtually Mentally Stimulating Activities (VMSA) program, engaging in challenging and novel cognitive activities. The program incorporated a variety of exercises, starting with a breathing exercise warm-up, followed by motor tasks and cognitive challenges such as writing with the non-dominant hand and recalling childhood living spaces. Short cognitive exercises, lasting 2-10 minutes each, targeted multiple cognitive domains, including visual-spatial skills, executive function, language, and sensory stimulation. Participants engaged in debrief sessions to discuss their experiences after each session. | M: Virtual (Computer) I: Group-based N = 4 | There was a positive mean change in loneliness and satisfaction with life scores across all participants; however, no significance test was conducted. |
| **Physical activity or exercise-based interventions**  **(n=48)**  *Physical activity or exercise-based interventions encourage participants to engage and enhance physical activity and movement to improve their health status and functional performance (Van der Bjj A, 2022)* | **Non-technology-mediated physical activity interventions (n = 24)** | | | | |
|  | **Aerobic Intervention**  *(McAuley E, 2000 from Cohen-Mansfield J, 2015)* | Quantitative (RCT) | Formerly sedentary adults 60-75 involved in an intervention with two forms of aerobic activity (walking, stretching, and toning) sessions conducted three times a week for 6 months. | M: In-person I: Group-based N = 174 | Loneliness significantly decreased in participants of both programs of aerobic exercise but increased at 6-month follow-up. |
|  | **Connecting Seniors to Their Community Through Walking Program** *(Walters K, 2022)* | Qualitative | Older adults (65 years or older) participated in an 8-month monthly walking program, completing at least six walks. Divided into groups by walking ability, participants were led by research assistants, and walks were tailored to their needs, utilizing local transportation. Each session included sign-in, distribution of walking accelerometers, health materials, and nametags. Group leaders provided instructions, and participants used local transportation together. Lunch was provided for socializing. | M: In-person I: Group-based N = 28 | Older adults participating in the CSCW program reported increased feelings of connection with their community. |
|  | **ENJOY project** *(Levinger P, 2020)* | Quantitative (Prospective controlled trial) | People (aged ≥ 60 years) underwent a 12-week structured supervised physical activity program using outdoor exercise park equipment followed by six months of unstructured independent use of the exercise park. The effects of sustained engagement of physical activity on mental, social, and physical health were evaluated. | M: In-person I: Group-based N = 95 | A significant increase in self rated quality of life (p = 0.04), well-being (p < 0.01), and loneliness (p = 0.03) was demonstrated following the intervention. No significant changes were demonstrated in social isolation (p > 0.05). |
|  | **Exercise for Those with Dementia** *(Long A, 2020)* | Mixed-and/or multi-method | This intervention targeted individuals with dementia, offering 1-hour sessions led by a physiotherapist and trained volunteers. The class included a warm-up, followed by smaller group circuit workouts with rotating stations and concluded with a group balance exercise. The study, utilizing mixed-methods, aimed to explore the impact of this dementia-friendly exercise class on participants and their carers. | M: In-person I: Group-based N = 16 | Following a three-month attendance at a dementia-friendly exercise class, there were improvements in loneliness for all participants. Activities of daily living and quality of life showed small deterioration of less than one percent. |
|  | **Exercise Intervention** *(Ho RT, 2020)* | Quantitative (RCT) | This intervention was for participants 79 years on average (with mild dementia) consisting of a moderate exercise program. This was conducted by a trained fitness professional. The session consisted of a warm-up, stretching, exercise, and cool down. The session was 12 weeks long and 1-hour sessions in groups. | M: In-person I: Group-based N = 204 | There was not a significant decrease in loneliness compared to control (no intervention) (p=0.91). |
|  | **Floorball (FB)** *(Pedersen MT, 2022)* | Mixed-and/or multi-method | The study involved participants aged 69-81 engaging in small-sided floorball games, conducted 5 times per week for 5 years, with a 5-year follow-up from baseline. The floorball sessions were 60 minutes long, comprising 5 small-sided games (5v5 or 6v6) on a 13x20m wooden surface, using plastic sticks. Each game lasted 8 minutes, separated by a 4-minute rest period, preceded by a 10-minute warm-up. The training was self-organized without researcher interference, and participants had the opportunity to play three times a week. | M: In-person I: Group-based  N = 29 | The present study showed that 5 years of floorball training performed twice a week in a municipality setting led to increased social capital in elderly men. |
|  | **General Physical Activity Interventions**  *(Veazie S, 2019 from Freedman A, 2020)* | Quantitative (Rapid review) | General physical activity interventions. | M: In-person I: Group-based N = NR | Physical activity interventions are more likely to be successful to address social isolation and loneliness if they have a health care provider involved in implementation and are delivered more than once weekly |
|  | **Gippsland Health and Well-being Program** *(Dabkowski E, 202 )* | Qualitative | A free community-based fitness program, aiming to improve the health and wellness of adults of varying levels of mobility. These 60-minute classes were facilitated by a qualified exercise therapist consisting of mobility, balance, and Tai Chi–based exercises, followed by a social and educational component. Participants socialised after the class, sharing morning tea and trading stories. | M: In-person I: Group-based N = 23 | Participants reported improvements in social connections. |
|  | **Group Fitness Program** *(Bidonde MJ, 2009 from Johnstone G, 2021)* | Qualitative | This intervention involved a group fitness program specifically designed for older women. Participants were compensated for their involvement in this program, which ran twice a week for 60 minutes per session. The program was led by instructors but administered by the participants themselves, emphasizing participant ownership. | M: In-person I: Group-based N = 9 | Involvement in the programme improved social networks and health. |
|  | **Group Outdoor Health Walks (GOHW)** *(Irvine KN, 2022)* | Qualitative | This intervention, targeting individuals aged 50-80, aimed to promote well-being through guided outdoor health walks (GOHWs) in a natural environment. Typically organized by community or third-party agencies, these walks were designed as socially supportive activities lasting for 1 hour over a 12-week period. GOHWs are defined as "short, safe, social, local, low level, led walks" primarily for relatively inactive individuals who could benefit from increased physical activity. These non-health service interventions are led by locally trained volunteer walk leaders. | M: In-person I: Group-based N = 9 | Several participants identified combating loneliness or social isolation as a reason why the group walks were good for people’s well-being. As a result of being part of the walking group, which enabled participants to engage in meaningful social interactions with others in the community, our interviewees demonstrated increased social well-being. |
|  | **Health Behaviour Interventions/ Health Education Interventions** *(McKay H, 2018; Mendoza-Ruvalcaba NM, 2016 from Tcymbal A, 2025)* | Mixed-and/or multi-method | This mixed method systematic review investigates the effects of interventions that promote physical activity and social participation among community dwelling older adults (65 years and older) and includes health behaviour interventions and health education. | M: In-person I: NR  N = NR | Quantitative: One study reporting on the “Choose to Move” program showed positive effects on social participation while another study reporting on the “Vital Aging” program had no effect on social participation. |
|  | **Healthy Ageing Promotion Program for You (HAPPY)** *(Merchant RA, 2021)* | Quantitative (Cross-sectional) | Participants 60+ were led by an exercise leader for 60 minutes once or twice per week (depends on the site). The multi-component program encompassed physical, cognitive, and social activities. The objective was to engage frail older adults that may or may not have cognitive impairment in a dual-task exercise program to help with social isolation. The exercises include low to moderate-intensity circuit and resistance training, focusing on physical function. | M: In-person I: Group-based  N = 700 | Overall, there was a significant improvement for social isolation, perceived health and frailty status. |
|  | **Leveraging Exercise to Age in Place (LEAP)** *(Mays AM, 2021)* | Quantitative (Pre-post) | Community-based group health classes were attended by older adults (age ≥ 50) who selected either Arthritis Exercise, Tai Chi for Arthritis, EnhanceFitness, or the Healthier Living Workshops. The intervention aimed to impact loneliness and social isolation. Instructors from two community-based non-profits taught all workshops. | M: In-person I: Group-based N = 382 | Loneliness and social connectedness improved significantly at 6-months (p < 0.001). |
|  | **Physical Activity Promotion Interventions** *(Barbosa BT, 2019; Barragan C, 2021; Bidonde MJ, 2009; Brustio PR, 2018; Cedergren A, 2007; Chan AW, 2017; Dionigi R, 2007;Ehlers DK, 2017; Figueira HA, 2012; Gomeñuka NA, 2019; Kohut ML, 2006; Komatsu H, 2017; Liu YWJ, 2013; Maki Y, 2012; McAuley E, 2000; Streber A, 2017; Wang DS, 2010; Wikman JM, 2017 from Tcymbal A, 2022)* | Mixed-and/or multi-method | This mixed method systematic review investigates the effects of interventions that promote physical activity and social participation among community dwelling older adults (65 years and older) and includes physical activity promotion interventions. | M: In-person I: NR  N = NR | Quantitative studies: Of the 13 quantitative studies, 53.3% reported positive associations between participating in the intervention and improvements in social participation outcomes. Qualitative studies: Participants from the five qualitative studies reported that involvement in the programs gave them the opportunity to feel more social engagement and social support, be more socially connected, and expand their communication. |
|  | **Self-Organized Swimming Groups for Healthy Ageing** *(Costello L, 2019)* | Qualitative | This program in Perth, Australia, focused on older adults participating in informal swimming groups with the goal of promoting healthy aging. The leisure-based and informal nature of the program was often perceived by participants as low-impact outdoor exercise. The research focused on the experiences of wild ocean swimming, particularly at iconic beaches, these beaches, known for their wide sandy shorelines and limited rocky outcrops, provided uninterrupted swimming. | M: In-person I: Group-based N = 17 | The ocean swimming groups, shared a common belief in the social and well-being benefits of swimming together. This shared practice served as a meaningful leisure activity, contributing to self-efficacy and resilience among participants, and played a crucial role in fostering group cohesion, social connectedness and positive health and well-being. |
|  | **Silver Sneakers Exercise Program**  *(Brady S, 2020)* | Quantitative (Quasi-experimental) | An exercise program available to older adults (age ≥65 years) as part of many Medicare Advantage and Medigap plans that provides gym memberships and offers specialized group exercise classes at no additional cost. | M: In-person I: Group-based N = 3143 | Members of the SilverSneakers program were less likely to experience feelings of loneliness, which was associated with improved health. Members were also less likely to be socially isolated. |
|  | **Social Activity with Physical Activity Component** *(Austin EN, 2006; Boyes M, 2013;Gagliardi C, 2019; Johnson JK, 2020; Vadineia da Silva M, 2016 from Tcymbal A, 2022)* | Mixed-and/or multi-method | This mixed method systematic review investigates the effects of interventions that promote physical activity and social participation among community dwelling older adults (65 years and older) and includes interventions with a social activity and physical activity component. | M: In-person I: NR  N = NR | Quantitative studies: Three of the four quantitative studies (75%) reported positive effects of the interventions on social participation outcomes. Qualitative studies: Analysis showed that participants in the two qualitative studies noticed improvements in their social participation. |
|  | **Steps for Change** *(Rodriguez Espinosa P, 2023)* | Qualitative | A targeted physical activity intervention, coupled with a community engagement strategy focused on evidence-based improvements in local environments, was implemented for older adults residing in senior housing. | M: In-person I: Group-based N = 35 | Participants reported both new and strengthened social connections with others because of their participation in Steps for Change. |
|  | **Supervised Activities** *(Pynnönen K, 2018 from Douglas NF, 2023)* | Quantitative (Cross-sectional) | This intervention incorporated supervised exercise, counselling, and social activities to reduce loneliness over the course of 6 months in 19-20 sessions of exercise and 4-5 sessions for counselling participants 77 years on average to decrease social isolation. | M: In-person I: Mixed (Group-based, one-to-one) N = 105 | There was a significant positive association for social integration. |
|  | **Supervised Seniors Exercise Park Program** *(Ng YL, 2023)* | Qualitative | This qualitative study aimed to explore the experiences of older adults with mild balance dysfunction participating in an 18-week supervised Seniors Exercise Park program, followed by 6 weeks of unsupervised independent practice. The Seniors Exercise Park within the retirement village provided exercise equipment, and participants were guided by a physical therapist during supervised sessions twice weekly for 18 weeks. The sessions included a 5-minute warm-up, up to 50 minutes of exercises using equipment, and a 5-minute cool-down. The last three supervised sessions included briefings and handouts on exercise and safety instructions for independent practice. | M: In-person I: Group-based N = 24 | All participants liked training in a group because they viewed this as a social activity and an opportunity to interact with their peers. By the end of the 18-week intervention, they valued the friendships that had developed. |
|  | **The Choose to Move (CTM) Intervention**  *(McKay H, 2018)* | Quantitative (Controlled before and after) | The intervention for older adult participants who were considered physically inactive pre-intervention was comprised of: (1) a 60-min one-to-one consultation with Activity Coaches; (2) participants attended four 60-min Motivational Group Meetings (1x in months 1–2; 2x in month 3) to connect socially with other participants (max 12/group) and their Activity Coach; (3) Activity Coaches called participants regularly by phone (15 min/call on average) to monitor progress, address challenges and modify the Action Plan as needed (3x in month 1; 2x in months 2 and 3; 1x in months 4–6). Active phase participants received a greater “dose” of Activity Coach support (one-to-one meeting, seven telephone calls, 4 group meetings) compared to the maintenance phase (3 telephone calls. | M: In-person I: Group-based N = 458 | Participants had significantly reduced loneliness post-intervention. Older participants reported greater improvements in loneliness than younger participants. Social exclusion significantly reduced for younger participants. However, there were no changes in social exclusion in older participants. |
|  | **The Physical Activity Intervention for Loneliness (PAIL)**  *(Shvedko AV, 2020)* | Mixed-and/or multi-method | The intervention included once-weekly group walk and health education workshops up to 90 minutes per session for 12 weeks. | M: In-person I: Group-based N = 25 | Qualitative results: The intervention led to no changes in loneliness but non-significant improvements in friendship development  Qualitative results: Participants felt that the walks promoted bonding and increased their desire for more friendships. |
|  | **Walk 'n' Talk intervention**  *(Hwang J, 2019)* | Qualitative | The 12-week community-based program, included socialization, health education, falls prevention exercise, and walking. It was held twice a week in local community centers and seniors’ residence buildings, with a 45-minute fitness program, a 30-minute pedometer-based group walk, a 60-minute interactive health education session, and 20-minute open socialization. The sessions were facilitated by a program coordinator and interdisciplinary volunteer groups of undergraduate students. | M: In-person I: Group-based N = 16 | The intervention had a positive impact on feelings of loneliness. Participant’s interactions with volunteers and the group activities provided a sense of belonging, and a positive impact on their psychosocial well-being. |
|  | **Walking Exercise Program** *(Rejeski W, 2014 from Pool MS, 2017)* | Quantitative (RCT) | Group-mediated, home-based physical activity intervention, combined with cognitive behaviour education in community dwelling older adults of minority groups with peripheral artery disease. The intervention included exercise plus advice/education during weekly 90-minute group sessions, as well as one-to-one feedback. | M: In-person I: Group-based N = 178 | Older adults had significantly improved social functioning after the group-mediated, home-based activity intervention (p=0.0008). |
|  | **Technology-mediated physical activity interventions (n = 16)** | | | | |
|  | **Active Plus**  *(Boekhout JM, 2021)* | Quantitative (RCT) | The Active Plus intervention was for chronically ill participants 65 and older who are living alone. The systematically developed computer-tailored intervention consisted of 3 pieces of advice to get participants physically active over four months. The first is based on the awareness of the benefits of being physical and of local exercise resources, the second is understanding the social benefits of being physically active, and lastly, planning physical activities in the participants' calendars (action planning). | M: Virtual (Computer) I: Self-directed N = 585 | Total and social loneliness significantly improved. However, there was no significant differences in emotional loneliness. |
|  | **Choose to Move (CTM)** *(McKay HA, 2023)* | Quantitative (Pre-post) | Choose To Move (CTM) supports individuals aged 60 and above in establishing personalized physical activity goals and overcoming barriers. Participants receive social support from peers and coaches, with assessments focusing on reducing social isolation, loneliness, and improving health-related quality of life. The 6-month program involves an initial intensive phase with a one-on-one consultation, motivational group meetings, and check-ins, followed by a reduced support phase in the last 3 months involving three telephone calls from coaches. | M: Virtual (Computer) I: Mixed (Group-based, one-to-one) N = 1012 | Social isolation and loneliness significantly decreased in all participants (at baseline-6 months). Health related quality of life not improve. |
|  | **Computer Exercise Advisor**  *(Bickmore TW, 2005 from Cohen-Mansfield J, 2015)* | Quantitative (RCT) | One-to-one intervention with a strong technological focus involving a computerized relational agent that was an exercise advisor. | M: Virtual (Computer) I: One-to-one N = 21 | There were no significant differences between the two groups on loneliness either before or after the intervention. |
|  | **Exercise Group** *(Düzel S, 2022)* | Quantitative (Pre-post) | Exercise group participants engaged in home-based moderate aerobic exercise three to four times per week using a bicycle ergometer (DKN Ergometer AM-50) and a Bluetooth-linked tablet with personalized interval training. Initial training duration was 30 min at individually set intensity, increasing by 3 min per interval and 3 to 4 watts approximately every 2 weeks. Participants could provide feedback on difficulty, and intensity was remotely adjusted. The exercise group also participated in weekly 1-h group sessions of toning and stretching led by an external instructor. | M: Virtual (Tablet) I: Mixed (Group-based, self-directed) N = 39 | There was no significant difference between the exercise and non-exercise groups over time for well-being (p=0.47) and loneliness (p=1.98). |
|  | **Exergaming**  *(Zhu YZ, 2023)* | Quantitative (Quasi-experimental) | In an eight-week exergaming intervention for older adults with cognitive frailty, participants engaged in group sessions twice a week, involving warm-up, exergaming activities targeting various physical and cognitive functions, and cool-down exercises. The study aimed to investigate the effects of exergaming on cognitive function and loneliness, with the hypothesis that it could improve cognitive functions and reduce loneliness in this population. | M: Virtual (Computer, television) I: Group-based N = 69 | Overall, the control group (receiving usual care and not placed under activity restrictions) had significantly improved loneliness compared to the intervention group. The intervention groups loneliness significantly increased during the intervention. |
|  | **Personalized Fall Prevention Exercise Program**  *(Baez M, 2017)* | Quantitative (RCT) | An intervention based on the “Otago” program for fall prevention for eight weeks. Older adults (age ≥65 years) living independently with non-to-low levels of frailty could join online group exercises using a tablet-based application. Participants were assigned either to the Control group, representing the traditional individual home-based training program, or the social group, representing the online group exercising program. | M: Virtual (Tablet) I: Group-based N = 37 | Both groups demonstrated a non-significant improvement from baseline in subjective well-being and non-significant decrease in loneliness. However, there was a correlation between the number of private messages sent and decrease in loneliness score for the experimental group. |
|  | **Personalized Online Exercise Programme** *(Zengin Alpozgen A, 2022 from Li M, 2023)* | Quantitative (RCT) | Older adults in urban areas engaged in personalized physical activities conducted three times a week for a period of 6 weeks, with each session lasting 40-45 minutes. | M: Virtual (Not Specified) I: NR  N = 30 | Loneliness worsened in the control group (P = 0.016), but there was no significant difference in the study group (P = 0.162). |
|  | **Playing Wii** *(Kahlbaugh PE, 2011 from Heins P, 2021)* | Quantitative (Clinical controlled trial) | The intervention examined using Wii for 1 hour per week with an undergraduate student for a 10-week period. | M: In-person I: One-to-one  N = 36 | Significant decrease in loneliness from pretest to posttest in intervention group (p < 0.005) with an increase in loneliness in comparison group. |
|  | **Tablet for Exercise** *(Báez M, 2016 from Rivera-Torres S, 2021)* | Quantitative (RCT) | This intervention involved providing participants aged 65 and above with tablets in their homes, enabling them to engage in virtual classrooms with messaging capabilities, video chat, and web-based live exercise classes. The goal was to enhance social participation and communication, allowing older adults to interact with instructors and fellow participants in virtual spaces. | M: Virtual (Computer) I: Group-based N = 37 | There was no significant effect on loneliness. |
|  | **Tele-Exercise Program** *(Alpogen AZ, 2022)* | Quantitative (RCT) | Older adults (65 and above) in the intervention group received an 18-session online personalized exercise program over 6 weeks, guided by a physiotherapist. The exercise program included a 5-minute warm-up and cool-down, incorporating exercises for muscle endurance, flexibility, and balance, aligned with WHO physical activity recommendations for older adults. | M: Virtual (Computer) I: Group-based N = 30 | Overall, the intervention group demonstrated a significant difference in total scores of health-related quality of life (p < 0.05). Loneliness scores worsened in the control group (p = 0.016), while in the intervention group, there was no significant difference (p = 0.162). Notably, differences in loneliness were found to be significantly different between the two groups (p < 0.05). |
|  | **Using the Internet for Applications with a Phone and Tablet** *(Baez M, 2017 from Todd E, 2022)* | Mixed-and/or multi-method | This intervention was a simulated gym program that was accessed through a tablet or phone application. Participants were able to message each other and connect. | M: Virtual (Smartphone, tablet) I: NR  N = NR | Overall, loneliness did not significantly decrease. |
|  | **Virtual Space Messaging** *(Isaacson M, 2019 from Rivera-Torres S, 2021)* | Mixed-and/or multi-method | This intervention targeted individuals aged 75 and above, aiming to enhance social participation and communication. It involved creating a virtual space with messaging capabilities and video chats, utilizing technology such as TV, remote controls, and webcams. Approximately 5% of the studies implemented web-based live exercise classes, enabling older adults to interact virtually with instructors and fellow participants. | M: Virtual (Computer) I: Group-based N = 40 | Participants exhibited less loneliness and increased social engagement. |
|  | **Wii Exergame** *(Li J, 2017 from Choi HK, 2021)* | Quantitative (N-RCT) | Wii Exergame, designed for community-dwelling elderly individuals aged 65 and above, integrates video games with exercise to reduce loneliness and improve quality of life. Tailored to the interests and physical conditions of the elderly, five new exergames aim to enhance overall well-being. | M: Virtual (Wii)  I: Self-directed  N = 30 | There were no significant decreases in loneliness, life satisfaction, or self-efficacy. |
|  | **Wii Fit U**  (Chao Y, 2018 from Li J, 2018) | Quantitative (Pre-post) | Balance games, yoga poses, strength training, aerobics, and dance games for older adults (mean age 64.17). | M: In-person I: NR  N = 12 | Wii Fit U program encouraged participants to get connected with others. |
|  | **Wii Kinect Exercise Games**  *(Xu X, 2016 from Li J, 2018)* | Quantitative (Pre-post) | Wii Kinect Games among older adults (mean age 75 years) at senior’s activity centres. | M: In-person I: Group-based N = 89 | A significant increase in sociability and a significant decrease in loneliness was observed, although little differences were found across different play types or age groups (young-old vs old-old). Social anxiousness did not change significantly across all participants, except in the young-old participants who played exergames with youths. |
|  | **Wii Sports + Wii Sports Resort Games**  *(Wollersheim D, 2010 from Chao YY, 2015)* | Quantitative (Pre-post) | Archery, table tennis, bowling, boxing, cycling, frisbee, golf, sword fighting, tennis for females (mean age 73.5) with a disability or who are socially isolated. | M: In-person I: Group-based N = 15 | The Wii games provided increased social connections within the group and to participants’ family members. An improved sense of social well-being was reported after the 6-week intervention. |
|  | **Combination of technology-mediated and non-technology-mediated physical activity interventions (n = 3)** | | | | |
|  | **Choose to Move (CTM)** *(Franke T, 2021)* | Mixed-and/or multi-method | The community based Choose to Move (CTM) intervention targets adults aged 60 and above with low physical activity (PA). Over six months, CTM promotes social connectedness and reduces loneliness by implementing personalized physical activity plans. The program includes one-on-one consultations, regular phone check-ins, and motivational group meetings led by an activity coach. Participants set personal PA goals, create action plans, receive social support, and engage in educational content. | M: Mixed (In-person and virtual (telephone)) I: Mixed (Group-based, one-to-one) N = 458 | Loneliness scores at baseline were significantly different between participants identifying as lonely and not lonely, with a significant decrease in loneliness observed at 3 and 6 months for those initially identifying as lonely, while the 'not lonely' group showed no change at 3 months and a significant increase at 6 months compared to baseline. |
|  | **Choose to Move (CTM)** *(McKay HA, 2021)* | Quantitative (Cross-sectional) | Participants 60+ who were enrolled in the Choose to Move (CTM) intervention were evaluated whether the benefits of social connectedness was maintained. CTM is a physical activity intervention that is based on a 60-minute consultation, motivational group meetings, and telephone check-ins over the course of 6 months. In this evaluation data was used at baseline, 6, and 18 months after the intervention. | M: Mixed (In-person and virtual (telephone)) I: Mixed (Group-based, one-to-one) N = 458 | Among the younger participants (60-74), the social isolation score was higher (indicating lower social isolation) at 18 months compared with baseline (+0.7; 95% CI, 0.2 to 1.1; P = .001). Among the older participants (75+), the social isolation score at 18 months was significantly lower (indicating greater social isolation) as compared with baseline (−0.7; 95% CI, −1.4 to −0.05; P = .033). Loneliness did not significantly change in any participants. |
|  | **SITLESS Intervention** *(Blackburn NE, 2021)* | Qualitative | SITLESS is a two-part multi-country study aimed at enhancing physical activity and social inclusion in community-based individuals aged 65 and above. The intervention includes a 16-week exercise referral scheme (ERS) with two weekly sessions led by trained professionals, accompanied by seven self-management strategy (SMS) sessions and four professional calls over 30 weeks to promote physical activity goals. The project, recognized as a complex intervention, incorporates various components such as structured exercise, one-on-one visits, group sessions, and telephone follow-ups. | M: Mixed (In-person and virtual (telephone)) I: Mixed (Group-based, one-to-one) N = 150 | Participants perceived their sense of belonging as increasing. Social relationships, social health, and capacity for activities of daily living improved. |
|  | **Unclear mode of delivery physical activity interventions (n = 5)** | | | | |
|  | **Exercise and Social Engagement Interventions** *(Baez M, 2017; Chan AW, 2017; Larsen RT, 2021; Li J, 2017; from Yu DS, 2023)* | Quantitative (RCT, N-RCT) | This systematic review compared the effects of diverse non-pharmacological interventions, such as exercise and social engagement interventions, on loneliness in community-dwelling older adults*.  **Some intervention types included in this review may also fall into other domains; however, this classification represents the majority.* | M: NR I: NR  N = NR | Pairwise meta-analysis did not show significant effects of exercise with social engagement on loneliness (Hedges' g = −0.24; 95%CI [−0.68 0.20]; Z = 1.75, p = 0.18; I2 : 0%, τ2 : <0.0001; p = 0.502) |
|  | **Exercise Interventions** *(Baez, M, 2017; Larsen RT, 2021; Li J, 2017; Jing L, 2018; McAuley E, 2000; Mutrie N, 2012; Pinheiro HA, 2020 from Yu DS, 2023)* | Quantitative (RCT, N-RCT) | The interventions included individual-based exercise interventions addressing both physical and mental risk factors (n=6) and group-based exercise interventions with active strategies to enhance social interaction (n=4). | M: NR I: NR  N = NR | Narrative analysis showed that three exercise interventions reported non-significant intervention effects on loneliness. Within-group improvement was reported in two studies. |
|  | **Exergames Program** *(Unbehaun D, 2018 from Heins P, 2021)* | Qualitative | The intervention examined regular use of exergame programs delivered by trained research assistants twice per week for 8 months. | M: NR  I: NR  N = 23 | Benefits for people with dementia included increased social interaction. |
|  | **Physical Activity Intervention** *(Reviews from Adekpedjou R, 2023)* | Quantitative (Umbrella review) | Participants in the physical activity intervention were community-dwelling older adults who had a mean age of 65+ years. | M: NR  I: NR  N = NR | Physical activity may improve social functioning. |
|  | **Physical Activity Interventions** *(Agmon M, 2011; Chao YY, 2018; Jung Y, 2009; Rendon AA, 2012; Rosenberg D, 2012; Wollersheim D, 2010; Xu X, 2016; Bartlett H, 2013; Hopman-Rock M, 2002; Kamegaya T, 2014; McAuley E, 2000; Seino S, 2017 from Paquet C, 2023)* | Mixed-and/or multi-method | This intervention category includes interventions with and educational and exercise program (combined) or just exercise component (singular). | M: NR  I: NR  N = NR | There was an increase in social network size and social support and a decrease in loneliness. This, however, was dependent on the intervention type. For example, exergames (those with educational and recreational components) were most effective. |
| **Spirituality interventions**  **(n=3)**  *Spirituality can be described as finding life’s meaning and purpose by connecting with the sacred (Marques A, 2022). Religious/spiritual interventions facilitate, educate, and operate from a religious/spiritual lens. These interventions do not include physical activity/movement as the dominant component.* | **Christian Faith-Based Intervention** *(Don'L B, 2023)* | Quantitative (Pre-post) | A Christian faith-based intervention that ran virtually over 12 weeks for 30 minutes weekly for participants ≥55 years. The intervention components included prayer, scripture readings, mindfulness, and gratitude practice. This intervention was conducted via Zoom by local ministers with guided materials, and individual meetings were additionally offered via social media. The aim was to increase feelings of social inclusion. | M: Virtual (Computer) I: Mixed (Group-based, one-to-one) N = 16 | There was a significant decrease in social loneliness, but no significant decrease in social quality of life. |
|  | **mHealth-Supported Volunteer-Assisted Spiritual Well-Being (mVS)** *(Lou VW, 2023)* | Quantitative (Quasi-experimental) | Older adults (aged ≥ 60) received the mVS (mHealth-Supported Volunteer-Assisted Spiritual Well-Being) intervention to impact spiritual well-being. The mVS intervention supported by the mobile application Fu Le Man Xin, adapts the SEGCE protocol into an eight-session individualized model, introducing concepts such as the meaning of life, spiritual breathing, self-care, family support, friendship, community contribution, and personal values. Trained volunteers guide participants through each session, fostering a holistic approach to spiritual well-being rooted in Chinese cultural principles and perspectives on spirituality. | M: In-person I: One-to-one N = 161 | Relationship with others (p = .014) significantly improved. Relationship with family did not improve. |
|  | **Religious Quran Intervention**  *(Borji M, 2020)* | Quantitative (Semi-experimental) | The religious intervention was based on Quran verses and narrations of infallible, with major foci of recourse, gratitude, patience, forgiveness and amnesty, participation in public affairs, Dhikr (reciting prayers), and pilgrimage. The religious interventions were performed through 20 sessions, each lasting for 30–45 min by nurses. | M: In-person I: Group-based N = 88 | The religious intervention significantly reduced loneliness in older adults and the effect was maintained at least 1-month after the intervention. |

*****M=mode of delivery; I = level of interaction with interventions; N=number of participants; NR = Not reported

**Supplementary Table 2d:** Study and intervention characteristics, including a summary of study results and outcomes for *General resource interventions* (n = 2)

| **Intervention Type** (number of studies)  *Definition* | **Intervention name**  *(Author, year)* | **Study design** | **Intervention and population description** | **Intervention details** | **Summary of study results and outcomes** |
| --- | --- | --- | --- | --- | --- |
| **General Resource Interventions (n=2)** | **Environmental Interventions** *(Schmidt T, 2021 from Tcymbal A, 2026)* | Mixed-and/or multi-method | One study, "Move the Neighborhood," utilized a participatory research approach involving collaboration between local older adults and landscape architects to enhance neighborhood open spaces. The environmental intervention demonstrated positive effects on social participation and physical activity through renovations based on collaborative ideas, as evaluated through quantitative measures and qualitative interviews with older participants. | M: In-person I: NR  N = 10 | A single mixed method environmental study showed that renovating the neighborhood open spaces had some positive effects on social participation. |
|  | **Hearing Aid and Hearing Diary**  *(Tesch-Römer C, 1997 from Cohen-Mansfield J, 2015)* | Quantitative (Quasi-experimental) | Older adults age ≥55 years with mild-to-moderate hearing loss who received a hearing aid for the first time and kept a ‘‘hearing diary’’ for about 2 months. | M: In-person I: Self-directed N = 140 | The intervention group showed a nonsignificant decrease in loneliness from baseline to 6-month follow-up. |

*****M=mode of delivery; I = level of interaction with interventions; N=number of participants; NR = Not reported.
