# Supplementary material for: Interventions that have potential to help older adults living with social frailty: a systematic scoping review
Source: BMC Geriatr. 2024 Jun 15;24:521. doi: 10.1186/s12877-024-05096-w (PMC11179268; doi:10.1186/s12877-024-05096-w)
Supplement: Supplementary file 3 — Supplementary Material 3. [file 12877_2024_5096_MOESM3_ESM.docx]

**Supplemental Table 3:** Categorization of outcomes according to Bunt *et al*’s social frailty model

| **Outcome** **category, sub-category, outcome** (number of interventions) | **Intervention category**  *(Number of interventions; *number of effective interventions defined as: reported as statistically significant positive impact on quantitative outcome)* | | | | |
| --- | --- | --- | --- | --- | --- |
|  | **Social resource-related interventions (n=196)** | **Self-management related interventions (n=157)** | | **Social behavioural activity related interventions (n=140)** | **General resource interventions (n=2)** |
| **Interventions investigating loneliness outcomes (n=312 interventions)** *The subjective experience of social isolation characterized as an imbalance/discrepancy between desired social contacts and actual social contact (Veazie S, 2019).* | | | | | |
| **Quantitative outcomes (n=275 interventions)** | | | | | |
| **Loneliness or perceived loneliness**  *109/263 interventions effective (41.4%)* | - Aging in place (n=17; *n=7) - Befriending (n=12; *n=1) - General social support (n=1;*n=1) - ICT-based (n=22; *n=7) - Intergenerational (n=13; *n=9) - Mentorship (n=6; *n=3) - Peer support (n=9; *n=3) - Socially assistive robots or conversational agents (n=10; *n=7) | - Psychological self-management (n=34; *n=17) - Self-management education (n=64; *n=20) - Social prescribing or asset-based (n=5; *n=4) | | - Arts-based (n=9; *n=5) - Leisure activity (n=22; *n=9) - Mind-body (n=10; *n=5) - Physical activity (n=27; *n=10) - Spiritual-based (n=1; *n=1) | - Hearing aid (n=1; *n=0) |
| **Social loneliness**  *8/13 interventions effective (61.5%)* | - ICT-based (n=1; *n=0) - Intergenerational (n=1; *n=0) - Peer support (n=3; *n=2) | - Psychological self-management (n=1; *n=1) - Self-management education (n=3; *n=2) | | - Mind-body (n=2; *n=1) - Physical activity (n=1; *n=1) - Spiritual-based (n=1; *n=1) | - |
| **Emotional loneliness**  *6/10 interventions effective (60%)* | - ICT-based (n=1; *n=0) - Intergenerational (n=1; *n=1) - Peer support (n=2; *n=1) | - Psychological self-management (n=1; *n=1) - Self-management education (n=3; *n=2) | | - Mind-body (n=1; *n=1) - Physical activity (n=1; *n=0) | - |
| **Feeling of loneliness or subjective feelings of loneliness**  *2/4 interventions effective (50%)* | - Aging in place (n=1; *n=1) - ICT-based (n=1; *n=0) | - Self-management education (n=1; *n=0) | | - Arts-based (n=1; *n=1) | - |
| **Overall loneliness**  *2/3 interventions effective (66.7%)* | - | - Psychological self-management (n=1; *n=1) - Self-management education (n=2; *n=1) | | - | - |
| **General loneliness** | - ICT-based (n=1; *n=0) | - | | - | - |
| **Maladaptive social cognitions for loneliness and emotional deprivation** | - | - Psychological self-management (n=1; *n=1) | | - | - |
| **Qualitative outcomes (perceptions) (n=44 interventions)** | | | | | |
| **Loneliness** *(n=39 interventions)* | - Aging in place (n=2) - Befriending (n=4) - ICT-based (n=5) - Intergenerational (n=2) - Peer support (n=3) - Socially assistive robots or conversational agents (n=7) | - Psychological self-management (n=4) - Self-management education (n=1) | | - Arts-based (n=3) - Leisure activity (n=4) - Physical activity (n=4) | - |
| **Feelings of loneliness** *(n=4 interventions)* | - Befriending (n=1) - Socially assistive robots or conversational agents (n=2) | - Self-management education (n=1) | | - | - |
| **Temporal loneliness** *(n=2 interventions)* | - ICT-based (n=1) | - Self-management education (n=1) | | - | - |
| **Interventions investigating social cohesion and connectedness outcomes (n=120)** *Social cohesion refers to the strength of relationships and the sense of solidarity among members of a community (Manca AR, 2015).* | | | | | |
| **Quantitative outcomes (n=33 interventions)** | | | | | |
| **Quality of relationships** | | | | | |
| **Quality of friendships**  *0/3 interventions effective (0%)* | - | - Self-management education (n=3; *n=0) | | - | - |
| **Relationships with family**  *0/2 interventions effective (0%)* | - | - Psychological self-management (n=1; *n=0) | | - Spiritual-based (n=1; *n=0) | - |
| **Positive relationships** | - | - | | - Leisure activity (n=1; *n=1) | - |
| **Quality of friend network** | - | - | | - Leisure activity (n=1; *n=0) | - |
| **Relationships with friends** | - | - Psychological self-management (n=1; *n=0) | | - | - |
| **Relationships with others** | - | - | | - Spiritual-based (n=1; *n=1) | - |
| **Social network quality** | - | - Self-management education (n=1; *n=1) | | - | - |
| **Social quality** | - | - | | - Arts-based (n=1; *n=1) | - |
| **Social connections** | | | | | |
| **Social connectedness or social connection**  *3/12 interventions effective (25%)* | - Aging in place (n=2; *n=0) - ICT-based (n=1; *n=0) - Intergenerational (n=1; *n=0) | - Self-management education (n=3; *n=0) | | - Leisure activity (n=3; *n=2) - Mind-body (n=1; *n=0) - Physical activity (n=1; n=1) | - |
| **Belonging or sense/feeling of belonging**  *1/3 interventions effective (33.3%)* | - Aging in place (n=1; *n=0) - ICT-based (n=1; *n=1) | - Psychological self-management (n=1; *n=0) | | - | - |
| **Community belonging**  *0/2 interventions effective (0%)* | - Aging in place (n=1; *n=0) | - | | - Arts-based (n=1; *n=0) | - |
| **Attachment** | - | - | | - Leisure activity (n=1; *n=0) | - |
| **Closeness to children** | - Intergenerational (n=1; *n=1) | - | | - | - |
| **Companionship** | - Befriending (n=1; *n=0) | - | | - | - |
| **Connectedness with community** | - | - | | - Physical activity (n=1; n=0) | - |
| **In touch with community** | - Intergenerational (n=1; *n=0) | - | | - | - |
| **Relatedness** | - | - Self-management education (n=1; *n=1) | | - | - |
| **Sense of community** | - | - | | - Arts-based (n=1; *n=0) | - |
| **Social integration** | - | - | | - Leisure activity (n=1; *n=0) | - |
| **Togetherness** | - | - | | - Leisure activity (n=1; *n=0) | - |
| **Total connection** | - | - | | - Mind-body (n=1; *n=1) | - |
| **Qualitative outcomes (perceptions) (n=89 interventions)** | | | | | |
| **Quality of relationships** | | | | | |
| **Deepened or enhanced relationships**  *(n=6 interventions)* | - Aging in place (n=1) - ICT-based (n=3) | - Self-management education (n=1) | | - Arts-based (n=1) | - |
| **Meaningful relationships or relationship meaningfulness**  ***(****n=3 interventions)* | - Befriending (n=1) - ICT-based (n=1) - Intergenerational (n=1) | - | | - | - |
| **Relationship value**  *(n=2 interventions* | - Befriending (n=2) | - | | - | - |
| **Enhanced friendships** | - | - | | - Leisure activity (n=1) | - |
| **Meaningful intergenerational relationships** | - Intergenerational (n=1) | - | | - | - |
| **Meaningful peer relationships** | - Aging in place (n=1) | - | | - | - |
| **Peer relationships** | - | - Self-management education (n=1) | | - | - |
| **Positive social contact** | - | - Self-management education (n=1) | | - | - |
| **Relationship authenticity** | - Befriending (n=1) | - | | - | - |
| **Relationships** | - | - Psychological self-management (n=1) | | - | - |
| **Relationships to self and others** | - | - | | - Arts-based (n=1) | - |
| **Social life** | - Mentorship (n=1) | - | | - | - |
| **Social relationships** | - | - | | - Physical activity (n=1) | - |
| **Social connections** | | | | | |
| **Social connectedness or social connection**  *(n=36 interventions)* | - Aging in place (n=2) - ICT-based (n=5) - Intergenerational (n=4) - Peer support (n=1) - Socially assistive robots or convsersational agents (n=1) | - Self-management education (n=11) | | - Arts-based (n=5) - Leisure activity (n=3) - Physical activity (n=4) | - |
| **Belonging or sense/feeling of belonging**  *(n=13 interventions)* | - Befriending (n=2) - Peer support (n=3) | - Self-management education (n=2) | | - Arts-based (n=3) - Leisure activity (n=1) - Physical activity (n=2) | - |
| **Companionship**  *(n=12 interventions)* | - Aging in place (n=1) - Intergenerational (n=1) - Peer support (n=1) - Socially assistive robots or conversational agents (n=9) | - | | - | - |
| **Social cohesion**  *(n=3 interventions)* | - | - | | - Arts-based (n=3) | - |
| **Connection with others**  *(n=2 interventions)* | - | - | | - Arts-based (n=1) - Physical activity (n=1) | - |
| **Feeling of closeness with others**  *(n=2 interventions)* | - ICT-based (n=2) | - | | - | - |
| **Feeling part of a community**  *(n=2 interventions)* | - Befriending (n=2) | - | | - | - |
| **Feelings of connection with the world**  *(n=2 interventions)* | - Befriending (n=1) | - Self-management education (n=1) | | - | - |
| **Sense of community**  *(n=2 interventions)* | - Intergenerational (n=1) | - | | - Arts-based (n=1) |  |
| **Social connectivity**  *(n=2 interventions)* | - ICT-based (n=2) | - | | - | - |
| **Bonding** | - | - | | - Physical activity (n=1) | - |
| **Connectedness with the community** | - | - Psychological self-management (n=1) | | - | - |
| **Feelings of reciprocity** | - | - | | - Leisure activity (n=1) | - |
| **Fellowship** | - Peer support (n=1) | - | | - | - |
| **Group cohesion** | - | - | | - Physical activity (n=1) | - |
| **Intergeneration closeness** | - Intergenerational (n=1) | - | | - | - |
| **Intergenerational connection** | - Intergenerational (n=1) | - | | - | - |
| **Interpersonal connection** | - Intergenerational (n=1) | - | | - | - |
| **Interventions investigating the quantity of social relationships (n=98)** *Outcomes related to the size, quantity, frequency or an expansion/addition of new relationships, interactions or activities.* | | | | | |
| **Quantitative outcomes (n=47 interventions)** | | | | | |
| **Number, amount or frequency of connections, contacts or interactions** | | | | | |
| **Social network size or social networks**  *0/8 interventions effective (0%)* | - Befriending (n=1; *n=0) - Peer support (n=1; *n=0) - ICT-based (n=1; *n=0) | - Psychological self-management (n=1; *n=0) - Self-management education (n=2; *n=0) | | - Leisure activity (n=1; *n=0) - Physical activity (n=1; *n=0) | - |
| **Amount of interactions or number of social interactions**  *2/5 interventions effective (40%)* | - | - Psychological self-management (n=3; *n=2) - Self-management education (n=2; *n=0) | | - | - |
| **Amount of social contact**  *3/4 interventions effective (75%)* | - Peer support (n=1; *n=1) | - Psychological self-management (n=1; *n=1) - Self-management education (n=1; *n=1) | | - Arts-based (n=1; *n=0) | - |
| **Number of friends or friendships**  *1/3 interventions effective (33.3%)* | - | - Self-management education (n=3; *n=1) | | - | - |
| **Social network scores**  *1/3 interventions effective (33.3%)* | - Aging in place (n=1; *n=0) | - | | - Leisure activity (n=1; *n=1) - Arts-based (n=1; *n=0) | - |
| **Frequency of talking with friends**  *0/2 interventions effective (0%)* | - Aging in place (n=1; *n=0) - ICT-based (n=1; *n=0) | - | | - - | - |
| **Amount of social connections**  *0/2 interventions effective (0%)* | - ICT-based (n=1; *n=0) | - | | - Physical activity (n=1; *n=0) | - |
| **Amount of social contacts**  *2/2 interventions effective (100%)* | - Befriending (n=1; *n=1) | - | | - Leisure activity (n=1; *n=1) | - |
| **Amount of contact with family and friends** | - Aging in place (n=1; *n=0) | - | | - | - |
| **Amount of telephone contact** | - | - Psychological self-management (n=1; *n=0) | | - | - |
| **Contact with children outside of neighborhoods through volunteering** | - | - | | - Leisure activity (n=1; *n=1) | - |
| **Frequency of interacting with friends** | - | - | | - Leisure activity (n=1; *n=0) | - |
| **Frequency of social interactions** | - | - Self-management education (n=1; *n=0) | | - | - |
| **Frequency of talking time with families living together** | - ICT-based (n=1; *n=1) | - | | - | - |
| **Frequency of talking time with families not living together** | - ICT-based (n=1; *n=1) | - | | - | - |
| **Interpersonal contact** | - | - Psychological self-management (n=1; *n=1) | | - | - |
| **Number of adults seen in a week** | - | - | | - Leisure activity (n=1; *n=0) | - |
| **Number of distant friends** | - | - | | - Leisure activity (n=1; *n=1) | - |
| **Size of friend network** | - | - | | - Leisure activity (n=1; *n=0) | - |
| **Number of new contacts or relationships** | | | | | |
| **New friendships**  *2/7 interventions effective (28.6%)* | - Mentorship (n=1; *n=0) - Peer support (n=1; *n=1) | - Psychological self-management (n=1; *n=0) - Self-management education (n=2; *n=0) | | - Leisure activity (n=1; *n=1) - Physical activity (n=1; n=0) | - |
| **New relationships**  *0/2 interventions effective (0%)* | - | - Self-management education (n=1; *n=0) | | - Leisure activity (n=1; *n=0) | - |
| **New social interactions**  *0/2 interventions effective (0%)* | - ICT-based (n=1; *n=0) | - Self-management education (n=1; *n=0) | | - | - |
| **New social ties built**  *0/2 interventions effective (0%)* | - Mentorship (n=1; *n=0) | - | | - Leisure activity (n=1; *n=0) | - |
| **New social connections** | - | - | | - Leisure activity (n=1; *n=0) | - |
| **Number, amount or frequency of social activities engagement** | | | | | |
| **Amount of social activity or social activity**  *3/6 interventions effective (50%)* | - | - Self-management education (n=1; *n=0) | | - Arts-based (n=2; *n=1) - Leisure activity (n=3; *n=2) | - |
| **Amount of activities** | - | - Self-management education (n=1; *n=0) | | - | - |
| **Amount of activity engagement** | - | - | | - Leisure activity (n=1; *n=0) | - |
| **Amount of online engagement** | - | - Self-management education (n=1; *n=0) | | - | - |
| **Getting together socially** | - Aging in place (n=1; *n=0) | - | | - | - |
| **Initiating conversation with young participants** | - Intergenerational (n=1; *n=0) | - | | - | - |
| **Involvement in outside activities** | - | - Psychological self-management (n=1; *n=0) | | - | - |
| **Meaningful activity frequency** | - | - Self-management education (n=1; *n=1) | | - | - |
| **Number of activities involving social connections** | - | - Self-management education (n=1; *n=0) | | - | - |
| **Qualitative outcomes (perceptions) (n=52 interventions)** | | | | | |
| **Number, amount or frequency of connections, contacts or interactions** | | | | | |
| **Social network size or social networks**  *(n=8 interventions)* | - Aging in place (n=1) - ICT-based (n=1) - Intergenerational (n=2) | - | | - Arts-based (n=1) - Leisure activity (n=1) - Physical activity (n=2) | - |
| **Amount of contact with others or social contact**  *(n=4 interventions)* | - ICT-based (n=2) | - Self-management education (n=1) | | - Arts-based (n=1) | - |
| **Amount or expansion of social interactions**  *(n=2 interventions)* | - ICT-based (n=1) - Socially assistive robots or conversational agents (n=1) | - | | - | - |
| **Number of community connections**  *(n=2 interventions)* | - Befriending (n=1) | - | | - Arts-based (n=1) | - |
| **Amount of cross generation connections** | - Intergenerational (n=1) | - | | - | - |
| **Amount of interaction with friends and family** | - | - Self-management education (n=1) | | - | - |
| **Amount of ordinary conversation** | - Befriending (n=1) | - | | - | - |
| **Contact with grandchildren** | - | - Self-management education (n=1) | | - | - |
| **Frequency of social interaction** | - ICT-based (n=1) | - | | - | - |
| **Number of social relationships** | - Peer support (n=1) | - | | - | - |
| **Social contacts** | - | - | | - Leisure activity (n=1) | - |
| **Number of new contacts or relationships** | | | | | |
| **New connections or social connections**  *(n=8 interventions)* | - Peer support (n=1) - Socially assistive robots or conversational agents (n=2) | - Self-management education (n=2) - Social prescribing or asset-based (n=1) | | - Arts-based (n=1) - Physical activity (n=1) | - |
| **New friendships**  *(n=6 interventions)* | - Befriending (n=1) - ICT-based (n=1) - Mentorship (n=1) | - Self-management education (n=1) | | - Arts-based (n=1) - Physical activity (n=1) | - |
| **New relationships built**  *(n=3 interventions)* | - Intergenerational (n=2) | - | | - Arts-based (n=1) | - |
| **Developing social relationships**  *(n=2 interventions)* | - ICT-based (n=2) | - | | - | - |
| **Meeting new friends**  *(n=2 interventions)* | - Intergenerational (n=1) | - | | - Leisure activity (n=1) | - |
| **Meeting other generations**  *(n=2 interventions)* | - Intergenerational (n=2) | - | | - | - |
| **Creating new or reviving social relationships** | - | - | | - Leisure activity (n=1) | - |
| **Creation of strong relationships** | - | - | | - Leisure activity (n=1) | - |
| **Cultivation of intergenerational bonds** | - | - | | - Arts-based (n=1) | - |
| **Developing meaningful connections** | - | - | | - Arts-based (n=1) | - |
| **Forming intimate friendships** | - Befriending (n=1) | - | | - | - |
| **Forming light-hearted friendships** | - Befriending (n=1) | - | | - | - |
| **Foster meaningful relationships** | - | - | | - Arts-based (n=1) | - |
| **Meeting new people** | - | - | | - Leisure activity (n=1) | - |
| **New friendship networks** | - | - | | - Leisure activity (n=1) | - |
| **New real friendships** | - | - Self-management education (n=1) | | - | - |
| **New supportive friends with similar experiences** | - | - Self-management education (n=1) | | - | - |
| **Number, amount or frequency of social activities engagement** | | | | | |
| **Amount of social activity**  (n=3 interventions) | - Befriending (n=1) - Mentorship (n=1) | - Self-management education (n=1) | | - | - |
| **New experiences** | - ICT-based (n=1) | - | | - | - |
| **Social networking** | - | - | | - Arts-based (n=1) | - |
| **Interventions investigating social capital outcomes (n=95)**  *Defined as the resources available to individuals and groups through social connections and social relations with others (Cannuscio C, 2003), and can include social support, access to knowledge, and satisfaction with social support.* | | | | | |
| **Quantitative outcomes (n=76 interventions)** | | | | | |
| **Social support** | | | | | |
| **Social support or perceived social support**  *19/50 interventions effective (38%)* | - Aging in place (n=1; *n=1) - Befriending (n=2; *n=1) - ICT-based (n=9; *n=3) - Intergeneration (n=2; *n=0) - Mentorship (n=3; *n=1) - Peer support (n=1; *n=1) - Socially assistive robots or conversational agents (n=1; *n=0) | - Psychological self-management (n=3; *n=0) - Self-management education (n=20; *n=8) - Social prescribing or asset-based (n=2; *n=1) | - Leisure activity (n=4; *n=2) - Mind-body (n=1; *n=1) - Physical activity (n=1; *n=0) | | - |
| **Social support received**  *1/3 interventions effective (33.3%)* | - Aging in place (n=1; *n=0) | - Self-management education (n=1; *n=0) | - Leisure activity (n=1; *n=1) | | - |
| **Structured or structural social support**  *3/3 interventions effective (100%)* | - Peer support (n=1; *n=1) | - Self-management education (n=1; *n=1) | - Leisure activity (n=1; *n=1) | | - |
| **Functional social support**  *1/2 interventions effective (50%)* | - | - | - Leisure activity (n=1; *n=1) | | - |
| **Desired support** | - | - Self-management education (n=1; *n=1) | - | | - |
| **Emotional support** | - | - | - Leisure activity (n=1; *n=0) | | - |
| **Social satisfaction** | | | | | |
| **Satisfaction social support**  *3/4 interventions effective (75%)* | - Mentorship (n=1; *n=0) | - Psychological self-management (n=2; *n=2) - Social prescribing or asset-based (n=1; *n=1) | | - | - |
| **Satisfaction with social activities**  *0/2 interventions effective (0%)* | - | - Self-management education (n=1; *n=0) | | - Leisure activity (n=1; *n=0) | - |
| **Relationship satisfaction** | - | - Self-management education (n=1; *n=1) | | - | - |
| **Satisfaction for the relationship with families living together** | - ICT-based (n=1; *n=1) | - | | - | - |
| **Satisfaction social participation** | - | - | | - Leisure activity (n=1; *n=1) | - |
| **Satisfaction with social connection** | - Aging in place (n=1; *n=0) | - | | - | - |
| **Satisfaction with socialization** | - | - Psychological self-management (n=1; *n=0) | | - | - |
| **Social satisfaction** | - Aging in place (n=1; *n=0) | - | | - | - |
| **Support satisfaction** | - Peer support (n=1; *n=1) | - | | - | - |
| **Other** | | | | | |
| **Social capital**  *0/2 interventions effective (0%)* | - Aging in place (n=1; *n=0) - Intergenerational (n=1; *n=0) | - | | - | - |
| **Access to social support** | - | - Self-management education (n=1; *n=0) | | - | - |
| **Familiarity with services in the community** | - | - Self-management education (n=1; *n=2) | | - | - |
| **Number of adults one could depend on** | - | - | | - Leisure activity (n=1; *n=0) | - |
| **Number of people one could turn to for help** | - | - | | - Leisure activity (n=1; *n=0) | - |
| **Number of people participants felt they could reach out to** | - | - | | - Leisure activity (n=1; *n=1) | - |
| **Relationship status** | - Socially assistive robots or conversational agents (n=1;*n=0) | - | | - | - |
| **Social support provided** | - | - Self-management education (n=1; *n=1) | | - | - |
| **Unmet social needs** | - Aging in place (n=1; *n=1) | - | | - | - |
| **Qualitative outcomes (perceptions) (n=20 interventions)** | | | | | |
| **Social support** | | | | | |
| **Social support**  *(n=7 interventions)* | - ICT-based (n=1) - Socially assistive robots or conversational agents (n=2) | - Self-management education (n=1) | | - Physical activity (n=2) | - |
| **Emotional support** | - Befriending (n=1) | - | | - | - |
| **Practical support** | - Aging in place (n=1) | - | | - | - |
| **Social support networks** | - Peer support (n=1) | - | | - | - |
| **Social support system** | - | - | | - Arts-based (n=1) | - |
| **Social satisfaction** | | | | | |
| **Enjoyment of experiences** | - Intergenerational (n=1) | - | | - | - |
| **Enjoyment of social activities** | - | - Self-management education (n=1) | | - | - |
| **Other** | | | | | |
| **Access to information** | - | - Self-management education (n=1) | | - | - |
| **Desire for more friendships** | - | - | | - Physical activity (n=1) | - |
| **Knowledge of how to access support and services** | - Befriending (n=1) | - | | - | - |
| **Knowledge of other activities in the neighbourhood** | - | - Self-management education (n=1) | | - | - |
| **Risk of social isolation** | - | - | | - Arts-based (n=1) | - |
| **Social capital** | - | - | | - Physical activity (n=1) | - |
| **Social needs** | - | - | | - Arts-based (n=1) | - |
| **Social needs met** | - | - Self-management education (n=1) | | - | - |
| **Interventions investigating health and wellbeing outcomes (n=83)**  *An individual’s subjective sense of optimal functioning in position in life or health (Gasper D, 2010).* | | | | | |
| **Quantitative outcomes (n=54 interventions)** | | | | | |
| **General health and wellbeing outcomes** | | | | | |
| **Well-being**  *8/15 interventions effective (53.3%)* | - Aging in place (n=2; *n=2) - ICT-based (n=1; *n=0) - Socially assistive robots or conversational agents (n=1; *n=0) | - Self-management education (n=6; *n=2) | | - Arts-based (n=1; *n=1) - Mind-body (n=2; *n=2) - Physical activity (n=2; *n=1) | - |
| **Subjective well-being**  *3/5 interventions effective (60%)* | - | - Self-management education (n=2; *n=2) | | - Arts-based (n=1; *n=1) - Mind-body (n=1; *n=0) - Physical activity (n=1; *n=0) | - |
| **Self-perceived self-reported, or self-rated health**  *3/4 interventions effective (75%)* | - Mentorship (n=1; *n=1) | - Self-management education (n=2; *n=1) - Physical activity (n=1; *n=1) | | - | - |
| **Activities of daily living**  *0/3 interventions effective (0%)* | - Mentorship (n=1; *n=0) | - | | - Arts-based (n=1; *n=0) - Physical activity (n=1; *n=0) | - |
| **Overall health**  *2/3 interventions effective (66.7%)* | - | - | | - Arts-based (n=2; *n=2) - Mind-body (n=1; *n=0) | - |
| **Health**  *1/2 interventions effective (50%)* | - | - | | - Leisure activity (n=1; *n=1) - Physical activity (n=1; *n=0) | - |
| **Health status or self-reported health status**  *0/2 interventions effective (0%)* | - Aging in place (n=1; *n=0) - Intergenerational (n=1; *n=0) | - | | - | - |
| **Social or sense of social well-being**  0/2 interventions effective (0%) | - | - Self-management education (n=1; *n=0) | | - Physical activity (n=1; *n=0) | - |
| **Instrumental activities of daily living**  *0/2 interventions effective (0%)* | - Mentorship (n=1; *n=0) | - | | - Leisure activity (n=1; *n=0) | - |
| **Aging in place** | - Aging in place (n=1; *n=0) | - | | - | - |
| **Everyday functioning** | - | - Self-management education (n=1; *n=0) | | - | - |
| **Functioning autonomy** | - | - | | - Leisure activity (n=1; *n=1) | - |
| **General health** | - | - | | - Arts-based (n=1; *n=0) | - |
| **Global functioning** | - Aging in place (n=1; *n=1) | - | | - | - |
| **Health and psychosocial functioning** | - Aging in place (n=1; *n=1) | - | | - | - |
| **Independence of life** | - | - | | - Leisure activity (n=1; *n=1) | - |
| **Independent living** | - | - Self-management education (n=1; *n=1) | | - | - |
| **Independent living performance** | - Mentorship (n=1; *n=0) | - | | - | - |
| **Knowledge and general skills outcomes** | | | | | |
| **Resilience**  *5/8 interventions effective (62.5%)* | - Mentorship (n=2; *n=2) - Socially assistive robots or conversational agents (n=1; *n=1) | - Psychological self-management (n=3; *n=1) - Self-management education (n=1; *n=0) | | - Mind-body (n=1; *n=1) | - |
| **Self-efficacy**  *2/5 interventions effective (40%)* | - Mentorship (n=1; *n=0) | - Self-management education (n=3; *n=2) | | - Physical activity (n=1; *n=0) | - |
| **Levels of goal attainment** | - | - Psychological self-management (n=1; *n=0) | | - | - |
| **Perceived control** | - | - Self-management education (n=1; *n=0) | | - | - |
| **Self-confidence** | - Intergenerational (n=1; *n=1) | - | | - | - |
| **Qualitative outcomes (perceptions) (n=32 interventions)** | | | | | |
| **General health and wellbeing outcomes** | | | | | |
| **Well-being or perceived well-being**  *(n=12 interventions)* | - Aging in place (n=1) - ICT-based (n=1) - Intergenerational (n=1) - Peer support (n=2) - Socially assistive robots or conversational agents (n=1) | - Self-management education (n=3) | | - Arts-based (n=1) - Leisure activity (n=1) - Physical activity (n=1) | - |
| **Health or subjective health**  *(n=6 interventions)* | - Aging in place (n=1) - Intergenerational (n=1) - Socially assistive robots or conversational agents (n=1) | - Self-management education (n=1) | | - Physical activity (n=2) | - |
| **Independence**  *(n=4 interventions)* | - Befriending (n=1) - Mentorship (n=1) - Socially assistive robots or conversational agents (n=1) | - Self-management education (n=1) | | - | - |
| **Social well-being**  *(n=2 interventions)* | - Socially assistive robots or conversational agents (n=1) | - | | - Physical activity (n=1) | - |
| **Capacity for activities of daily living** | - | - | | - Physical activity (n=1) | - |
| **Eudemonic well-being** | - | - Psychological self-management (n=1) | | - | - |
| **General health** | - Befriending (n=1) | - | | - | - |
| **Overall well-being** | - | - | | - Arts-based (n=1) | - |
| **Psychosocial well-being** | - | - | | - Physical activity (n=1) | - |
| **Social health** | - | - | | - Physical activity (n=1) | - |
| **Knowledge and general skills outcomes** | | | | | |
| **Confidence or self-confidence**  *(n=8 interventions)* | - Befriending (n=4) - Mentorship (n=1) | - Social prescribing or asset-based (n=2) | | - Arts-based (n=1) | - |
| **Coping**  *(n=2 interventions)* | - Befriending (n=1) - Peer support (n=1) | - | | - | - |
| **Feelings of control** | - | - Social prescribing or asset-based (n=1) | | - | - |
| **Problem solving abilities** | - | - | | - Arts-based (n=1) | - |
| **Resilience** | - | - | | - Physical activity (n=1) | - |
| **Self-efficacy** | - | - | | - Physical activity (n=1) | - |
| **Interventions investigating social engagement outcomes (n=78)**  *Social engagement is as a multidimensional relational concept featuring psychological and behavioral attributes of connection, interaction, participation, and involvement, designed to achieve or elicit an outcome at individual or social levels (Johnston KA, 2018). Includes outcomes related to the engagement of activities and opportunities, including interacting with others. Specifically, these outcomes are about the process of engagement, not the quality of quantity of it.* | | | | | |
| **Quantitative outcomes (n=41 interventions)** | | | | | |
| **Social participation or subjective social participation**  *3/12 interventions effective (25%)* | - Mentorship (n=1; *n=0) | - Psychological self-management (n=3; *n=1) - Self-management education (n=2; *n=0) - Social prescribing or asset-based (n=2; *n=2) | | - Leisure activity (n=1; *n=0) - Physical activity (n=3; *n=0) | - |
| **Social integration**  *2/6 interventions effective (33.3%)* | - ICT-based (n=1; *n=0) - Peer support (n=2; *n=0) | - Self-management education (n=1; *n=0) | | - Leisure activity (n=1; *n=1) - Physical activity (n=1; n=1) | - |
| **Social interactions**  *3/6 interventions effective (50%)* | - ICT-based (n=2; *n=0) | - Psychological self-management (n=1; *n=1) - Self-management education (n=1; *n=0) - Social prescribing or asset-based (n=1; *n=1) | | - Arts-based (n=1; *n=1) | - |
| **Social engagement**  *3/4 interventions effective (75%)* | - Aging in place (n=1; *n=1) | - Psychological self-management (n=1; *n=1) - Self-management education (n=1; *n=1) | | - Arts-based (n=1; *n=0) | - |
| **Social contact**  *1/3 interventions effective (33.3%)* | - Socially assistive robots or conversational agents (n=1; *n=0) | - Self-management education (n=1; *n=1) | | - Leisure activity (n=1; *n=0) | - |
| **Communal involvement** | - Intergenerational (n=1; *n=1) | - | | - | - |
| **Interpersonal communication** | - | - Self-management education (n=1; *n=1) | | - | - |
| **Opportunities to meet friends** | - Peer support (n=1; *n=0) | - | | - | - |
| **Opportunities to try new things** | - Peer support (n=1; *n=0) | - | | - | - |
| **Participating in activities** | - | - Self-management education (n=1; *n=1) | | - | - |
| **Social life** | - Intergenerational (n=1; *n=1) | - | | - | - |
| **Socializing** | - Peer support (n=1; *n=0) | - | | - | - |
| **Socializing with friends and family** | - | - Self-management education (n=1; *n=1) | | - | - |
| **Qualitative outcomes (perceptions) (n=41 interventions)** | | | | | |
| **Social interaction**  *(n=8 interventions)* | - ICT-based (n=2) - Intergenerational (n=1) - Socially assistive robots or conversational agents (n=2) | - Self-management education (n=1) | | - Physical activity (n=2) | - |
| **Social engagement or engagement**  *(n=6 interventions)* | - Befriending (n=1) | - Self-management education (n=2) | | - Leisure activity (n=1) - Physical activity (n=2) | - |
| **Social participation**  *(n=6 interventions)* | - Befriending (n=1) - ICT-based (n=1) | - Self-management education (n=2) | | - Physical activity (n=1) | - Environmental (n=1) |
| **Communication**  *(n=2 interventions)* | - ICT-based (n=1) | - | | - Physical activity (n=1) | - |
| **Opportunities to connect with others**  *(n=2 interventions)* | - | - Self-management education (n=1) | | - Arts-based (n=1) | - |
| **Socialization**  *(n=3 interventions)* | - | - Self-management education (n=1) | | - Leisure activity (n=2) | - |
| **Activities** | - Aging in place (n=1) | - | | - | - |
| **Anticipation of social events** | - | - Self-management education (n=1) | | - | - |
| **Engage in conversation (n=1)** | - | - | | - Leisure activity (n=1) | - |
| **Engagement in social activities** | - Peer support (n=1) | - | | - | - |
| **Engagement with others** | - | - Self-management education (n=1) | | - | - |
| **Everyday activity facilitation** | - | - Self-management education (n=1) | | - | - |
| **Intense social experiences** | - | - | | - Arts-based (n=1) | - |
| **Intergenerational engagement** | - | - Self-management education (n=1) | | - | - |
| **New ways of linking with people** | - | - Self-management education (n=1) | | - | - |
| **Novel social experiences** | - | - | | - Arts-based (n=1) | - |
| **Opportunities for forming new friendships** | - | - | | - Arts-based (n=1) | - |
| **Opportunities for social engagement** | - | - | | - Leisure activity (n=1) | - |
| **Opportunities for social interaction** | - | - | | - Arts-based (n=1) | - |
| **Opportunities to meet people** | - | - | | - Leisure activity (n=1) | - |
| **Organization of activities** | - | - Self-management education (n=1) | | - | - |
| **Participating in community** | - Peer support (n=1) | - | | - | - |
| **Participation in social activities** | - | - Self-management education (n=1) | | - | - |
| **Possibility for meeting people** | - Peer support (n=1) | - | | - | - |
| **Productive engagement** | - Aging in place (n=1) | - | | - | - |
| **Re-engage with the community** | - Befriending (n=1) | - | | - | - |
| **Re-engagement with community** | - Befriending (n=1) | - | | - | - |
| **Social activation** | - Peer support (n=1) | - | | - | - |
| **Social life** | - Mentorship (n=1) | - | |  |  |
| **Subjective social participation** | - | - Psychological self-management (n=1) | | - | - |
| **Interventions investigating social isolation outcomes (n=75 interventions)** *Defined as an objective lack of meaningful social connections (Veazie S, 2019). Often measured by the Lubben social network scale; self-reported measures of social engagement.* | | | | | |
| **Quantitative outcomes (n=54 interventions)** | | | | | |
| **Social isolation or perceived isolation**  *20/53 interventions effective (37.7%)* | - Aging in place (n=5; *n=1) - Befriending (n=5; *n=1) - ICT-based (n=4; *n=3) - Intergenerational (n=3; *n=2) - Mentorship (n=3; *n=1) - Peer support (n=2; *n=0) | - Psychological self-management (n=2; *n=0) - Self-management education (n=10; *n=4) - Social prescribing or asset-based (n=2; *n=1) | | - Arts-based (n=4; *n=1) - Leisure activity (n=6; *n=3) - Mind-body (n=1; *n=1) - Physical activity (n=6; *n=2) | - |
| **Social exclusion**  *0/2 interventions effective (0%)* | - | - | | - Physical activity (n=1; *n=0) - Arts-based (n=1; *n=0) | - |
| **Qualitative outcomes (perceptions) (n=22 interventions)** | | | | | |
| **Social isolation**  *(n=22 interventions)* | - Aging in place (n=2) - Befriending (n=2) - Intergenerational (n=1) - Peer support (n=2) - Socially assistive robots or conversational agents (n=2) | - Psychological self-management (n=1) - Self-management education (n=5) - Social prescribing or asset-based (n=1) | | - Arts-based (n=2) - Leisure activity (n=3) - Physical activity (n=1) | - |
| **Interventions investigating social functioning and skills outcomes (n=37 interventions)**  *Social functioning defines an individual’s ability to interact with their environment, family/significant others, fulfil their roles within environments such as work, and pursue leisure/recreational activates through using social skills. It is not about relationship quality or quantity, but an individual’s ability to manage or improve relationship quality (Bosc M, 2000)* | | | | | |
| **Quantitative outcomes (n=18 interventions)** | | | | | |
| **Social functioning**  *2/7 interventions effective (28.6%)* | - Intergenerational (n=3; *n=1) | - | | - Arts-based (n=1; *n=0) - Leisure activity (n=1; *n=0) - Physical activity (n=2; *n=1) | - |
| **Activity significance** | - | - Self-management education (n=1; *n=1) | | - | - |
| **Barriers to social participation** | - Mentorship (n=1; *n=1) | - | | - | - |
| **Confidence in social interactions** | - | - | | - Leisure activity (n=1; *n=0) | - |
| **Difficulties in social environment** | - | - | | - Leisure activity (n=1; *n=1) | - |
| **Getting along with others** | - Mentorship (n=1; *n=0) | - | | - | - |
| **Maintaining friendships** | - | - Self-management education (n=1; *n=0) | | - | - |
| **Making friends** | - | - Self-management education (n=1; *n=1) | | - | - |
| **Providing support to friends** | - | - | | - Leisure activity (n=1; *n=1) | - |
| **Self-management ability** | - | - Self-management education (n=1; *n=1) | | - | - |
| **Sociability** | - | - | | - Physical activity (n=1; *n=1) | - |
| **Social anxiousness** | - | - | | - Physical activity (n=1; *n=0) | - |
| **Social interaction skills** | - Aging in place (n=1; *n=0) | - | | - | - |
| **Qualitative outcomes (perceptions) (n=19 interventions)** | | | | | |
| **Maintaining social connections**  *(n=3 interventions)* | - Aging in place (n=1) | - Psychological self-management (n=1) | | - Leisure activity (n=1) | - |
| **Becoming socially active** | - Befriending (n=1) | - | | - | - |
| **Communication** | - ICT-based (n=1) | - | |  | - |
| **Confidence in social situations** | - | - | | - Leisure activity (n=1) | - |
| **Confidence to build social relationships** | - | - | | - Leisure activity (n=1) | - |
| **Confidence to initiate and maintain social contacts** | - | - Psychological self-management (n=1) | | - | - |
| **Ease in expressing emotions** | - Aging in place (n=1) | - | | - | - |
| **Ease of conversation** | - Intergenerational (n=1) | - | | - | - |
| **Interpersonal skills** | - | - | | - Mind-body (n=1) | - |
| **Maintaining friendships** | - | - | | - Arts-based (n=1) | - |
| **Maintaining social relationships** | - ICT-based | - | | - | - |
| **Maintenance of social interactions** | - ICT-based (n=1) | - | | - | - |
| **Motivation to make social connections** | - | - | | - Leisure activity (n=1) | - |
| **Motivation to seek out new activities** | - | - | | - Leisure activity (n=1) | - |
| **Perceived social self-efficacy** | - | - Psychological self-management (n=1) | | - | - |
| **Self-perceived barriers to socializing** | - Befriending (n=1) | - | | - | - |
| **Skills and knowledge in communicating** | - | - Self-management education (n=1) | | - | - |
| **Skills in developing social relationships** | - Peer support (n=1) | - | | - | - |
| **Social functioning** | - Befriending (n=1) | - | | - | - |
| **Social interaction behaviours** | - | - | | - Arts-based (n=1) |  |
| **Social skills** | - | - Psychological self-management (n=1) | | - | - |
| **Quality of life outcomes (n=54 interventions)**  *An individual’s appraisal of their position in life and health in the context of the culture and value systems in which they live and in relation to their goals, expectations, standards and concerns (WHOQOL)* | | | | | |
| **Quantitative outcomes (n=43 interventions)** | | | | | |
| **Quality of life or self-rated quality of life**  *10/29 interventions effective (34.5%)* | - Aging in place (n=3; *n=2) - Befriending (n=1; *n=0) - ICT-based (n=4; (n=1) - Intergenerational (n=1; *n=1) - Mentorship (n=1; *n=0) - Peer support (n=1; *n=0) - Socially assistive robots or conversational agents (n=1; *n=0) | - Psychological self-management (n=1; *n=1) - Self-management education (n=10; *n=3) | | - Arts-based (n=3; *n=1) - Leisure activity (n=1; *n=0) - Physical activity (n=2; *n=1) | - |
| **Life satisfaction or satisfaction with life**  *5/9 interventions effective (55.6%)* | - Aging in place (n=1; *n=0) - Mentorship (n=1; *n=0) | - Psychological self-management (n=1; *n=1) - Self-management education (n=2; *n=2) | | - Mind-body (n=3; *n=2) - Physical activity (n=1; *n=0) | - |
| **Health related quality of life**  *3/5 interventions effective (60%)* | - ICT-based (n=1; *n=0) | - Self-management education (n=2; *n=2) | | - Physical activity (n=2; *n=1) | - |
| **Social quality of life** | - | - | | - Spiritual-based (n=1; *n=0) | - |
| **Social-emotional quality of life** | - | - Psychological self-management (n=1; *n=1) | | - | - |
| **Qualitative outcomes (perceptions) (n=11 interventions)** | | | | | |
| **Quality of life**  *(n=7 interventions)* | - Aging in place (n=1) - Intergenerational (n=1) | - Self-management education (n=1) | | - Arts-based (n=1) - Leisure activity (n=3) | - |
| **Life satisfaction**  *(n=4 interventions)* | - ICT-based (n=1) | - Psychological self-management (n=1) - Self-management education (n=2) | | - | - |
| **Frailty outcomes (n=5 interventions)**  *Includes, physical, psychological, or social frailty outcomes* | | | | | |
| **Quantitative outcomes (n=5 interventions)** | | | | | |
| **Social frailty**  *0/2 interventions effective (0%)* | - Aging in place (n=1; *n=0) - Socially assistive robots or conversational agents (n=1; *n=0) | - | | - | - |
| **Frailty status** | - | - | | - Physical activity (n=1; *n=1) | - |
| **Physical frailty** | - | - | | - Arts-based (n=1; *n=1) | - |
| **Psychological frailty** | - ICT-based (n=1; *n=0) | - | | - | - |
| **Total frailty** | - ICT-based (n=1; *n=0) | - | | - | - |

**References:**

- Bosc M. Assessment of social functioning in depression. Comprehensive psychiatry. 2000;41(1):63–9
- Cannuscio C, Block J, Kawachi I. Social capital and successful aging: the role of senior housing. Annals of Int Med. 2003;139(5):395–9.
- Gasper D. Understanding the diversity of conceptions of well-being and quality of life. The Journal of socio-economics. 2010;39(3):351–60.
- Johnston KA. Toward a Theory of Social Engagement. In: The Handbook of Communication Engagement. Hoboken, NJ, USA: John Wiley & Sons, Inc; 2018. p. 17–32.
- Manca AR. Social Cohesion. In: Encyclopedia of Quality of Life and Well-being Research. Reference Reviews. 2015 Oct 12;29(7):22–22.
- Veazie S, Gilbert J, Winchell K, Paynter R, Guise JM. Addressing Social Isolation To Improve the Health of Older Adults: A Rapid Review [Internet]. Rockville (MD): Agency for Healthcare Research and Quality (US); 2019 [cited 2024 Mar 27]. (AHRQ Rapid Evidence Product Reports). Available from: <http://www.ncbi.nlm.nih.gov/books/NBK537909/>
- WHOQOL - Measuring Quality of Life| The World Health Organization [Internet]. [cited 2024 Mar 27]. Available from: https://www.who.int/tools/whoqol
