# Supplementary material for: Interventions that have potential to help older adults living with social frailty: a systematic scoping review
Source: BMC Geriatr. 2024 Jun 15;24:521. doi: 10.1186/s12877-024-05096-w (PMC11179268; doi:10.1186/s12877-024-05096-w)
Supplement: Supplementary file 5 — Supplementary Material 5. [file 12877_2024_5096_MOESM5_ESM.docx]

**Supplementary file 5**

References for all tables

Abdel-Aziz HR, El-Sebaie SR. Effect of nursing intervention program on depressive symptoms and feeling of loneliness among elderly people. NeuroQuantology. 2022;20(12):3422.

Åberg P. Nonformal learning and well-being among older adults: links between participation in Swedish study circles, feelings of well-being and social aspects of learning. Educ Gerontol. 2016 Jun;42(6):411–22.

Ae-Ri J, Kowoon L, Eun-A P. Development and evaluation of the information and communication technology-based Loneliness Alleviation Program for community-dwelling older adults: a pilot study and randomized controlled trial. Geriatr Nurs. 2023 Sep-Oct;53:204-211.

Agmon M, Perry CK, Phelan E, Demiris G, Nguyen HQ. A pilot study of Wii Fit exergames to improve balance in older adults. J Geriatr Phys Ther. 2011 Oct 1;34(4):161-7.

Airola E, Rasi P, Outila M. Older people as users and non-users of a video conferencing service for promoting social connectedness and well-being–a case study from Finnish Lapland. Educ Gerontol. 2020 May 3;46(5):258-69.

Alaviani, M., Khosravan, S., Alami, A., Moshki, M. The effect of a multi-strategy program on developing social behaviours based on Pender’s health pro prevent loneliness of old women referred to Gonabad urban health centers. Int. J. Commun. Based Nurs. Midwifery. 2015;3(2), 132–140.

Alcock CL, Camic PM, Barker C, Haridi C, Raven R. Intergenerational practice in the community: a focused ethnographic evaluation. J. Community Appl. Soc. Psychol. 2011 Sep;21(5):419-32.

Allison TA, Nápoles AM, Johnson JK, Stewart AL, Rodriguez-Salazar M, Peringer J, Sherman S, Ortez-Alfaro J, Villero O, Portacolone E. Multi-cultural perspectives on group singing among diverse older adults. Geriatr Nurs. 2020 Nov 1;41(6):1006-12.

Alpozgen AZ, Kardes K, Acikbas E, Demirhan F, Sagir K, Avcil E. The effectiveness of synchronous tele-exercise to maintain the physical fitness, quality of life, and mood of older people-a randomized and controlled study. Eur. Geriatr. Med. 2022 Oct;13(5):1177-85.

Anderson L. Intervention against loneliness in a group of elderly women: an impact evaluation. Soc. Sci. Med. 1985 Jan 1;20(4):355-64.

Appel L, Lewis S, Kisonas E, Recknagel J. VRCHIVE: experiences conducting an online workshop teaching intergenerational participants to create virtual reality films about their lives during the COVID pandemic. Educ Gerontol. 2022 Jul 3;48(7):305-30.

Arnaert A, Delesie L. Effectiveness of video-telephone nursing care for the homebound elderly. Can J Nurs Res. 2007 Mar;39(1):20–36.

Arnetz BB, Theorell T. Psychological, sociological and health behaviour aspects of a long term activation programme for institutionalized elderly people. Soc. Sci. Med. 1983 Jan 1;17(8):449-56.

Arthanat S, Vroman KG, Lysack C. A home-based individualized information communication technology training program for older adults: a demonstration of effectiveness and value. Disabil Rehabil Assist Technol. 2016;11(4):316-24.

Austin EN, Johnston YAM, Morgan LL. Community gardening in a senior center: a therapeutic intervention to improve the health of older adults. Ther Recreat J. (2006) 40:48–56.

Baez M, Far IK, Ibarra F, Ferron M, Didino D, Casati F. Effects of online group exercises for older adults on physical, psychological and social well-being: a randomized pilot trial. PeerJ. 2017 Apr 5;5:e3150.

Báez M, Ibarra F, Far IK, Ferron M, Casati F. Online group-exercises for older adults of different physical abilities. In2016 International Conference on Collaboration Technologies and Systems (CTS) 2016 Oct 31 (pp. 524-533). IEEE.

Baisch S, Kolling T, Schall A, Rühl S, Selic S, Kim Z, Rossberg H, Klein B, Pantel J, Oswald F, Knopf M. Acceptance of social robots by elder people: does psychosocial functioning matter? Int. J. Soc. Robot. 2017 Apr;9:293-307.

Baker S, Warburton J, Waycott J, Batchelor F, Hoang T, Dow B, Ozanne E, Vetere F. Combatting social isolation and increasing social participation of older adults through the use of technology: a systematic review of existing evidence. Australas J Ageing. 2018 Sep;37(3):184-93.

Balasubramanian GV, Beaney P, Chambers R. Digital personal assistants are smart ways for assistive technology to aid the health and well-being of patients and carers. BMC Geriatr. 2021 Dec;21:1-0.

Ballantyne A, Trenwith L, Zubrinich S, Corlis M. "‘I feel less lonely’: what older people say about participating in a social networking website." Qual. Ageing Older Adults. 2010;11(3):25-35.

Balta M, Katsas K, Grigoropoulou C, Diamantis DV, Kalogiannis D, Drougos N, Fagogeni E, Veloudaki A, Panagiotakos D, Linos A. Combating loneliness in older adults during the covid-19 pandemic: findings from a volunteer-based program in Greece. Behav Sci. 2023 Sep 27;13(10):804.

Banbury A, Chamberlain D, Nancarrow S, Dart J, Gray L, Parkinson L. Can videoconferencing affect older people's engagement and perception of their social support in long-term conditions management: a social network analysis from the Telehealth Literacy Project. Health Soc Care Community. 2017 May;25(3):938-950.

Banbury A, Parkinson L, Gordon S, Wood D. Implementing a peer-support programme by group videoconferencing for isolated carers of people with dementia. J Telemed Telecare. 2019 Oct;25(9):572-7.

Bantry-White E, O’Sullivan S, Kenny L, O’Connell C. The symbolic representation of community in social isolation and loneliness among older people: insights for intervention from a rural Irish case study. Health Soc Care Community. 2018 Jul;26(4):e552–9.

Barbosa BT, Da Silva RLS, De Meneses ABC, Brindeiro-Neto W, Bacurau TP, De Souza Rocha AIS, et al. Self-related quality of life of elderly submitted to a 12-week aquatic training program. J Hum Sport Exerc. (2019) 14:281–91.

Barbosa MR, Campinho A, Silva G. “give and receive”: the impact of an intergenerational program on institutionalized children and older adults. J Intergenerational Relationships. 2021 Jul 3;19(3):283-304.

Barbosa Neves B, Franz R, Judges R, Beermann C, Baecker R. Can digital technology enhance social connectedness among older adults? A feasibility study. J Appl Gerontol. 2019 Jan;38(1):49-72.

Barragan C. Social relationships and the importance of community-based fitness programs (CBFP). J Women Aging. (2021) 33:428–41.

Barrera M Jr, Glasgow RE, McKay HG, Boles SM, Feil EG. Do internet based support interventions change perceptions of social support? An experimental trial of approaches for supporting diabetes self-management. Am J Community Psychol. 2002;30:637–654.

Bartholomaeus JD, Van Agteren JEM, Iasiello MP, Jarden A, Kelly D. Positive aging: the impact of a community well-being and resilience program. Clin Gerontol. 2019;42(4):377–86.

Bartlett H, Warburton J, Lui CW, Peach L, Carroll M. Preventing social isolation in later life: findings and insights from a pilot Queensland intervention study. Ageing & Society. 2013 Oct;33(7):1167-89.

Bartlett MY, Arpin SN. Gratitude and loneliness: enhancing health and well-being in older adults. Res Aging. 2019 Sep;41(8):772–93.

Bartsch DA, Rodgers VK, Strong D. Outcomes of senior reach gatekeeper referrals: comparison of the Spokane gatekeeper program, Colorado Senior Reach, and Mid-Kansas Senior Outreach. Care Manag J. 2013 Mar 01;14(1):11-20.

Bartsch DA, Rodgers VK. Senior reach outcomes in comparison with the Spokane Gatekeeper program. Care Management Journals. 2009 Sep 1;10(3):82-8.

Baumgarten M, Thomas D, de Courval LP, Infante-Rivard C. Evaluation of a mutual help network for the elderly residents of planned housing. Psychol Aging. 1988 Dec;3(4):393.

Beauchet O, Matskiv J, Galery K, Goossens L, Lafontaine C, Sawchuk K. Benefits of a 3-month cycle of weekly virtual museum tours in community dwelling older adults: results of a randomized controlled trial. Front Med. 2022 Aug 16;9:2273.

Beausoleil K, Garbarino J, Lewis LF. "I loved interacting with this younger generation": exploring the impact of a virtual service-learning program on social connectedness among older adults during the COVID-19 pandemic. Gerontol Geriatr Educ. 2022 Oct 13:1-19.

Beer JM, Takayama L. Mobile remote presence systems for older adults: acceptance, benefits, and concerns. In Proceedings of the 6th international conference on Human-robot interaction 2011 Mar 6 (pp. 19-26).

Bentley, F.; Basapur, S.; Chowdhury, S.K. Promoting intergenerational communication through location-based asynchronous video communication. In Proceedings of the 2011 ACM International Conference on Ubiquitous Computing, New York, NY, USA, 17 September 2011.

Bickerdike L, Booth A, Wilson PM, Farley K, Wright K. Social prescribing: less rhetoric and more reality. A systematic review of the evidence. BMJ Open. 2017 Apr 7;7(4):e013384.

Bickmore TW, Caruso L, Clough-Gorr K, Heeren T. ‘It’s just like you talk to a friend’ relational agents for older adults. Interact Comput. 2005 Dec;17(6):711–35.

Bidonde MJ, Goodwin DL, Drinkwater DT. Older women's experiences of a fitness program: the importance of social networks. J Appl Sport Psychol. 2009 May 12;21(S1):S86-101.

Billipp SH. The psychosocial impact of interactive computer use within a vulnerable elderly population: a report on a randomized prospective trial in a home health care setting. Public Health Nursing. 2001;18:138–145.

Biniok P, Menke I. Societal participation of the elderly: Information and communication technologies as a “Social Junction.” Anthropol Aging. 2015;36(2):164-81.

Blackburn, N.E., Skjodt, M., Tully, M.A., Mc Mullan, I., Giné-Garriga, M., Caserotti, P., Blancafort, S., Santiago, M., Rodriguez-Garrido, S., Weinmayr, G. and John-Köhler, U., 2021. Older adults’ experiences of a physical activity and sedentary behaviour intervention: a nested qualitative study in the SITLESS multi-country randomised clinical trial. Int J Environ Res Public Health. 2021 May;18(9):4730.

Blažun H, Saranto K, Rissanen S. Impact of computer training courses on reduction of loneliness of older people in Finland and Slovenia. Comput Hum Behav. 2012 Jul 1;28(4):1202-12.

Boekhout JM, Volders E, Bolman CA, de Groot RH, Lechner L. Long-term effects on loneliness of a computer-tailored intervention for older adults with chronic diseases: a randomized controlled trial. J Aging Health. 2021 Dec;33(10):865-76.

Bond GE, Burr RL, Wolf FM, Feldt K. The effects of a web-based intervention on psychosocial well-being among adults aged 60 and older with diabetes: a randomized trial. Diabetes Educ. 2010;36:446–456.

Borji M, Tarjoman A. Investigating the effect of religious intervention on mental vitality and sense of loneliness among the elderly referring to community healthcare centers. J Relig Health. 2020 Feb;59(1):163–72.

Bornemann R. The impact of information and communication technology (ICT) usage on social isolation including loneliness in older adults. A systematic review. Magdeburg/Stendal University of Applied Sciences, 2014 M.Sc Rehabilitation Psychology; Magdeburg /Stendal University of Applied Sciences. https://www.researchgate.net/publication/316740931_The_impact_of_information_and_communication_technology_ICT_usage_on_social_isolation_including_loneliness_in_older_adults_A_systematic_review.

Botner E. Impact of a virtual learning program on social isolation for older adults. TRJ. 2018;52(2):126–39.

Bouwman TE, Aartsen MJ, van Tilburg TG, Stevens NL. Does stimulating various coping strategies alleviate loneliness? Results from an online friendship enrichment program. J Soc Pers Relat. 2017 Sep;34(6):793-811.

Bowes A, McColgan G. Telecare for older people: promoting independence, participation, and identity. Res Aging. 2013;35(1):32-49.

Boyes M. Outdoor adventure and successful ageing. Ageing Soc. 2013;33:644–65.

Brady S, D’Ambrosio LA, Felts A, Rula EY, Kell KP, Coughlin JF. Reducing isolation and loneliness through membership in a fitness program for older adults: implications for health. J Appl Gerontol. 2020 Mar;39(3):301–10.

Brandão, L.; Bauer, M.A.; Haas, A.N.; Silveira, R.D.S.; Alves, C.P.; Souza, D.N.; Beber, B.C.; Oliveira, W.F. Playing remotely in times of crisis: a program to overcome social isolation. Int. J. Geriatr. Psychiatry 2021, 37, 1–12.

Brandenburgh A, van Breda W, van der Ham W, Klein M, Moeskops L, Roelofsma P. Increasing physical and social activity through virtual coaching in an ambient environment. In Active Media Technology: 10th International Conference, AMT 2014, Warsaw, Poland, August 11-14, 2014. Proceedings 10 2014 (pp. 487-500). Springer International Publishing.

Breck BM, Dennis CB, Leedahl SN. Implementing reverse mentoring to address social isolation among older adults. J Gerontol Soc Work. 2018 Jul;61(5):513-525.

Brimelow RE, Wollin JA. Loneliness in old age: interventions to curb loneliness in long-term care facilities. Act Adapt Aging. 2017 Oct 2;41(4):301-15.

Bruce ML, Pepin R, Marti CN, Stevens CJ, Choi NG. One year impact on social connectedness for Homebound older adults: randomized controlled trial of Tele-delivered behavioral activation versus Tele-delivered friendly visits. Am J Geriatr Psychiatry. 2021 Aug 1;29(8):771-6.

Brustio PR, Liubicich ME, Chiabrero M, Rabaglietti E. Dancing in the golden age: a study on physical function, quality of life, and social engagement. Geriatr Nurs. 2018 Nov;39(6):635-639.

Burmeister OK, Bernoth M, Dietsch E, Cleary M. Enhancing connectedness through peer training for community-dwelling older people: a person centred approach. Issues Ment Health Nurs. 2016 Jun 2;37(6):406–11.

Butler SS. Evaluating the Senior Companion Program: a mixed-method approach. J Gerontol Soc Work. 2006;47(1–2):45–70.

Carandang RR, Shibanuma A, Kiriya J, Vardeleon KR, Asis E, Murayama H, Jimba M. Effectiveness of peer counseling, social engagement, and combination interventions in improving depressive symptoms of community-dwelling Filipino senior citizens. PLoS One. 2020 Apr 1;15(4):e0230770.

Carlson MC, Saczynski JS, Rebok GW, Seeman T, Glass TA, McGill S, Tielsch J, Frick KD, Hill J, Fried LP. Exploring the effects of an “everyday” activity program on executive function and memory in older adults: Experience Corps®. Gerontologist. 2008 Dec 1;48(6):793-801.

Carstensen L, Mason SE, Caldwell EC. Children's attitudes toward the elderly: an intergenerational technique for change. Educ Gerontol. 1982 May 1;8(3):291-301.

Casanova G, Zaccaria D, Rolandi E, Guaita A. The effect of information and communication technology and social networking site use on older people’s well-being in relation to loneliness: review of experimental studies. J Med Internet Res. 2021 Mar 1;23(3):e23588.

Cattan M, Kime N, Bagnall AM. The use of telephone befriending in low level support for socially isolated older people–an evaluation. Health Soc Care Community. 2011 Mar;19(2):198-206.

Cedergren A, King KA, Wagner DI, Wegley S. Perceived social health benefits among participants in a countrywide senior chair volleyball program. Act Adapt Aging. (2007) 31:23–36.

Cesta A, Cortellessa G, Orlandini A, Tiberio L. Long-term evaluation of a telepresence robot for the elderly: methodology and ecological case study. Int J Soc Robot. 2016 Jun;8:421-41.

Chan AW, Yu DS, Choi KC. Effects of tai chi qigong on psychosocial well-being among hidden elderly, using elderly neighborhood volunteer approach: a pilot randomized controlled trial. Clin Interv Aging. 2017;12:85–96.

Chao LL, Lee JA, Martinez S, Barlow C, Chesney MA, Mehling WE, Barnes DE. Preventing loss of independence through exercise (PLIÉ): a pilot trial in older adults with subjective memory decline and mild cognitive impairment. J Alzheimers Dis. 2021 Jan 1;82(4):1543-57.

Chao YY, Musanti R, Zha P, Katigbak C. The feasibility of an exergaming program in underserved older African Americans. West J Nurs Res. 2018 Jun;40(6):815-33.

Chen AT, Ge S, Cho S, Teng AK, Chu F, Demiris G, Zaslavsky O. Reactions to COVID-19, information and technology use, and social connectedness among older adults with pre-frailty and frailty. Geriatr Nurs. 2021 Jan 1;42(1):188-95.

Chen E, Wood D, Ysseldyk R. Online social networking and mental health among older adults: a scoping review. Can J Aging/La Rev Can Vieil. 2022 Mar;41(1):26-39.

Chen YR, Schulz PJ. The effect of information communication technology interventions on reducing social isolation in the elderly: a systematic review. J Med Internet Res. 2016 Jan 28;18(1):e4596.

Chen, M.-F., Tsai, C.-C. The effectiveness of a thanks, sorry, love, and farewell board game in older people in Taiwan: a quasi-experimental study. Int J Environ Res Public Health. 2022;19(5):3146.

Chi NC, Sparks O, Lin SY, Lazar A, Thompson HJ, Demiris G. Pilot testing a digital pet avatar for older adults. Geriatr Nurs. 2017 Nov-Dec;38(6):542-547.

Chiu CJ, Wu CH. Information and Communications technology as a health promotion method for older adults in assisted-living facilities: three-arm group-randomized trial. JMIR Aging. 2019 May 6;2(1):e12633. doi: 10.2196/12633.

Chiu T, Marziali E, Colantonio A, Carswell A, Gruneir M, Tang M, Eysenbach G. Internet-based caregiver support for Chinese Canadians taking care of a family member with Alzheimer disease and related dementia. Can J Aging. 2009 Dec;28(4):323-36. doi: 10.1017/S0714980809990114.

Choi HK, Lee SH. Trends and effectiveness of ICT interventions for the elderly to reduce loneliness: a systematic review. InHealthcare. 2021 Mar 7;9(3):293. doi: 10.3390/healthcare9030293.

Choi M, Kong S, Jung D. Computer and internet interventions for loneliness and depression in older adults: a meta-analysis. Health Inform Res. 2012 Sep 30;18(3):191-8. doi: 10.4258/hir.2012.18.3.191.

Choi NG, Marti CN, Wilson NL, Chen GJ, Sirrianni L, Hegel MT, Bruce ML, Kunik ME. Effect of telehealth treatment by lay counselors vs by clinicians on depressive symptoms among older adults who are homebound: a randomized clinical trial. JAMA Netw Open. 2020 Aug 3;3(8):e2015648. doi: 10.1001/jamanetworkopen.2020.15648.

Choi NG, Pepin R, Marti CN, Stevens CJ, Bruce ML. Improving social connectedness for homebound older adults: randomized controlled trial of tele-delivered behavioral activation versus tele-delivered friendly visits. Am J Geriatr Psychiatry. 2020 Jul 1;28(7):698-708. doi: 10.1016/j.jagp.2020.02.004.

Chow AYM, Caserta M, Lund D, Suen MHP, Xiu D, Chan IKN, et al. Dual-process bereavement group intervention (DPBGI) for widowed older adults. Gerontologist. 2019;59(5):983-94. doi: 10.1093/geront/gny048.

Christie HL, Dam AE, van Boxtel M, Köhler S, Verhey F, de Vugt ME. Lessons learned from an effectiveness evaluation of inlife, a web-based social support intervention for caregivers of people with dementia: randomized controlled trial. JMIR Aging. 2022 Dec 7;5(4):e38656. doi: 10.2196/38656.

Clarke M, Clarke SJ, Jagger C. Social intervention and the elderly: a randomized controlled trial. Am J Epidemiol. 1992 Dec 15;136(12):1517-23.

Clifford AM, Shanahan J, O'Leary H, O'Neill D, Ni Bhriain O. Social dance for health and well-being in later life. Complement Ther Clin Pract. 2019 Nov;37:6-10. doi: 10.1016/j.ctcp.2019.07.003.

Clift S, Skingley A, Coulton S, Rodriguez J. A controlled evaluation of the health benefits of a participative community singing programme for older people (Silver Song Clubs). Sidney De Haan Research Centre for Arts and Health, Canterbury Christ Church University, Folkestone, Kent, UK. 2012.

Cochrane Public Health Group, Noone C, McSharry J, Smalle M, Burns A, Dwan K, Devane D, Morrissey EC. Video calls for reducing social isolation and loneliness in older people: a rapid review. Cochrane Database Syst Rev. 2020 Jul 1;2020(7):CD013632. doi: 10.1002/14651858.CD013632.

Coffman D. Survey of new horizons international music association musicians. Int J Community Music. 2009 Mar 1;1(3):375-90.

Cohen GD, Perlstein S, Chapline J, Kelly J, Firth KM, Simmens S. The impact of professionally conducted cultural programs on the physical health, mental health, and social functioning of older adults. Gerontologist. 2006 Dec 1;46(6):726-34.

Cohen GD, Perlstein S, Chapline J, Kelly J, Firth KM, Simmens S. The impact of professionally conducted cultural programs on the physical health, mental health, and social functioning of older adults—2-year results. J Aging Human Arts. 2007 Jun 8;1(1-2):5-22. doi: 10.1080/19325610701410791.

Cohen-Mansfield J, Hazan H, Lerman Y, Shalom V, Birkenfeld S, Cohen R. Efficacy of the I-SOCIAL intervention for loneliness in old age: lessons from a randomized controlled trial. J Psychiatr Res. 2018 Apr 1;99:69-75. doi: 10.1016/j.jpsychires.2017.12.017.

Cohen-Mansfield J, Muff A, Meschiany G, Lev-Ari S. Adequacy of web-based activities as a substitute for in-person activities for older persons during the covid-19 pandemic: survey study. J Med Internet Res. 2021 Jan 22;23(1):e25848. doi: 10.2196/25848. Erratum in: J Med Internet Res. 2021 Feb 16;23(2):e27687.

Cohen-Mansfield J, Muff A. Comparing community-based intergenerational activities in israel: participants, programs, and perceived outcomes. J Gerontol Soc Work. 2022 Jul 4;65(5):495-511. doi: 10.1080/01634372.2021.1985782.

Cohen-Mansfield J, Perach R. Interventions for alleviating loneliness among older persons: a critical review. Am J Health Promot. 2015 Jan;29(3):e109-25. doi: 10.4278/ajhp.130218-LIT-70.

Coll-Planas L, del Valle Gomez G, Bonilla P, Masat T, Puig T, Monteserin R. Promoting social capital to alleviate loneliness and improve health among older people in Spain. Health Soc Care Community. 2017 Jan;25(1):145-57. doi: 10.1111/hsc.12292.

Coll-Planas L, Rodríguez-Arjona D, Pons-Vigués M, Nyqvist F, Puig T, Monteserín R. "not alone in loneliness": a qualitative evaluation of a program promoting social capital among lonely older people in primary health care. Int J Environ Res Public Health. 2021 May 23;18(11):5580. doi: 10.3390/ijerph18115580.

Collins CC, Benedict J. Evaluation of a community-based health promotion program for the elderly: lessons from Seniors CAN. Am J Health Promot. 2006 Sep;21(1):45-8. doi: 10.4278/0890-1171-21.1.45.

Conroy KM, Krishnan S, Mittelstaedt S, Patel SS. Technological advancements to address elderly loneliness: practical considerations and community resilience implications for COVID-19 pandemic. Working with Older People. 2020 Dec 14;24(4):257-64. doi: 10.1108/WWOP-07-2020-0030.

Constantino RE. Comparison of two group interventions for the bereaved. Image: J Nurs Scholarsh. 1988 Jun;20(2):83-7.

Cornejo R, Tentori M, Favela J. Enriching in-person encounters through social media: A study on family connectedness for the elderly. Int J Hum-Comput Stud. 2013 Sep 1;71(9):889-99. doi: 10.1016/j.ijhcs.2013.05.006.

Costello L, McDermott ML, Patel P, Dare J. ‘A lot better than medicine’-self-organised ocean swimming groups as facilitators for healthy ageing. Health Place. 2019 Nov 1;60:102212. doi: 10.1016/j.healthplace.2019.102212.

Cotten SR, Anderson WA, McCullough BM. Impact of internet use on loneliness and contact with others among older adults: cross-sectional analysis. J Med Internet Res. 2013 Feb 28;15(2):e39. doi: 10.2196/jmir.2306.

Cotten SR, Ford G, Ford S, Hale TM. Internet use and depression among older adults. Comput Human Behav. 2012 Mar 1;28(2):496-9. doi: 10.1016/j.chb.2011.10.021.

Cox EO, Green KE, Hobart K, Jang LJ, Seo H. Strengthening the late-life care process: effects of two forms of a care-receiver efficacy intervention. Gerontologist. 2007 Jun;47(3):388–97.

Creswell JD, Irwin MR, Burklund LJ, Lieberman MD, Arevalo JM, Ma J, Breen EC, Cole SW. Mindfulness-based stress reduction training reduces loneliness and pro-inflammatory gene expression in older adults: a small randomized controlled trial. Brain Behav Immun. 2012 Oct;26(7):1095-101.

Cristancho-Lacroix V, Wrobel J, Cantegreil-Kallen I, Dub T, Rouquette A, Rigaud AS. A web-based psychoeducational program for informal caregivers of patients with Alzheimer’s disease: a pilot randomized controlled trial. J Med Internet Res. 2015 May 12;17(5):e117.

Cryer S, Henderson-Wilson C, Lawson J. Pawsitive Connections: The role of pet support programs and pets on the elderly. Complement Ther Clin Pract. 2021 Feb 1;42:101298.

Cutler C, Hicks B, Innes A. Does digital gaming enable healthy aging for community-dwelling people with dementia? Games Cult. 2016 Jan;11(1-2):104-29.

Czaja SJ, Boot WR, Charness N, Rogers WA, Sharit J, Fisk AD, Lee CC, Nair SN. The personalized reminder information and social management system (PRISM) trial: rationale, methods and baseline characteristics. Contemp Clin Trials. 2015 Jan 1;40:35-46.

Czaja SJ, Boot WR, Charness N, Rogers WA, Sharit J. improving social support for older adults through technology: findings from the prism randomized controlled trial. Gerontologist. 2018 May 8;58(3):467-477.

Dabkowski E, Porter JE, Barbagallo M. A thematic analysis of the perceptions of a community-based exercise program on the health and well-being of ageing adults. Health Soc Care Community. 2021 Nov;29(6):1990-1997.

Damne S, Djabelkhir C, Ware C, Benovici J, Wu W-H, Kerherve H, Rigaud A-S. The Café-multimedia: a promising psychosocial intervention for frail older adults. Eur Geriatr Med. 2017: P060.

Davidson JW, McNamara B, Rosenwax L, Lange A, Jenkins S, Lewin G. Evaluating the potential of group singing to enhance the well-being of older people. Australas J Ageing. 2014 Jun;33(2):99-104.

de Craen AJM, Gussekloo J, Blauw GJ, Willems CG, Westendorp RGJ. Randomised controlled trial of unsolicited occupational therapy in community-dwelling elderly people: the LOTIS trial. PLoS Clin Trials. 2006 May;1(1):e2.

de Souza EM, Grundy E. Intergenerational interaction, social capital and health: results from a randomised controlled trial in Brazil. Soc Sci Med. 2007 Oct 1;65(7):1397-409.

Delello JA, McWhorter RR. Reducing the digital divide: connecting older adults to ipad technology. J Appl Gerontol. 2017 Jan;36(1):3–28.

Dew MA, Goycoolea JM, Harris RC, Lee A, Zomak R, Dunbar-Jacob J, Rotondi A, Griffith BP, Kormos RL. An internet-based intervention to improve psychosocial outcomes in heart transplant recipients and family caregivers: development and evaluation. J Heart Lung Transplant. 2004 Jun;23(6):745-58.

Dhillon JS, Ramos C, Wunsche BC, Lutteroth C. Designing a web-based telehealth system for elderly people: an interview study in New Zealand. In: 2011 24th Int Symp Comput-Based Med Syst (CBMS). IEEE; 2011. p. 1–6.

Dickens AP, Richards SH, Greaves CJ, Campbell JL. Interventions targeting social isolation in older people: a systematic review. BMC Public Health. 2011 Dec;11(1):1.

Dickens AP, Richards SH, Hawton A, Taylor RS, Greaves CJ, Green C, Edwards R, Campbell JL. An evaluation of the effectiveness of a community mentoring service for socially isolated older people: a controlled trial. BMC Public Health. 2011 Dec;11:1-4.

Dinkins CS. Socrates Café for older adults: intergenerational connectedness through facilitated conversation. J Psychosoc Nurs Ment Health Serv. 2019 Jan;57(1):11–5.

Dionigi R. Resistance training and older adults’ beliefs about psychological benefits: the importance of self-efficacy and social interaction. J Sport Exerc Psychol. (2007) 29:723–46.

Diwan S, Eliazar A, Pham D, Fuentes M. Evaluation of a culturally adapted reminiscence therapy intervention: improving mood, family and community connectedness in Spanish-and Vietnamese-speaking older adults. Transcult Psychiatry. 2023 Aug 24:13634615231191996.

Dodge HH, Zhu J, Mattek N, Bowman M, Ybarra O, Wild K, Loewenstein DA, Kaye JA. Web-enabled conversational interactions as a means to improve cognitive functions: results of a 6-week randomized controlled trial. Alzheimers Dement (N Y). 2015 May;1(1):1-12.

Dolovich L, Oliver D, Lamarche L, Thabane L, Valaitis R, Agarwal G, Carr T, Foster G, Griffith L, Javadi D, Kastner M. Combining volunteers and primary care teamwork to support health goals and needs of older adults: a pragmatic randomized controlled trial. CMAJ. 2019 May 6;191(18):E491-500.

Don'L B. A faith-based intervention to address social isolation and loneliness in older adults. J Christ Nurs. 2023 Jan 1;40(1):28-35.

Douka S, Zilidou VI, Lilou O, Tsolaki M. Greek traditional dances: a way to support intellectual, psychological, and motor functions in senior citizens at risk of neurodegeneration. Front Aging Neurosci. 2019 Jan 25;11:6.

Drentea P, Clay OJ, Roth DL, Mittelman MS. Predictors of improvement in social support: five-year effects of a structured intervention for caregivers of spouses with Alzheimer's disease. Soc Sci Med. 2006 Aug 1;63(4):957-67.

Düzel S, Drewelies J, Polk SE, Misgeld C, Porst J, Wolfarth B, Kühn S, Brandmaier AM, Wenger E. No evidence for a boost in psychosocial functioning in older age after a 6-months physical exercise intervention. Front Hum Neurosci. 2022 Mar 11;16:825454.

Ehlers DK, Daugherty AM, Burzynska AZ, Fanning J, Awick EA, ChaddockHeyman L, et al. Regional brain volumes moderate, but do not mediate, the effects of group-based exercise training on reductions in loneliness in older adults. Front Aging Neurosci. (2017) 9:110.

Eliezer K, Knei-Paz C, Zvi L, Schnall I, Gitlitz T, Gavriel-Fried B. ‘someone to talk to’: a short-term tele-therapy intervention with older people during the coronavirus pandemic. Br J Soc Work. 2022 Jun 1;52(4):2367-83.

Emas S, Montoya L, Chen A, Tran C, Tran P, Dharni A. Empowering older adults: incorporating technology for retirement adjustment. Phys Occup Ther Geriatr. 2018 Jul 3;36(2-3):245-57.

Engelbrecht R, Shoemark H. The acceptability and efficacy of using iPads in music therapy to support well-being with older adults: A pilot study. Aust J Music Ther. 2015 Jan;26:52-73.

Evans RL, Smith KM, Werkhoven WS, Fox HR, Pritzl DO. Cognitive Telephone group therapy with physically disabled elderly persons. Gerontologist. 1986 Feb 1;26(1):8–11.

Fields J, Cemballi A, Michalec C, Uchida D, DeSmidt H, Cuellar J, Chodos A, Lyles CR. In-home technology training to reduce social isolation among older adults: preliminary findings from the Tech Allies program. J Am Geriatr Soc. 2019;67:S226. https://doi.org/10.1177/0733464820910028

Fields J, Cemballi AG, Michalec C, Uchida D, Griffiths K, Cardes H, Cuellar J, Chodos AH, Lyles CR. In-home technology training among socially isolated older adults: findings from the tech allies program. J Appl Gerontol. 2021 May;40(5):489-499.

Fields N, Xu L, Greer J, Murphy E. Shall I compare thee… to a robot? An exploratory pilot study using participatory arts and social robotics to improve psychological well-being in later life. Aging Ment Health. 2021 Mar 4;25(3):575-84.

Fields NL, Lee K, Cassidy J, Kunz-Lomelin A, Stringfellow MK, Feinhals G. It gave me somebody else to think about besides myself: caring callers volunteer experiences with a telephone-based reassurance program for socially isolated older adults. J Appl Gerontol. 2023 Jan;42(1):49-58.

Figueira HA, Figueira AA, Cader SA, Guimarães AC, De Oliveira RJ, Figueira JA, et al. Effects of a physical activity governmental health programme on the quality of life of elderly people. Scand J Public Health. 2012;40:418–22.

Findlay RA. Interventions to reduce social isolation amongst older people: where is the evidence? Ageing Soc. 2003 Sep;23(5):647–58.

Fokkema T, Knipscheer K. Escape loneliness by going digital: a quantitative and qualitative evaluation of a Dutch experiment in using ECT to overcome loneliness among older adults. Aging Ment Health. 2007 Sep;11(5):496-504.

Forsman AK, Nordmyr J. Psychosocial links between internet use and mental health in later life: a systematic review of quantitative and qualitative evidence. J Appl Gerontol. 2017 Dec;36(12):1471-518.

Franck L, Molyneux N, Parkinson L. Systematic review of interventions addressing social isolation and depression in aged care clients. Qual Life Res. 2016 Jun;25:1395-407.

Franke T, Sims-Gould J, Nettlefold L, Ottoni C, McKay HA. “It makes me feel not so alone”: features of the Choose to Move physical activity intervention that reduce loneliness in older adults. BMC Public Health. 2021 Dec;21:1-5.

Franse CB, van Grieken A, Alhambra-Borrás T, Valía-Cotanda E, van Staveren R, Rentoumis T, et al. The effectiveness of a coordinated preventive care approach for healthy ageing (UHCE) among older persons in five European cities: a pre-post controlled trial. Int J Nurs Stud. 2018;88:153–62.

Freeman S, Martin J, Nash C, Hausknecht S, Skinner K. Use of a digital storytelling workshop to foster development of intergenerational relationships and preserve culture with the Nak’azdli first nation: Findings from the Nak’azdli Lha’hutit’en project. Can J Aging. 2020;39:284–93.

Fried LP, Carlson MC, Freedman M, Frick KD, Glass TA, Hill J, et al. A social model for health promotion for an aging population: initial evidence on the Experience Corps model. J Urban Health. 2004 Mar;81:64-78.

Friedman EM, Ruini C, Foy R, Jaros L, Sampson H, Ryff CD. Lighten UP! A community-based group intervention to promote psychological well-being in older adults. Aging Ment Health. 2017 Feb 1;21(2):199-205.

Fu MC, Belza B, Nguyen H, Logsdon R, Demorest S. Impact of group-singing on older adult health in senior living communities: A pilot study. Arch Gerontol Geriatr. 2018 May-Jun;76:138-146.

Fujiwara Y, Sakuma N, Ohba H, Nishi M, Lee S, Watanabe N, et al. Effects of an intergenerational health promotion program for older adults in Japan. J Intergenerational Relationships. 2009 Mar 12;7(1):17-39.

Fullen MC, Smith JL, Clarke PB, Westcott JB, McCoy R, Tomlin CC. Holistic wellness coaching for older adults: preliminary evidence for a novel wellness intervention in senior living communities. J Appl Gerontol. 2023 Mar;42(3):427-37.

Fuller SM, Kotwal AA, Tha SH, Hill D, Perissinotto C, Myers JJ. Key elements and mechanisms of a peer-support intervention to reduce loneliness and isolation among low-income older adults: a qualitative implementation science study. J Appl Gerontol. 2022 Dec;41(12):2574-82.

Gaggioli A, Morganti L, Bonfiglio S, Scaratti C, Cipresso P, Serino S, Riva G. Intergenerational group reminiscence: a potentially effective intervention to enhance elderly psychosocial well-being and to improve children's perception of aging. Educ Gerontol. 2014 Jul 1;40(7):486-98.

Gagliardi C, Pillemer K, Gambella E, Piccinini F, Fabbietti P. Benefits for older people engaged in environmental volunteering and socializing activities in city parks: preliminary results of a program in italy. Int J Environ Res Public Health. 2020 May 26;17(11):3772.

Gagliardi C, Santini S, Piccinini F, Fabbietti P, di Rosa M. A pilot programme evaluation of social farming horticultural and occupational activities for older people in Italy. Health Soc Care Commun. 2019;27:207–14.

Galinha IC, García-Martín M, Lima ML. Sing4Health: randomised controlled trial of the effects of a singing group program on the subjective and social well-being of older adults. Appl Psychol Health Well Being. 2022;14(1):176–195.

Gamliel T, Gabay N. Knowledge exchange, social interactions, and empowerment in an intergenerational technology program at school. Educ Gerontol. 2014 Aug 1;40(8):597-617.

Garattini C, Wherton J, Prendergast D. Linking the lonely: an exploration of a communication technology designed to support social interaction among older adults. Univ Access Inf Soc. 2012 Jun;11(2):211-22.

Gardiner C, Barnes S. The impact of volunteer befriending services for older people at the end of life: mechanisms supporting well-being. Prog Palliat Care. 2016 May;24(3):159–64.

Gardiner C, Geldenhuys G, Gott M. Interventions to reduce social isolation and loneliness among older people: an integrative review. Health Soc Care Community. 2018 Mar;26(2):147-57.

Gasteiger N, Loveys K, Law M, Broadbent E. Friends from the future: a scoping review of research into robots and computer agents to combat loneliness in older people. Clin Interv Aging. 2021 May 24:941-71.

Gilbody S, Littlewood E, McMillan D, Chew-Graham CA, Bailey D, Gascoyne S, Sloan C, Burke L, Coventry P, Crosland S, Fairhurst C. Behavioural activation to prevent depression and loneliness among socially isolated older people with long-term conditions: the BASIL COVID-19 pilot randomised controlled trial. PLoS Med. 2021 Oct 12;18(10):e1003779.

Gleibs IH, Haslam C, Jones JM, Alexander Haslam S, McNeill J, Connolly H. No country for old men? The role of a ‘Gentlemen's Club’ in promoting social engagement and psychological well-being in residential care. Aging Ment Health. 2011 May 1;15(4):456-66.

Gomeñuka NA, Oliveira HB, Silva ES, Costa RR, Kanitz AC, Liedtke GV, et al. Effects of Nordic walking training on quality of life, balance and functional mobility in elderly: a randomized clinical trial. PLoS ONE. 2019;14:e0211472.

Gorenko JA, Moran C, Flynn M, Dobson K, Konnert C. Social isolation and psychological distress among older adults related to COVID-19: a narrative review of remotely-delivered interventions and recommendations. J Appl Gerontol. 2021 Jan;40(1):3-13.

Gosline MB. Client participation to enhance socialization for frail elders. Geriatr Nurs. 2003 Sep 1;24(5):286-9.

Goumopoulos C, Papa I, Stavrianos A. Development and evaluation of a mobile application suite for enhancing the social inclusion and well-being of seniors. In Informatics 2017 Jun 22 (Vol. 4, No. 3, p. 15). MDPI.

Gouvêa JA, Antunes MD, Bortolozzi F, Marques AG, Bertolini SM. Impact of Senior Dance on emotional and motor parameters and quality of life of the elderly. Rev Rene. 2017;18(1):51-8.

Gracia N, Moyle W, Oxlade D, Radford K. Addressing loneliness in a retirement village community: a pilot test of a print-delivered intervention. Australas J Ageing. 2010 Dec;29(4):179-82.

Graham C, Scharlach AE, Kurtovich E. Do villages promote aging in place? Results of a longitudinal study. J Appl Gerontol. 2018 Mar;37(3):310-31.

Graham CL, Scharlach AE, Price Wolf J. The impact of the “village” model on health, well being, service access, and social engagement of older adults. Health Educ Behav. 2014 Oct;41(1_suppl):91S-7S.

Graham CL, Scharlach AE, Stark B. Impact of the village model: results of a national survey. J Gerontol Soc Work. 2017 Jul 4;60(5):335-54.

Granbom, M., Kristensson, J., & Sandberg, M. (2017). Effects on leisure activities and social participation of a case management intervention for frail older people living at home: a randomised controlled trial. Health Soc Care Community, 25(4), 1416–1429. https://doi.org/10.1111/hsc.12442.

Greaves CJ, Farbus L. Effects of creative and social activity on the health and well-being of socially isolated older people: outcomes from a multi-method observational study. J R Soc Promot Health. 2006 May;126(3):134–42.

Gross H, Mueller S, Schroeter C, et al. Robot companion for domestic health assistance: implementation, test and case study under everyday conditions in private apartments. In: 2015 IEEE/RSJ International Conference on Intelligent Robots and Systems (IROS). IEEE; 2015.

Gruenewald TL, Tanner EK, Fried LP, Carlson MC, Xue QL, Parisi JM, Rebok GW, Yarnell LM, Seeman TE. The Baltimore Experience Corps Trial: enhancing generativity via intergenerational activity engagement in later life. J Gerontol B Psychol Sci Soc Sci. 2016 Jul 1;71(4):661-70.

Gustafson DH, McTavish FM, Stengle W et al. Use and impact of eHealth system by low-income women with breast cancer. J Health Commun. 2005;10(Suppl. 1):195–218.

Gustafson Jr DH, Gustafson Sr DH, Cody OJ, Chih MY, Johnston DC, Asthana S. Pilot test of a computer-based system to help family caregivers of dementia patients. J Alzheimers Dis. 2019 Jan 1;70(2):541-52.

Gustafson Sr DH, Kornfield R, Mares ML, Johnston DC, Cody OJ, Yang EF, Gustafson Jr DH, Hwang J, Mahoney JE, Curtin JJ, Tahk A. Effect of an eHealth intervention on older adults’ quality of life and health-related outcomes: a randomized clinical trial. J Gen Intern Med. 2022 Feb;37(3):521-30.

Gustafsson S, Berglund H, Faronbi J, Barenfeld E, Ottenvall Hammar I. Minor positive effects of health-promoting senior meetings for older community-dwelling persons on loneliness, social network, and social support. Clin Interv Aging. 2017;12:1867–1877.

Gutierrez FJ, Ochoa SF, Vassileva J. Mediating intergenerational family communication with computer-supported domestic technology. In: Proceedings of the CYTED-RITOS International Workshop on Groupware. Springer, Cham, Switzerland; 2017.

Hagan R, Manktelow R, Taylor BJ, Mallett J. Reducing loneliness amongst older people: a systematic search and narrative review. Aging Ment Health. 2014 Aug 18;18(6):683-93.

Hall N, De Beck P, Johnson D, Mackinnon K, Gutman G, Glick N. Randomized trial of a health promotion program for frail elders. Can J Aging. 1992;11(1):72-91.

Hallam S, Creech A, Varvarigou M, McQueen H, Gaunt H. Does active engagement in community music support the well-being of older people?. Arts Health. 2014 May 4;6(2):101-16.

Hallam S, Creech A. Can active music making promote health and well-being in older citizens? Findings of the music for life project. London J Prim Care (Abingdon). 2016 Mar 11;8(2):21-25.

Halpin SN, Dillard RL, Idler E, Clevenger C, Rothschild E, Blanton S, Wilson J, Flacker JM. The benefits of being a senior mentor: cultivating resilience through the mentorship of health professions students. Gerontol Geriatr Educ. 2017 Jul 3;38(3):283-94.

Hand C, Schouten K, Dellamora M, Letts L, Drenth T. Exploring neighbourhood-based programming for older adults: a seniors’ satellite. Act Adapt Aging. 2022 Jul 3;46(3):190-217.

Harris JE, Bodden JL. An activity group experience for disengaged elderly persons. J Couns Psychol. 1978 Jul;25(4):325–30.

Hausknecht S, Vanchu-Orosco M, Kaufman D. Digitising the wisdom of our elders: connectedness through digital storytelling. Ageing Soc. 2019;39:2714–2734.

Hausknecht S, Vanchu-Orosco M, Kaufman D. Sharing life stories: design and evaluation of a digital storytelling workshop for older adults. In: Communications in Computer and Information Science; Costagliola G, Uhomoibhi J, Zvacek S, McLaren BM, Eds. Springer: Cham, Switzerland, 2017; Volume 739, pp. 497–512.

Heins P, Boots LM, Koh WQ, Neven A, Verhey FR, de Vugt ME. The effects of technological interventions on social participation of community-dwelling older adults with and without dementia: a systematic review. J Clin Med. 2021 May 25;10(11):2308.

Heller K, Thompson MG, Trueba PE, Hogg JR, Vlachos-Weber I. Peer support telephone dyads for elderly women: was this the wrong intervention? Am J Community Psychol. 1991;19(1):53–74.

Hemberg J, Santamäki Fischer R. A window toward the world: older adults' experiences of becoming in health and developing as human beings through interacting with others using real video communication. Holist Nurs Pract. 2018 Mar/Apr;32(2):90-97.

Hemingway A, Jack E. Reducing social isolation and promoting well-being in older people. Qual Ageing Older Adults. 2013 Mar 8;14(1):25-35.

Hernández-Ascanio J, Perula-de Torres LÁ, Rich-Ruiz M, González-Santos J, Mielgo-Ayuso J, González-Bernal J; ASyS Study Collaborative Group. Effectiveness of a multicomponent intervention to reduce social isolation and loneliness in community-dwelling elders: a randomized clinical trial. Nurs Open. 2023 Jan;10(1):48-60.

Hill W, Weinert C, Cudney S. Influence of a computer intervention on the psychological status of chronically ill rural women: preliminary results. Nurs Res. 2006;55:34–42.

Hillman S. Participatory singing for older people: a perception of benefit. Health Educ. 2002 Aug 1;102(4):163-71.

Hind D, Mountain G, Gossage-Worrall R, Walters SJ, Duncan R, Newbould L, Rex S, Jones C, Bowling A, Cattan M, Cairns A, Cooper C, Goyder E, Edwards RT. Putting Life in Years (PLINY): a randomised controlled trial and mixed-methods process evaluation of a telephone friendship intervention to improve mental well-being in independently living older people. Southampton (UK): NIHR Journals Library; 2014 Dec.

Ho RT, Fong TC, Chan WC, Kwan JS, Chiu PK, Yau JC, Lam LC. Psychophysiological effects of dance movement therapy and physical exercise on older adults with mild dementia: a randomized controlled trial. J Gerontol B Psychol Sci Soc Sci. 2020 Feb 14;75(3):560-70.

Honigh-de Vlaming R, Haveman-Nies A, Heinrich J, van’t Veer P, de Groot LCPGM. Effect evaluation of a two-year complex intervention to reduce loneliness in non-institutionalised elderly Dutch people. BMC Public Health. 2013 Oct;13:984.

Hopman-Rock M, Westhoff MH. Development and evaluation of “aging well and healthily”: a health-education and exercise program for community-living older adults. J Aging Phys Act. 2002 Oct 1;10(4):364-81.

Hoyumpa G, Narro A, Law M, Ciavarra B, Phung V, Chiang S, Oyler T, Lydick H, Flores R, Lee J, Burnett J. Medical student-led social phone calls with elder mistreatment victims: changes in loneliness, depression, and perspectives of aging. J Am Geriatr Soc. 2022 Jun;70(6):1876-1878.

Hudson J, Ungar R, Albright L, Tkatch R, Schaeffer J, Wicker ER. Robotic pet use among community-dwelling older adults. J Gerontol B Psychol Sci Soc Sci. 2020 Oct 16;75(9):2018-2028.

Hudson J, Zarling A, Ungar R, Albright L, Tkatch R, Russell D, Schaeffer J, Wicker ER. Older adults’ experiences in a web-based intervention for loneliness. Gerontol Geriatr Med. 2023 Mar;9:23337214231163004.

Hwang J, Wang L, Siever J, Medico TD, Jones CA. Loneliness and social isolation among older adults in a community exercise program: a qualitative study. Aging Ment Health. 2019 Jun 3;23(6):736–42.

Ibarra F, Baez M, Cernuzzi L, Casati F. A systematic review on technology–supported interventions to improve old–age social well-being: loneliness, social isolation, and connectedness. J Healthc Eng. 2020;2020:2036842.

Ibrahim AF, Tan MP, Teoh GK, Muda SM, Chong MC. Health benefits of social participation interventions among community-dwelling older persons: a review article. Exp Aging Res. 2022 May 27;48(3):234-60.

Irvine KN, Fisher D, Marselle MR, Currie M, Colley K, Warber SL. Social isolation in older adults: a qualitative study on the social dimensions of group outdoor health walks. Int J Environ Res Public Health. 2022 Apr 28;19(9):5353.

Isaacson M, Cohen I, Shpigelman CN. Leveraging emotional well-being and social engagement of the oldest old by using advanced communication technologies: a pilot study using uniper-care’s technology. In: 3rd IET International Conference on Technologies for Active and Assisted Living (TechAAL 2019), London, UK, March 2019.

Isabet B, Pino M, Lewis M, Benveniste S, Rigaud AS. Social telepresence robots: a narrative review of experiments involving older adults before and during the COVID-19 pandemic. Int J Environ Res Public Health. 2021 Mar 30;18(7):3597.

Jackman PC, Lane A, Allen-Collinson J, Henderson H. Older adults' and service providers' experiences of a settings-based health promotion initiative in English football. Health Promot Int. 2023 Jun 1;38(3):daad027.

Jansen-Kosterink SM, Bergsma J, Francissen A, Naafs A. The first evaluation of a mobile application to encourage social participation for community-dwelling older adults. Health Technol. 2020 Sep;10(5):1107-13.

Jarvis MA, Chipps J, Padmanabhanunni A. “This phone saved my life”: older persons’ experiences and appraisals of an mHealth intervention aimed at addressing loneliness. J Psychol Afr. 2019 Mar 4;29(2):159-66.

Jarvis MA, Padmanabhanunni A, Chipps J. An evaluation of a low-intensity cognitive behavioral therapy mHealth-supported intervention to reduce loneliness in older people. Int J Environ Res Public Health. 2019 Apr;16(7):1305.

Jeste DV, Glorioso DK, Depp CA, Lee EE, Daly RE, Jester DJ, Palmer BW, Mausbach BT. Remotely administered resilience-and wisdom-focused intervention to reduce perceived stress and loneliness: pilot controlled clinical trial in older adults. Am J Geriatr Psychiatry. 2023 Jan 1;31(1):58-64.

Jing L, Jin Y, Zhang X, Wang F, Song Y, Xing F. The effect of Baduanjin qigong combined with CBT on physical fitness and psychological health of elderly housebound. Medicine. 2018 Dec;97(51).

Johansson-Pajala RM, Gusdal A, Eklund C, Florin U, Wågert PVH. A codesigned web platform for reducing social isolation and loneliness in older people: a feasibility study. Inform Health Soc Care. 2023 Apr 3;48(2):109-124.

Johnson JK, Louhivuori J, Stewart AL, Tolvanen A, Ross L, Era P. Quality of life (QOL) of older adult community choral singers in Finland. Int Psychogeriatr. 2013 Jul;25(7):1055-64.

Johnson JK, Stewart AL, Acree M, Nápoles AM, Flatt JD, Max WB, et al. A community choir intervention to promote well-being among diverse older adults: results from the Community of Voices Trial. J Gerontol. 2020;75:549–59.

Johnson L, Hardy S. Isolation and loneliness: pilot of a coffee morning hosted in a general practice. Pract Nurs. 2020 Oct 2;31(10):428-33.

Jones RB, Ashurst EJ, Atkey J, Duffy B. Older people going online: its value and before-after evaluation of volunteer support. J Med Internet Res. 2015 May 18;17(5):e122.

Jones VK, Hanus M, Yan C, Shade MY, Boron JB, Bicudo RM. Reducing loneliness among aging adults: the roles of personal voice assistants and anthropomorphic interactions. Front Public Health. 2021 Dec;9.

Juang C, Huh JT, Iyer S, Beaudreau SA, Gould CE. Feasibility, acceptance, and initial evaluation of a telephone-based program designed to increase socialization in older veterans. J Geriatr Psychiatry Neurol. 2021 Nov;34(6):594-605.

Juang C, Knight BG, Carlson M, Schepens Niemiec SL, Vigen C, Clark F. Understanding the Mechanisms of Change in a Lifestyle Intervention for Older Adults. Gerontologist. 2018 Mar 19;58(2):353–61.

Judges RA, Laanemets C, Stern A, Baecker RM. “InTouch” with seniors: exploring adoption of a simplified interface for social communication and related socioemotional outcomes. Comput Human Behav. 2017;75:912-21.

Jung H, Lee JE. The impact of community-based eHealth self-management intervention among elderly living alone with hypertension. J Telemed Telecare. 2017 Jan;23(1):167-73.

Jung Y, Li KJ, Janissa NS, Gladys WL, Lee KM. Games for a better life: effects of playing Wii games on the well-being of seniors in a long-term care facility. In Proceedings of the Sixth Australasian Conference on Interactive Entertainment 2009 Dec 17 (pp. 1-6).

Juris JJ, Bouldin ED, Uva K, Cardwell CD, Schulhoff A, Hiegl N. Virtual intergenerational reverse-mentoring program reduces loneliness among older adults: results from a pilot evaluation. Int J Environ Res Public Health. 2022 Jun 10;19(12):7121.

Kahlbaugh PE, Sperandio AJ, Carlson AL, Hauselt J. Effects of playing Wii on well-being in the elderly: physical activity, loneliness, and mood. Activities Adaptation Aging. 2011;35:331–344.

Kamegaya T, Araki Y, Kigure H, Long‐Term‐Care Prevention Team of Maebashi City, Yamaguchi H. Twelve‐week physical and leisure activity programme improved cognitive function in community‐dwelling elderly subjects: a randomized controlled trial. Psychogeriatrics. 2014 Mar;14(1):47-54.

Kamei T, Itoi W, Kajii F, Kawakami C, Hasegawa M, Sugimoto T. Six month outcomes of an innovative weekly intergenerational day program with older adults and school‐aged children in a Japanese urban community. Japan J Nurs Sci. 2011 Jun;8(1):95-107.

Karlsson E, Axelsson K, Zingmark K, Fahlander K, Sävenstedt S. Supporting conversations between individuals with dementia and their family members. J Gerontol Nurs. 2014;40:38–46.

Kattenstroth JC, Kalisch T, Holt S, Tegenthoff M, Dinse HR. Six months of dance intervention enhances postural, sensorimotor, and cognitive performance in elderly without affecting cardio-respiratory functions. Front Aging Neurosci. 2013 Feb 26;5:5.

Keisari S, Palgi Y, Yaniv D, Gesser-Edelsburg A. Participation in life-review playback theater enhances mental health of community-dwelling older adults: a randomized controlled trial. Psychol Aesthet Creat Arts. 2022;16(2):302–317.

Khosravi P, Ghapanchi AH. Investigating the effectiveness of technologies applied to assist seniors: a systematic literature review. Int J Med Inform. 2016 Jan 1;85(1):17-26.

Khosravi P, Rezvani A, Wiewiora A. The impact of technology on older adults’ social isolation. Comput Human Behav. 2016 Oct 1;63:594-603.

Kim J, Gray J. Qualitative evaluation of an intervention program for sustained internet use among low-income older adults. Ageing Int. 2016 Sep;41:240-53.

Kim JE, Lee YL, Chung MA, Yoon HJ, Shin DE, Choi JH, Lee S, Kim HK, Nam EW. Effects of social prescribing pilot project for the elderly in rural area of South Korea during COVID‐19 pandemic. Health Sci Rep. 2021 Sep;4(3):e320.

Kim S, Yao W, Du X. Exploring older adults’ adoption and use of a tablet computer during COVID-19: longitudinal qualitative study. JMIR Aging. 2022 Mar 8;5(1):e32957.

Kirk L, Eull D, Flaten C, Paun O. Combating social isolation in older adults: an intergenerational nursing communication project. J Psychosoc Nurs Ment Health Serv. 2023 Oct;61(10):7-11.

Kleinberger R, Rieger A, Sands J, Baker J. Supporting elder connectedness through cognitively sustainable design interactions with the memory music box. In Proceedings of the 32nd Annual ACM Symposium on User Interface Software and Technology 2019 Oct 17 (pp. 355-369).

Kocken PL, Voorham AJ. Effects of a peer-led senior health education program. Patient Educ Couns. 1998 May 1;34(1):15-23.

Koga M. The music making and wellness project. Am Music Teacher. 2001 Oct 1;51(2):18.

Kohut ML, McCann DA, Russell DW, Konopka DN, Cunnick JE, Franke WD, et al. Aerobic exercise, but not flexibility/resistance exercise, reduces serum IL-18, CRP, and IL-6 independent of β-blockers, BMI, and psychosocial factors in older adults. Brain Behav Immun. 2006;20:201–9.

Komatsu H, Yagasaki K, Saito Y, Oguma Y. Regular group exercise contributes to balanced health in older adults in Japan: a qualitative study. BMC Geriatr. 2017;17.

Kotwal AA, Fuller SM, Myers JJ, Hill D, Tha SH, Smith AK, M Perissinotto C. A peer intervention reduces loneliness and improves social well-being in low-income older adults: A mixed-methods study. J Am Geriatr Soc. 2021 Dec;69(12):3365-3376.

Kremers IP, Steverink N, Albersnagel FA, Slaets JP. Improved self-management ability and well-being in older women after a short group intervention. Aging Ment Health. 2006 Sep 1;10(5):476-84.

Kumar AB, Zide BS, Bhardwaj T, Lipschitz JM, Altman AN, Donovan NJ. Evaluating feasibility, value and characteristics of an intergenerational friendly telephone visit program during the Covid-19 pandemic. Am J Geriatr Psychiatry. 2023 May 1;31(5):341-52.

Kuru Alıcı N, Zorba Bahceli P, Emiroğlu ON. The preliminary effects of laughter therapy on loneliness and death anxiety among older adults living in nursing homes: a nonrandomised pilot study. Int J Older People Nurs. 2018 Dec;13(4):e12206. doi: 10.1111/opn.12206. Epub 2018 Jul 13. PMID: 30004172.

Lai DW, Li J, Ou X, Li CY. Effectiveness of a peer-based intervention on loneliness and social isolation of older Chinese immigrants in Canada: a randomized controlled trial. BMC Geriatr. 2020 Dec;20:1-2.

Lapena C, Continente X, Sánchez Mascuñano A, Mari dell’Olmo M, López MJ. Effectiveness of a community intervention to reduce social isolation among older people in low-income neighbourhoods. Eur J Public Health. 2022 Oct 1;32(5):677-83.

Lapena C, Continente X, Sanchez Mascunano A, Pons Vigues M, Pujol Ribera E, López MJ. Qualitative evaluation of a community-based intervention to reduce social isolation among older people in disadvantaged urban areas of Barcelona. Health Soc Care Community. 2020 Sep;28(5):1488-503.

Larkey LK, James T, Han S, James DL. Pilot study of Qigong/Tai Chi Easy acute effects of meditative movement, breath focus and “flow” on blood pressure, mood and oxytocin in older adults. Complement Ther Med. 2023 Mar 1;72:102918

Larsen, R.T., Korfitsen, C.B., Keller, C., Christensen, J., Andersen, H.B., Juhl, C., et al. The MIPAM trial – motivational interviewing and physical activity monitoring to enhance the daily level of physical activity among older adults – a randomized controlled trial. Eur Rev Aging Phys Act. 2021;18 (1), 12.

Larsson E, Padyab M, Larsson-Lund M, Nilsson I. Effects of a social internet-based intervention programme for older adults: an explorative randomised crossover study. Br J Occup Ther. 2016 Oct;79(10):629-36.

Larsson, E., 2016. Promoting social activities and participation among seniors: exploring and evaluating social and internet-based occupational therapy interventions. (Doctoral thesis, comprehensive summary)Umeå University, Umeå.

Lee K, Fields N, Cassidy J, Kusek V, Feinhals G, Calhoun M. Caring callers: the impact of the telephone reassurance program on homebound older adults during COVID-19. Home Health Care Serv Q. 2021 Oct-Dec;40(4):247-261.

Lee OE, Kim D, Lee H, Beum KA. Information communication technology use to improve ehealth literacy, technophobia, and social connection among community dwelling older adults. Educ Gerontol. 2022;48(10):445-457.

Lee OE, Kim DH. Bridging the digital divide for older adults via intergenerational mentor-up. Res Soc Work Pract. 2019 Oct;29(7):786-95

Lee OE, Lee H, Park A, Choi NG. My Precious friend: human-robot interactions in home care for socially isolated older adults. Clin Gerontol. 2023 Dec;47(1):161-170.

Lester H, Mead N, Graham CC, Gask L, Reilly S. An exploration of the value and mechanisms of befriending for older adults in England. Ageing Soc. 2012 Feb;32(2):307-28.

Leszko M. The role of online communication among spousal caregivers of individuals with Alzheimer’s disease. J Women Aging. 2020 Jul 3;32(4):462-80.

Levasseur M, Lefebvre H, Levert MJ, Lacasse-Bédard J, Desrosiers J, Therriault PY, Tourigny A, Couturier Y, Carbonneau H. Personalized citizen assistance for social participation (APIC): a promising intervention for increasing mobility, accomplishment of social activities and frequency of leisure activities in older adults having disabilities. Arch Gerontol Geriatr. 2016 May-Jun;64:96-102.

Levinger P, Panisset M, Dunn J, Haines T, Dow B, Batchelor F, Biddle S, Duque G, Hill KD. Exercise interveNtion outdoor proJect in the cOmmunitY for older people - results from the ENJOY Seniors Exercise Park project translation research in the community. BMC Geriatr. 2020 Nov 4;20(1):446.

Li J, Erdt M, Chen L, Cao Y, Lee SQ, Theng YL. The social effects of exergames on older adults: systematic review and metric analysis. J Med Internet Res. 2018 Jun 28;20(6):e10486.

Li J, Xu X, Pham TP, Theng YL, Katajapuu N, Luimula M. Exergames designed for older adults: a pilot evaluation on psychosocial well-being. Games Health J. 2017 Dec 1;6(6):371-8.

Li S, Dai Y, Zhou Y, Zhang J, Zhou C. Efficacy of group reminiscence therapy based on Chinese traditional festival activities (CTFA-GRT) on loneliness and perceived stress of rural older adults living alone in China: a randomized controlled trial. Aging Ment Health. 2022 Jul 3;26(7):1377-84.

Lieberman MA, Goldstein BA. Self-help on-line: An outcome evaluation of breast cancer bulletin boards. J Health Psychol. 2005;10:855–862.

Lim J. Effects of a cognitive-based intervention program using social robot PIO on cognitive function, depression, loneliness, and quality of life of older adults living alone. Front Public Health. 2023 Feb 6;11:313.

Lim JW, Park HO, Kim MJ. Effects of safety and care services on psychological outcomes and housing satisfaction in Korean middle-aged and older adults living alone. J Public Health. 2023 Jul 13:fdad118.

Lindblad K, De Boise S. Musical engagement and subjective well-being amongst men in the third age. Nordic J Music Ther. 2020 Jan;29(1):20–38.

Littlewood E, McMillan D, Graham CC, Bailey D, Gascoyne S, Sloane C, Burke L, Coventry P, Crosland S, Fairhurst C, Henry A. Can we mitigate the psychological impacts of social isolation using behavioural activation? Long-term results of the UK BASIL urgent public health COVID-19 pilot randomised controlled trial and living systematic review. BMJ Ment Health. 2022 Dec 1;25(e1):e49-57.

Liu CW, Hsieh PL, Yang SY, Lin YL, Wang JY. The effectiveness of facilitator-led remote interactive intervention for loneliness, quality of life, and social support among seniors in communities during the COVID-19 pandemic: a randomized controlled experiment. Psychol Health. 2023 Sep 21:1-14.

Liu SJ, Lin CJ, Chen YM, Huang XY. The effects of reminiscence group therapy on self-esteem, depression, loneliness and life satisfaction of elderly people living alone. Mid-Taiwan J Med. 2007 Sep 1;12(3):133-42.

Liu YWJ, Tsui CM. A randomized trial comparing Tai Chi with and without cognitive-behavioral intervention (CBI) to reduce fear of falling in community-dwelling elderly people. Arch Gerontol Geriatr. 2013;59:317–25.

Loe, M. The digital life history project: intergenerational collaborative research. Gerontol Geriatr Educ. 2013;34:26–42.

Long A, Di Lorito C, Logan P, Booth V, Howe L, Hood-Moore V, van der Wardt V. The impact of a dementia-friendly exercise class on people living with dementia: A mixed-methods study. Int J Environ Res Public Health. 2020 Jun;17(12):4562.

Long EM. Promoting connection in the faith community through letter writing. J Christ Nurs. 2023 Oct 1;40(4):260-5.

Lorente-Martínez R, Brotons-Rodes P, Sitges-Maciá E. Benefits of a psychosocial intervention programme using volunteers for the prevention of loneliness among older women living alone in Spain. Health Soc Care Community. 2022 Sep;30(5):2000-2012.

Lou VW, Cheng CY, Ng DK, Chan FH, Mo SS, Kung EK, Luk JK. A mHealth-supported volunteer-assisted spiritual well-being intervention for discharged older patients: a tripartite collaboration. J Gerontol Soc Work. 2023 Feb-Mar;66(2):189-207. doi: 10.1080/01634372.2022.2068722. Epub 2022 Apr 27. PMID: 35477345.

Low LF, Baker JR, Harrison F, Jeon YH, Haertsch M, Camp C, Skropeta M. The lifestyle engagement activity program (leap): implementing social and recreational activity into case-managed home care. J Am Med Dir Assoc. 2015 Dec;16(12):1069-76.

Lowe JA, Collins T, Sallis P. ‘Getting old is not all bingo and knitting.’ An exploration of positive ageing and occupational participation through engagement with community leisure activities: a qualitative study. Int J Ther Rehabil. 2023 Sep 2;30(9):1-1.

Lunt C, Shiels C, Dowrick C, Lloyd-Williams M. Outcomes for older people with long-term conditions attending day care services delivered by paid staff or volunteers: a comparative study. Palliat Care Soc Pract. 2021 Jul;15:26323524211030283.

Machesney D, Wexler SS, Chen T, Coppola JF. Gerontechnology companion: virtual pets for dementia patients. In: Proceedings of the IEEE Long Island Systems, Applications and Technology (LISAT) Conference. Farmingdale, NY, USA; 2014. p. 1–3.

MacIntyre I, Corradetti P, Roberts J, Browne G, Watt S, Lane A. Pilot study of a visitor volunteer programme for community elderly people receiving home health care. Health Soc Care Community. 1999;7(3):225–232.

Mahoney DF, Tarlow BJ, Jones RN. Effects of an automated telephone support system on caregiver burden and anxiety: findings from the reach for TLC intervention study. The Gerontologist. 2003;43:556–567.

Mahoney N, Wilson NJ, Buchanan A, Milbourn B, Hoey C, Cordier R. Older male mentors: outcomes and perspectives of an intergenerational mentoring program for young adult males with intellectual disability. Health Promot J Austr. 2020 Jan;31(1):16-25.

Maki Y, Ura C, Yamaguchi T, Murai T, Isahai M, Kaiho A, et al. Effects of intervention using a community-based walking program for prevention of mental decline: a randomized controlled trial. J Am Geriatr Soc. 2012;60:505–10.

Malaktaris A, Lang AJ, Casmar P, Baca S, Hurst S, Jeste DV, Palmer BW. Pilot study of compassion meditation training to improve well-being among older adults. Clin Gerontol. 2022 Mar 15;45(2):287-300.

Malyn BO, Thomas Z, Ramsey-Wade CE. Reading and writing for well-being: a qualitative exploration of the therapeutic experience of older adult participants in a bibliotherapy and creative writing group. Couns Psychother Res. 2020 Dec;20(4):715-24.

Marquez DX, Bustamante EE, Aguiñaga S, Hernandez R. BAILAMOS: development, pilot testing, and future directions of a latin dance program for older Latinos. Health Educ Behav. 2015 Oct;42(5):604-10.

Martina CM, Stevens NL, Westerhof GJ. Change and stability in loneliness and friendship after an intervention for older women. Ageing Soc. 2018 Mar;38(3):435-54.

Martina CM, Stevens NL. Breaking the cycle of loneliness? Psychological effects of a friendship enrichment program for older women. Aging Ment Health. 2006 Sep 1;10(5):467-75.

Masi CM, Chen HY, Hawkley LC, Cacioppo JT. A meta-analysis of interventions to reduce loneliness. Pers Soc Psychol Rev. 2011 Aug;15(3):219-66.

Masoud SS, Meyer KN, Martin Sweet L, Prado PJ, White CL. “We don't feel so alone”: a qualitative study of virtual memory cafés to support social connectedness among individuals living with dementia and care partners during COVID-19. Front Public Health. 2021 May 13;9:660144

Matz-Costa C, Lubben J, Lachman ME, Lee H, Choi YJ. A pilot randomized trial of an intervention to enhance the health-promoting effects of older adults' activity portfolios: The Engaged4Life Program. J Gerontol Soc Work. 2018 Nov-Dec;61(8):792-816.

Maya Cobalt Angio Septianingtyas, Vivi Sovianti, Arni Nur Rahmawati. The effect of plant therapy on the level of lonely elderly at the wreda house. Indonesian J Community Health Nurs. 2023 Feb 28;8(1):23–8.

Mays AM, Kim S, Rosales K, Au T, Rosen S. The Leveraging Exercise to Age in Place (LEAP) study: engaging older adults in community-based exercise classes to impact loneliness and social isolation. Am J Geriatr Psychiatry. 2021 Aug;29(8):777-788.

McAuley E, Blissmer B, Marquez DX, Jerome GJ, Kramer AF, Katula J. Social relations, physical activity, and well-being in older adults. Prev Med. 2000 Nov 1;31(5):608-17.

McDaid D, Park AL. addressing loneliness in older people through a personalized support and community response program. J Aging Soc Policy. 2023 Jun 29:1-17.

McEwan RT, Davison N, Forster DP, Pearson P, Stirling E. Screening elderly people in primary care: a randomized controlled trial. Br J Gen Pract. 1990 Mar 1;40(332):94-7.

McGovern J. Improving undergraduate competence in multicultural gerontology practice with fresh pedagogies: a digital storytelling case example. Gerontol. Geriatr. Educ. 2019;40:508–518.

McKay H, Nettlefold L, Bauman A, Hoy C, Gray SM, Lau E, et al. Implementation of a co-designed physical activity program for older adults: positive impact when delivered at scale. BMC Public Health. 2018 Nov;18(1):1289.

McKay HA, Macdonald HM, Nettlefold L, Weatherson K, Gray SM, Bauman A, Khan KM, Sims Gould J. What is the 'voltage drop' when an effective health promoting intervention for older adults-Choose to Move (Phase 3)-Is implemented at broad scale? PLoS One. 2023 May 5;18(5):e0268164.

McKay HA, Nettlefold L, Sims-Gould J, Macdonald HM, Khan KM, Bauman A. Status Quo or Drop-Off: Do older adults maintain benefits from choose to move—A Scaled-Up Physical Activity Program—12 Months After Withdrawing the Intervention? J. Phys. Act. Health. 2021 Aug 18;18(10):1236-44.

McKechnie V, Barker C, Stott J. The effectiveness of an internet support forum for carers of people with dementia: a pre-post cohort study. J. Med. Internet Res. 2014 Feb 28;16(2):e68.

McKeon G, Tiedemann A, Sherrington C, Teasdale S, Mastrogiovanni C, Wells R, Steel Z, Rosenbaum S. Feasibility of an online, mental health-informed lifestyle program for people aged 60+ years during the COVID-19 pandemic. Health Promot. J. Austr. 2022 Jul;33(3):545-552.

McNeely ME, Mai MM, Duncan RP, Earhart GM. Differential effects of tango versus dance for pd in Parkinson disease. Front. Aging Neurosci. 2015 Dec 21;7:239.

Melin AL, Bygren LO. Perceived functional health of frail elderly in a primary home care programme and correlation of self-perception with objective measurements. Scand. J. Soc. Med. 1993;21(4):256–263.

Mellor D, Firth L, Moore K. Can the internet improve the well-being of the elderly? Ageing Int. 2008 Mar;32:25-42.

Mendoza-Ruvalcaba NM, Fernández-Ballesteros R. Effectiveness of the vital aging program to promote active aging in Mexican older adults. Clin. Interv. Aging. 2016;11:1631–44.

Merchant RA, Tsoi CT, Tan WM, Lau W, Sandrasageran S, Arai H. Community-based peer-led intervention for healthy ageing and evaluation of the ‘HAPPY’ program. J. Nutr. Health Aging. 2021 Apr;25:520-7.

Merom D, Grunseit A, Eramudugolla R, Jefferis B, Mcneill J, Anstey KJ. Cognitive benefits of social dancing and walking in old age: the dancing mind randomized controlled trial. Front. Aging Neurosci. 2016 Feb 22;8:26.

Merom D, Mathieu E, Cerin E, Morton RL, Simpson JM, Rissel C, Anstey KJ, Sherrington C, Lord SR, Cumming RG. Social dancing and incidence of falls in older adults: a cluster randomised controlled trial. PLoS Med. 2016 Aug 30;13(8):e1002112.

Middling S, Bailey J, Maslin-Prothero S, Scharf T. Gardening and the social engagement of older people. Work. Older People. 2011 Sep 16;15(3):112-22.

Milligan C, Payne S, Bingley A, Cockshott Z. Place and well-being: shedding light on activity interventions for older men. Ageing Soc. 2015 Jan;35(1):124–49.

Moffatt S, Steer M, Lawson S, Penn L, O’Brien N. Link Worker social prescribing to improve health and well-being for people with long-term conditions: qualitative study of service user perceptions. BMJ Open. 2017 Jul;7(7):e015203.

Moieni M, Seeman TE, Robles TF, Lieberman MD, Okimoto S, Lengacher C, Irwin MR, Eisenberger NI. Generativity and social well-being in older women: expectations regarding aging matter. J. Gerontol. B Psychol. Sci. Soc. Sci. 2021 Feb 1;76(2):289-94.

Montoro-Rodriguez J, Hayslip B, Ramsey J. a proactive behavioral activities program (EWA) and the influence of covid-19 among seniors in congregate living communities. J. Appl. Gerontol. 2022 Oct;41(10):2214-2225.

Moody E, Phinney A. A community-engaged art program for older people: fostering social inclusion. Can J Aging. 2012 Mar;31(1):55–64.

Morganti L, Scaratti C, Cipresso P, Gaggioli A, Bonfiglio S, Riva G. How can technology help intergenerational reminiscence? A pilot study. Int J Web Based Commun. 2016;12(1):35-54.

Morgenstern LB, Adelman EE, Hughes R, Wing JJ, Lisabeth LD. The women independently living alone with a medical alert device (WILMA) trial. Transl Stroke Res. 2015 Oct;6:355-60.

Morris ME, Adair B, Ozanne E, Kurowski W, Miller KJ, Pearce AJ, Santamaria N, Long M, Ventura C, Said CM. Smart technologies to enhance social connectedness in older people who live at home. Australas J Ageing. 2014 Sep;33(3):142-52.

Morrow-Howell N, Becker-Kemppainen S, Judy L. Evaluating an intervention for the elderly at increased risk of suicide. Res Social Work Pract. 1998 Jan;8(1):28-46.

Morrow-Howell N, Lee YS, McCrary S, McBride A. Volunteering as a pathway to productive and social engagement among older adults. Health Educ Behav. 2014 Oct;41(1_suppl):84S-90S.

Morse LA, Xiong L, Ramirez-Zohfeld V, Anne S, Barish B, Lindquist LA. Humor doesn’t retire: improvisation as a health-promoting intervention for older adults. Arch Gerontol Geriatr. 2018;75:1–5.

Morton TA, Wilson N, Haslam C, Birney M, Kingston R, McCloskey LG. Activating and guiding the engagement of seniors with online social networking: experimental findings from the AGES 2.0 project. J Aging Health. 2018 Jan;30(1):27-51.

Mountain G, Windle G, Hind D, Walters S, Keertharuth A, Chatters R, Sprange K, Craig C, Cook S, Lee E, Chater T, Woods R, Newbould L, Powell L, Shortland K, Roberts J. A preventative lifestyle intervention for older adults (lifestyle matters): a randomised controlled trial. Age Ageing. 2017 Jul 1;46(4):627-634.

Mountain GA, Hind D, Gossage-Worrall R, Walters SJ, Duncan R, Newbould L, Rex S, Jones C, Bowling A, Cattan M, Cairns A. ‘Putting Life in Years’(PLINY) telephone friendship groups research study: pilot randomised controlled trial. Trials. 2014 Dec;15(1):1-2.

Mulligan MA, Bennett R. Assessment of mental health and social problems during multiple friendly visits: the development and evaluation of a friendly visiting program for the isolated elderly. Int J Aging Hum Dev. 1978 Jan;8(1):43-65.

Mullins LB, Skemp L, Reed D, Emerson M. internet programming to reduce loneliness and social isolation in aging. Res Gerontol Nurs. 2020 Sep 1;13(5):233-242.

Murray M, Crummett A. ‘I don’t think they knew we could do these sorts of things’: social representations of community and participation in community arts by older people. J Health Psychol. 2010 Jul;15(5):777–85.

Mutrie N, Doolin O, Fitzsimons CF, Grant PM, Granat M, Grealy M, et al. Increasing older adults’ walking through primary care: results of a pilot randomized controlled trial. Fam Pract. 2012;29(6):633–642.

Myhre JW, Mehl MR, Glisky EL. Cognitive benefits of online social networking for healthy older adults. J Gerontol B Psychol Sci Soc Sci. 2017 Sep 1;72(5):752-760.

Naseri C, Beilby J, Vaz S, Jacques A, Xu D, Garswood L, O'Connell H, Hill AM. CONNECT 60+: a wellness program for older adults delivered from a community hub. Prog Community Health Partnersh. 2023;17(2):193-205.

Neil-Sztramko SE, Coletta G, Dobbins M, Marr S. Impact of the AGE-ON Tablet training program on social isolation, loneliness, and attitudes toward technology in older adults: single-group pre-post study. JMIR Aging. 2020 Apr 20;3(1):e18398.

Neves BB, Franz RL, Munteanu C, Baecker R. Adoption and feasibility of a communication app to enhance social connectedness amongst frail institutionalized oldest old: an embedded case study. Inf Commun Soc. 2018 Nov 2;21(11):1681-99.

Newall NEG, Menec VH. Targeting Socially isolated older adults: a process evaluation of the senior centre without walls social and educational program. J Appl Gerontol. 2015 Dec;34(8):958–76.

Ng KST, Sia A, Ng MKW, Tan CTY, Chan HY, Tan CH, Rawtaer I, Feng L, Mahendran R, Larbi A, Kua EH, Ho RCM. Effects of horticultural therapy on asian older adults: a randomized controlled trial. Int J Environ Res Public Health. 2018 Aug 9;15(8):1705.

Ng YL, Hill KD, Burton E. Experiences of older adults with mild balance dysfunction who participated in a supervised seniors exercise park program progressing to independent practice. J Aging Phys Act. 2023 Jan 13;31(4):600-610.

Ngiam NH, Yee WQ, Teo N, Yow KS, Soundararajan A, Lim JX, Lim HA, Tey A, Tang KW, Tham CY, Tan JP. Building digital literacy in older adults of low socioeconomic status in singapore (Project Wire Up): nonrandomized controlled trial. J Med Internet Res. 2022 Dec 2;24(12):e40341.

Nguyen LT, Prophater LE, Fazio S, Hülür G, Tate R, Sherwin K, Shatzer J, Peterson LJ, Haley WE. Project VITAL at home: impact of technology on loneliness and well-being of family caregivers of people with dementia. Clin Gerontol. 2022 Oct 27:1-2.

Nicholson NR, Shellman J. Decreasing Social isolation in older adults: effects of an empowerment intervention offered through the CARELINK program. Res Gerontol Nurs. 2013 Apr;6(2):89–97.

Niemann AL, Million RM. Addressing social isolation and loneliness in older adults at the health plan level: a quality improvement project. Geriatr Nurs. 2023 Sep-Oct;53:307-309.

Noble LW, Olson E, Woodall T, Jones J, Smythe T, Whitlock C, Silver M, Hewitt L, Lanou AJ. The social bridging project: intergenerational phone-based connections with older adults during the covid-19 pandemic. Gerontol Geriatr Med. 2022 Apr 4;8:23337214221083473.

Noguchi T, Sato M, Saito T. An approach to psychosocial health among middle-aged and older people by remote sharing of photos and videos from family members not living together: a feasibility study. Front Public Health. 2022 Nov 10;10:962977.

Nolan RW, Friedman S, Carson J, Gibb Z, Acklin C, Reed PS. Exploring the impact of The NEST collaborative's remote social intervention on feelings of depression and isolation. Gerontol Geriatr Med. 2022 Sep 20;8:23337214221125357.

Nomura K, Kobayashi N. Developing a group program for older males to participate in social activities in japan: a mixed-methods study. Am J Mens Health. 2021 Mar-Apr;15(2):1557988321989899.

Noone C, McSharry J, Smalle M, Burns A, Dwan K, Devane D, Morrissey EC. Video calls for reducing social isolation and loneliness in older people: a rapid review. Cochrane Database Syst Rev. 1996 Sep 1;2020(7).

Nurmi MA, Mackenzie CS, Roger K, Reynolds K, Urquhart J. Older men's perceptions of the need for and access to male-focused community programmes such as Men's Sheds. Ageing Soc. 2018 Apr;38(4):794-816.

O’Connor MF, Arizmendi BJ, Kaszniak AW. Virtually supportive: a feasibility pilot study of an online support group for dementia caregivers in a 3D virtual environment. J Aging Stud. 2014 Aug;30:87–93.

O’Leary MF, Barreto M, Bowtell JL. Evaluating the effect of a home-delivered meals service on the physical and psychological well-being of a uk population of older adults – a pilot and feasibility study. J Nutr Gerontol Geriatr. 2020 Jan 2;39(1):1–15.

O’Rourke HM, Collins L, Sidani S. Interventions to address social connectedness and loneliness for older adults: a scoping review. BMC Geriatr. 2018 Dec;18(1):1-3.

O’Shea E, Léime ÁN. The impact of the Bealtaine arts programme on the quality of life, well-being and social interaction of older people in Ireland. Ageing Soc. 2012 Jul;32(5):851–72.

O’Toole L, Ryder R, Connor R, Yurick L, Hegarty F, Connolly D. Impact of a dance programme on health and well-being for community dwelling adults aged 50 years and over. Phys Occup Ther Geriatr. 2015 Oct 2;33(4):303-19.

Oetzel JG, Cameron MP, Simpson ML, Reddy R, Nock S, Greensill H, Meha P, Johnston K, Harding T, Shelford P, Smith LT, Hokowhitu B. Kaumātua Mana Motuhake: peer education intervention to help Māori elders during later-stage life transitions. BMC Geriatr. 2020 May 29;20(1):186.

Ojha, H., Yadav, N.P., 2016. Effects of some yogic practices on psychological well-being of the aged: an intervention study. J Indian Acad Appl Psychol. 42 (2), 291–298.

Ollonqvist K, Palkeinen H, Aaltonen T, Pohjolainen T, Puukka P, Hinkka K, Pöntinen S. Alleviating loneliness among frail older people–findings from a randomised controlled trial. Int J Ment Health Promot. 2008 May 1;10(2):26-34.

Orellana K, Manthorpe J, Tinker A. Day centres for older people - attender characteristics, access routes and outcomes of regular attendance: findings of exploratory mixed methods case study research. BMC Geriatr. 2020 May 4;20(1):158.

Ottoni CA, Sims-Gould J, McKay HA. Video for knowledge translation: engaging older adults in social and physical activity. Can J Aging. 2020 Mar;39(1):31–41.

Owen L, Nolan K, Tierney R, Pritchard C, Leng G. Cost-effectiveness of a befriending intervention to improve the well-being and reduce loneliness of older women. The Lancet. 2016 Nov;388:S84.

Ožić S, Vasiljev V, Ivković V, Bilajac L, Rukavina T. Interventions aimed at loneliness and fall prevention reduce frailty in elderly urban population. Medicine (Baltimore). 2020 Feb;99(8):e19145.

Pacheco E, Hoyos DP, Watt WJ, Lema L, Arango CM. Feasibility study: Colombian Caribbean folk dances to increase physical fitness and health-related quality of life in older women. J Aging Phys Act. 2016 Apr;24(2):284-9.

Pandya SP. Meditation program mitigates loneliness and promotes well-being, life satisfaction and contentment among retired older adults: a two-year follow-up study in four South Asian cities. Aging Ment Health. 2021 Feb;25(2):286-298.

Pandya SP. Spiritual counselling mitigates loneliness and promotes affect balance for older empty nester couples: a study in some international cities. Couns Psychother Res. 2021 Jun;21(2):269-80.

Panigrahi M, Shree P, Swain DP. Effect of integrated approach of yoga therapy on loneliness in elderly: an interventional study. Biomedicine. 2023 Feb 26;43(1):47-51.

Parisi JM, Kuo J, Rebok GW, Xue QL, Fried LP, Gruenewald TL, Huang J, Seeman TE, Roth DL, Tanner EK, Carlson MC. Increases in lifestyle activities as a result of experience Corps® participation. J Urban Health. 2015 Feb;92(1):55-66.

Park J, Heilman KJ, Sullivan M, Surage J, Levine H, Hung L, Ortega M, Wiese LA, Ahn H. Remotely supervised home-based online chair yoga intervention for older adults with dementia: feasibility study. Complement Ther Clin Pract. 2022 Aug 1;48:101617.

Park M, Sung K. Effects of a self-care reinforcement program for socially vulnerable elderly women with metabolic syndrome in Korea. J Korean Acad Community Health Nurs. 2019;30(3):271.

Parkinson D, Turner J. Alleviating social isolation through intergenerational programming: DOROT’s Summer Teen Internship program. J Intergenerational Relationships. 2019 Jul;17(3):388–95.

Parlak MM, Bizbinar Ö, Köse A. The effect of holistic therapy in alzheimer’s disease. Altern Ther Health Med. 2023 Apr 1;29(3).

Pauly T, Lay JC, Kozik P, Graf P, Mahmood A, Hoppmann CA. Technology, physical activity, loneliness, and cognitive functioning in old age. GeroPsych. 2019 Aug 22.

Pedersen MT, Nørregaard LB, Jensen TD, Frederiksen AS, Ottesen L, Bangsbo J. The effect of 5 years of team sport on elderly males' health and social capital—An interdisciplinary follow‐up study. Health Sci Rep. 2022 Sep;5(5):e760.

Pepin R, Stevens CJ, Choi NG, Feeney SM, Bruce ML. Modifying behavioral activation to reduce social isolation and loneliness among older adults. Am J Geriatr Psychiatry. 2021 Aug 1;29(8):761-70.

Peterat L, Mayersmith J. Farm friends: Exploring intergenerational environmental learning. J Intergenerational Relationships. 2006 Apr 18;4(1):107-16.

Pettigrew S, Roberts M. Addressing loneliness in later life. Aging Ment Health. 2008 May 1;12(3):302-9.

Pierce LL, Steiner VL, Khuder SA, Govoni AL, Horn LJ. The effect of a web-based stroke intervention on carers' well-being and survivors' use of healthcare services. Disabil Rehabil. 2009;31:1676–1684.

Pinheiro, H.A., Cerceau, V.R., Pereira, L.C., Funghetto, S.S., Menezes, Rld. Nutritional intervention and functional exercises improve depression, loneliness and quality of life in elderly women with sarcopenia: a randomized clinical trial. Fisioterapia em Movimento. 2020;33.

Pollak C, Wexler SS, Drury L. Effect of a Robotic pet on social and physical frailty in community-dwelling older adults: a randomized controlled trial. Res Gerontol Nurs. 2022 Sep 1;15(5):229-37.

Poscia A, Stojanovic J, La Milia DI, Duplaga M, Grysztar M, Moscato U, Onder G, Collamati A, Ricciardi W, Magnavita N. Interventions targeting loneliness and social isolation among the older people: an update systematic review. Exp Gerontol. 2018 Feb 1;102:133-44.

Preston C, Moore S. Ringing the changes: the role of telephone communication in a helpline and befriending service targeting loneliness in older people. Ageing Soc. 2019 Jul;39(7):1528-51.

Pynnönen K, Törmäkangas T, Rantanen T, Tiikkainen P, Kallinen M. Effect of a social intervention of choice vs. control on depressive symptoms, melancholy, feeling of loneliness, and perceived togetherness in older Finnish people: a randomized controlled trial. Aging Ment Health. 2018 Jan 2;22(1):77-84.

Rejeski WJ, Spring B, Domanchuk K, Tao H, Tian L, Zhao L, McDermott MM. A group-mediated, home-based physical activity intervention for patients with peripheral artery disease: effects on social and psychological function. J Transl Med. 2014 Jan 28;12:29.

Ren Y, Tang R, Sun H, Li X. Intervention effect of group reminiscence therapy in combination with physical exercise in improving spiritual well-being of the elderly. Iran J Public Health. 2021 Mar;50(3):531-539.

Rendon AA, Lohman EB, Thorpe D, Johnson EG, Medina E, Bradley B. The effect of virtual reality gaming on dynamic balance in older adults. Age Ageing. 2012 Jul 1;41(4):549-52.

Reynolds KA, Mackenzie CS, Medved M, Roger K. The experiences of older male adults throughout their involvement in a community programme for men. Ageing Soc. 2015 Mar;35(3):531-51.

Ring L, Barry B, Totzke K, Bickmore T. Addressing loneliness and isolation in older adults: proactive affective agents provide better support. in: 2013 humaine association conference on affective computing and intelligent interaction. IEEE; 2013. p. 61–6.

Ring L, Shi L, Totzke K, Bickmore T. Social support agents for older adults: longitudinal affective computing in the home. J Multimodal User Interf. 2015;9(1):79–88.

Ristolainen H, Kannasoja S, Tiilikainen E, Hakala M, Närhi K, Rissanen S. Effects of ‘participatory group-based care management’ on well-being of older people living alone: a randomized controlled trial. Arch Gerontol Geriatr. 2020 Jul;89:104095.

Roberts JR, Windle G. Evaluation of an intervention targeting loneliness and isolation for older people in North Wales. Perspect Public Health. 2020 May;140(3):153-161.

Roberts JS, Ferber RA, Funk CN, Harrington AW, Maixner SM, Porte JL, Schissler P, Votta CM, Deldin PJ, Connell CM. Mood Lifters for seniors: development and evaluation of an online, peer-led mental health program for older adults. Gerontol Geriatr Med. 2022 Aug 10;8:23337214221117431.

Rodriguez Espinosa P, King AC, Blanco-Velazquez I, Banchoff AW, Campero MI, Chen WT, Rosas LG. Engaging diverse midlife and older adults in a multilevel participatory physical activity intervention: evaluating impacts using Ripple Effects Mapping. Transl Behav Med. 2023 Sep 12;13(9):666-674.

Rodríguez-Romero R, Herranz-Rodríguez C, Kostov B, Gené-Badia J, Sisó-Almirall A. Intervention to reduce perceived loneliness in community-dwelling older people. Scand J Caring Sci. 2021 Jun;35(2):366-374.

Roland H, Ilin Shpilkerman Y, Schaub J, Comeau AC. Connection through calls: The impact of a seniors center without walls on older adults’ social isolation and loneliness. Gerontol Geriatr Med. 2021 Dec 11;7:23337214211063102.

Rook KS, Sorkin DH. Fostering social ties through a volunteer role: implications for older-adults’ psychological health. Int J Aging Hum Dev. 2003;57(4):313–337.

Rosenberg D, Depp CA, Vahia IV, Reichstadt J, Palmer BW, Kerr J, Norman G, Jeste DV. Exergames for subsyndromal depression in older adults: a pilot study of a novel intervention. Am J Geriatr Psychiatry. 2010 Mar 1;18(3):221-6.

Ross JM, Sanchez A, Epps JB, Arikawa A, Wright l. the Impact of a Food Recovery-Meal Delivery Program on Homebound Seniors' Food Security, Nutrition, and Well-Being. J Nutr Gerontol Geriatr. 2022 Apr-Jun;41(2):175-189.

Routasalo PE, Tilvis RS, Kautiainen H, Pitkala KH. Effects of psychosocial group rehabilitation on social functioning, loneliness and well-being of lonely, older people: randomized controlled trial. J Adv Nurs. 2009 Feb;65(2):297–305.

Sadarangani T, Missaelides L, Eilertsen E, Jaganathan H, Wu B. A mixed-methods evaluation of a nurse-led community-based health home for ethnically diverse older adults with multimorbidity in the adult day health setting. Policy, Politics, & Nursing Practice. 2019 Aug;20(3):131–44.

Saghaee A, Ghahari S, Nasli-Esfahani E, Sharifi F, Alizadeh-Khoei M, Rezaee M. Evaluation of the effectiveness of Persian diabetes self-management education in older adults with type 2 diabetes at a diabetes outpatient clinic in Tehran: a pilot randomized control trial. J Diabetes Metab Disord. 2020 Nov 11;19(2):1491-1504.

Saito T, Kai I, Takizawa A. Effects of a program to prevent social isolation on loneliness, depression, and subjective well-being of older adults: a randomized trial among older migrants in Japan. Arch Gerontol Geriatr. 2012 Nov 1;55(3):539-47.

Sakurai R, Yasunaga M, Murayama Y, Ohba H, Nonaka K, Suzuki H, Sakuma N, Nishi M, Uchida H, Shinkai S, Rebok GW. Long-term effects of an intergenerational program on functional capacity in older adults: results from a seven-year follow-up of the REPRINTS study. Arch Gerontol Geriatr. 2016 May 1;64:13-20.

Samoocha D, Snels IAK, Bruinvels DJ, Anema JR, van der Beek AJ. Effectiveness of an interactive website aimed at empowerment of disability benefit claimants: results of a pragmatic randomized controlled trial. J Occup Rehabil. 2011;21:410–420.

Sandu S, Sreedhar S, Chang L, Cohen L, Cruz A, Olson HR, Sreedhar R, Gomez K, Carrion A. 21st Century Good Neighbor program: an easily generalizable program to reduce social isolation in older adults. Front Public Health. 2021 Dec 20;9:766706.

Santos-Olmo AB, Ausín B, Muñoz M. People over 65 years old in social isolation: description of an effective community intervention in the city of Madrid (Spain). Int J Environ Res Public Health. 2022 Feb 25;19(5):2665.

Savikko N, Routasalo P, Tilvis R, Pitkälä K. Psychosocial group rehabilitation for lonely older people: favourable processes and mediating factors of the intervention leading to alleviated loneliness. Int J Older People Nurs. 2010 Mar;5(1):16–24.

Savolainen L, Hanson E, Magnusson L, Gustavsson T. An Internet-based videoconferencing system for supporting frail elderly people and their carers. J Telemed Telecare. 2008;14(2):79–82.

Sayied NE, Abd-Elaziz NM. Effect of counseling sessions as a nursing intervention on depression and loneliness among elderly at AssiutCity. IOSR J Nurs Health Sci. 2015;4(6):16–22.

Scharlach AE, Graham CL, Berridge C. An integrated model of co-ordinated community-based care. The Gerontologist. 2015 Aug 1;55(4):677-87.

Schmidt T, Pawlowski CS, Kerr J, Schipperijn J. Investigating the WHAT and WHY on older adults’ use of neighborhood open spaces following an environmental intervention. Transl Behav Med. (2021) 11:582–96.

Schoales C, Jones H, Jones-Bonofiglio K, Stroink M. Older adults’ experience of digital storytelling on the perceptions of well-being. Perspectives. 2020;41:13–21.

Schwei RJ, Hetzel S, Kim K, Mahoney J, DeYoung K, Frumer J, Lanzafame RP, Madlof J, Simpson A, Zambrano-Morales E, Jacobs EA. Peer-to-peer support and changes in health and well-being in older adults over time. JAMA Netw Open. 2021 Jun 1;4(6):e2112441-.

Seelye AM, Wild KV, Larimer N, Maxwell S, Kearns P, Kaye JA. Reactions to a remote-controlled video-communication robot in seniors' homes: a pilot study of feasibility and acceptance. Telemed e-Health. 2012 Dec 1;18(10):755-9.

Sehrawat S, Jones CA, Orlando J, Bowers T, Rubins A. Digital storytelling: A tool for social connectedness. Gerontechnology. 2017 Jan 1;16(1).

Seinfeld S, Figueroa H, Ortiz-Gil J, Sanchez-Vives MV. Effects of music learning and piano practice on cognitive function, mood and quality of life in older adults. Front Psychol. 2013 Nov 1;4:810.

Seino S, Nishi M, Murayama H, Narita M, Yokoyama Y, Nofuji Y, Taniguchi Y, Amano H, Kitamura A, Shinkai S. Effects of a multifactorial intervention comprising resistance exercise, nutritional and psychosocial programs on frailty and functional health in community‐dwelling older adults: a randomized, controlled, cross‐over trial. Geriatr Gerontol Int. 2017 Nov;17(11):2034-45.

Seo JH, Sungkajun A, Garcia B. Developing the art-technology intergenerational community program for older adults' health and social connectedness. Front Public Health. 2021 Jun 23;9:589589.

Shah SG, Nogueras D, van Woerden HC, Kiparoglou V. Evaluation of the effectiveness of digital technology interventions to reduce loneliness in older adults: systematic review and meta-analysis. J Med Internet Res. 2021 Jun 4;23(6):e24712.

Shanahan J, Bhriain ON, Morris ME, Volpe D, Clifford AM. Irish set dancing classes for people with Parkinson's disease: The needs of participants and dance teachers. Complement Ther Med. 2016 Aug;27:12-7.

Shanahan J, Morris ME, Bhriain ON, Volpe D, Lynch T, Clifford AM. Dancing for Parkinson disease: a randomized trial of Irish set dancing compared with usual care. Arch Phys Med Rehabil. 2017 Sep;98(9):1744-1751.

Shapira S, Cohn-Schwartz E, Yeshua-Katz D, Aharonson-Daniel L, Clarfield AM, Sarid O. Teaching and practicing cognitive-behavioral and mindfulness skills in a web-based platform among older adults through the COVID-19 pandemic: a pilot randomized controlled trial. Int J Environ Res Public Health. 2021 Oct 9;18(20):10563.

Shapira S, Yeshua-Katz D, Cohn-Schwartz E, Aharonson-Daniel L, Sarid O, Clarfield AM. A pilot randomized controlled trial of a group intervention via Zoom to relieve loneliness and depressive symptoms among older persons during the COVID-19 outbreak. Internet Interv. 2021 Apr;24:100368.

Shinokawa S, Abe H, Takashima R, Onishi R, Hirano M. Verification of the effectiveness of a communication application in improving social connectedness and physical health among unacquainted older men: a mixed-methods pilot study. Int J Environ Res Public Health. 2023 Jan 19;20(3):1884.

Shvedko AV, Thompson JL, Greig CA, Whittaker AC. Physical activity intervention for loneliness (PAIL) in community-dwelling older adults: a randomised feasibility study. Pilot Feasibility Study. 2020;6:73.

Sidner CL, Bickmore T, Nooraie B, et al. Creating new technologies for companionable agents to support isolated older adults. ACM Trans Interact Intell Syst. 2018;8(3):1–27.

Siette J, Jorgensen M, Nguyen A, Knaggs G, Miller S, Westbrook JI. A mixed-methods study evaluating the impact of an excursion-based social group on quality of life of older adults. BMC Geriatr. 2021 Jun 10;21(1):356.

Simpson ML, Oetzel J, Nock S, Greensill HI, Meha P, Reddy R, Johnston K, Cameron M, Harding T, Shelford P, Smith LT. Māori becoming peer educators in later life: impacts on identity, well-being, and social connectedness. J Gerontol B Psychol Sci Soc Sci. 2021 Jul 1;76(6):1140-50.

Skingley A, De'Ath S, Napleton L. Evaluation of EDNA: arts and dance for older people. Work Older People. 2016 Mar 14;20(1):46-56.

Slegers K, van Boxtel MP, Jolles J. Effects of computer training and Internet usage on the well-being and quality of life of older adults: a randomized, controlled study. J Gerontol B Psychol Sci Soc Sci. 2008 May;63(3):P176-84.

Sljivic, H.; Sutherland, I.; Stannard, C.; Ioppolo, C.; Morrisby, C. Changing attitudes towards older adults: eliciting empathy through digital storytelling. Gerontol. Geriatr. Educ. 2022, 43, 360–373.

Smith ML, Chen E, Lau CA, Davis D, Simmons JW, Merianos AL. Effectiveness of chronic disease self-management education (CDSME) programs to reduce loneliness. Chronic Illness. 2023 Sep;19(3):646-64.

Smith R, Drennan V, Mackenzie A, Greenwood N. The impact of befriending and peer support on family carers of people living with dementia: A mixed methods study. Arch Gerontol Geriatr. 2018 May;76:188–95.

Smith R, Wuthrich V, Johnco C, Belcher J. Effect of group cognitive behavioural therapy on loneliness in a community sample of older adults: a secondary analysis of a randomized controlled trial. Clin Gerontol. 2021 Aug 8;44(4):439-49.

Solé C, Mercadal-Brotons M, Gallego S, Riera M. Contributions of music to aging adults' quality of life. J Music Ther. 2010 Oct 1;47(3):264-81.

Sørensen KH, Sivertsen J. Follow-up three years after intervention to relieve unmet medical and social needs of old people. Compr Gerontol B. 1988 Aug 1;2(2):85-91.

Steinman L, Parrish A, Mayotte C, Bravo Acevedo P, Torres E, Markova M, Boddie M, Lachenmayr S, Montoya CN, Parker L, Conton-Pelaez E, Silsby J, Snowden M. Increasing Social connectedness for underserved older adults living with depression: a pre-post evaluation of PEARLS. Am J Geriatr Psychiatry. 2021 Aug;29(8):828-842.

Stenhouse, R.; Tait, J.; Hardy, P.; Sumner, T. Dangling conversations: reflections on the process of creating digital stories during a workshop with people with early-stage dementia. J Psychiatr Ment Health Nurs. 2013, 20, 134–141.

Stevens NA. Combating loneliness: a friendship enrichment programme for older women. Ageing Soc. 2001 Mar;21(2):183-202.

Stevens NL, Martina CM, Westerhof GJ. Meeting the need to belong: predicting effects of a friendship enrichment program for older women. The Gerontologist. 2006 Aug 1;46(4):495-502.

Stewart M, Craig D, MacPherson K, Alexander S. Promoting positive affect and diminishing loneliness of widowed seniors through a support intervention. Public Health Nurs. 2001;18(1):54–63.

Streber A, Abu-Omar K, Hentschke C, Rütten A. A multicenter controlled study for dementia prevention through physical, cognitive and social activities - GESTALT-kompakt. Clin Interv Aging. (2017) 12:2109–21.

Sun Q, Lou VW, Dai A, To C, Wong SY. The effectiveness of the young–old link and growth intergenerational program in reducing age stereotypes. Research on Social Work Practice. 2019 Jul;29(5):519-28.

Sutherland L, Dunkle RE, Pace GT. Enhancing social connections through an acting and improvisation course for older Americans in low-income housing. Arts & Health. 2023 May 10:1-6.

Sweeney, L.; Wolverson, E.; Clarke, C. Understanding the shared experiences of creating a digital life story with individuals with dementia and their spouse. Dementia 2021, 20, 1791–1813.

Széman Z. A new pattern in long-term care in Hungary: Skype and youth volunteers. Anthropological notebooks. 2014 Mar 30;20(1).

Tan EJ, Xue QL, Li T, Carlson MC, Fried LP. Volunteering: a physical activity intervention for older adults—the experience Corps® program in Baltimore. Journal of Urban Health. 2006 Sep;83:954-69.

Taube, E., Kristensson, J., Midlöv, P., Jakobsson, U. The use of case management for community-dwelling older people: the effects on loneliness, symptoms of depression and life satisfaction in a randomised controlled trial. Scand. J. Caring Sci. 2018;32 (2), 889–901.

Taylor-Piliae RE, Haskell WL, Waters CM, Froelicher ES. Change in perceived psychosocial status following a 12-week Tai Chi exercise programme. J Adv Nurs. 2006 May;54(3):313–29.

Teater B, Baldwin M. Singing for successful ageing: The perceived benefits of participating in the golden oldies community-arts programme. British Journal of Social Work. 2014 Jan 1;44(1):81-99.

Ten Bruggencate T, Luijkx KG, Sturm J. To meet, to matter, and to have fun: the development, implementation, and evaluation of an intervention to fulfil the social needs of older people. IJERPH. 2019 Jun 28;16(13):2307.

Tesch-Römer C. Psychological effects of hearing aid use in older adults. J Gerontol B Psychol Sci Soc Sci. 1997 May;52(3):P127-138.

Theeke LA, Mallow JA, Moore J, McBurney A, Rellick S, VanGilder R. Effectiveness of LISTEN on loneliness, neuroimmunological stress response, psychosocial functioning, quality of life, and physical health measures of chronic illness. International journal of nursing sciences. 2016 Sep 1;3(3):242-51.

Thiel C, Günther L, Osterhoff A, Sommer S, Grüneberg C. Feasibility of smartphone-supported, combined physical and cognitive activities in the Neighbourhood for stimulating social participation of the elderly. BMC Geriatr. 2022 Jul 30;22(1):629.

Thomas KS, Akobundu U, Dosa D. More than a meal? a randomized control trial comparing the effects of home-delivered meals programs on participants’ feelings of loneliness. GERONB. 2016 Nov;71(6):1049–58.

Thomas, B.H., Ciliska, D., Dobbins, M., Micucci, S. A process for systematically reviewing the literature: providing the research evidence for public health nursing interventions. Worldviews Evid.-Based Nurs. 2004;1 (3), 176–184.

Tilburg NS. Stimulating friendship in later life: A strategy for reducing loneliness among older women. Educ Gerontol. 2000 Jan 1;26(1):15-35.

Tkatch R, Wu L, MacLeod S, Ungar R, Albright L, Russell D, Murphy J, Schaeffer J, Yeh CS. Reducing loneliness and improving well-being among older adults with animatronic pets. Aging Ment Health. 2021 Jul;25(7):1239-1245.

Todd C, Camic PM, Lockyer B, Thomson LJM, Chatterjee HJ. Museum-based programs for socially isolated older adults: Understanding what works. Health Place. 2017 Nov;48:47–55.

Tomasino KN, Lattie EG, Ho J, Palac HL, Kaiser SM, Mohr DC. Harnessing peer support in an online intervention for older adults with depression. Am J Geriatr Psychiatry. 2017 Oct;25(10):1109-1119.

Torp S, Hanson E, Hauge S, Ulstein I, Magnusson L. A pilot study of how information and communication technology may contribute to health promotion among elderly spousal carers in Norway. Health Soc Care Community. 2008; 16: 75–85

Tsai HH, Cheng CY, Shieh WY, Chang YC. Effects of a smartphone-based videoconferencing program for older nursing home residents on depression, loneliness, and quality of life: a quasi-experimental study. BMC Geriatr. 2020 Jan 28;20(1):27.

Tsai HH, Tsai YF, Wang HH, Chang YC, Chu HH. Videoconference program enhances social support, loneliness, and depressive status of elderly nursing home residents. Aging Ment Health. 2010 Nov;14(8):947-54.

Tse T, Linsey H. Adult day groups: addressing older people's needs for activity and companionship. Australas J Ageing. 2005 Sep;24(3):134-40.

Unbehaun D, Vaziri DD, Aal K, Wieching R, Tolmie P, Wulf V. Exploring the potential of exergames to affect the social and daily life of people with dementia and their caregivers. In Proceedings of the 2018 chi conference on human factors in computing systems 2018 Apr 19 (pp. 1-15).

Vadineia da Silva M, Alves Lima D, da Conceição Silva TM, de Carvalho Melo TMT, Pereira da Cunha V, Pessoa da Silva MN. Dance: a therapeutic resource in the third age. J Nurs UFPE. 2016;10:232–8.

Valadez AA, Lumadue C, Gutierrez B, de Vries-Kell S. Las Comadres and adult day care centers: the perceived impact of socialization on mental wellness. J Aging Stud. 2006 Jan 1;20(1):39-53.

Van Assche M, Moreels T, Petrovic M, Cambier D, Calders P, Van de Velde D. The role of a socially assistive robot in enabling older adults with mild cognitive impairment to cope with the measures of the COVID-19 lockdown: a qualitative study. Scand J Occup Ther. 2023 Jan;30(1):42-52.

Van Der Heide LA, Willems CG, Spreeuwenberg MD, Rietman J, De Witte LP. Implementation of CareTV in care for the elderly: The effects on feelings of loneliness and safety and future challenges. Technol Disabil. 2012 Dec 12;24(4):283–91.

Van Orden KA, Areán PA, Conwell Y. A pilot randomized trial of engage psychotherapy to increase social connection and reduce suicide risk in later life. Am J Geriatr Psychiatry. 2021 Aug 1;29(8):789-800.

Van Orden KA, Bower E, Lutz J, Silva C. Engage coaching for caregivers: a pilot trial to reduce loneliness in dementia caregivers. Aging Ment Health. 2023 Mar 3:1-8.

Van Rossum E, Frederiks CM, Philipsen H, Portengen K, Wiskerke J, Knipschild P. Effects of preventive home visits to elderly people. BMJ. 1993 Jul 7;307(6895):27.

Van Straten A, Cuijpers P, Smits N. Effectiveness of a web-based selfhelp intervention for symptoms of depression, anxiety, and stress: Randomized controlled trial. J Med Internet Res. 2008;10: e7

Vanoh D, Shahar S, Razali R, Ali NM, Manaf ZA, Mohd Noah SA, Nur AM. the effectiveness of a web-based health education tool, WESIHAT 2.0, among older adults: a randomized controlled trial. J Alzheimers Dis. 2019;70(s1):S255-S270.

Vardoulakis LP, Ring L, Barry B, Sidner CL, Bickmore T. Designing relational agents as long term social companions for older adults. In: International Conference on Intelligent Virtual Agents; 2012;289–302.

Vassilev I, Rogers A, Kennedy A, Oatley C, James E. Identifying the processes of change and engagement from using a social network intervention for people with long‐term conditions. A qualitative study. Health Expectations. 2019 Apr;22(2):173-82.

Veazie S, Gilbert J, Winchell K, Paynter R, Guise JM. Addressing social isolation to improve the health of older adults: a rapid review. AHRQ publication no. 19-EHC009- EF. Rockville, MD: Agency for Healthcare Research and Quality; 2019.

Verghese J, Lipton RB, Katz MJ, Hall CB, Derby CA, Kuslansky G, Ambrose AF, Sliwinski M, Buschke H. Leisure activities and the risk of dementia in the elderly. New England Journal of Medicine. 2003 Jun 19;348(25):2508-16.

Vetter NJ, Jones DA, Victor CR. Effect of health visitors working with elderly patients in general practice: a randomised controlled trial. Br Med J (Clin Res Ed). 1984 Feb 4;288(6414):369-72.

Walters K, Marshall M, Wilkinson AN, Natividad MD. An intentionally designed walking program for seniors results in enhanced community connection. J Aging Phys Act. 2022 Feb 1;30(1):44-53.

Wang DS. Feasibility of a yoga intervention for enhancing the mental well-being and physical functioning of older adults living in the community. Act Adapt Aging. (2010) 34:85–97.

Wang RH, Sudhama A, Begum M, Huq R, Mihailidis A. Robots to assist daily activities: views of older adults with Alzheimer’s disease and their caregivers. Int Psychogeriatr. 2017;29(1):67–79.

Wang S, Reaves S, Newman M, Castaneda S, Emery-Tiburcio E. CATCH-ON Connect: a tablet intervention to address social isolation and loneliness. Aging Ment Health. 2023 Jun 6:1-5.

Ward, A.; Thoft, D.S.; Lomax, H.; Parkes, J. A visual and creative approach to exploring people with dementia’s experiences of being students at a school in Denmark. Dementia 2020, 19, 786–804.

Watson B, Das A, Maguire S, Fleet G, Punamiya A. The little intervention that could: creative aging implies healthy aging among Canadian seniors. Aging Ment Health. 2023 Aug 21:1-12.

Weaver C, Sardina AL, Newsham TM, Fugate-Whitlock E. A preliminary feasibility study of a virtual mentally stimulating activities program for older adults. American Journal of Recreation Therapy. 2022 Dec 1;21(4):17-30.

Weinert C, Cudney S, Comstock B, Bansal A. Computer intervention impact on psychosocial adaptation of rural women with chronic conditions. Nursing Research 2011; 60: 82–91

Weinert C, Cudney S, Hill WG. Rural women, technology, and selfmanagement of chronic illness. The Canadian Journal of Nursing Research 2008; 40: 114–134

Weiss LA, Oude Voshaar MAH, Bohlmeijer ET, Westerhof GJ. The long and winding road to happiness: a randomized controlled trial and cost-effectiveness analysis of a positive psychology intervention for lonely people with health problems and a low socio-economic status. Health Qual Life Outcomes. 2020 Jun 2;18(1):162.

Welch L, Orlando R, Lin SX, Vassilev I, Rogers A. Findings from a pilot randomised trial of a social network self-management intervention in COPD. BMC Pulm Med. 2020 Jun 8;20(1):162.

Weselman T, Naseri C, Vaz S, Beilby J, Garswood L, O’Connell H, Hill AM. Older adults’ experiences of a community wellness program (Connect 60+) that focused on physical activity and social connections: a qualitative exploratory study. Australian Journal of Primary Health. 2022 Nov 3;29(1):64-73.

Westheimer O, McRae C, Henchcliffe C, Fesharaki A, Glazman S, Ene H, Bodis-Wollner I. Dance for PD: a preliminary investigation of effects on motor function and quality of life among persons with Parkinson's disease (PD). J Neural Transm. 2015 Sep;122(9):1263

White H, McConnell E, Clipp E, Branch LG, Sloane R, Pieper C, Box TL. A randomized controlled trial of the psychosocial impact of providing internet training and access to older adults. Aging Ment Health. 2002 Aug;6(3):213-21.

Wikman JM, Nistrup A, Vorup J, Pedersen MT, Melchor PS, Bangsbo J, et al. The effect of floorball training on health status, psychological health and social capital in older men. AIMS Public Health. (2017) 4:364–82.

Wikström BM. Social interaction associated with visual art discussions: a controlled intervention study. Aging & Mental Health. 2002 Feb 1;6(1):82-7.

Wildman JM, Valtorta N, Moffatt S, Hanratty B. ‘What works here doesn’t work there’: the significance of local context for a sustainable and replicable asset‐based community intervention aimed at promoting social interaction in later life. Health Soc Care Community. 2019 Jul;27(4):1102–10.

Wiles J, Morgan T, Moeke-Maxwell T, Black S, Park HJ, Dewes O, Williams LA, Gott M. Befriending services for culturally diverse older people. Journal of gerontological social work. 2019 Oct 3;62(7):776-93.

Wollersheim D, Merkes M, Shields N, Liamputtong P, Wallis L, Reynolds F, Koh L. Physical and psychosocial effects of Wii video game use among older women. International journal of emerging technologies and society. 2010 Jul 1;8(2):85-98.

Woodward AT, Freddolino PP, Blaschke-Thompson CM, Wishart DJ, Bakk L, Kobayashi R, Tupper C. Technology and aging project: training outcomes and efficacy from a randomized field trial. Ageing International. 2011 Mar;36:46-65.

Wright L, Vance L, Sudduth C, Epps JB. The Impact of a Home-Delivered Meal Program on Nutritional Risk, Dietary Intake, Food Security, Loneliness, and Social Well-Being. Journal of Nutrition in Gerontology and Geriatrics. 2015 Apr 3;34(2):218–27.

Wu VX, Yap XY, Tam WS, Goh J, Mok WY, Ramazanu S. Qualitative inquiry of a community dance program for older adults in Singapore. Nursing & Health Sciences. 2023 Sep;25(3):341-53.

Xu L, Fields NL, Chen Z, Zhou A, Merchant A, Zhou A. Big and Mini: a promising intergenerational program for social connections. International journal of environmental research and public health. 2022 Apr 11;19(8):4566.

Xu X, Li J, Pham TP, Salmon CT, Theng YL. Improving psychosocial well-being of older adults through exergaming: the moderation effects of intergenerational communication and age cohorts. Games for health journal. 2016 Dec 1;5(6):389-97.

Yang, S.Y., Yang, C.C., Lee, Y.C., Hsieh, P.L., Lin, Y.L.. Investigating the effectiveness of online interactive courses on loneliness and quality of life of older adults in the community during the COVID-19 pandemic: a pilot study and a randomized controlled trial. Geriatr. Gerontol. Int. 2023;23 (2), 91–97.

Yap AF, Kwan YH, Tan CS, Ibrahim S, Ang SB. Rhythm-centred music making in community living elderly: a randomized pilot study. BMC complementary and alternative medicine. 2017 Dec;17(1):1-8.

Yavuz C, Şahin S. The impact of a videoconferencing‐implemented program on older adults' psychosocial health in the COVID‐19 pandemic: an experimental study. Psychogeriatrics. 2023 Apr 18.

Young TL, Janke MC. Perceived benefits and concerns of older adults in a community intergenerational program: Does race matter? Activities, Adaptation & Aging. 2013 Apr 1;37(2):121-40.

Yu F, Mathiason MA, Johnson K, Gaugler JE, Klassen D. Memory matters in dementia: efficacy of a mobile reminiscing therapy app. Alzheimers Dement (N Y). 2019 Oct 23;5:644-651.

Zaine I, Frohlich D, da Hora Rodrigues KR, da Cunha BC, Orlando AF, Scalco LF, Pimentel MD. Promoting social connection and deepen relations in older people: design of media parcels towards facilitating time-based media sharing. JMIR. 2019;21(10).

Zarling A, Kim J, Russell D, Cutrona C. Online acceptance and commitment therapy as treatment for loneliness among older adults: report of a pilot study. J Am Geriatr Soc. 2023 Mar 27.

Zengin Alpozgen A, Kardes K, Acikbas E, Demirhan F, Sagir K, Avcil E. The effectiveness of synchronous tele-exercise to maintain the physical fitness, quality of life, and mood of older people-a randomized and controlled study. Eur Geriatr Med. 2022 Oct;13(5):1177-85.

Zhu YZ, Lin CF, Yang HL, Jin G, Chiu HL. Effects of exergaming on cognitive functions and loneliness of older adults with cognitive frailty. Int J Geriatr Psychiatry. 2023 Jun;38(6):e5944.

Zuckerman O, Walker D, Grishko A, et al. Companionship is not a function: the effect of a novel robotic object on healthy older adults’ feelings of"being-seen". Proc CHI Conf Hum Factors Comput Syst. New York: Association for Computing Machinery; 2020.

**References of knowledge syntheses:**

Adekpedjou R, Léon P, Dewidar O, Al‐Zubaidi A, Jbilou J, Kaczorowski J, Muscedere J, Hirdes J, Heckman G, Girard M, Hébert PC. Effectiveness of interventions to address different types of vulnerabilities in community‐dwelling older adults: an umbrella review of systematic reviews. Campbell Syst Rev. 2023 Jun;19(2):e1323.

Alici NK, Dönmez AA. A systematic review of the effect of laughter yoga on physical function and psychosocial outcomes in older adults. Complement Ther Clin Pract. 2020 Nov 1;41:101252.

Astasio-Picado Á, Cobos-Moreno P, Gómez-Martín B, Verdú-Garcés L, Zabala-Baños MD. Efficacy of interventions based on the use of information and communication technologies for the promotion of active aging. Int J Environ Res Public Health. 2022 Jan 29;19(3):1534.

Carvalho MI, Póvoa MJ, Neves M, Bernardo J, Loureiro R, Bernardes RA, Almeida IF, Santana E, Silva R. Intergenerationality programs—between children and older adults—for Portuguese Population: A scoping review. Nurs Rep. 2022 Nov 14;12(4):836-49.

Casanova G, Zaccaria D, Rolandi E, Guaita A. The effect of information and communication technology and social networking site use on older people’s well-being in relation to loneliness: review of experimental studies. J Med Internet Res. 2021 Mar 1;23(3):e23588.

Chang H, Do Y, Ahn J. Digital storytelling as an intervention for older adults: a scoping review. Int J Environ Res Public Health. 2023 Jan 11;20(2):1344.

Chao YY, Scherer YK, Montgomery CA. Effects of using Nintendo WiiTM exergames in older adults: a review of the literature. J Aging Health. 2015 Apr;27(3):379–402.

Chen YRR, Schulz PJ. The effect of information communication technology interventions on reducing social isolation in the elderly: a systematic review. J Med Internet Res. 2016 Jan 28;18(1):e18.

Chipps J, Jarvis MA, Ramlall S. The effectiveness of e-Interventions on reducing social isolation in older persons: a systematic review of systematic reviews. J Telemed Telecare. 2017 Dec;23(10):817–27.

Choi HK, Lee SH. Trends and effectiveness of ICT interventions for the elderly to reduce loneliness: a systematic review. Healthcare. 2021;9(3):293.

Chua CMS, Chua JYX, Shorey S. Effectiveness of home-based interventions in improving loneliness and social connectedness among older adults: a systematic review and meta-analysis. Aging Ment Health. 2023 Jul 2;28(1):1–10.

Cohen-Mansfield J, Perach R. Interventions for alleviating loneliness among older persons: a critical review. Am J Health Promot. 2015 Jan;29(3):e109–25.

Cummings SM, Kropf NP, Cassie KM, Bride B. Evidenced-based treatment for older adults. J Evid Based Soc Work. 2004 May;1(4):53–81.

DesChâtelets JR, Khowaja AR, Mechelse K, Koning H, Ventresca D. Exploring the access and use of social technologies by older adults in support of their mental health during the COVID-19 pandemic: a rapid review. Can J Aging. 2023 Sep 20:1-6.

Dickens AP, Richards SH, Greaves CJ, Campbell JL. Interventions targeting social isolation in older people: a systematic review. BMC Public Health. 2011 Dec;11(1):647.

Döring N, Conde M, Brandenburg K, Broll W, Gross HM, Werner S, Raake A. Can communication technologies reduce loneliness and social isolation in older people? A scoping review of reviews. Int J Environ Res Public Health. 2022 Sep 8;19(18):11310.

Douglas NF, Archer B, Azios JH, Strong KA, Simmons-Mackie N, Worrall L. A scoping review of friendship intervention for older adults: lessons for designing intervention for people with aphasia. Disabil Rehabil. 2023 Aug 28;45(18):3012-31.

Freedman A, Nicolle J. Social isolation and loneliness: the new geriatric giants: approach for primary care. Can Fam Physician. 2020 Mar;66(3):176–82.

Gasteiger N, Loveys K, Law M, Broadbent E. Friends from the future: a scoping review of research into robots and computer agents to combat loneliness in older people. Clin Interv Aging. 2021 May 24;16:941-971.

Hagan R, Manktelow R, Taylor BJ, Mallett J. Reducing loneliness amongst older people: a systematic search and narrative review. Aging Ment Health. 2014 Aug;18(6):683–93.

Heins P, Boots LMM, Koh WQ, Neven A, Verhey FRJ, de Vugt ME. The effects of technological interventions on social participation of community-dwelling older adults with and without dementia: a systematic review. J Clin Med. 2021 May 25;10(11):2308.

Hickin N, Käll A, Shafran R, Sutcliffe S, Manzotti G, Langan D. The effectiveness of psychological interventions for loneliness: a systematic review and meta-analysis. Clin Psychol Rev. 2021 Aug 1;88:102066.

Hutchinson TD. Intergenerational programming: insights into program length and type of interaction to achieve health and well-being benefits for older adults. Perspect: J Can Gerontol Nurs Assoc. 2022 Jul 1;43(3).

Ibarra F, Baez M, Cernuzzi L, Casati F. A systematic review on technology-supported interventions to improve old-age social well-being: loneliness, social isolation, and connectedness. J Healthc Eng. 2020 Jul 12;2020.

Ibrahim AF, Tan MP, Teoh GK, Muda SM, Chong MC. Health benefits of social participation interventions among community-dwelling older persons: a review article. Exp Aging Res. 2022 May 27;48(3):234-60.

Isabet B, Pino M, Lewis M, Benveniste S, Rigaud AS. Social telepresence robots: a narrative review of experiments involving older adults before and during the COVID-19 pandemic. Int J Environ Res Public Health. 2021 Mar 30;18(7):3597.

Jarvis MA, Padmanabhanunni A, Balakrishna Y, Chipps J. The effectiveness of interventions addressing loneliness in older persons: an umbrella review. Int J Afr Nurs Sci. 2020;12:100177.

Johnstone G, Dickins M, Lowthian J, Renehan E, Enticott J, Mortimer D, Ogrin R. Interventions to improve the health and well-being of older people living alone: a mixed-methods systematic review of effectiveness and accessibility. Ageing & Society. 2021 Jul;41(7):1587-636.

Khosravi P, Rezvani A, Wiewiora A. The impact of technology on older adults’ social isolation. Comput Human Behav. 2016 Oct;63:594–603.

Krzeczkowska A, Spalding DM, McGeown WJ, Gow AJ, Carlson MC, Nicholls LA. A systematic review of the impacts of intergenerational engagement on older adults’ cognitive, social, and health outcomes. Ageing Res Rev. 2021 Nov 1;71:101400.

Larsson E, Fahlström G, Bertilsson G, Fristedt S. An evidence map of digital tools to support social engagement in older adults living with mental illness or those who are at risk for mental health decline. Gerontechnology. 2020 Jul 1;19(3).

Lestari WA. The effectiveness of technology-based non-clinical community interventions to reduce loneliness in the elderly: a literature review. J Vocat Nurs. 2023;4(1):45-51.

Li J, Erdt M, Chen L, Cao Y, Lee SQ, Theng YL. The social effects of exergames on older adults: systematic review and metric analysis. J Med Internet Res. 2018 Jun;20(6):e10486.

Li M, Rao W, Su Y, Sul Y, Caron G, D’Arcy C, Fleury MJ, Meng X. Psychological interventions for loneliness and social isolation among older adults during medical pandemics: a systematic review and meta-analysis. Age Ageing. 2023 Jun 1;52(6):afad076.

Mao W, Qi X, Chi I, Wichinsky L, Wu B. Technology-based interventions to address social isolation and loneliness among informal dementia caregivers: a scoping review. J Am Med Dir Assoc. 2023 Sep 5.

McQuade L, O'Sullivan R. Examining arts and creativity in later life and its impact on older people's health and well-being: a systematic review of the evidence. Perspect Public Health. 2023 Mar 11:17579139231157533.

Morris ME, Adair B, Ozanne E, Kurowski W, Miller KJ, Pearce AJ, et al. Smart technologies to enhance social connectedness in older people who live at home: smart technology and social connectedness. Australas J Ageing. 2014 Sep;33(3):142–52.

Noone C, Yang K. Community-based responses to loneliness in older people: a systematic review of qualitative studies. Health Soc Care Community. 2022 Jul;30(4):e859-e873.

Paquet C, Whitehead J, Shah R, Adams AM, Dooley D, Spreng RN, Aunio AL, Dubé L. Social prescription interventions addressing social isolation and loneliness in older adults: meta-review integrating on-the-ground resources. J Med Internet Res. 2023 May 17;25:e40213.

Peters R, Ee N, Ward SA, Kenning G, Radford K, Goldwater M, Dodge HH, Lewis E, Xu Y, Kudrna G, Hamilton M. Intergenerational programmes bringing together community dwelling non-familial older adults and children: a systematic review. Arch Gerontol Geriatr. 2021 May 1;94:104356.

Pool MS, Agyemang CO, Smalbrugge M. Interventions to improve social determinants of health among elderly ethnic minority groups: a review. Eur J Public Health. 2017 Dec;27(6):1048–54.

Poscia A, Stojanovic J, La Milia DI, Duplaga M, Grysztar M, Moscato U, et al. Interventions targeting loneliness and social isolation among the older people: an update systematic review. Exp Gerontol. 2018 Feb;102:133–44.

Rivera-Torres S, Mpofu E, Jean Keller M, Ingman S. Older adults’ mental health through leisure activities during COVID-19: a scoping review. Gerontol Geriatr Med. 2021 Sep 7;7:23337214211036776.

Shah SG, Nogueras D, van Woerden HC, Kiparoglou V. Evaluation of the effectiveness of digital technology interventions to reduce loneliness in older adults: systematic review and meta-analysis. J Med Internet Res. 2021 Jun 4;23(6):e24712.

Tcymbal A, Abu-Omar K, Hartung V, Bußkamp A, Comito C, Rossmann C, Meinzinger D, Reimers AK. Interventions simultaneously promoting social participation and physical activity in community living older adults: a systematic review. Front Public Health. 2022 Dec 7;10:1048496.

Todd E, Bidstrup B, Mutch A. Using information and communication technology learnings to alleviate social isolation for older people during periods of mandated isolation: a review. Australas J Ageing. 2022 Sep;41(3):e227-39.

Tricco AC, Thomas SM, Radhakrishnan A, Ramkissoon N, Mitchell G, Fortune J, Jiang Y, de Groh M, Anderson K, Barker J, Gauthier-Beaupré A. Interventions for social isolation in older adults who have experienced a fall: a systematic review. BMJ Open. 2022 Mar 1;12(3):e056540.

van Haastregt JC, Diederiks JP, van Rossum E, de Witte LP, Crebolder HF. Effects of preventive home visits to elderly people living in the community: systematic review. BMJ. 2000 Mar 18;320(7237):754-8.

Yu DS, Li PW, Lin RS, Kee F, Chiu A, Wu W. Effects of non-pharmacological interventions on loneliness among community-dwelling older adults: a systematic review, network meta-analysis, and meta-regression. Int J Nurs Stud. 2023 Aug;144:104524.
